# Tailored SirReal-type inhibitors enhance SIRT2 inhibition through ligand stabilization and disruption of NAD^+^ co-factor binding

**DOI:** 10.1039/d5md00144g

**Published:** 2025-08-19

**Authors:** Ricky Wirawan, Matthias Frei, Anna Heider, Niklas Papenkordt, Florian Friedrich, Thomas Wein, Manfred Jung, Michael Groll, Eva M. Huber, Franz Bracher

**Affiliations:** a Department of Pharmacy, Ludwig-Maximilians University Munich Butenandtstraße 5-13 81377 Munich Germany franz.bracher@cup.uni-muenchen.de; b Center for Protein Assemblies, Technical University of Munich Ernst-Otto-Fischer Straße 8 85748 Garching Germany; c Institute of Pharmaceutical Sciences, Albert-Ludwigs-Universität Freiburg Albertstraße 25 79104 Freiburg im Breisgau Germany

## Abstract

Human sirtuin 2 (SIRT2) is an NAD^+^ dependant enzyme that has been linked to the pathogenesis of various diseases, making it a promising target for pharmaceutical intervention. This study presents a systematic investigation on the inhibitory effects of SIRT2 inhibitors functionalized with diverse electrophilic functional groups. Guided by initial docking studies, we designed and synthesised 14 derivatives of two published potent lead structures 24a and SirReal2. The most potent and subtype selective SIRT2 inhibitor 29 (RW-78) exhibits an IC_50_ of 26 nM, which outperforms its lead structure 24a (IC_50_ = 79 nM) by a factor of 3. The increased potency of 29 is explained by halogen–π interactions with SIRT2 residues as visualized by X-ray crystallography. Furthermore, 29 interferes with NAD^+^ binding, highlighting co-factor displacement as a valid strategy to inhibit SIRT2. Additionally, we showed cellular target engagement *via* NanoBRET assays in HEK293T cells (EC_50_ = 15 nM). Altogether our findings provide a deeper insight into the structure–activity relationships of these SirReal-type inhibitors and offer new avenues for optimisation of SIRT2 inhibitors.

## Abstract 2

The duality of halogen-bonding-mediated ligand stabilization and the inteference of NAD^+^ co-factor binding represents an effective strategy in the development of highly potent SIRT2 inhibitors.

## Graphical abstract

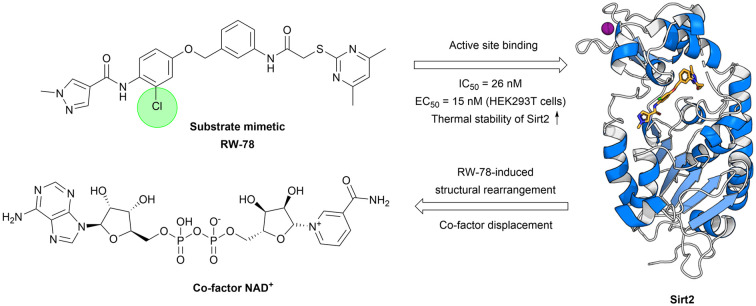

## Introduction

**Fig. 1:**
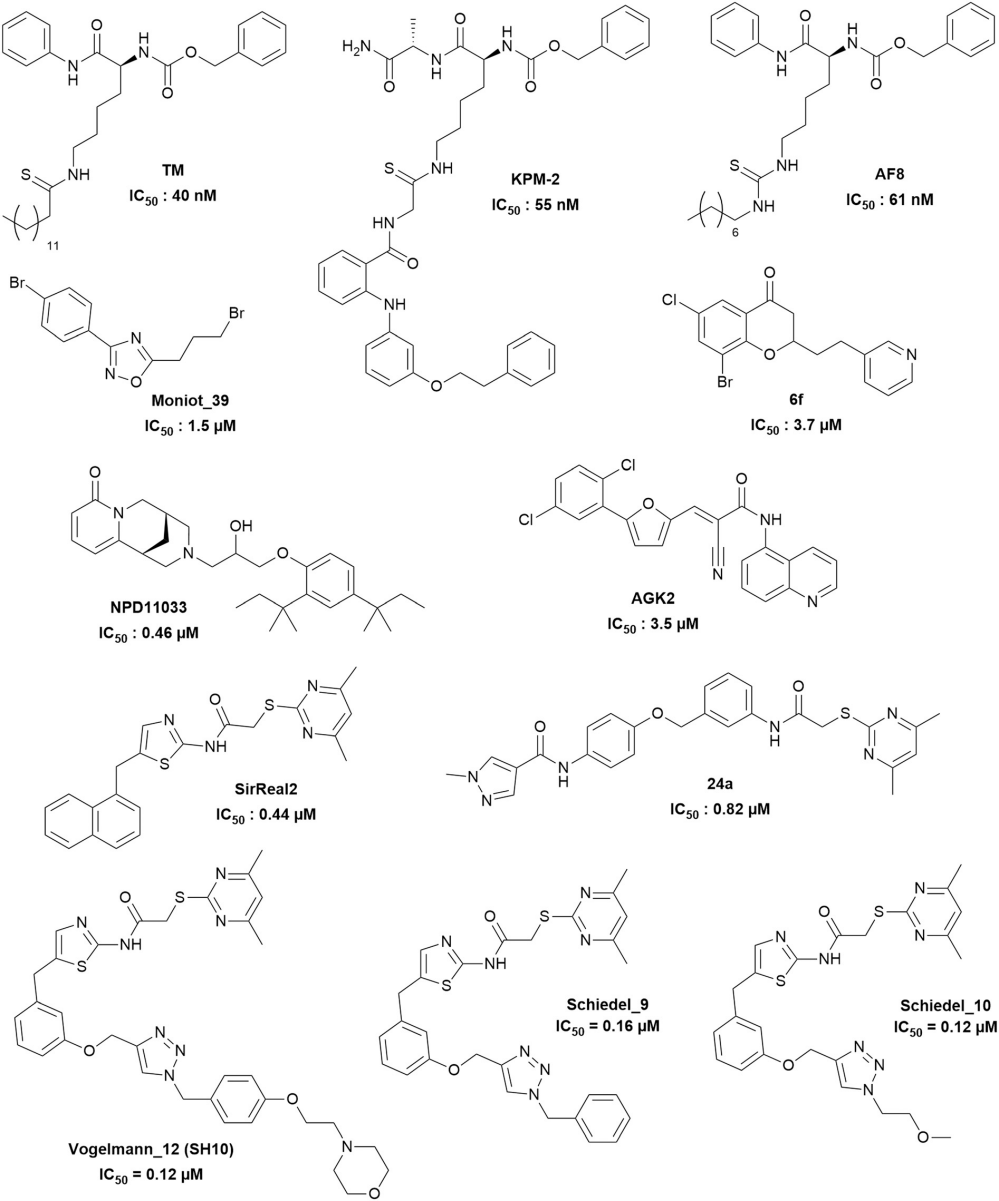
Examples of published SIRT2 inhibitors.

Sirtuins, belonging to class III histone deacetylases, constitute a family of highly conservative NAD^+^-dependant proteins that are involved in the regulation of various biological processes such as metabolism, aging, inflammation and oxidative stress.^1^ Although sirtuins were initially recognised solely for their deacetylation activity,^2^ recent studies unveiled a broader catalytic scope that includes desuccinylation,^3^ demalonylation,^3^ demyristoylation^4^ and ADP-ribosylation.^5^ Furthermore, sirtuin substrates extend beyond histones to include other proteins, such as α-tubulin,^6^ p53 (ref. 7) and NF-κB.^8^ Of the seven sirtuin subtypes, SIRT2 emerged as a key target, particularly due to its involvement in the pathogenesis of cancer,^9^ viral infections^10^ and neurodegenerative diseases.^11,12^ Despite numerous efforts over the past few years, the development of potent and subtype selective SIRT2 inhibitors with satisfactory physicochemical properties still poses a significant challenge. Published SIRT2 inhibitors with drug-like properties have been shown to possess a wide range of structural diversity with various binding mechanisms (Fig. 1). SIRT2 inhibitors with greater potency tend to incorporate thioamide (KPM-2 (ref. 13) and TM^9^) and thiourea (AF8)^14^ scaffolds that can form stable covalent intermediates with the essential co-factor NAD^+^. Additionally, SIRT2 inhibitors with other structural motifs such as oxadiazole (Moniot_39)^15^ and chroman-4-one (6f)^16^ were identified with IC_50_ values covering low micromolar ranges. The alkaloid cytisine derived compound NPD11033 was shown by Kudo *et al.* to likewise be a potent SIRT2 inhibitor.^17^ Further potent SIRT2 inhibitors such as AGK2 were detected by means of high-throughput screening.^18^ Similarly, a series of aminothiazoles were discovered through library screening, in which the term sirtuin rearranging ligands (SirReals) was coined.^19,20^ Among these aminothiazoles, SirReal2 displayed high inhibitory potency with an IC_50_ value of 0.44 μM.^19^ X-ray crystallographic studies on a SIRT2–SirReal2–NAD^+^ complex unveiled a ligand-induced structural rearrangement of the active site that generates the emergence of a selectivity pocket that is occupied by the 4,6-dimethyl-2-mercaptopyrimidine motif. Further optimisations employing triazole motifs led to SIRT2 inhibitors with improved potency (Vogelmann_12 (SH10)^21^ and Schiedel_9 and Schiedel_10^22^). Extending efforts for the development of alternative SIRT2 inhibitors exploiting this selectivity pocket, Yang *et al.* synthesised a library of *N*-acylaniline derivatives that identified 24a as a potent and selective SIRT2 inhibitor with an IC_50_ value of 0.82 μM.^23,24^

## Design rationale

**Fig. 2:**
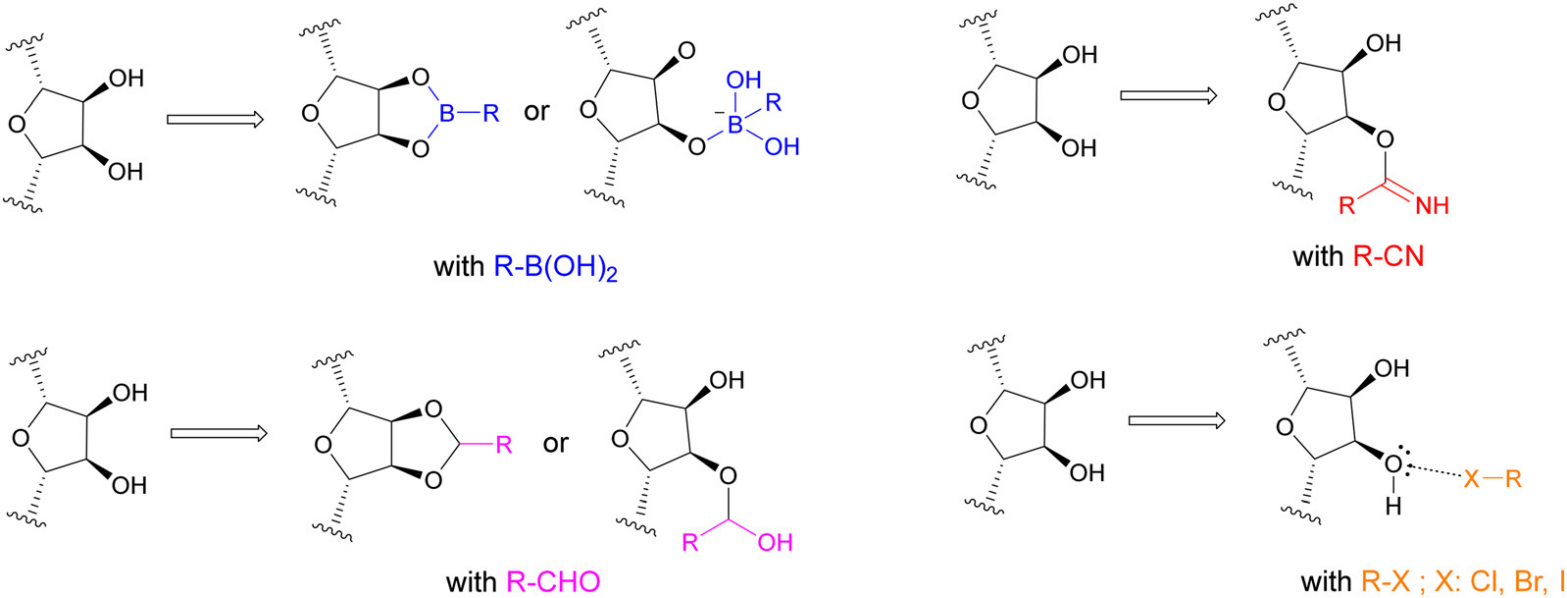
Proposed binding mechanisms and interactions of the selected functional groups with the ribose unit of the co-factor NAD^+^. Boronic acids can undergo reversible covalent bonding with one or both hydroxy groups to form (cyclic) boronates. Nitriles can form iminoethers, and aldehydes can form hemiacetals or cyclic acetals. Halogen residues such as chlorine, bromine and iodine can form halogen bonds with the ribose hydroxy groups.

Based on the published co-crystal structures of SIRT2 with either 24a (PDB ID: 5YQO)^23^ or SirReal2 and NAD^+^ (PDB ID: 4RMG),^19^ we hypothesised that significant increase in the potency of these lead structures can be achieved through strong, targeted interactions with the vicinal hydroxy groups of the ribose unit of NAD^+^ by utilizing appropriate functional groups that enable reversible covalent binding or halogen bonding. Although reactive warheads such as Michael acceptors, β-lactones and β-lactams, epoxides and sulfonyl fluorides have seen application in drug development and present themselves as attractive irreversible covalent binders, the potential for immunogenic reactions and off-target toxicity is almost inevitable.^25–27^ In contrast, reversible covalent inhibitors can dissociate from off-targets, reducing the potential of unwanted side effects while retaining extended binding at the intended target protein.^28^ Several reversible covalent inhibitors have been successfully approved as therapeutic drugs, such as the boronic acid bortezomib that inhibits the 20S proteasome for the treatment of multiple myeloma,^29,30^ the nitrile-based dipeptidylpeptidase 4 (DPP4) inhibitor saxagliptin for the treatment of type 2 diabetes mellitus,^31^ and the aldehyde-bearing voxelotor for the treatment of sickle cell anaemia.^32^ Functional groups such as boronic acids, nitriles and aldehydes do not only possess the capability to undergo reversible covalent bonding, but can also form strong non-covalent interactions in the form of hydrogen bonding, *e.g.* with the ribose hydroxy groups of the co-factor NAD^+^. Comparably, non-covalent interactions *via* halogen bonding, which is sometimes referred to as the hydrophobic equivalent of hydrogen bonding,^33^ represent an area of interest in current rational drug design approaches as demonstrated by the optimisation of PDE5 (ref. 34) and HIV-1 reverse transcriptase inhibitors^35,36^ and offer, as such, a promising drug optimisation technique. This non-covalent interaction arises from the anisotropic electron distribution in halogen residues, creating a σ-hole with depleted electron density that enables strong bonding with diverse nucleophiles.^37–40^ By tailoring lead structures 24a and SirReal2 with such functional groups, we aimed to achieve, in a (to our knowledge) unprecedented manner in the sirtuin field, reversible covalent binding and strong halogen bonding with the co-factor NAD^+^ of SIRT2 that strives for significant enhancement in potency (Fig. 2).

**Fig. 3:**
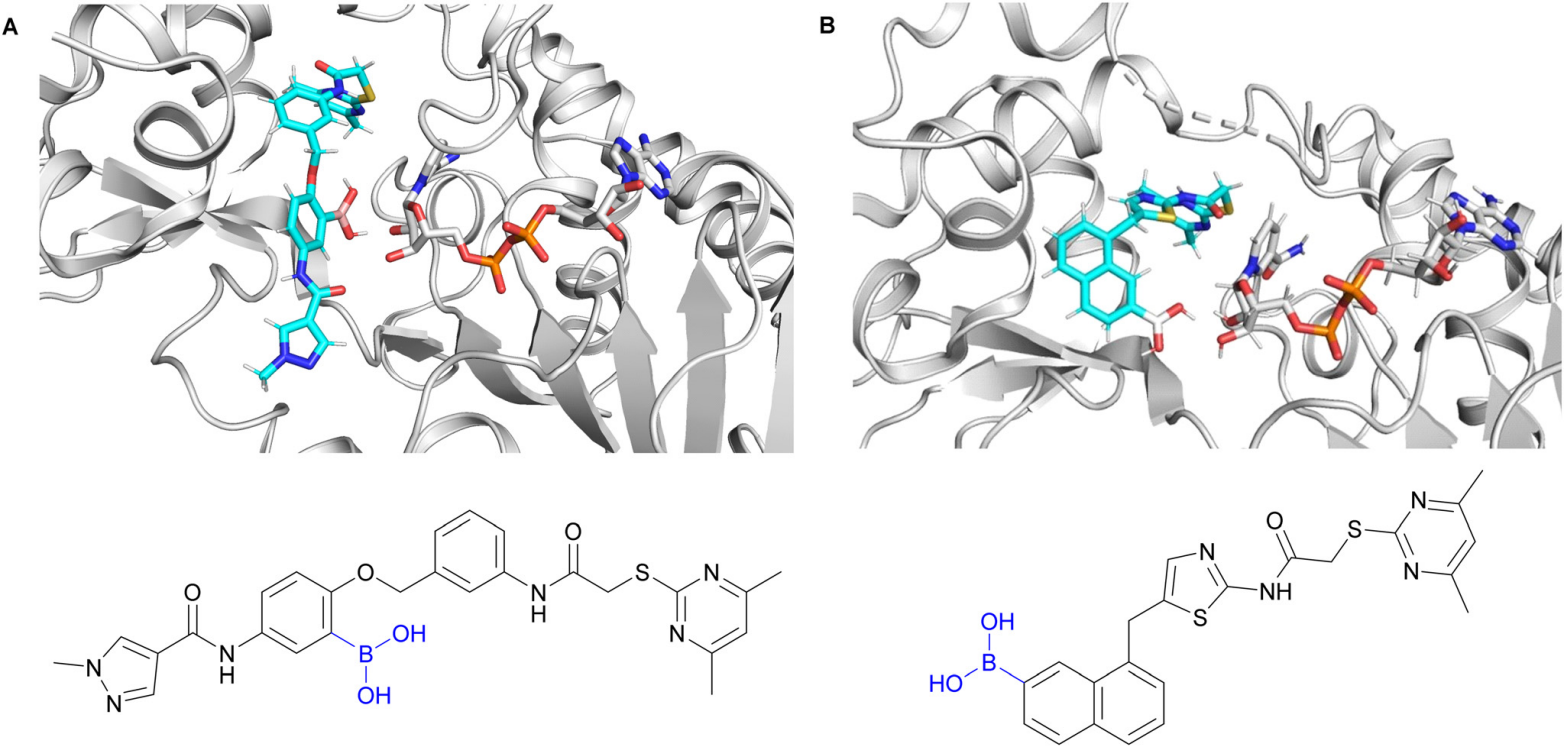
(A) Docking poses of the envisaged 24a boronic acid derivative (cyan) based on PDB ID: 5YQO^23^ (NAD^+^ shown was extracted separately from PDB ID: 4RMG^19^) and (B) SirReal2 boronic acid derivative (cyan) based on PDB ID: 4RMG^19^ in the presence of NAD^+^ showing the boronic acids in proximity to the vicinal diol unit of ribose of NAD^+^ for reversible covalent bonding.

Encouraged by initial docking studies of the envisaged boronic acid derivatives of 24a and SirReal2 that showed poses of the boronic acid moiety in proximity to the vicinal diol unit of NAD^+^ (Fig. 3), we continued our efforts^41^ in developing highly potent and sub-type selective SIRT2 inhibitors through systematic investigation of the effects of such functional groups on these two selected lead structures.

## Results and discussion

### Chemistry

**Scheme 1:**
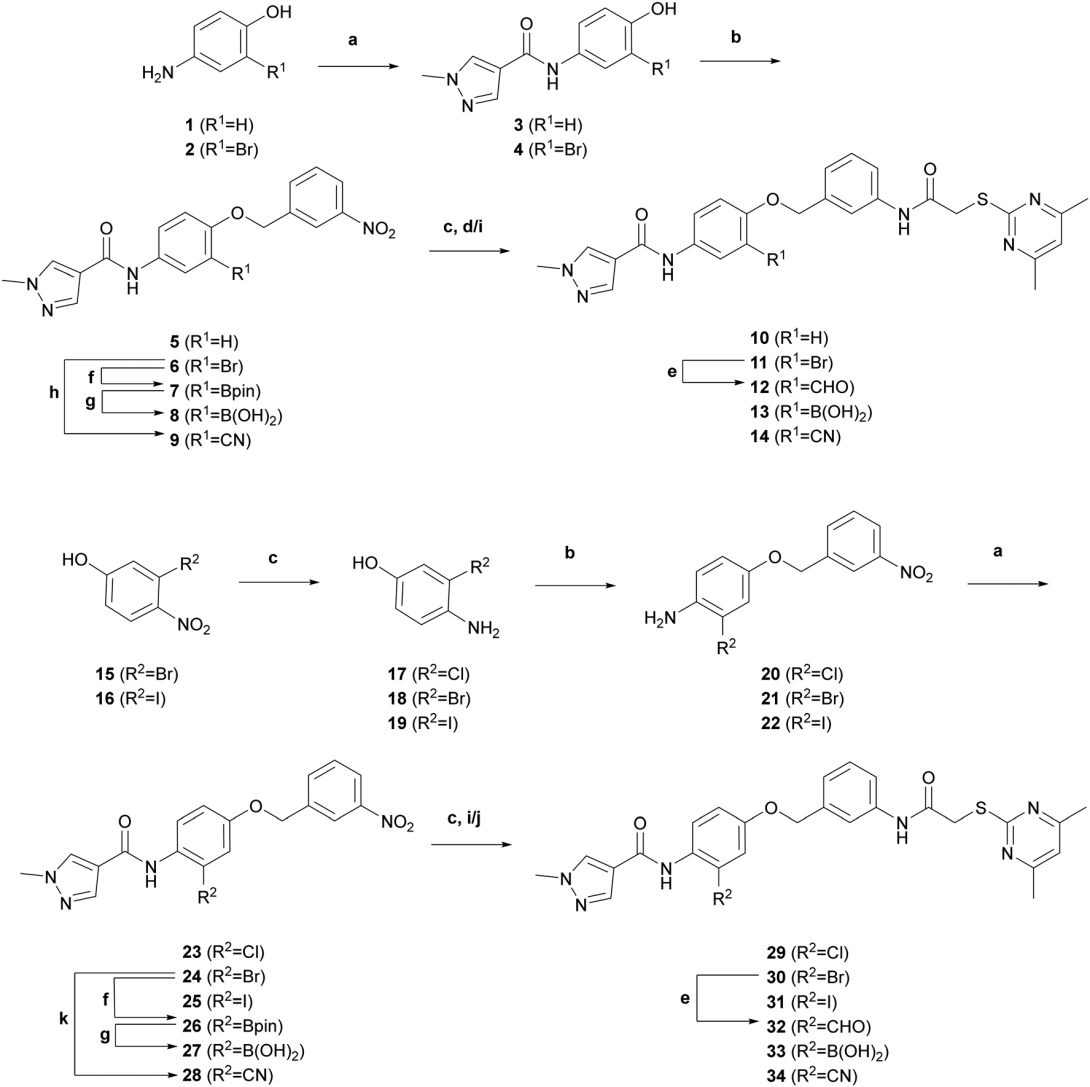
Reagents and conditions: (a) 1-methyl-1*H*-pyrazole-4-carboxylic acid, HATU, DIPEA, THF, rt–65 °C, 3 h–6 d, 21–71%; (b) 3-nitrobenzyl bromide, K_2_CO_3_, DMF, 0 °C–rt, 5 h–16 h, 38% quant.; (c) Fe, NH_4_Cl, EtOH, 90 °C, 2 h, 86–89%; (d) 2-((4,6-dimethylpyrimidin-2-yl)thio)acetic acid, EDC, HOBt, DIPEA, DCM, rt, 18 h, 62% (over 2 steps); (e) Pd(OAc)_2_, dppb, *N*-formylsaccharin, Na_2_CO_3_, Et_3_SiH, DMF, 75 °C, 19 h, 3%; (f) B_2_pin_2_, PdCl_2_(dppf)·DCM, KOAc, 1,4-dioxane, 95 °C, 19 h, 26–45%; (g) NaIO_4_, NH_4_OAc or HCl, acetone/H_2_O or THF/H_2_O, rt, 2 h–15 h; 45% quant. (h) Zn(CN)_2_, Pd(PPh_3_)_4_, DMF, 150 °C, 22 h, 7%; (i) 2-((4,6-dimethylpyrimidin-2-yl)thio)acetic acid, EDC·HCl, 4-DMAP, DMF, rt, 18 h–7 d, 40–69% (over 2 steps); (j) bromoacetyl bromide, DMF, rt, 30 min; then 4,6-dimethylpyrimidine-2-thiol, *t*-BuOK, DMF, rt, 19 h, 26% (over 3 steps); (k) CuCN, DMF, 150 °C, 22 h, 46%.

The lead structure 24a (here 10) was synthesised according to literature with slight modifications in the chronology.^23,24^ Syntheses of functionalised 24a derivatives 11–14 were initiated by the amide coupling of 4-amino-2-bromophenol (2) and 1-methyl-1*H*-pyrazole-4-carboxylic acid to give amide 4 (Scheme 1). Williamson ether synthesis of 4 with 3-nitrobenzyl bromide gave bromobenzene intermediate 6, which afforded nitrile 9 with zinc(ii) cyanide following published procedures,^42^ and boronic acid 8 following Miyaura borylation protocols^43^ and subsequent oxidative cleavage of boronic acid pinacol ester 7. In the final step, reduction of the nitrobenzene derivatives 6, 8 and 9 followed by amide coupling with 2-((4,6-dimethylpyrimidin-2-yl)thio)acetic acid gave bromobenzene derivative 11, boronic acid 13 and nitrile 14, respectively. In contrast, the introduction of the aldehyde functional group was performed in the final step due to its instability. Here, bromobenzene derivative 11 was formylated to aldehyde 12 with *in situ* generated carbon monoxide from *N*-formylsaccharin following protocols from Ueda *et al.*^44^ A liquid–liquid extraction protocol^45^ utilising sodium bisulfite to form a charged bisulfite adduct with subsequent regeneration of the aldehyde *via* basification was necessary, since purification with flash column chromatography alone did not afford the desired aldehyde 12 with sufficient purity. However, the obtained 3% yield was rather unsatisfactory, owing to stability issues. Nevertheless, the amount of product 12 obtained sufficed for chemical analysis and biological testing. Analogously, the preparation of the isomeric functionalised 24a derivatives 29–34 followed a similar synthetic route. Building blocks 4-amino-3-bromophenol (18) and 4-amino-3-iodophenol (19) were obtained from the reduction of 3-bromo-4-nitrophenol (15) and 3-iodo-4-nitrophenol (16), respectively. These phenols and the commercially available 4-amino-3-chlorophenol (17) were then subjected to Williamson ether synthesis prior to amide coupling with 1-methyl-1*H*-pyrazole-4-carboxylic acid as we noticed a significantly higher reactivity of the phenols compared to the aromatic amines. Similar to the previous synthetic route, bromobenzene intermediate 24 served as a key intermediate for the preparation of boronic acid 27 and nitrile 28. Reduction of the nitrobenzene derivatives 23–25 and 28 and subsequent amide coupling with 2-((4,6-dimethylpyrimidin-2-yl)thio)acetic acid in the final step afforded halogenated derivatives 29–31 and nitrile 34, respectively. It is noteworthy that the amide coupling with 2-((4,6-dimethylpyrimidin-2-yl)thio)acetic acid after the reduction of the boronic acid-bearing nitrobenzene 27 failed with various amide coupling reagents. To overcome this problem, the highly reactive bromoacetyl bromide was instead implemented, thereby generating a α-bromoacetamide intermediate that underwent nucleophilic substitution with 4,6-dimethylpyrimidine-2-thiol to give boronic acid 33. Aldehyde 32 was obtained from bromobenzene derivative 30 with the same formylation^44^ and bisulfite-mediated purification^45^ protocol that was developed for the synthesis of aldehyde 12.

**Scheme 2:**
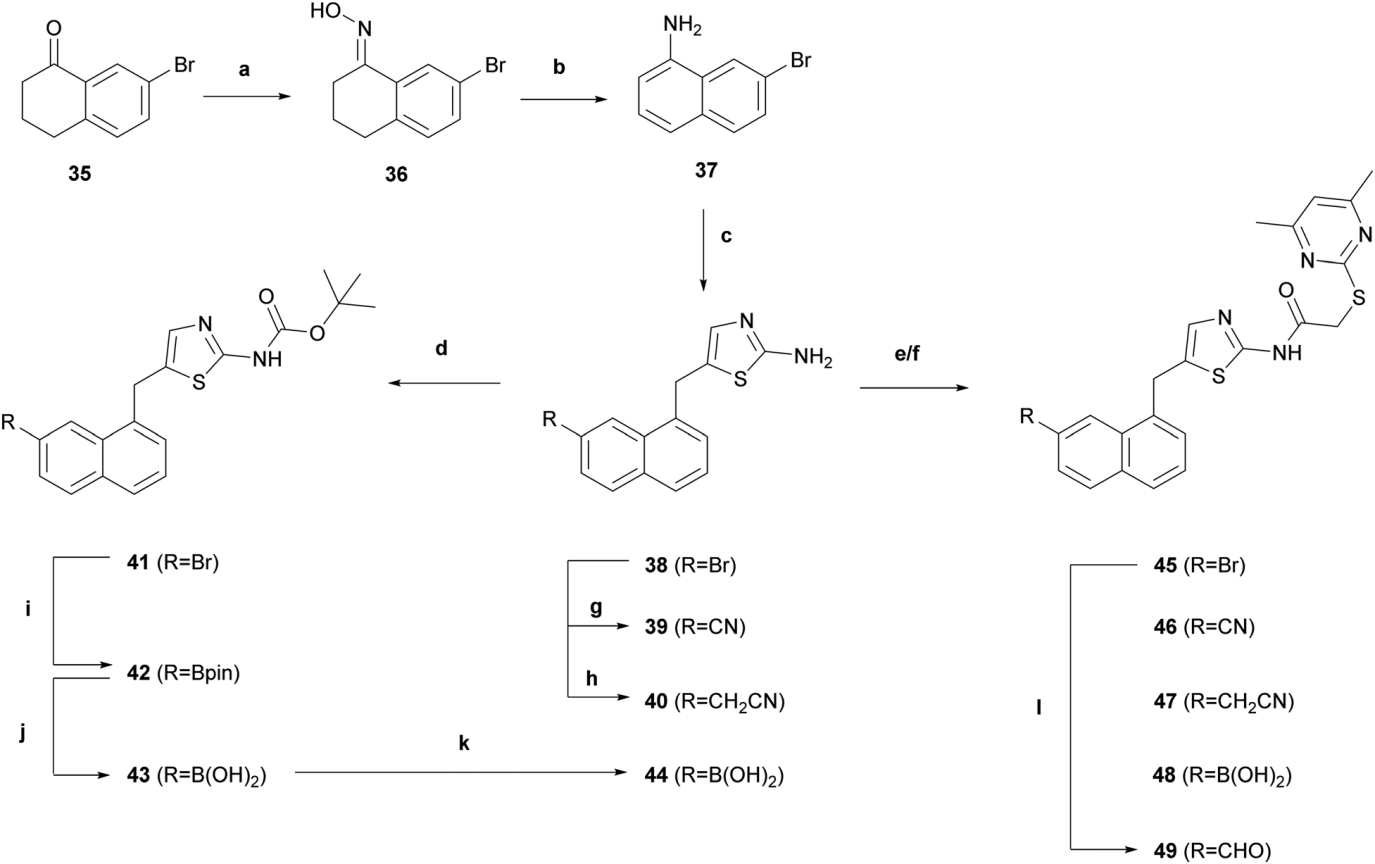
Reagents and conditions: (a) NH_2_OH·HCl, pyridine, EtOH/H_2_O, rt, 24 h, quant.; (b) Ac_2_O, H_2_SO_4_, AcOH, 120 °C, 24 h, 25%. (c) NaNO_2_, HCl; then FeCl_3_·6H_2_O, HCl, H_2_O, 0 °C, 10 min; then CuCl_2_·2H_2_O, HCl, acetone/EtOH, 0 °C, 10 min; then acrolein, acetone/H_2_O, rt, 4 h; then thiourea, EtOH, 80 °C, 30 h, 41% (over 4 steps); (d) Boc_2_O, toluene, 100 °C, 4.5 h, 85%; (e) 2-((4,6-dimethylpyrimidin-2-yl)thio)acetic acid, HATU, DIPEA, DMF, rt, 18 h, 29–40%; (f) 2-((4,6-dimethylpyrimidin-2-yl)thio)acetic acid, EDC·HCl, 4-DMAP, DMF, rt, 16 h, 17–52%; (g) Zn(CN)_2_, Pd(PPh_3_)_4_, DMF, 80 °C, 18 h, 61%; (h) 4-(4,4,5,5-tetramethyl-1,3,2-dioxaborolan-2-yl)isoxazole, PdCl_2_(dppf)·DCM, DMF, 90 °C, 20 h, 26% (i) B_2_pin_2_, PdCl_2_(dppf)·DCM, KOAc, 1,4-dioxane, 80 °C, 1 h; then rt, 2 h, 51%; (j) NaIO_4_, HCl, THF/H_2_O, rt, 4 h, 84%; (k) TFA, CHCl_3_, rt, 17 h, quant.; (l) Pd(OAc)_2_, dppf, *N*-formylsaccharin, Na_2_CO_3_, Et_3_SiH, DMF, 75 °C, 19 h, 11%.

The synthesis of functionalised SirReal2 derivatives was initiated by the preparation of aminothiazole 38 mainly according to literature (Scheme 2).^20^ In deviation therefrom, intermediate 7-bromonaphtalene-1-amine (37) was prepared in a two-step process according to a patented method.^46^ Starting from 7-bromo-1-tetralone (35), oxime 36 was prepared and then converted to 7-bromonaphtalene-1-amine (37) *via* Semmler–Wolff aromatisation. Subsequently, a modified Meerwein arylation afforded aminothiazole 38 that served as a key intermediate for further functional group modifications, such as the palladium-catalysed synthesis^42,47^ of nitrile 39 and cyanomethyl derivative 40. Notably, the introduction of the boronic acid necessitated the protection of the amine. *N*-Boc protection of aminothiazole 38 gave bromobenzene intermediate 41, which underwent Miyaura borylation^43^ to give boronic acid pinacol ester 42. Oxidative cleavage of 42 and subsequent *N*-Boc cleavage with TFA gave boronic acid 44. The functionalised SirReal2 derivatives 45–48 were then obtained in the final step from 38–40 and 44*via* amide coupling with 2-((4,6-dimethylpyrimidin-2-yl)thio)acetic acid. Formylation of 45 was again performed in the final step of the synthesis, in this case using a protocol from Konishi *et al.*^48^ that utilises a different phosphine ligand, which generated a better yield for the preparation of aldehyde 49. Laborious purification of aldehyde 49 with the aforementioned liquid–liquid extraction protocol^45^*via* a bisulfite adduct was not necessary in this case.

### *In vitro* sirtuin inhibitory activities

The determination of SIRT2 inhibitory activity was performed by Reaction Biology Corporation (Malvern, USA) with a fluorescence-based assay utilizing the fluorogenic peptide of p53 residues 379–382 (RHKK(Ac)-Fl). In general, the determined IC_50_ values of literature-known lead structures 24a (here 10)^23^ and SirReal2 (ref. 20) in this assay are in accordance with their published values. Furthermore, all 24a derivatives showed potent and sub-type selective inhibition of SIRT2 in the nanomolar range (Table 1). However, the introduction of the boronic acid, nitrile and aldehyde moieties in both positions R^1^ and R^2^ (12–14, 32–34) showed no improvement, and to some extent a decrease in the potency compared to the lead structure 24a (10). Similarly, halogen modifications with bromine at the R^1^ position (11) showed no significant improvement in potency. On the contrary, we observed a significant increase in potency by the introduction of halogens at the R^2^ position (compounds 29–31), highlighting the importance of the position of the functional group modifications. In particular, chlorobenzene derivative 29 displayed a 3-fold increase in potency, exhibiting an IC_50_ value of 26 nM, but also the iodine (compound 31, IC_50_ = 29 nM) and bromine derivatives (compound 30, IC_50_ = 54 nM) were highly potent. These results lead to the following ranking in descending order of potency: Cl > I > Br. Although in theory the chlorine atom should form the weakest halogen bond with the ribose hydroxy groups of NAD^+^,^37^ the observation that the chlorobenzene derivative 29 outperformed both bromobenzene and iodobenzene derivatives 30 and 31 suggests a much more complex mechanism than mere halogen bonding for the underlying increase in potency. Additional factors such as the atomic radius and thus steric requirements of these halogens may also play a significant role in the increase in potency, where the smaller chlorine atom may experience less steric effects upon binding at the active site compared to the larger bromine and iodine atoms. Halogen modification of SirReal2 with bromine (compound 45) pointed similarly to an increase in potency. This is consistent with the published IC_50_ values of SirReal2 and 7-bromo-SirReal2 (45).^20^ Although the introduction of boronic acid and aldehyde groups (compounds 48, 49) did not display any significant improvement in potency, the SirReal2 derivative 46 bearing a nitrile group showed a 2-fold increase in SIRT2 inhibition with an IC_50_ value of 122 nM. In comparison, the homologous cyanomethyl derivative 47 only had similar potency to SirReal2 (Table 1).


*In vitro* inhibition of human SIRT1, 2, 3 and 5 by functionalised derivatives of 24a and SirReal2. IC_50_ values against SIRT2 are given as mean with standard deviations (*n* = 3). Inhibition percentages at 50 μM for SIRT1, 3 and 5 are given as mean without standard deviations (*n* = 2)

**Table:** 

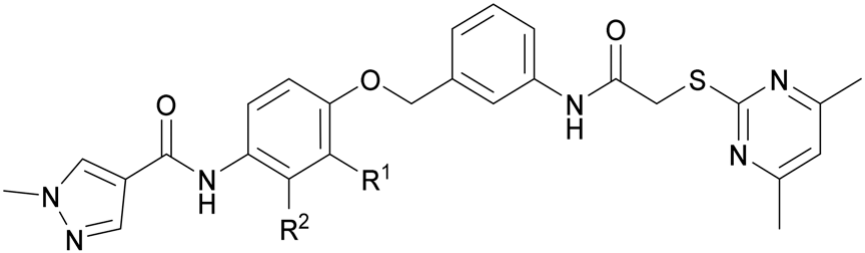						
Compound ID	R^1^	R^2^	IC_50_ (nM)	Inhibition of SIRT1/3/5 in %@50 μM		
			SIRT2	SIRT1	SIRT3	SIRT5
10 (24a^a^)	H	H	79 ± 4	n.d.	n.d.	n.d.
11	Br	H	81 ± 10	n.i.	24	13
12	CHO	H	147 ± 13	2	24	16
13	B(OH)_2_	H	294 ± 17	29	5	10
14	CN	H	125 ± 18	6	35	2
29	H	Cl	26 ± 2	12	26	7
30	H	Br	54 ± 7	4	43	1
31	H	I	29 ± 2	5	22	12
32	H	CHO	184 ± 9	n.i.	7	6
33	H	B(OH)_2_	202 ± 6	71	24	14
34	H	CN	91 ± 7	23	31	4

**Table:** 

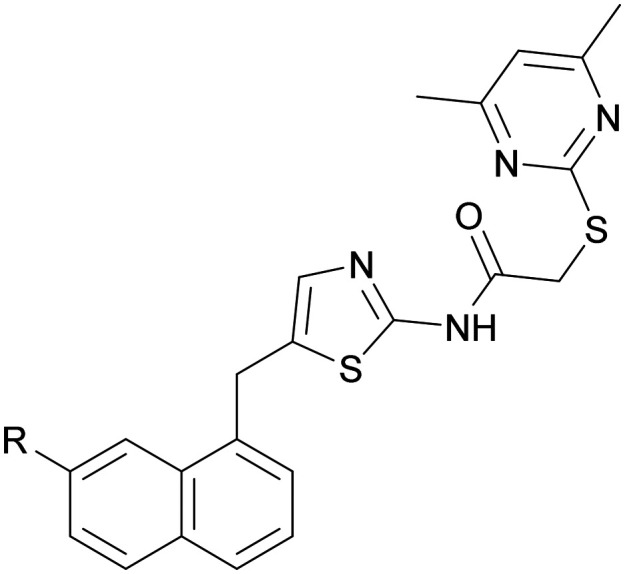					
Compound ID	R	IC_50_ (nM)	Inhibition of SIRT1/3/5 in %@50 μM		
		SIRT2	SIRT1	SIRT3	SIRT5
SirReal2^b^	H	235 ± 10	n.d.	n.d.	n.d.
45^c^	Br	196 ± 17	n.d.	n.d.	n.d.
46	CN	122 ± 8	6	9	8
47	CH_2_CN	235 ± 19	n.i.	15	2
48	B(OH)_2_	235 ± 36	19	12	23
49	CHO	356 ± 42	11	13	21

a Literature-known compound 24a by Yang *et al.* with a published IC_50_ of 0.82 μM for SIRT2.^23^

b Literature-known compound SirReal2 by Rumpf *et al.* with a published IC_50_ of 0.44 μM for SIRT2.^19^

c Literature-known compound 45 by Schiedel *et al.* with a published IC_50_ of 0.21 μM for SIRT2 (ref. 20).

The determination of subtype selectivity against SIRT1, 3 and 5 was performed by measuring the residual enzyme activity after treatment with the corresponding inhibitor at a fixed concentration of 50 μM and subsequent calculation of the percentage inhibition. All target compounds tested showed selectivity towards SIRT2, as corresponding inhibition values of SIRT1, 3 and 5 indicate IC_50_ values of over 50 μM with an exception for compound 33 that showed 71% inhibition at 50 μM. Nevertheless, considering the low IC_50_ value of 202 nM for the desired target SIRT2, selectivity is considered as sufficient.

### Thermal stability of SIRT2–inhibitor complexes

**Fig. 4:**
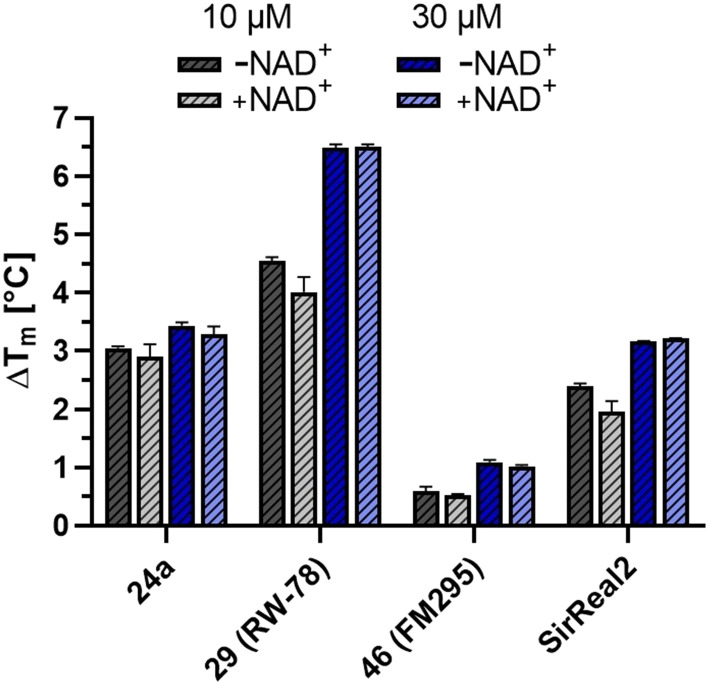
Melting point shifts of SIRT2 with inhibitors at 10 μM and 30 μM in the presence and absence of 2.5 mM co-factor NAD^+^ with no dependency on the co-factor observed. All melting temperature shifts measured were referenced to the melting temperature of SIRT2 without inhibitor, also in the presence and absence of the co-factor. Under both these conditions, the reference melting temperatures showed no significant change, each yielding a melting temperature of 53 °C.

To investigate the potential role of the co-factor NAD^+^ in the potency enhancement of the chloro-derivate 29 (RW-78) and nitrile 46 (FM295) compared to their corresponding lead structures 24a and SirReal2, we performed fluorescence thermal shift assays (Fig. 4). The thermal stability of SIRT2 was determined in the presence of 10 μM and 30 μM inhibitor and in the presence and absence of 2.5 mM co-factor NAD^+^. The presence of 30 μM of compound 29 resulted in a significant increase in the melting temperature of SIRT2 (Δ*T* = 6.5 °C) compared to its lead structure 24a (Δ*T* = 3 °C). However, results showed that the inhibitor-induced SIRT2 stabilization was independent from NAD^+^, contradicting our initial proposed binding mode. Similar results were obtained with the nitrile 46.

### Co-crystal structure of SIRT2 with compounds 29 (RW-78) and 31 (RW-80)

**Fig. 5:**
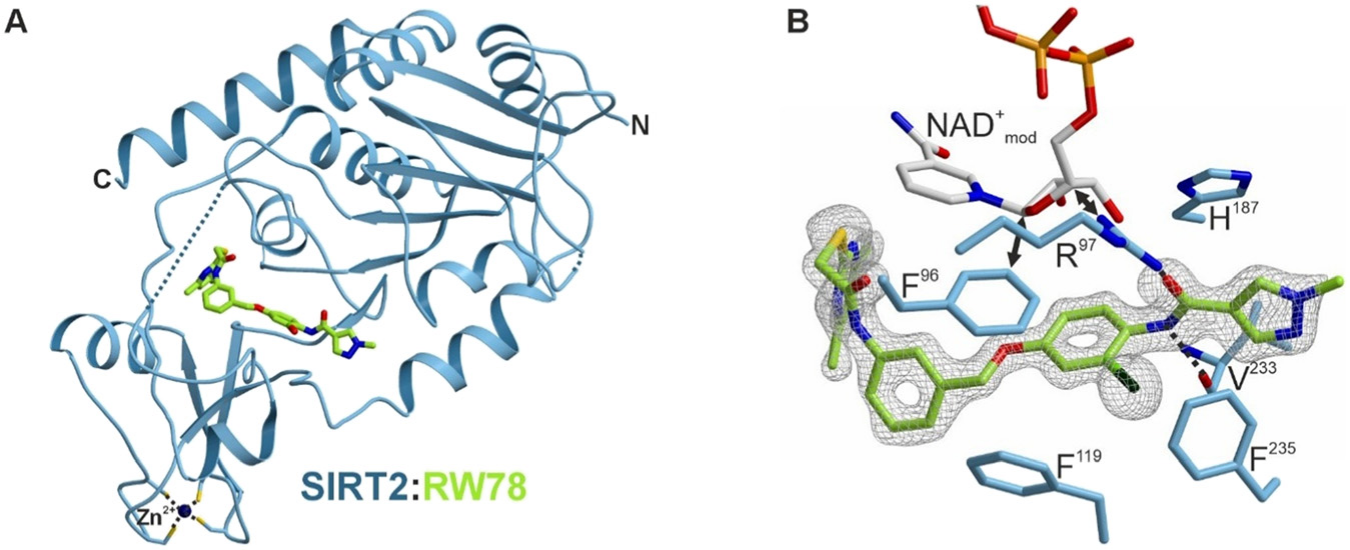
(A) Co-crystal structure of human SIRT2 (blue) with its inhibitor 29 (RW-78, green) (PDB ID: 9S46). Residues 104–106 and 303 are disordered and indicated by blue dotted lines. The zinc ion bound to SIRT2 is shown as a dark blue sphere and coordinating cysteines are depicted as sticks. (B) Close-up view of 29 (green) bound to SIRT2. The experimental *F*_O_–*F*_C_ omit electron density is shown as a gray mesh contoured to 3*σ*. Coordinating amino acid side chains (blue) are shown as sticks and labelled by the one-letter code. 29 is stabilized by hydrogen bonds to R^97^ and the V^233^ carbonyl oxygen (black dotted lines). The chlorine atom of 29 interacts with F^235^*via* halogen–π-bonding. Binding of 29 induces movements of F^96^ and R^97^ into the NAD^+^-binding site (see also Fig. S2C). Modelling of the NAD^+^ co-factor (extracted from the SIRT2:SirReal2 coordinates (PDB ID: 4RMG))^19^ into the SIRT2:RW-78 structure by superposition illustrates that F^96^ and R^97^ clash with NAD^+^ (black double arrows) and hence displace the co-factor.

To clarify the binding mode of the most potent inhibitors we aimed for co-crystal structures with human SIRT2. To this end, we expressed human SIRT2 56-356 as a N-terminal His_6_-SUMO fusion in *Escherichia coli* and purified the protein *via* affinity and size exclusion chromatography with tag removal in between (Fig. S1). Crystallization trials finally yielded an apo structure of SIRT2 (2.15 Å resolution, Table S1, PDB ID: 9S44) and two complex structures with 29 (RW-78) (1.45 Å, Table S2, Fig. 5, PDB ID: 9S46) and 31 (RW-80, Fig. S2A) (1.45 Å, Table S3, PDB ID: 9S48) in the same space group.

Both 29 and 31 occupy a pocket located slightly below the NAD^+^-binding cleft, previously exploited by other potent and selective SIRT2 inhibitors like SirReal2 and 24a (Fig. 5A and S2A and B).^19,23^ Compared to our SIRT2 apo structure, the ligand bound structures adopt the “open-locked-state” reported first in the SIRT2:SirReal2 co-crystal structure (PDB ID: 4RMG; root mean square deviation ≤0.223 Å over 218 C^α^ atoms).^19,49^ Both 29 and 31 are coordinated by the same protein residues and only differ by their halogen atom (Fig. 5B and S2B). In the binding pocket, the inhibitor is stabilized by multiple interactions previously described for the lead structure 24a (PDB: 5YQO, Fig. S2B).^23^ Most importantly, the amide next to the methyl pyrazole hydrogen bonds to the carbonyl oxygen of V^233^ and R^97^. Additionally, the chlorine (29) or iodine (31) atom engages in halogen–π-interactions with F^235^ (Fig. 5B and S2A and B). The spacing between the chlorine atom of 29 and the centroid of the π-system of F^235^ (3.6 Å) matches the average distance of C–Cl⋯π-bonds (3.854 Å),^50^ explaining the high potency of 29. Contrary to our design, 29 and 31 do not engage in interactions with the SIRT2 co-factor NAD^+^ but stabilize the enzyme in an inactive state. This state is established by rearrangements of residues near the active site (Fig. 5B and S2C). For instance, the catalytic histidine H^187^ is slightly shifted compared to SIRT2 apo structures (PDB ID: 1J8F,^49^ PDB ID: 9S44). In addition, by interacting with the inhibitor, F^96^ and R^97^ move into the NAD^+^-binding pocket, displacing the co-factor and prohibiting NAD^+^-binding. Therefore, the determined structures do not show electron densities for the co-factor, although 5 mM NAD^+^ was included in the crystallization screens. Instead of the co-factor, the NAD^+^-binding site contains many water molecules. Considering the high affinity of 29 and 31 (Table 1), the entropic barrier that is associated with the displacement of NAD^+^ and the binding of water molecules must be counteracted by an enormous enthalpic stabilization of the inhibitors in the SIRT2 pocket. Notably, the NAD^+^ displacement is supported by thermal shift assays (Fig. 4), which revealed that 29 increases the melting temperature of SIRT2 independently of NAD^+^.

### Cellular target engagement in HEK293T cells *via* NanoBRET assay

**Fig. 6:**
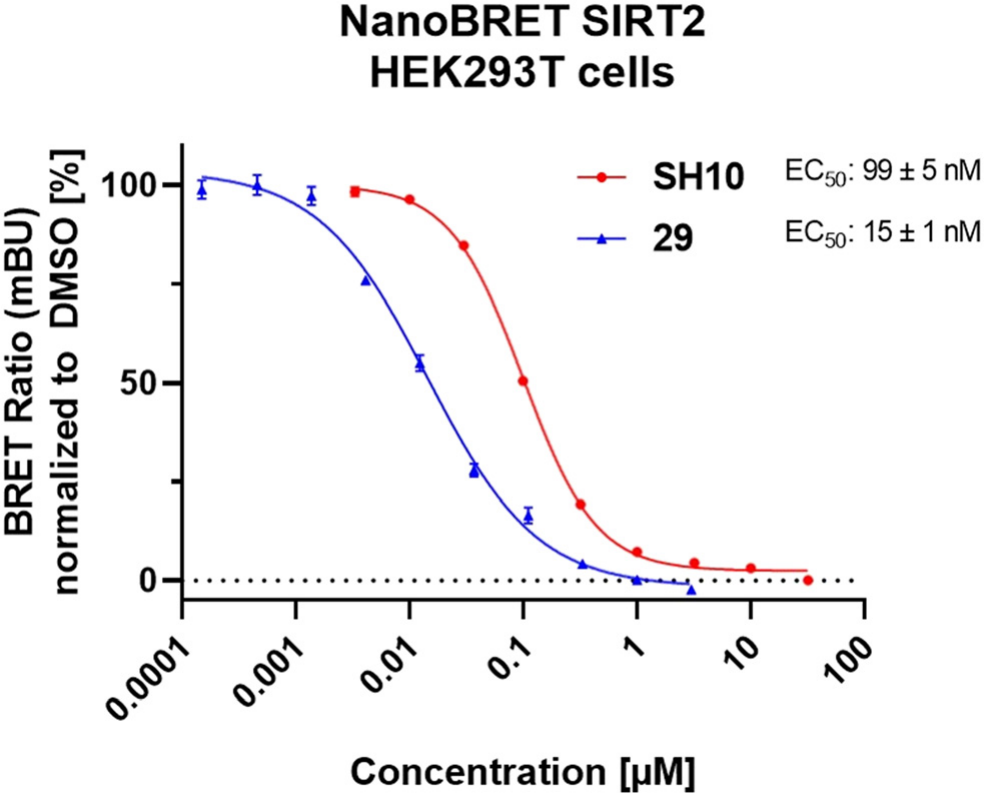
NanoBRET-based cellular target engagement of the SIRT2 inhibitor 29. Cellular binding of 29 was evaluated using a NanoBRET target engagement assay in live HEK293T cells. Cells were incubated with 2 μM NanoBRET tracer and treated with increasing concentrations 29 for 2 hours at 37 °C. Binding for SH10 is plotted as a reference. For compound 29, a stable HEK293T cell line expressing NanoLuc-SIRT2 fusion construct was used, whereas for SH10 HEK293T cells were transiently transfected with the NanoLuc-SIRT2 fusion construct.

Cellular SIRT2 target engagement of chloro-derivative 29 was assessed in HEK293T cells *via* NanoBRET assay that was developed by Vogelmann *et al.*^21^ The NanoBRET tracer pre-treated cells were incubated with increasing concentrations of chloro-derivative 29, reflecting its intracellular binding to SIRT2. Compound 29 showed high target engagement with an EC_50_ value of 15 nM (Fig. 6), validating our data obtained from *in vitro* inhibition studies. For comparison, the highly potent triazole-based SirReal inhibitor Vogelmann_12 (SH10) was used as a reference and showed an EC_50_ value of 99 nM.

## Conclusions

Based on the rationale to target the essential co-factor NAD^+^ of SIRT2 *via* reversible covalent binding or halogen bonding with appropriate functional groups, a total of 14 potent and subtype selective SIRT2 inhibitors were synthesised. While lead structure modifications of SirReal2 and 24a with polar, potential reversible covalent binding warheads showed ambiguous results, nitrile 46 (FM295; IC_50_ = 122 nM) demonstrated significant potency enhancement compared to its lead structure SirReal2 (IC_50_ = 235 nM). Among the halogenated derivatives of 24a (10), compounds 30 (IC_50_ = 54 nM) and 31 (IC_50_ = 29 nM) outperformed the lead structure 24a (IC_50_ = 79 nM). In addition, ligand 29 (RW-78) emerged as the best member of the whole series and as one of the most potent SIRT2 inhibitors known to date (IC_50_ = 26 nM) while maintaining high subtype selectivity. X-ray crystallographic data visualized that compound 29 (RW-78) undergoes halogen–π interactions with SIRT2 and induces structural changes that interfere with co-factor binding. These results are in agreement with thermal shift assays indicating that the inhibition of compound 29 (RW-78) is NAD^+^ independent. In addition, the utility of compound 29 (RW-78) in the cellular context was validated *via* NanoBRET assay in HEK293T cells that showed high target engagement to SIRT2 (EC_50_ = 15 nM). In conclusion, our findings provide a valuable contribution to a deeper understanding of the structure–activity relationships of SIRT2 inhibitors and a foundation for further optimisation of SIRT2-selective inhibitors.

## Experimental

### Chemistry

#### Materials and instruments

All solvents and reagents were purchased from commercial sources and used without further purification. Standard vacuum line techniques were applied. Reactions were monitored *via* thin layer silica gel chromatography (TLC) using polyester sheets POLYGRAM SIL G/UV254 coated with 0.2 mm silica gel (Macherey-Nagel). Plates were visualised using UV light (254 nm or 365 nm) or staining with KMnO_4_, CAM (ceric ammonium molybdate) or DNPH (dinitrophenylhydrazine). Products were purified by flash column chromatography (normal-phase silica gel chromatography) using SiO_2_ 60 (0.040–0.063 mm, 230–400 mesh ASTM) from Merck. NMR spectra were recorded with Avance III HD 400 MHz Bruker BioSpin and Avance III HD 500 MHz Bruker BioSpin (^1^H-NMR: 400 MHz and 500 MHz, ^13^C-NMR: 101 MHz and 126 MHz) using the deuterated solvent stated. Chemical shifts (*δ*) are quoted in parts per million (ppm) and referenced to the residual solvent peak. Multiplicities are denoted as s-singlet, d-doublet, t-triplet, q-quartet and quin-quintet. Coupling constants *J* are given in Hz and round to the nearest 0.1 Hz. Infrared spectra were recorded from 4000 to 650 cm^−1^ on a PERKIN ELMER Spectrum BX-59343 FT-IR instrument. A Smiths Detection DuraSamp IR II Diamond ATR sensor was used for detection. The absorption bands are reported in wavenumbers [cm^−1^]. High resolution mass spectra (HR-MS) were recorded using a Jeol Mstation 700 or JMS GCmate II Jeol instrument for electron impact ionisation (EI). Thermo Finnigan LTQ was used for electrospray ionisation (ESI). Melting points were measured with a Büchi Schmelzpunktapparatur B-540. HPLC analytical measurements at 210 nm and 254 nm for purities determination was performed with the following methods:

**Method 1**:

Zorbax SB C18 3.5 μm (4.6 × 100 mm), injection volume 5 μL.

a) MeCN/water (35 : 65); flow rate 1.0 mL min^−1^; temp. 35 °C.

b) MeCN/water (50 : 50); flow rate 1.2 mL min^−1^; temp. 35 °C.

c) MeCN/water (50 : 50); flow rate 1.2 mL min^−1^; temp. 50 °C.

**Method 2**:

Raptor C18 5 μm (4.6 × 150 mm), injection volume 5 μL.

a) MeCN/water (35 : 65); flow rate 0.7 mL min^−1^; temp. 35 °C.

**Method 3**:

Zorbax Eclipse Plus® C18 5 μm (4.6 × 150 mm), injection volume 5 μL.

a) MeCN/water (70 : 30); flow rate 1.0–1.5 mL min^−1^; temp. 30–50 °C.

b) MeCN/water (50 : 50); flow rate 1.0–1.5 mL min^−1^; temp. 30–50 °C.

c) MeCN/phosphate buffer pH = 5 (70 : 30); flow rate 1.0–1.2 mL min^−1^; temp. 30–50 °C.

### Synthetic procedures

#### General procedure A – amide coupling (I) with HATU and DIPEA

To a stirred solution of the appropriate carboxylic acid (1.0 equivalent) in THF with a concentration of 0.20 M were added DIPEA (3.0 equivalents) and HATU (1.5 equivalents). The reaction mixture was stirred at room temperature for 1 h. The appropriate amine (1.0 equivalent) was then added and unless stated otherwise, the reaction mixture was further stirred at room temperature for 3 h. DCM and water were then added, the resulting two phases were separated, and the aqueous phase was extracted with DCM (3×). The combined organic phases were dried using a phase separation paper and the solvent was removed *in vacuo*. Unless stated otherwise, the crude product was then purified by FCC using the indicated eluent.

#### General procedure B – amide coupling (II) with EDC, HOBt and DIPEA

To a stirred solution of the appropriate amine (1.0 equivalent) in DCM with a concentration of 0.10 M were added HOBt (1.5 equivalents), EDC (2.0 equivalents), DIPEA (2.0 equivalents) and the appropriate carboxylic acid (2.0 equivalents). The reaction mixture was stirred at room temperature for 18 h. DCM and water were then added, the resulting two phases were separated, and the aqueous phase was extracted with DCM (3×). The combined organic phases were dried using a phase separation paper and the solvent was removed *in vacuo*. The crude product was then purified by FCC using the indicated eluent.

#### General procedure C – amide coupling (III) with EDC·HCl and 4-DMAP

To a stirred solution of the appropriate carboxylic acid (2.0 equivalents) in DMF with a concentration of 0.40 M were added EDC·HCl (2.0 equivalents) and 4-DMAP (2.0 equivalents). The reaction mixture was stirred at room temperature for 15 minutes. Afterwards, the appropriate amine (1.0 equivalent) was added and the solution was stirred at the stated temperature for the stated time. The solution was then diluted with EtOAc and the organic phase was washed with brine (3×). The organic phase was dried using a phase separation paper and the solvent was removed *in vacuo*. The crude product was then purified by FCC using the indicated eluent.

#### General procedure D – Williamson ether synthesis

To a stirred solution of the appropriate phenol (1.0 equivalent) in DMF with a concentration of 0.10 M was added K_2_CO_3_ (3.0 equivalents). The reaction mixture was stirred at room temperature for 30 minutes. Afterwards, the appropriate alkyl halide (1.2 equivalents) was added and the mixture was stirred at room temperature for 16 h. The solution was then diluted with EtOAc and the organic phase was washed with brine (3×). The organic phase was dried using a phase separation paper and the solvent was removed *in vacuo*. The crude product was then purified by the stated procedure.

#### General procedure E – nitrobenzene reduction

To a stirred solution of the appropriate nitrobenzene derivative (1.0 equivalent) in EtOH with a concentration of 0.010 M were added iron powder (5.0 equivalents) and 0.30 M aq. NH_4_Cl (5.0 equivalents) at 50 °C. The reaction mixture was then refluxed at 90 °C for 2 h. Afterwards, the iron powder was filtered off from the still hot mixture, and the filtrate was concentrated *in vacuo*. Unless stated otherwise, the crude product was used for the next step without further purification.

#### *N*-(4-Hydroxyphenyl)-1-methyl-1*H*-pyrazole-4-carboxamide (3)

Prepared according to General procedure A from 1-methyl-1*H*-pyrazole-4-carboxylic acid (500 mg, 3.96 mmol) and aminophenol 1 (433 mg, 3.96 mmol). The crude product was purified by FCC (DCM/MeOH 96 : 4) to give 3 (179 mg, 3.96 mmol, 21%) as a white solid; m.p. 225 °C; IR (ATR) *ν̃*/cm^−1^ 3347, 3020, 1641, 1602, 1558, 1530, 1510, 1430, 1351, 1202, 1099, 1006, 860, 813, 749; *δ*_H_ (400 MHz; (CD_3_)_2_SO) 9.57 (s, 1H, CONH), 9.18 (s, 1H, OH), 8.24 (s, 1H, 5-H), 7.96 (s, 1H, 3-H), 7.47–7.41 (m, 2H, 2′-H and 6′-H), 6.74–6.68 (m, 2H, 3′-H and 5′-H), 3.87 (s, 3H, CH_3_). *δ*_C_ (101 MHz; (CD_3_)_2_SO) 160.00 (CONH), 153.40 (C-4′), 138.62 (C-3), 132.34 (C-5), 130.63 (C-1′), 121.92 (C-2′ and C-6′), 118.72 (C-4), 114.96 (C-3′ and C-5′), 38.79 (CH_3_); HRMS (ESI): calcd.: 218.0924; found: {*m*/*z*: [M + H]^+^ 218.0928}.

#### *N*-(3-Bromo-4-hydroxyphenyl)-1-methyl-1*H*-pyrazole-4-carboxamide (4)

Prepared according to General procedure A from 1-methyl-1*H*-pyrazole-4-carboxylic acid (500 mg, 3.96 mmol) and aminophenol 2 (761 mg, 3.96 mmol). The crude product was purified by FCC (DCM/MeOH 98 : 2) to give amide 4 (649 mg, 2.19 mmol, 55%) as a light pink solid; m.p. 218 °C; IR (ATR) *ν̃*/cm^−1^ 3108, 1637, 1596, 1558, 1410, 1268, 1227, 1197, 1008, 852, 801, 753, 657; *δ*_H_ (400 MHz; (CD_3_)_2_SO) 9.98 (s, 1H, OH), 9.69 (s, 1H, CONH), 8.25 (s, 1H, 5-H), 7.96 (s, 1H, 3-H), 7.89 (d, *J* = 2.5 Hz, 1H, 2′-H), 7.45 (dd, *J* = 8.8, 2.5 Hz, 1H, 6′-H), 6.90 (d, 1H, 5′-H), 3.88 (s, 3H, CH_3_). *δ*_C_ (101 MHz; (CD_3_)_2_SO) 160.14 (CONH), 150.02 (C-4′), 138.65 (C-3), 132.50 (C-5), 131.83 (C-1′), 124.45 (C-2′), 120.73 (C-6′), 118.37 (C-4), 116.01 (C-5′), 108.52 (C-3′), 38.83 (CH_3_); HRMS (ESI): calcd.: 296.0029; found: {*m*/*z*: [M + H]^+^ 296.0033}.

#### 1-Methyl-*N*-(4-((3-nitrobenzyl)oxy)phenyl)-1*H*-pyrazole-4-carboxamide (5)

Prepared according to General procedure D from phenol 3 (165 mg, 0.760 mmol) and 1-(bromomethyl)-3-nitrobenzene (197 mg, 0.911 mmol). The crude product was purified by FCC (DCM/MeOH 95 : 5) to give ether 5 (221 mg, 0.626 mmol, 82%) as a yellow solid. Analytical data are in alignment with literature.^23^

#### *N*-(3-Bromo-4-((3-nitrobenzyl)oxy)phenyl)-1-methyl-1*H*-pyrazole-4-carboxamide (6)

Prepared according to General procedure D from phenol 4 (3.10 g, 10.5 mmol) and 1-(bromomethyl)-3-nitrobenzene (2.71 g, 12.6 mmol). The crude product was resuspended in hexanes, vacuum filtered, and the residue washed sequentially with hexanes (2×), EtOAc (2×) and DCM (1×). The filtrate was discarded and the solid residue collected to give ether 6 (4.52 g, 10.5 mmol, quant.) as a beige solid; m.p. 226 °C; IR (ATR) *ν̃*/cm^−1^ 3394, 3114, 1649, 1593, 1550, 1520, 1499, 1397, 1357, 1275, 1229, 1057, 1001, 881, 853, 790, 728.; *δ*_H_ (400 MHz; (CD_3_)_2_SO) 9.82 (s, 1H, CONH), 8.38–8.36 (m, 1H, 2′′-H), 8.27 (s, 1H, 5-H), 8.22–8.19 (m, 1H, 4′′-H), 8.04 (d, *J* = 2.5 Hz, 1H, 2′-H), 8.00–7.97 (m, 1H, 3-H), 7.96–7.91 (m, 1H, 6′′-H), 7.73 (t, *J* = 7.9 Hz, 1H, 5′′-H), 7.65 (dd, *J* = 8.9, 2.6 Hz, 1H, 6′-H), 7.22 (d, *J* = 9.0 Hz, 1H, 5′-H), 5.34 (s, 2H, CH_2_), 3.89 (s, 3H, CH_3_). *δ*_C_ (101 MHz; (CD_3_)_2_SO) 160.31 (CONH), 149.96 (C-4′), 147.86 (C-3′′), 139.16 (C-1′′), 138.71 (C-3), 133.87 (C-1′), 133.74 (C-6′′), 132.63 (C-5), 130.10 (C-5′′), 124.42 (C-2′), 122.78 (C-4′′), 121.78 (C-2′′), 120.29 (C-6′), 118.20 (C-4), 114.43 (C-5′), 110.77 (C-3′), 69.05 (CH_2_), 38.85 (CH_3_); HRMS (ESI): calcd.: 431.0349; found: {*m*/*z*: [M + H]^+^ 431.0355}.

#### 1-Methyl-*N*-(4-((3-nitrobenzyl)oxy)-3-(4,4,5,5-tetramethyl-1,3,2-dioxaborolan-2-yl)phenyl)-1*H*-pyrazole-4-carboxamide (7)

PdCl_2_(dppf)·DCM (93.3 mg, 0.128 mmol), bis(pinacolato)diboron (648 mg, 2.55 mmol), bromo derivative 6 (550 mg, 1.28 mmol) and KOAc (501 mg, 5.10 mmol) were dissolved in degassed 1,4-dioxane (40 mL) under N_2_ atmosphere and stirred at 95 °C for 19 h. The reaction mixture was cooled to room temperature and then filtered. The filtrate was then diluted with EtOAc (50 mL). Water (50 mL) was added, the resulting two phases were separated, and the aqueous phase was extracted with EtOAc (3 × 60 mL). The combined organic phases were then dried using a phase separation paper and concentrated *in vacuo.* The crude product was purified by FCC (hexanes/EtOAc 20 : 80) to give boronic acid pinacol ester 7 (277 mg, 0.579 mmol, 45%) as a yellow solid; m.p. 254 °C; IR (ATR) *ν̃*/cm^−1^ 3339, 2982, 1640, 1557, 1530, 1495, 1344, 1318, 1239, 1140, 1068, 1008, 812, 731; *δ*_H_ (400 MHz; (CD_3_)_2_SO) 9.75 (s, 1H, CONH), 8.55 (s, 1H, 2′′-H), 8.28 (s, 1H, 5-H), 8.21–8.16 (m, 1H, 4′′-H), 8.03–7.99 (m, 2H, 3-H and 6′′-H), 7.97 (dd, *J* = 9.0, 2.7 Hz, 1H, 6′-H), 7.83 (d, *J* = 2.8 Hz, 1H, 2′-H), 7.71 (t, *J* = 8.0 Hz, 1H, 5′′-H), 7.09 (d, *J* = 9.0 Hz, 1H, 5′-H), 5.25 (s, 2H, CH_2_), 3.89 (s, 3H, NCH_3_), 1.33 (s, 12H, (CH_3_)_4_). *δ*_C_ (101 MHz; (CD_3_)_2_SO) 160.14 (CONH), 158.47 (C-4′), 147.97 (C-3′′), 140.25 (C-1′′), 138.72 (C-3), 133.04 (C-6′′), 132.48 (C-5 and C-1′), 129.59 (C-5′′), 128.41 (C-2′), 124.72 (C-6′), 122.23 (C-4′′), 120.94 (C-2′′), 118.52 (C-4), 117.15 (C-3′), 112.48 (C-5′), 83.30 ((**C**(CH_3_)_2_)_2_), 68.07 (CH_2_), 38.83 (NCH_3_), 24.63 ((CH_3_)_4_); HRMS (ESI): calcd.: 479.2096; found: {*m*/*z*: [M + H]^+^ 479.2114}.

#### (5-(1-Methyl-1*H*-pyrazole-4-carboxamido)-2-((3-nitrobenzyl)oxy)phenyl)boronic acid (8)

To a stirred solution of boronic acid pinacol ester 7 (80.0 mg, 0.167 mmol) in acetone (20 mL) were added sodium periodate (107 mg, 0.502 mmol), ammonium acetate (38.7 mg, 0.502 mmol) and water (7 mL). The reaction mixture was stirred vigorously at room temperature for 2 h. Afterwards, acetone was removed *in vacuo* and the reaction mixture was diluted with DCM (20 mL). Water (25 mL) was added, the resulting two phases were separated, and the aqueous phase was extracted with DCM (3 × 25 mL). The combined organic phases were then dried with Na_2_SO_4_ and then concentrated *in vacuo.* The crude product was then purified by FCC (DCM/MeOH 97 : 3) to give boronic acid 8 (30.0 mg, 75.7 μmol, 45%) as an off-white solid; m.p. 190 °C (decomposition); IR (ATR) *ν̃*/cm^−1^ 3292, 2919, 1638, 1557, 1528, 1492, 1417, 1351, 1316, 1227, 1157, 1008, 861, 808, 759, 733, 673; *δ*_H_ (400 MHz; (CD_3_)_2_SO) 9.68 (s, 1H, CONH), 8.40–8.38 (m, 1H, 2′-H), 8.27 (s, 1H, 5′′-H), 8.21–8.17 (m, 1H, 4′-H), 7.99 (s, 1H, 3′′-H), 7.95 (d, *J* = 7.4 Hz, 1H, 6′-H), 7.86 (s, 2H, B(OH)_2_), 7.81–7.76 (m, 1H, 4-H), 7.74–7.70 (m, 2H, 6-H and 5′-H), 7.01 (d, *J* = 8.9 Hz, 1H, 3-H), 5.30 (s, 2H, CH_2_), 3.88 (s, 3H, CH_3_). *δ*_C_ (101 MHz; (CD_3_)_2_SO) 160.11 (CONH), 157.64 (C-2), 147.88 (C-3′), 139.80 (C-1′), 138.70 (C-3′′), 133.96 (C-6′), 132.44 (C-5 and C-5′′), 129.99 (C-5′), 127.17 (C-6), 123.02 (C-4), 122.65 (C-4′), 122.04 (C-2′), 118.59 (C-4′′), 111.88 (C-3), 68.30 (CH_2_), 38.81 (CH_3_); HRMS (ESI): calcd.: 419.1133; found: {*m*/*z*: [M + Na]^+^ 419.1140}.

#### *N*-(3-Cyano-4-((3-nitrobenzyl)oxy)phenyl)-1-methyl-1*H*-pyrazole-4-carboxamide (9)

Bromo derivative 6 (200 mg, 0.464 mmol), Pd(PPh_3_)_4_ (53.6 mg, 46.4 μmol) and Zn(CN)_2_ (32.7 mg, 0.278 mmol) were dissolved in dry DMF (1 mL) under N_2_ atmosphere and stirred at 150 °C for 22 h. The reaction mixture was cooled to room temperature and then diluted with EtOAc (25 mL). The organic phase was washed with brine (4 × 30 mL) and then dried with Na_2_SO_4_ and concentrated *in vacuo.* The crude product was purified by FCC (DCM/MeOH 98 : 2) to give nitrile 9 (11.6 mg, 30.7 μmol, 7%) as a beige solid; m.p. 251 °C; IR (ATR) *ν̃*/cm^−1^ 3397, 2226, 1668, 1593, 1553, 1528, 1510, 1351, 1319, 1287, 1247, 1207, 1011, 870, 861, 814, 748, 731; *δ*_H_ (400 MHz; (CD_3_)_2_SO) 9.97 (s, 1H, CONH), 8.37 (s, 1H, 2′′-H), 8.29 (s, 1H, 5-H), 8.23 (d, *J* = 8.3 Hz, 1H, 4′′-H), 8.08 (s, 1H, 2′-H), 7.99 (s, 1H, 3-H), 7.95–7.88 (m, 2H, 6′-H and 6′′-H), 7.75 (t, *J* = 7.9 Hz, 1H, 5′′-H), 7.37 (d, *J* = 9.2 Hz, 1H, 5′-H), 5.43 (s, 2H, CH_2_), 3.89 (s, 3H, CH_3_). *δ*_C_ (101 MHz; (CD_3_)_2_SO) 160.50 (CONH), 155.32 (C-4′), 147.89 (C-3′′), 138.75 (C-3), 138.51 (C-1′′), 133.96 (C-6′′), 133.11 (C-1′), 132.75 (C-5), 130.24 (C-5′′), 126.68 (C-6′), 124.31 (C-2′), 123.04 (C-4′′), 122.06 (C-2′′), 117.98 (C-4), 116.15 (CN), 114.17 (C-5′), 100.54 (C-3′), 69.01 (CH_2_), 38.87 (CH_3_); HRMS (ESI): calcd.: 378.1197; found: {*m*/*z*: [M + H]^+^ 378.1195}.

#### *N*-(4-((3-(2-((4,6-Dimethylpyrimidin-2-yl)thio)acetamido)benzyl)oxy)phenyl)-1-methyl-1*H*-pyrazole-4-carboxamide (10)

Prepared according to literature.^23^

#### *N*-(3-Bromo-4-((3-(2-((4,6-dimethylpyrimidin-2-yl)thio)acetamido)benzyl)oxy)phenyl)-1-methyl-1*H*-pyrazole-4-carboxamide (11)

Prepared according to General procedure E from nitrobenzene 6 (1.00 g, 2.32 mmol). The obtained crude amine was reacted with 2-((4,6-dimethylpyrimidin-2-yl)thio)acetic acid (924 mg, 4.66 mmol) according to General procedure C. The reaction mixture was stirred at room temperature for 4 days. The crude product was purified by FCC (DCM/MeOH 98 : 2) to give 11 (610 mg, 1.05 mmol, 45% over two steps) as a pale-yellow solid; m.p. 96 °C; IR (ATR) *ν̃*/cm^−1^ 3284, 1646, 1582, 1551, 1526, 1490, 1441, 1339, 1264, 1223, 1048, 1005, 873, 755, 690; *δ*_H_ (400 MHz; (CD_3_)_2_SO) 10.29 (s, 1H, 3′′-NHCO), 9.79 (s, 1H, 4-CONH), 8.27 (s, 1H, 5-H), 8.02 (d, *J* = 2.5 Hz, 1H, 2′-H), 7.98 (s, 1H, 3-H), 7.69 (t, *J* = 1.9 Hz, 1H, 2′′-H), 7.60 (dd, *J* = 9.0, 2.5 Hz, 1H, 6′-H), 7.57–7.53 (m, 1H, 4′′-H), 7.34 (t, *J* = 7.8 Hz, 1H, 5′′-H), 7.18–7.17 (m, 1H, 5′-H or 6′′-H), 7.16–7.14 (m, 1H, 5′-H or 6′′-H), 6.96 (s, 1H, 5′′′-H), 5.15 (s, 2H, OCH_2_), 4.04 (s, 2H, SCH_2_), 3.89 (s, 3H, NCH_3_), 2.32 (s, 6H, 4′′′-CH_3_ and 6′′′-CH_3_). *δ*_C_ (101 MHz; (CD_3_)_2_SO) 169.32 (C-2′′′), 166.95 (C-4′′′ and C-6′′′), 166.62 (3′′-NHCO), 160.27 (4-CONH), 150.34 (C-4′), 139.19 (C-3′′), 138.71 (C-3), 137.39 (C-1′′), 133.53 (C-1′), 132.61 (C-5), 128.89 (C-5′′), 124.41 (C-2′), 122.26 (C-6′′), 120.22 (C-6′), 118.65 (C-4′′), 118.24 (C-4), 117.95 (C-2′′), 116.05 (C-5′′), 114.29 (C-5′), 110.68 (C-3′), 70.27 (OCH_2_), 38.84 (NCH_3_), 35.47 (SCH_2_), 23.33 (4′′-CH_3_ and 6′′-CH_3_); HRMS (ESI): calcd.: 581.0965; found: {*m*/*z*: [M + H]^+^ 581.0973}; purity (HPLC): 210 nm: >95%; 254 nm: >95% (method 1a).

#### *N*-(4-((3-(2-((4,6-Dimethylpyrimidin-2-yl)thio)acetamido)benzyl)oxy)-3-formylphenyl)-1-methyl-1*H*-pyrazole-4-carboxamide (12)

Bromoarene 11 (250 mg, 0.430 mmol), Pd(OAc)_2_ (2.90 mg, 12.9 μmol, 3 mol%), dppb (8.25 mg, 19.3 μmol, 4.5 mol%), *N*-formylsaccharin (287 mg, 1.29 mmol) and Na_2_CO_3_ (137 mg, 1.29 mmol) were added to a 30 mL glass tube, which was then evacuated and backfilled three times with N_2_. A degassed solution of Et_3_SiH (90.3 μL, 0.560 mmol) in DMF (1 mL) was added to the glass tube under N_2_ atmosphere. The mixture was stirred for 15 min at room temperature and subsequently warmed to 75 °C and stirred for another 19 h. The reaction mixture was cooled to room temperature, then diluted with EtOAc (15 mL) and washed with brine (3 × 15 mL). The organic phase was dried with a phase separation paper and concentrated *in vacuo*. The crude product was redissolved in MeOH (5 mL), sat. aq. NaHSO_3_ (25 mL) was added, stirred for approximately 30 s, diluted with H_2_O (25 mL), and then extracted with EtOAc (3 × 25 mL). The collected aqueous phase was basified with 50% NaOH (10 mL) and extracted with DCM (3 × 25 mL). The combined organic layers were dried with Na_2_SO_4_ and concentrated *in vacuo*. The obtained crude residue was purified by FCC (DCM/MeOH 98 : 2) to give 13 (7.0 mg, 13 μmol, 3%) as an off-white solid; m.p. 92 °C; IR (ATR) *ν̃*/cm^−1^ 3284, 2921, 2852, 1667, 1582, 1553, 1532, 1442, 1308, 1264, 1223, 1168, 1005, 873, 755, 690; *δ*_H_ (400 MHz; (CD_3_)_2_SO) 10.48 (s, 1H, CHO), 9.59 (s, 1H, 3′′-NHCO), 7.97 (dd, *J* = 9.0, 2.9 Hz, 1H, 6′-H), 7.90 (s, 1H, 5-H), 7.83–7.80 (m, 2H, 3-H and 2′-H), 7.63 (t, 1H, 2′′-H), 7.59 (s, 1H, 4-CONH), 7.38 (dt, 1H, 4′′-H), 7.33 (t, *J* = 7.7 Hz, 1H, 5′′-H), 7.17 (dt, *J* = 7.5 Hz, 1H, 6′′-H), 7.05 (d, *J* = 9.0 Hz, 1H, 5′-H), 6.85 (s, 1H, 5′′′-H), 5.18 (s, 2H, OCH_2_), 3.92 (s, 3H, NCH_3_), 3.85 (s, 2H, SCH_2_), 2.46 (s, 6H, 4′′′-CH_3_ and 6′′′-CH_3_). *δ*_C_ (101 MHz; (CD_3_)_2_SO) 189.29 (CHO), 170.40 (C-2′′′), 168.40 (3′′-NHCO), 168.11 (C-4′′′ and C-6′′′), 160.89 (4-CONH), 157.95 (C-4′), 139.17 (C-3′′), 138.30 (C-3), 137.70 (C-1′′), 132.25 (C-1′ or C-3′), 132.23 (C-5), 129.66 (C-5′′), 128.70 (C-6′), 125.49 (C-1′ or C-3′), 123.13 (C-6′′), 119.83 (C-2′), 119.61 (C-4′′), 119.04 (C-4), 118.68 (C-2′′), 117.09 (C-5′′′), 114.35 (C-5′), 70.98 (OCH_2_), 39.71 (NCH_3_), 35.90 (SCH_2_), 24.09 (4′′′-CH_3_ and 6′′′-CH_3_).; HRMS (ESI): calcd.: 529.1658; found: {*m*/*z*: [M–H]^−^ 529.1665}; purity (HPLC): 210 nm: >95%; 254 nm: >95% (method 1a).

#### (2-((3-(2-((4,6-Dimethylpyrimidin-2-yl)thio)acetamido)benzyl)oxy)-5-(1-methyl-1*H*-pyrazole-4-carboxamido)phenyl)boronic acid (13)

Prepared according to General procedure E from nitrobenzene 8 (15.0 mg, 37.9 μmol). The obtained crude amine was reacted with 2-((4,6-dimethylpyrimidin-2-yl)thio)acetic acid (15.2 mg, 76.5 μmol) according to General procedure B. The crude product was purified by FCC (DCM/MeOH 96 : 4) to give 13 (13.0 mg, 23.8 μmol, 62% over two steps) as a white solid; m.p. 193 °C; IR (ATR) *ν̃*/cm^−1^ 3435, 3325, 1683, 1625, 1581, 1550, 1530, 1495, 1443, 1374, 1322, 1265, 1225, 1166, 1043, 1017, 885, 815, 750; *δ*_H_ (400 MHz; (CD_3_)_2_SO) 10.30 (s, 1H, 3′′-NHCO), 9.69 (s, 1H, 4′-CONH), 8.27 (s, 1H, 5′-H), 7.99 (s, 1H, 3′-H), 7.83–7.76 (m, 2H, 4-H and 6-H), 7.74 (s, 2H, B(OH)_2_), 7.67 (s, 1H, 2′′-H), 7.55 (d, *J* = 8.1 Hz, 1H, 4′′-H), 7.34 (t, *J* = 7.8 Hz, 1H, 5′′-H), 7.18 (d, *J* = 7.6 Hz, 1H, 6′′-H), 7.01 (d, *J* = 8.9 Hz, 1H, 3-H), 6.95 (s, 1H, 5′′′-H), 5.13 (s, 2H, OCH_2_), 4.04 (s, 2H, SCH_2_), 3.88 (s, 3H, NCH_3_), 2.32 (s, 6H,4′′′-CH_3_ and 6′′′-CH_3_). *δ*_C_ (101 MHz; (CD_3_)_2_SO) 169.31 (C-2′′′), 166.96 (C-4′′′ and C-6′′′), 166.62 (3′′-NHCO), 160.12 (4′-CONH), 158.53 (C-2), 139.23 (C-3′′), 138.71 (C-3′), 137.76 (C-1′′), 132.44 (C-5), 132.33 (C-5′), 129.01 (C-5′′), 127.56 (C-4 or C-6), 123.46 (C-4 or C-6), 122.53 (C-6′′), 121.82 (C-1), 118.68 (C-4′), 118.61 (C-4′′), 118.13 (C-2′′), 116.06 (C-5′′′), 111.89 (C-5′′), 69.79 (OCH_2_), 38.82 (NCH_3_), 35.47 (SCH_2_), 23.33 (4′′′-CH_3_ and 6′′′-CH_3_); HRMS (ESI): calcd.: 545.1784; found: {*m*/*z*: [M–H]^−^ 545.1788}; purity (HPLC): 210 nm: 92%; 254 nm: >95% (method 1a).

#### *N*-(3-Cyano-4-((3-(2-((4,6-dimethylpyrimidin-2-yl)thio)acetamido)benzyl)oxy)phenyl)-1-methyl-1*H*-pyrazole-4-carboxamide (14)

Prepared according to General procedure E from nitrobenzene 9 (90.0 mg, 0.238 mmol). The obtained crude amine was reacted with 2-((4,6-dimethylpyrimidin-2-yl)thio)acetic acid (100 mg, 0.507 mmol) according to General procedure B. The crude product was purified by FCC (DCM/MeOH 96 : 4) to give 14 (82.5 mg, 0.156 mmol, 62% over two steps) as a yellow solid; m.p. 165 °C; IR (ATR) *ν̃*/cm^−1^ 3353, 2227, 1661, 1587, 1555, 1535, 1503, 1446, 1410, 1263, 1232, 1004, 860, 817, 752, 688; *δ*_H_ (400 MHz; (CD_3_)_2_SO) 10.31 (s, 1H, 3′′-NHCO), 9.94 (s, 1H, 4-CONH), 8.29 (s, 1H, 5-H), 8.07 (s, 1H, 2′-H), 7.99 (s, 1H, 3-H), 7.85 (d, *J* = 9.1 Hz, 1H, 6′-H), 7.67 (s, 1H, 2′′-H), 7.57 (d, *J* = 8.1 Hz, 1H, 4′′-H), 7.39–7.33 (m, 1H, 5′′-H), 7.33–7.28 (m, 1H, 5′-H), 7.17 (d, *J* = 7.6 Hz, 1H, 6′′-H), 6.94 (s, 1H, 5′′′-H), 5.25 (s, 2H, OCH_2_), 4.04 (s, 2H, SCH_2_), 3.89 (s, 3H, NCH_3_), 2.31 (s, 6H, 4′′′-CH_3_ and 6′′′-CH_3_). *δ*_C_ (101 MHz; (CD_3_)_2_SO) 169.31 (C-2′′′), 166.95 (C-4′′′ and C-6′′′), 166.66 (3′′-NHCO), 160.47 (4-CONH), 155.68 (C-4′), 139.28 (C-3′′), 138.74 (C-3), 136.78 (C-1′′), 132.78 (C-1′), 132.72 (C-5), 129.03 (C-5′′), 126.61 (C-6′), 124.30 (C-2′), 122.43 (C-6′′), 118.87 (C-4′′), 118.02 (C-2′), 116.28 (CN), 116.04 (C-5′′′), 114.08 (C-5′), 100.36 (C-3′), 70.20 (OCH_2_), 38.86 (NCH_3_), 35.48 (SCH_2_), 23.32 (4′′′-CH_3_ and 6′′′-CH_3_); HRMS (ESI): calcd.: 550.1637; found: {*m*/*z*: [M + Na]^+^ 550.1633}; purity (HPLC): 210 nm: >95%; 254 nm: >95% (method 1a).

#### 4-Amino-3-bromophenol (18)

Prepared according to General procedure E from nitroarene 15 (5.00 g, 22.9 mmol). The crude product was purified by FCC (DCM/MeOH 97 : 3) to give 4-amino-3-bromophenol (18) (3.85 g, 20.5 mmol, 89%) as a light pink solid. Analytical data are in alignment with literature.^51^

#### 4-Amino-3-iodophenol (19)

Prepared according to General procedure E from nitroarene 16 (1.00 g, 3.66 mmol). The crude product was purified by FCC (DCM/MeOH 97 : 3) to give aniline 19 (738 mg, 3.14 mmol, 86%) as a grey-white solid. Analytical data are in alignment with literature.^51^

#### 2-Chloro-4-((3-nitrobenzyl)oxy)aniline (20)

To a stirred solution of phenol 17 (500 mg, 3.41 mmol) in DMF (25 mL) was added K_2_CO_3_ (1.42 g, 10.2 mmol) and the reaction mixture was stirred for 30 minutes. 1-(Bromomethyl)-3-nitrobenzene (885 mg, 4.10 mmol) was then added and the reaction mixture was stirred at 0 °C for another 5 h. The reaction mixture was diluted with EtOAc and washed with brine (3 × 200 mL). The organic phase was dried using a phase separation paper and concentrated *in vacuo.* The crude product was purified by FCC (DCM) to give ether 20 (475 mg, 1.70 mmol, 50%) as an orange solid; m.p. 102 °C; IR (ATR) *ν̃*/cm^−1^ 3443, 3361, 3090, 2866, 1607, 1572, 1527, 1500, 1480, 1467, 1387, 1350, 1231, 1093, 1041, 901, 887, 823, 808, 796, 732; *δ*_H_ (400 MHz; (CD_3_)_2_SO) 8.27 (dt, *J* = 2.3, 0.6 Hz, 1H; 2′-H), 8.18 (ddd, *J* = 8.2, 2.4, 1.0 Hz, 1H, 4′-H), 7.87 (ddd, *J* = 7.7, 1.7, 1.0 Hz, 1H, 6′-H), 7.69 (t, *J* = 7.9 Hz, 1H, 5′-H), 6.97 (d, *J* = 2.7 Hz, 1H, 3-H), 6.81 (dd, *J* = 8.8, 2.8 Hz, 1H, 5-H), 6.75 (d, *J* = 8.8 Hz, 1H, 6-H), 5.15 (d, *J* = 0.8 Hz, 2H, CH_2_), 4.91 (s, 2H, NH_2_). *δ*_C_ (101 MHz; (CD_3_)_2_SO) 149.24 (C-4), 147.81 (C-3′), 139.75 (C-1′), 139.16 (C-1), 133.99 (C-6′), 130.00 (C-5′), 122.62 (C-4′), 121.88 (C-2′), 117.28 (C-2), 116.21 (C-6), 115.63 (C-3 or C-5), 115.53 (C-3 or C-5), 68.72 (CH_2_); HRMS (ESI): calcd.: 279.0531; found: {*m*/*z*: [M + H]^+^ 279.0532}.

#### 2-Bromo-4-((3-nitrobenzyl)oxy)aniline (21)

Prepared according to General procedure D from phenol 18 (3.85 g, 20.5 mmol) and 1-(bromomethyl)-3-nitrobenzene (5.31 g, 24.6 mmol). The crude product was purified by FCC (DCM + 0.5% MeOH) to give ether 21 (3.15 g, 9.75 mmol, 48%) as an orange solid; m.p. 84 °C; IR (ATR) *ν̃*/cm^−1^ 3417, 3340, 3073, 1707, 1601, 1581, 1525, 1499, 1387, 1343, 1318, 1228, 1094, 1050, 1030, 811, 729, 667; *δ*_H_ (400 MHz; (CD_3_)_2_SO) 8.28–8.25 (m, 1H, 2′-H), 8.18 (ddd, *J* = 8.2, 2.4, 1.0 Hz, 1H, 4′-H), 7.87 (ddd, 1H, 6′-H), 7.69 (t, *J* = 7.9 Hz, 1H, 5′-H), 7.11 (d, *J* = 2.8 Hz, 1H, 3-H), 6.85 (dd, *J* = 8.8, 2.8 Hz, 1H, 5-H), 6.76 (d, *J* = 8.8 Hz, 1H, 6-H), 5.15 (s, 2H, CH_2_), 4.88 (s, 2H, NH_2_). *δ*_C_ (101 MHz; (CD_3_)_2_SO) 149.41 (C-4), 147.81 (C-3′), 140.34 (C-1), 139.74 (C-1′), 133.99 (C-6′), 130.00 (C-5′), 122.62 (C-4′), 121.88 (C-2′), 118.37 (C-3), 116.30 (C-5 or C-6), 116.10 (C-5 or C-6), 107.43 (C-2), 68.75 (CH_2_); HRMS (EI): calcd.: 321.9953; found: {*m*/*z*: [M]˙^+^ 321.9940}.

#### 2-Iodo-4-((3-nitrobenzyl)oxy)aniline (22)

Prepared according to General procedure D from phenol 19 (682 mg, 2.90 mmol) and 1-(bromomethyl)-3-nitrobenzene (752 mg, 3.48 mmol). The crude product was purified by FCC (DCM/hexanes 90 : 10) to give ether 22 (405 mg, 1.09 mmol, 38%) as a brown solid; m.p. 109 °C; IR (ATR) *ν̃*/cm^−1^ 3420, 3345, 3050, 2856, 1615, 1599, 1569, 1519, 1493, 1452, 1346, 1317, 1230, 1091, 1043, 930, 869, 802, 729, 665; *δ*_H_ (500 MHz; (CD_3_)_2_SO) 8.27 (td, *J* = 1.7, 0.8 Hz, 1H, 2′-H), 8.18 (ddd, *J* = 8.3, 2.4, 1.1 Hz, 1H, 4′-H), 7.87 (ddd, *J* = 7.6, 1.7, 1.0 Hz, 1H, 6′-H), 7.69 (t, *J* = 7.9 Hz, 1H, 5′-H), 7.28 (d, *J* = 2.8 Hz, 1H, 3-H), 6.87 (dd, *J* = 8.8, 2.9 Hz, 1H, 5-H), 6.72 (d, *J* = 8.8 Hz, 1H, 6-H), 5.13 (s, 2H, CH_2_), 4.81 (s, 2H, NH_2_). *δ*_C_ (126 MHz; (CD_3_)_2_SO) 149.63 (C-4), 147.81 (C-3′), 143.22 (C-1), 139.80 (C-1′), 133.99 (C-6′), 129.99 (C-5′), 124.28 (C-3), 122.61 (C-4′), 121.88 (C-2′), 117.04 (C-5), 114.88 (C-6), 83.11 (C-2), 68.73 (CH_2_); HRMS (ESI): calcd.: 370.9887; found: {*m*/*z*: [M + H]^+^ 370.9886}.

#### *N*-(2-Chloro-4-((3-nitrobenzyl)oxy)phenyl)-1-methyl-1*H*-pyrazole-4-carboxamide (23)

Prepared according to General procedure A from 1-methyl-1*H*-pyrazole-4-carboxylic acid (195 mg, 1.55 mmol) and aniline 20 (440 mg, 1.55 mmol). The reaction mixture was stirred at 65 °C for 3 days. The crude product was purified by recrystallisation from DCM to give amide 23 (363 mg, 0.938 mmol, 61%) as an off-white solid; m.p. 191 °C; IR (ATR) *ν̃*/cm^−1^ 3413, 3119, 1671, 1584, 1552, 1527, 1477, 1349, 1279, 1248, 1216, 1093, 1050, 1003, 876, 850, 807, 800, 750, 731, 708; *δ*_H_ (400 MHz; (CD_3_)_2_SO) 9.53 (s, 1H, CONH), 8.33 (t, *J* = 2.0 Hz, 1H, 2′′-H), 8.27 (s, 1H, 5-H), 8.21 (ddd, *J* = 8.2, 2.4, 1.1 Hz, 1H, 4′′-H), 7.97 (s, 1H, 3-H), 7.93 (ddd, *J* = 7.7, 1.7, 1.0 Hz, 1H, 6′′-H), 7.72 (t, *J* = 7.9 Hz, 1H, 5′′-H), 7.41 (d, *J* = 8.8 Hz, 1H, 6′-H), 7.26 (d, *J* = 2.8 Hz, 1H, 3′-H), 7.06 (dd, *J* = 8.8, 2.9 Hz, 1H, 5′-H), 5.32 (s, 2H, CH_2_), 3.88 (s, 3H, CH_3_). *δ*_C_ (101 MHz; (CD_3_)_2_SO) 160.71 (CONH), 156.35 (C-4′), 147.87 (C-3′′), 139.04 (C-1′′), 138.83 (C-3), 134.14 (C-6′′), 132.61 (C-5), 130.53 (C-2′), 130.13 (C-5′′), 129.70 (C-6′), 128.13 (C-1′), 122.88 (C-4′′), 122.11 (C-2′′), 117.82 (C-4), 115.47 (C-3′), 114.23 (C-5′), 68.41 (CH_2_), 38.83 (CH_3_); HRMS (ESI): calcd.: 387.0855; found: {*m*/*z*: [M + H]^+^ 387.0854}.

#### *N*-(2-Bromo-4-((3-nitrobenzyl)oxy)phenyl)-1-methyl-1*H*-pyrazole-4-carboxamide (24)

Prepared according to General procedure A from 1-methyl-1*H*-pyrazole-4-carboxylic acid (1.23 g, 9.75 mmol) and aniline 21 (3.15 g, 9.75 mmol). The reaction mixture was stirred at 65 °C for 6 days. The crude product was purified by recrystallisation from DCM to give amide 24 (2.97 g, 6.89 mmol, 71%) as a white solid; m.p. 192 °C; IR (ATR) *ν̃*/cm^−1^ 3399, 3116, 2992, 1674, 1605, 1587, 1523, 1475, 1410, 1385, 1342, 1279, 1217, 1046, 1028, 852, 811, 749, 732.; *δ*_H_ (400 MHz; (CD_3_)_2_SO) 9.52 (s, 1H, CONH), 8.33 (t, *J* = 2.0 Hz, 1H, 2′′-H), 8.26 (s, 1H, 5-H), 8.21 (dt, *J* = 8.2, 1.6 Hz, 1H, 4′′-H), 7.97 (s, 1H, 3-H), 7.93 (d, *J* = 7.7 Hz, 1H, 6′′-H), 7.72 (t, *J* = 7.9 Hz, 1H, 5′′-H), 7.41 (d, *J* = 2.8 Hz, 1H, 3′-H), 7.38 (d, *J* = 8.8 Hz, 1H, 6′-H), 7.10 (dd, *J* = 8.8, 2.8 Hz, 1H, 5′-H), 5.32 (s, 2H, CH_2_), 3.88 (s, 3H, CH_3_). *δ*_C_ (101 MHz; (CD_3_)_2_SO) 160.45 (CONH), 156.24 (C-4′), 147.61 (C-3′′), 138.79 (C-1′′), 138.55 (C-3), 133.88 (C-6′′), 132.31 (C-5), 129.87 (C-6′ or C-5′′), 129.70 (C-6′ or C-5′′), 129.37 (C-1′), 122.61 (C-4′′), 121.84 (C-2′′), 121.24 (C-2′), 118.14 (C-3′), 117.62 (C-4), 114.54 (C-5′), 68.16 (CH_2_), 38.57 (CH_3_); HRMS (ESI): calcd.: 429.0204; found: {*m*/*z*: [M–H]^−^ 429.0203}.

#### *N*-(2-Iodo-4-((3-nitrobenzyl)oxy)phenyl)-1-methyl-1*H*-pyrazole-4-carboxamide (25)

Prepared according to General procedure A from 1-methyl-1*H*-pyrazole-4-carboxylic acid (122 mg, 0.964 mmol) and aniline 22 (357 mg, 0.964 mmol). The reaction mixture was stirred at 65 °C for 16 h. The crude product was purified by FCC (DCM/MeOH 98 : 2) to give amide 25 (216 mg, 0.452 mmol, 47%) as a brown solid; m.p. 213 °C; IR (ATR) *ν̃*/cm^−1^ 3378, 3125, 1657, 1579, 1553, 1520, 1440, 1399, 1347, 1299, 1273, 1218, 1095, 1057, 998, 866, 846, 808, 752, 727, 697; *δ*_H_ (500 MHz; (CD_3_)_2_SO) 9.51 (s, 1H, CONH), 8.33 (t, *J* = 2.0 Hz, 1H, 2′′-H), 8.25 (s, 1H, 6-H), 8.21 (ddd, *J* = 8.3, 2.4, 1.1 Hz, 1H, 4′′-H), 7.97 (s, 1H, 4-H), 7.94–7.91 (m, 1H, 6′′-H), 7.72 (t, *J* = 7.9 Hz, 1H, 5′′-H), 7.58 (d, *J* = 2.8 Hz, 1H, 3′-H), 7.28 (d, *J* = 8.7 Hz, 1H, 6′-H), 7.11 (dd, *J* = 8.7, 2.8 Hz, 1H, 5′-H), 5.30 (s, 2H, CH_2_), 3.89 (s, 3H, CH_3_). *δ*_C_ (126 MHz; (CD_3_)_2_SO) 160.69 (CONH), 156.46 (C-4′), 147.86 (C-3′′), 139.13 (C-1′′), 138.78 (C-4), 134.12 (C-6′′), 133.10 (C-1′), 132.52 (C-6), 130.12 (C-5′′), 129.26 (C-6′), 124.29 (C-3′), 122.84 (C-4′′), 122.07 (C-2′′), 118.08 (C-5), 115.41 (C-5′), 99.74 (C-2′), 68.33 (CH_2_), 38.24 (CH_3_); HRMS (ESI): calcd.: 479.0211; found: {*m*/*z*: [M + H]^+^ 479.0208}.

#### 1-Methyl-*N*-(4-((3-nitrobenzyl)oxy)-2-(4,4,5,5-tetramethyl-1,3,2-dioxaborolan-2-yl)phenyl)-1*H*-pyrazole-4-carboxamide (26)

PdCl_2_(dppf)·DCM (20.4 mg, 27.8 μmol), bis(pinacolato)diboron (141 mg, 0.557 mmol), bromoarene 24 (120 mg, 0.278 mmol), and KOAc (109 mg, 1.11 mmol) were dissolved in degassed 1,4-dioxane (15 mL) under N_2_ atmosphere and stirred at 95 °C for 20 h. The reaction mixture was cooled to room temperature and then filtered. The filtrate was diluted with EtOAc (50 mL). Water (50 mL) was added, the resulting two phases were separated, and the aqueous phase was extracted with EtOAc (3 × 60 mL). The combined organic phases were dried using a phase separation paper and concentrated *in vacuo.* The crude product was purified by FCC (hexanes/EtOAc 4 : 96 + 3% MeOH) to give boronic acid pinacol ester 26 (34.5 mg, 72.1 μmol, 26%) as a pale-yellow solid; m.p. 199 °C; IR (ATR) *ν̃*/cm^−1^ 2967, 1641, 1606, 1530, 1482, 1349, 1315, 1266, 1157, 1129, 1013, 876, 802, 726; *δ*_H_ (400 MHz; (CD_3_)_2_SO) 11.67 (s, 1H, CONH), 8.42 (s, 1H, 5-H), 8.31 (t, *J* = 2.0 Hz, 1H, 2′′-H), 8.19 (ddd, *J* = 8.2, 2.4, 1.0 Hz, 1H, 4′′-H), 8.13 (s, 1H, 3-H), 7.93–7.90 (m, 1H, 6′′-H), 7.70 (t, *J* = 7.9 Hz, 1H, 5′′-H), 7.16 (d, 1H, 6′-H), 6.98–6.94 (m, 2H, 5′-H), 5.26 (s, 2H, CH_2_), 3.95 (s, 3H, NCH_3_), 1.16 (s, 12H, (CH_3_)_4_). *δ*_C_ (101 MHz; (CD_3_)_2_SO) 159.77 (CONH), 155.40 (C-4′), 147.83 (C-3′′), 139.95 (C-1′′), 139.05 (C-3), 133.89 (C-6′′), 133.72 (C-5), 132.37 (C-1′), 130.07 (C-5′′), 122.58 (C-4′′), 121.85 (C-2′′), 117.34 (C-3′ and C-6′), 114.48 (C-5′), 113.92 (C-4), 79.44 ((**C**(CH_3_)_2_)_2_), 67.98 (CH_2_), 38.89 (NCH_3_), 26.05 ((CH_3_)_4_); HRMS (ESI): calcd.: 479.2096; found: {*m*/*z*: [M + H]^+^ 479.2092}.

#### (2-(1-Methyl-1*H*-pyrazole-4-carboxamido)-5-((3-nitrobenzyl)oxy)phenyl)boronic acid (27)

To a stirred solution of boronic acid pinacol ester 26 (330 mg, 0.690 mmol) in THF/water 4 : 1 v/v (25 mL) was added sodium periodate (738 mg, 3.45 mmol) and the reaction mixture was stirred vigorously at room temperature for 45 minutes. 1 M HCl (0.828 mmol, 0.828 mL) was then added, and the mixture stirred for 15 h. The milky suspension was diluted with DCM (15 mL), filtered and the residue washed with DCM (1×) and MeOH (1×). The filtrate was discarded and the solid residue collected to give boronic acid 27 (274 mg, 0.692 mmol, quant.) as an off-white solid; m.p. 260 °C (decomposition); IR (ATR) *ν̃*/cm^−1^ 3545, 3295, 3107, 2858, 1640, 1602, 1532, 1476, 1346, 1206, 1098, 1015, 882, 805, 739; *δ*_H_ (400 MHz; CF_3_COOD) 9.00 (s, 1H, 3′′-H), 8.65 (s, 1H, 5′′-H), 8.46 (s, 1H, 2′-H), 8.34 (d, *J* = 8.8 Hz, 1H, 4′-H), 7.92 (d, *J* = 7.6 Hz, 1H, 6′-H), 7.69 (t, *J* = 8.0 Hz, 1H, 5′-H), 7.38–7.33 (m, 2H, 3-H and 6-H), 7.25 (dd, *J* = 8.7, 2.8 Hz, 1H, 4-H), 5.38 (s, 2H, CH_2_), 4.29 (s, 3H, CH_3_). *δ*_C_ (101 MHz; CF_3_COOD) 160.53 (C-5), 159.44 (CONH), 149.88 (C-3′), 140.65 (C-1′), 139.87 (C-3′′), 139.00 (C-5′′), 136.12 (C-6′), 132.11 (C-2), 131.70 (C-5′), 125.19 (C-4′), 124.13 (C-2′), 120.57 (C-3), 119.89 (C-4), 117.07 (C-6), 114.82 (C-4′′), 71.13 (CH_2_), 40.45 (CH_3_); HRMS (ESI): calcd.: 379.1208; found: {*m*/*z*: [M–H_2_O + H]^+^ 379.1205}.

#### *N*-(2-Cyano-4-((3-nitrobenzyl)oxy)phenyl)-1-methyl-1*H*-pyrazole-4-carboxamide (28)

To a stirred solution of bromoarene 24 (350 mg, 0.812 mmol) in dry DMF (4 mL) was added CuCN (147 mg, 1.62 mmol). The reaction mixture was stirred at 150 °C for 22 h. Afterwards, the reaction mixture was concentrated *in vacuo* and the crude residue was resuspended in 20 mL aq. NH_4_OH. The resulting suspension was vacuum filtered, and the residue was washed with aq. NH_4_OH until the filtrate was no longer blue. The filtrate was discarded, and the filter residue was further washed sequentially with water (3 × 25 mL), DCM (4 × 25 mL), EtOAc (2 × 25 mL) and MeOH (2 × 25 mL). The filter residue was collected to give nitrile 28 (142 mg, 0.376 mmol, 46%) as a brownish-yellow solid; m.p. 197 °C; IR (ATR) *ν̃*/cm^−1^ 3293, 3117, 3074, 2922, 2229, 1651, 1615, 1587, 1518, 1483, 1419, 1387, 1348, 1286, 1227, 1161, 1101, 1048, 1003, 977, 886, 875, 823, 730; *δ*_H_ (400 MHz; (CD_3_)_2_SO) 10.06 (s, 1H, CONH), 8.34 (t, *J* = 2.0 Hz, 1H, 2′′-H), 8.29 (s, 1H, 5-H), 8.24–8.20 (m, 1H, 4′′-H), 7.99 (s, 1H, 3-H), 7.95–7.91 (m, 1H, 6′′-H), 7.72 (t, *J* = 8.0 Hz, 1H, 5′′-H), 7.57 (d, *J* = 2.6 Hz, 1H, 3′-H), 7.44–7.40 (m, 2H, 5′-H and 6′-H), 5.35 (s, 2H, OCH_2_), 3.90 (s, 3H, CH_3_). *δ*_C_ (101 MHz; (CD_3_)_2_SO) 160.77 (CONH), 155.43 (C-4′), 147.88 (C-3′′), 138.90 (C-1′′), 138.77 (C-3), 134.23 (C-6′′), 133.61 (C-1′), 132.86 (C-5), 130.16 (C-5′′), 128.68 (C-6′), 122.97 (C-4′′), 122.22 (C-2′′), 121.10 (C-5′), 117.98 (C-3′), 117.45 (C-4), 116.72 (CN), 110.38 (C-2′), 68.58 (OCH_2_), 38.89 (CH_3_); HRMS (ESI): calcd.: 376.1046; found: {*m*/*z*: [M–H]^−^ 376.1053}.

#### *N*-(2-Chloro-4-((3-(2-((4,6-dimethylpyrimidin-2-yl)thio)acetamido)benzyl)oxy)phenyl)-1-methyl-1*H*-pyrazole-4-carboxamide (29)

Prepared according to General procedure E from nitroarene 23 (150 mg, 0.388 mmol). The obtained crude amine was reacted with 2-((4,6-dimethylpyrimidin-2-yl)thio)acetic acid (154 mg, 0.779 mmol) according to General procedure C. The reaction mixture was stirred at room temperature for 2 days. The crude product was purified by FCC (DCM/MeOH 98 : 2) to give 29 (145 mg, 0.269 mmol, 69% over two steps) as a pale-yellow solid; m.p. 86 °C; IR (ATR) *ν̃*/cm^−1^ 3270, 3081, 1651, 1580, 1552, 1514, 1490, 1440, 1264, 1203, 1045, 1004, 874, 784, 755, 690; *δ*_H_ (400 MHz; (CD_3_)_2_SO) 10.29 (s, 1H, 3′′-NHCO), 9.51 (s, 1H, 4-CONH), 8.26 (s, 1H, 5-H), 7.97 (s, 1H, 3-H), 7.69 (t, *J* = 1.9 Hz, 1H, 2′′-H), 7.54 (ddd, *J* = 8.2, 2.2, 1.1 Hz, 1H, 4′′-H), 7.38 (d, *J* = 8.9 Hz, 1H, 6′-H), 7.33 (t, *J* = 7.8 Hz, 1H, 5′′-H), 7.18 (d, *J* = 2.8 Hz, 1H, 3′-H), 7.16–7.12 (m, 1H, 6′′-H), 6.99 (dd, *J* = 8.8, 2.8 Hz, 1H, 5′-H), 6.96 (s, 1H, 5′′′-H), 5.13 (s, 2H, OCH_2_), 4.04 (s, 2H, SCH_2_), 3.88 (s, 3H, NCH_3_), 2.32 (d, *J* = 0.5 Hz, 6H, 4′′′-CH_3_ and 6′′′-CH_3_). *δ*_C_ (101 MHz; (CD_3_)_2_SO) 169.31 (C-2′′′), 166.96 (C-4′′′ and C-6′′′), 166.63 (3′′-NHCO), 160.71 (4-CONH), 156.70 (C-4′), 139.23 (C-3′′), 138.82 (C-3), 137.33 (C-1′′), 132.59 (C-5), 130.48 (C-2′), 129.64 (C-6′), 128.92 (C-5′′), 127.81 (C-1′), 122.48 (C-6′′), 118.65 (C-4′′), 118.08 (C-2′′), 117.85 (C-4), 116.06 (C-5′′′), 115.32 (C-3′), 114.18 (C-5′), 69.65 (OCH_2_), 38.82 (NCH_3_), 35.47 (SCH_2_), 23.32 (4′′′-CH_3_ and 6′′′-CH_3_); HRMS (ESI): calcd.: 537.1470; found: {*m*/*z*: [M + H]^+^ 537.1466}; purity (HPLC): 210 nm: >95%; 254 nm: 89% (method 1a).

#### *N*-(2-Bromo-4-((3-(2-((4,6-dimethylpyrimidin-2-yl)thio)acetamido)benzyl)oxy)phenyl)-1-methyl-1*H*-pyrazole-4-carboxamide (30)

Prepared according to General procedure E from nitroarene 24 (1.36 g, 3.14 mmol). The obtained crude amine was reacted with 2-((4,6-dimethylpyrimidin-2-yl)thio)acetic acid (1.25 g, 6.31 mmol) according to General procedure C. The reaction mixture was stirred at room temperature for 4 days. The crude product was purified by FCC (DCM/MeOH 98 : 2) to give 30 (1.18 g, 2.03 mmol, 64% over two steps) as a yellow solid; m.p. 83 °C; IR (ATR) *ν̃*/cm^−1^ 3269, 2923, 1651, 1581, 1553, 1514, 1490, 1441, 1265, 1219, 1203, 1032, 1004, 872, 785, 757, 692; *δ*_H_ (500 MHz; (CD_3_)_2_SO) 10.29 (s, 1H, 3′′-NHCO), 9.50 (s, 1H, 4-CONH), 8.26 (s, 1H, 5-H), 7.96 (s, 1H, 3-H), 7.68 (t, *J* = 1.8 Hz, 1H, 2′′-H), 7.54 (dt, *J* = 8.0, 1.7 Hz, 1H, 4′′-H), 7.37–7.31 (m, 3H, 3′-H, 6′-H and 5′′-H), 7.14 (dt, *J* = 7.7 Hz, 1H, 6′′-H), 7.04 (dd, *J* = 8.8, 2.8 Hz, 1H, 5′-H), 6.96 (s, 1H, 5′′′-H), 5.13 (s, 2H, OCH_2_), 4.04 (s, 2H, SCH_2_), 3.88 (s, 3H, NCH_3_), 2.32 (s, 6H, 4′′′-CH_3_ and 6′′′-CH_3_). *δ*_C_ (126 MHz; (CD_3_)_2_SO) 169.31 (C-2′′′), 166.96 (C-4′′′ and C-6′′′), 166.63 (3′′-NHCO), 160.71 (4-CONH), 156.85 (C-4′), 139.23 (C-3′′), 138.81 (C-3), 137.34 (C-1′′), 132.57 (C-5), 129.90 (C-6′), 129.31 (C-1′), 128.93 (C-5′′), 122.47 (C-6′′), 121.47 (C-2′), 118.64 (C-4′′), 118.26 (C-3′), 118.06 (C-2′′), 117.91 (C-4), 116.06 (C-5′′′), 114.73 (C-5′), 69.65 (OCH_2_), 38.83 (NCH_3_), 35.47 (SCH_2_), 23.33 (4′′′-CH_3_ and 6′′′-CH_3_); HRMS (ESI): calcd.: 581.0965; found: {*m*/*z*: [M + H]^+^ 581.0967}; purity (HPLC): 210 nm: >95%; 254 nm: >95% (method 1a).

#### *N*-(4-((3-(2-((4,6-Dimethylpyrimidin-2-yl)thio)acetamido)benzyl)oxy)-2-iodophenyl)-1-methyl-1*H*-pyrazole-4-carboxamide (31)

Prepared according to General procedure E from nitroarene 25 (100 mg, 0.209 mmol). The obtained crude amine was reacted with 2-((4,6-dimethylpyrimidin-2-yl)thio)acetic acid (83.1 mg, 0.419 mmol) according to General procedure C. The reaction mixture was stirred at room temperature for 18 h. The crude product was purified by FCC (DCM/MeOH 98 : 2) to give 31 (83.7 mg, 0.133 mmol, 64% over two steps) as a beige solid; m.p. 107 °C; IR (ATR) *ν̃*/cm^−1^ 3269, 2919, 1649, 1581, 1552, 1511, 1487, 1441, 1265, 1205, 1004, 978, 867, 784, 756, 691; *δ*_H_ (500 MHz; (CD_3_)_2_SO) 10.29 (s, 1H, 3′′-NHCO), 9.49 (s, 1H, 4-CONH), 8.25 (s, 1H, 5-H), 7.97 (s, 1H, 3-H), 7.68 (t, *J* = 1.9 Hz, 1H, 2′′-H), 7.54 (dt, *J* = 8.3, 1.5 Hz, 1H, 4′′-H), 7.51 (d, *J* = 2.8 Hz, 1H, 3′-H), 7.33 (t, *J* = 7.8 Hz, 1H, 5′′-H), 7.25 (d, *J* = 8.7 Hz, 1H, 6′-H), 7.13 (d, *J* = 7.6 Hz, 1H, 6′′-H), 7.05 (dd, *J* = 8.7, 2.9 Hz, 1H, 5′-H), 6.97 (s, 1H, 5′′′-H), 5.11 (s, 2H, OCH_2_), 4.04 (s, 2H, SCH_2_), 3.88 (s, 3H, NCH_3_), 2.32 (s, 6H, 4′′′-CH_3_ and 6′′′-CH_3_). *δ*_C_ (126 MHz; (CD_3_)_2_SO) 169.31 (C-2′′′), 166.96 (C-4′′′ and C-6′′′), 166.62 (3′′-NHCO), 160.69 (4-CONH), 156.81 (C-4′), 139.22 (C-3′′), 138.78 (C-3), 137.42 (C-1′′), 132.78 (C-1′), 132.51 (C-5), 129.20 (C-6′), 128.91 (C-5′′), 124.20 (C-3′), 122.44 (C-6′′), 118.62 (C-4′′), 118.10 (C-4), 118.02 (C-2′′), 116.06 (C-5′′′), 115.31 (C-5′), 99.71 (C-2′), 69.56 (OCH_2_), 38.83 (NCH_3_), 35.47 (SCH_2_), 23.33 (4′′′-CH_3_ and 6′′′-CH_3_); HRMS (ESI): calcd.: 629.0826; found: {*m*/*z*: [M + H]^+^ 629.0822}; purity (HPLC): 210 nm: >95%; 254 nm: >95% (method 1c).

#### *N*-(4-((3-(2-((4,6-Dimethylpyrimidin-2-yl)thio)acetamido)benzyl)oxy)-2-formylphenyl)-1-methyl-1*H*-pyrazole-4-carboxamide (32)

30 (150 mg, 0.258 mmol), Pd(OAc)_2_ (1.74 mg, 7.74 μmol, 3 mol%), dppb (4.95 mg, 11.6 μmol, 4.5 mol%), *N*-formylsaccharin (172 mg, 0.774 mmol), and Na_2_CO_3_ (82.0 mg, 0.774 mmol) were added to a 30 mL glass tube, which was then evacuated and backfilled three times with N_2_. A degassed solution of Et_3_SiH (54.2 μL, 0.335 mmol) in DMF (0.6 mL) was added to the glass tube under N_2_ atmosphere. The mixture was stirred for 15 min at room temperature and subsequently warmed to 75 °C and stirred for another 19 h. The reaction mixture was cooled to room temperature, then diluted with EtOAc (15 mL) and washed with brine (3 × 15 mL). The organic phase was dried with a phase separation paper and concentrated *in vacuo*. The crude product was redissolved in MeOH (5 mL), sat. aq. NaHSO_3_ (25 mL) was added, stirred for approximately 30 seconds, diluted with H_2_O (25 mL), and then extracted with EtOAc (3 × 25 mL). The aqueous phase was basified with 50% NaOH (10 mL) and extracted with DCM (3 × 25 mL). The combined organic layers were dried with Na_2_SO_4_ and concentrated *in vacuo*. The obtained crude residue was purified by FCC (DCM/MeOH 98 : 2) to give 32 (3.8 mg, 7.2 μmol, 3%) as a yellow solid; m.p. 87 °C; IR (ATR) *ν̃*/cm^−1^ 3232, 2921, 2852, 1727, 1654, 1582, 1553, 1529, 1487, 1435, 1286, 1264, 1220, 1154, 1031, 893, 785, 696; *δ*_H_ (500 MHz; (CD_3_)_2_SO) 11.45 (s, 1H, 4-CONH), 9.91 (s, 1H, CHO), 9.58 (s, 1H, 3′′-NHCO), 8.76 (d, *J* = 10.1 Hz, 1H, 6′-H), 7.96 (s, 2H, 3-H and 5-H), 7.63 (t, 1H, 2′′-H), 7.38 (dt, *J* = 8.0, 1.6 Hz, 1H, 4′′-H), 7.32 (t, *J* = 7.8 Hz, 1H, 5′′-H), 7.28–7.25 (m, 2H, 3′-H and 5′-H), 7.16 (d, *J* = 7.4 Hz, 1H, 6′′-H), 6.85 (s, 1H, 5′′′-H), 5.11 (s, 2H, OCH_2_), 3.95 (s, 3H, NCH_3_), 3.85 (s, 2H, SCH_2_), 2.47 (s, 6H, 4′′′-CH_3_ and 6′′′-CH_3_). *δ*_C_ (126 MHz; (CD_3_)_2_SO) 195.98 (CHO), 170.43 (C-2′′′), 168.31 (3′′-NHCO), 168.11 (4′′′-CH_3_ and 6′′′-CH_3_), 161.20 (4-CONH), 154.24 (C-4′), 139.16 (C-3′′), 139.03 (C-3), 138.00 (C-1′′), 135.85 (C-1′), 131.99 (C-5), 129.60 (C-5′′), 123.70 (C-5′), 123.20 (C-6′′), 122.70 (C-2′), 121.61 (C-6′), 121.03 (C-3′), 119.76 (C-4), 119.46 (C-4′′), 118.76 (C-2′′), 117.06 (C-5′′′), 70.71 (OCH_2_), 39.73 (NCH_3_), 35.90 (SCH_2_), 30.09 (4′′′-CH_3_ and 6′′′-CH_3_); HRMS (ESI): calcd.: 553.1633; found: {*m*/*z*: [M + Na]^+^ 553.1633}; purity (HPLC): 210 nm: >95%; 254 nm: >95% (method 1b).

#### (5-((3-(2-((4,6-Dimethylpyrimidin-2-yl)thio)acetamido)benzyl)oxy)-2-(1-methyl-1*H*-pyrazole-4-carboxamido)phenyl)boronic acid (33)

Prepared according to General procedure F from nitroarene 27 (90.0 mg, 0.227 mmol). The obtained crude amine was dissolved in dry DMF (3 mL) and bromoacetyl bromide (23.8 μL, 0.274 mmol) was added. The reaction mixture was stirred at room temperature for 30 minutes. The solution was then diluted with EtOAc (50 mL) and washed with brine (3 × 20 mL). The organic phase was dried using a phase separation paper and concentrated *in vacuo*. The brown oily crude product was redissolved in dry DMF (8 mL), 4,6-dimethylpyrimidine-2-thiol (64.5 mg, 0.46 mmol) and *t*-BuOK (51.6 mg, 0.46 mmol) were added, and the reaction mixture was stirred at room temperature for 19 h. The solution was then diluted with EtOAc (150 mL) and washed with brine (3 × 100 mL). The organic phase was dried using a phase separation paper and concentrated *in vacuo*. The crude product was purified by FCC (DCM/10% NH_3_ in MeOH 88 : 12) to give product 33 (32.1 mg, 58.7 μmol, 26% over three steps) as a pale-yellow solid; m.p. 165 °C; IR (ATR) *ν̃*/cm^−1^ 3271, 2922, 2853, 1667, 1633, 1601, 1581, 1553, 1532, 1480, 1441, 1312, 1264, 1205, 1009, 874, 787, 749, 690; *δ*_H_ (400 MHz; (CD_3_)_2_SO) 11.78 (s, 1H, 4′-CONH), 10.20 (s, 1H, 3′′-NHCO), 8.25 (s, 1H, 5′-H), 7.96 (s, 1H, 3′-H), 7.62 (d, *J* = 8.3 Hz, 1H, 3-H), 7.58 (s, 1H, 2′′-H), 7.53 (d, *J* = 8.2 Hz, 1H, 4′′-H), 7.30 (d, *J* = 3.0 Hz, 1H, 6-H), 7.21 (t, *J* = 7.8 Hz, 1H, 5′′-H), 7.04 (d, *J* = 7.6 Hz, 1H, 6′′-H), 6.94 (dd, *J* = 8.9, 3.0 Hz, 1H, 4-H), 6.86 (s, 1H, 5′′′-H), 5.00 (s, 2H, OCH_2_), 3.98 (s, 2H, SCH_2_), 3.71 (s, 3H, NCH_3_), 2.28 (s, 6H, 4′′′-CH_3_ and 6′′′-CH_3_). *δ*_C_ (101 MHz; (CD_3_)_2_SO) 169.29 (C-2′′′), 166.90 (C-4′′′ and C-6′′′), 166.57 (3′′-NHCO), 159.32 (4′-CONH), 155.13 (C-5) 139.07 (C-3′′), 138.65 (C-3′), 137.98 (C-1′′), 133.14 (C-2 and C-5′), 128.68 (C-5′′), 122.30 (C-6′′), 119.01 (C-6), 118.40 (C-4′′), 117.91 (C-2′′), 117.72 (C-3), 115.98 (C-4′ and C-5′′′), 114.14 (C-4), 69.07 (OCH_2_), 38.75 (NCH_3_), 35.41 (SCH_2_), 23.28 (4′′′-CH_3_ and 6′′′-CH_3_); HRMS (ESI): calcd.: 527.1673; found: {*m*/*z*: [M–H_2_O–H]^−^ 527.1680}; purity (HPLC): 210 nm: >95%; 254 nm: >95% (method 2a).

#### *N*-(2-Cyano-4-((3-(2-((4,6-dimethylpyrimidin-2-yl)thio)acetamido)benzyl)oxy)phenyl)-1-methyl-1*H*-pyrazole-4-carboxamide (34)

Prepared according to General procedure E from nitroarene 28 (130 mg, 0.344 mmol). The obtained crude amine was reacted with 2-((4,6-dimethylpyrimidin-2-yl)thio)acetic acid (103 mg, 0.520 mmol) according to General procedure C. The reaction mixture was stirred at room temperature for 7 days. The crude product was purified by FCC (DCM/10% NH_3_ in MeOH 99 : 1) to give 34 (73.4 mg, 0.139 mmol, 40% over two steps) as a yellow solid; m.p. 93 °C; IR (ATR) *ν̃*/cm^−1^ 3271, 2922, 2853, 2229, 1652, 1581, 1552, 1490, 1441, 1265, 1226, 1161, 1003, 873, 785, 756, 691; *δ*_H_ (500 MHz; (CD_3_)_2_SO) 10.30 (s, 1H, 3′′-NHCO), 10.04 (s, 1H, 4-CONH), 8.29 (s, 1H, 5-H), 7.98 (s, 1H, 3-H), 7.70 (t, *J* = 2.0 Hz, 1H, 2′′-H), 7.54 (dt, *J* = 8.1, 1.9 Hz, 1H, 4′′-H), 7.50 (d, *J* = 2.9 Hz, 1H, 3′-H or 5′-H), 7.41 (d, *J* = 8.9 Hz, 1H, 6′-H), 7.36–7.35 (m, 1H, 5′′-H), 7.34–7.32 (m, 1H, 3′-H or 5′-H), 7.14 (d, *J* = 7.5 Hz, 1H, 6′′-H), 6.96 (s, 1H, 5′′′-H), 5.16 (s, 2H, OCH_2_), 4.04 (s, 2H, SCH_2_), 3.90 (s, 3H, NCH_3_), 2.32 (s, 6H, 4′′′-CH_3_ and 6′′′-CH_3_). *δ*_C_ (126 MHz; (CD_3_)_2_SO) 169.31 (C-2′′′), 166.96 (C-4′′′ and C-6′′′), 166.64 (3′′-NHCO), 160.76 (4-CONH), 155.75 (C-4′), 139.25 (C-3′′), 138.89 (C-3), 137.08 (C-1′′), 133.30 (C-1′), 132.85 (C-5), 128.96 (C-3′ or C-5′), 128.63 (C-6′), 122.59 (C-6′′), 121.03 (C-5′′), 118.73 (C-4′′), 118.17 (C-2′′), 117.82 (C-3′ or C-5′), 117.47 (C-4), 116.77 (CN), 116.06 (C-5′′′), 110.36 (C-2′), 69.80 (OCH_2_), 38.87 (NCH_3_), 35.48 (SCH_2_), 23.32 (4′′′-CH_3_ and 6′′′-CH_3_); HRMS (ESI): calcd.: 528.1812; found: {*m*/*z*: [M + H]^+^ 528.1814}; purity (HPLC): 210 nm: >95%; 254 nm: >95% (method 1a).

#### 8-((2-Aminothiazol-5-yl)methyl)-2-naphthonitrile (39)

To a stirred solution of bromo derivative 38 (500 mg, 1.57 mmol) in dry DMF (5 mL) were added zinc cyanide (110 mg, 0.940 mmol) and Pd(PPh_3_)_4_ (181 mg, 0.157 mmol). The reaction mixture was stirred at 80 °C for 18 h under N_2_ atmosphere, then was diluted with water (50 mL) and extracted with EtOAc (3 × 100 mL). The combined organic layers were dried over sodium sulfate, the solvent was evaporated *in vacuo* and the crude product was purified by FCC (hexanes/EtOAc/NEt_3_ 30 : 70 : 1) to give 39 (254 mg, 0.957 mmol, 61%) as a light-yellow solid; m.p. 216 °C; IR (ATR) *ν̃*/cm^−1^ 3397, 3283, 3116, 2221, 1629, 1517, 1438, 1377, 1326, 1310, 1270, 1204, 1160, 1120, 1052, 889, 861, 835, 794, 749, 721, 688; *δ*_H_ (500 MHz; (CD_3_)_2_SO) 8.75 (d, *J* = 1.6 Hz 1H, 8-H), 8.13 (d, *J* = 8.4 Hz, 1H, 5-H), 7.95 (d, *J* = 8.2 Hz, 1H, 4-H), 7.80 (dd, *J* = 8.5, 1.5 Hz, 1H, 6-H), 7.67 (dd, *J* = 8.2, 7.0 Hz, 1H, 3-H), 7.57 (dd, *J* = 7.2, 1.2 Hz, 1H, 2-H), 6.77 (s, 1H, 4′-H), 6.69 (s, 2H, NH_2_), 4.46 (s, 2H, CH_2_). *δ*_C_ (126 MHz; (CD_3_)_2_SO) 167.80 (C-2′), 137.86 (C-1), 135.88 (C-4′), 134.94 (C-4a), 130.64 (C-8), 130.14 (C-8a), 130.13 (C-5), 129.10 (C-3), 127.99 (C-2), 127.25 (C-4), 126.23 (C-6), 124.03 (C-5′), 119.37 (CN), 108.50 (C-7), 29.46 (CH_2_); HRMS (ESI): calcd.: 266.0746; found: {*m*/*z*: [M + H]^+^ 266.0745}.

#### 2-(8-((2-Aminothiazol-5-yl)methyl)naphthalen-2-yl)acetonitrile (40)

To a stirred solution of bromoarene 38 (323 mg, 1.01 mmol) in DMF (1.21 mL) was added 4-(4,4,5,5-tetramethyl-1,3,2-dioxaborolan-2-yl)isoxazole (237 mg, 1.22 mmol) and 1 M KF solution (513 μL, 513 μmol). The mixture was degassed in an ultrasonic bath under nitrogen atmosphere. PdCl_2_(dppf)·DCM (12.5 mg, 0.0171 mmol) was then added under nitrogen counterflow and the reaction mixture was stirred at 90 °C for 20 h. The mixture was allowed to cool to room temperature, diluted with water (50 mL) and brine (50 mL), and extracted with EtOAc (3 × 50 mL). The combined organic layers were dried over sodium sulfate. After evaporating the solvent *in vacuo*, the crude product was purified by FCC (hexanes/EtOAc 50 : 50) to give cyanomethyl derivative 40 (74.4 mg, 0.266 mmol, 26%) as a light brown solid; m.p. 203 °C; IR (ATR) *ν̃*/cm^−1^ 3463, 3112, 1613, 1519, 1403, 1354, 1301, 1195, 1035, 930, 844, 825, 794, 770, 754, 673; *δ*_H_ (400 MHz; (CD_3_)_2_SO) 8.14 (d, *J* = 1.6 Hz, 1H, 8-H), 7.98 (d, *J* = 8.4 Hz, 1H, 5-H), 7.84 (dd, *J* = 8.1,1.6 Hz 1H, 4-H), 7.52–7.41 (m, 3H, 2-H, 3-H, 6-H), 6.77 (s, 1H, 4′-H), 6.66 (s, 2H, NH_2_), 4.37 (s, 2H, CH_2_), 4.23 (s, 2H, CH_2_CN). *δ*_C_ (101 MHz; (CD_3_)_2_SO) 167.69 (C-2′), 136.37 (C-1), 135.67 (C-4′), 132.58 (C-4a), 131.01 (C-8a), 129.46 (C-5), 128.98 (C-7), 126.95 (C-4), 126.89 (C-2), 125.99 (C-3), 135.87 (C-6), 124.19 (C-5′), 122.85 (C-8), 119.21 (CN), 29.74 (CH_2_), 22.89 (CH_2_CN); HRMS (ESI): calcd.: 280.0903; found: {*m*/*z*: [M + H]^+^ 280.0902}.

#### *tert*-Butyl (5-((7-bromonaphthalen-1-yl)methyl)thiazol-2-yl)carbamate (41)

To a stirred solution of aminothiazole 38 (0.738 g, 2.31 mmol) in toluene (75 mL) was added di-*tert*-butyl dicarbonate (1.98 mL, 9.24 mmol) dropwise at room temperature. The reaction mixture was stirred at 100 °C for 4.5 h and then the solvent was evaporated *in vacuo*. The crude product was purified by FCC (hexanes/EtOAc 80 : 20) to give 41 (828 mg, 1.98 mmol, 85%) as a beige solid; m.p. 296 °C; IR (ATR) *ν̃*/cm^−1^ 2726, 1710, 1579, 1546, 1495, 1447, 1391, 1368, 1321, 1297, 1251, 1236, 1080, 1056, 1028, 880, 860, 844, 825, 809, 763, 750, 683; *δ*_H_ (400 MHz; (CD_3_)_2_SO) 11.25 (s, 1H, NHCO), 8.32 (d, *J* = 2.0 Hz, 1H, 8-H), 7.93 (d, *J* = 8.8 Hz, 1H, 5-H), 7.87 (m, 1H, 4-H), 7.65 (dd, *J* = 8.8, 1.9 Hz, 1H, 6-H), 7.56–7.48 (m, 2H, 2-H, 3-H), 7.19 (s, 1H, 4′-H), 4.52 (s, 2H, CH_2_), 1.41 (s, 9H, C(CH_3_)_3_). *δ*_C_ (126 MHz; (CD_3_)_2_SO) 158.38 (C-2′), 152.70 (NHCO), 135.65 (C-1), 134.90 (C-4′), 132.28 (C-8a), 132.08 (C-4a), 130.91 (C-5), 130.39 (C-5′), 128.88 (C-6), 127.75 (C-2), 127.37 (C-4), 126.40 (C-3), 126.02 (C-8), 119.78 (C-7), 80.88 (C̲(CH_3_)_3_), 29.29 (CH_2_), 27.84 (C(CH_3_)_3_); HRMS (ESI): calcd.: 417.0278; found: {*m*/*z*: [M–H]^−^ 417.0285}.

#### *tert*-Butyl (5-((7-(4,4,5,5-tetramethyl-1,3,2-dioxaborolan-2-yl)naphthalen-1-yl)methyl)thiazol-2-yl)carbamate (42)

PdCl_2_(dppf)·DCM (281 mg, 0.383 mmol), bis(pinacolato)diboron (1.46 g, 5.75 mmol), bromoarene derivative 41 (804 mg, 1.92 mmol) and KOAc (753 mg, 7.67 mmol) were dissolved in degassed anhydrous 1,4-dioxane (15 mL) under N_2_ atmosphere and stirred at 80 °C for 1 h and then at room temperature for another 2 h. Subsequently the mixture was diluted with water (100 mL) and brine (100 mL) and extracted with EtOAc (3 × 100 mL). The combined organic layers were dried over sodium sulfate. After evaporating the solvent *in vacuo*, the crude product was purified by FCC (hexanes/EtOAc 100 : 0 → 100 : 20) to give boronic acid pinacol ester 42 (456 mg, 0.978 mmol, 51%) as a beige solid; m.p. 203 °C; IR (ATR) *ν̃*/cm^−1^ 2975, 1719, 1624, 1568, 1457, 1369, 1341, 1309, 1251, 1240, 1142, 1090, 1059, 1009, 982, 962, 836, 804, 768, 755, 690; *δ*_H_ (400 MHz; (CD_3_)_2_SO) 11.24 (s, 1H, NHCO), 8.49 (s, 1H, 8-H), 7.93 (d, *J* = 8.2 Hz, 1H, 5-H), 7.85 (d, *J* = 8.1 Hz, 1H, 4-H), 7.73 (dd, *J* = 8.2, 1.0 Hz, 1H, 6-H), 7.53 (dd, *J* = 8.2, 7.0 Hz, 1H, 3-H), 7.45 (d, *J* = 7.0 Hz 1H, 2-H), 7.13 (s, 1H, 4′-H), 4.55 (s, 2H, CH_2_), 1.41 (s, 9H, C(CH_3_)_3_), 1.33 (s, 12H, O_2_C_2_(CH_3_)_4_). *δ*_C_ (101 MHz; (CD_3_)_2_SO) 158.35 (C-2′), 152.69 (NHCO), 136.82 (C-1), 135.08 (C-4a), 134.86 (C-4), 131.27 (C-8), 130.34 (C-5′), 130.31 (C-8a), 130.00 (C-6), 127.95 (C-5), 127.23 (C-4), 126.76 (C-3), 126.70 (C-2), 125.41 (C-7), 83.83 (O_2_C̲_2_(CH_3_)_4_), 80.86 (C̲(CH_3_)_3_), 29.25 (CH_2_), 27.82 (C(CH_3_)_3_), 24.71 (O_2_C_2_(CH_3_)_4_); HRMS (ESI): calcd.: 465.2025; found: {*m*/*z*: [M–H]^−^ 465.2033}.

#### (8-((2-((*tert*-Butoxycarbonyl)amino)thiazol-5-yl)methyl)naphthalen-2-yl)boronic acid (43)

To a stirred solution of 42 (541 mg, 1.16 mmol) in THF/water 3 : 1 v/v (6 mL), was added sodium periodate (744 mg, 3.48 mmol) and the reaction mixture was stirred at room temperature for 1 h. Then, 1 M HCl (1.16 mL) was added and the mixture was stirred for another 3 h, then the mixture was extracted with EtOAc (3 × 50 mL). The combined organic layers were dried over sodium sulfate. After evaporating the solvent *in vacuo*, the crude product was purified by FCC (DCM/MeOH 100 : 2) to give boronic acid 43 (376 mg, 0.978 mmol, 84%) as a white solid; m.p. 194 °C (decomposition); IR (ATR) *ν̃*/cm^−1^ 2976, 1716, 1624, 1558, 1458, 1368, 1346, 1306, 1250, 1151, 1094, 1061, 1024, 837, 755, 697; *δ*_H_ (400 MHz; (CD_3_)_2_SO) 11.21 (s, 1H, NHCO), 8.68 (s, 1H, 8-H), 8.23 (s, 2H, B(OH)_2_), 7.88–7.86 (m, 2H, 5-H, 6-H), 7.80 (d, *J* = 8.1 Hz, 1H, 4-H), 7.48 (dd, *J* = 8.1, 7.0 Hz, 1H, 3-H), 7.42 (dd, *J* = 7.0, 1.3 Hz, 1H, 2-H), 7.19 (s, 1H, 4′-H), 4.54 (s, 2H, CH_2_), 1.41 (s, 9H, C(CH_3_)_3_). *δ*_C_ (126 MHz; (CD_3_)_2_SO) 158.33 (C-2′), 152.73 (NHCO), 136.95 (C-1), 134.82 (C-4), 134.57 (C-4a), 131.72 (C-7), 130.74 (C-6, C-8a), 130.55 (C-8), 130.46 (C-5′), 127.28 (C-5), 127.02 (C-4), 126.41 (C-3), 126.32 (C-2), 80.90 (C̲(CH_3_)_3_), 29.23 (CH_2_), 27.88 (C(CH_3_)_3_); HRMS (ESI): calcd.: 385.1388; found: {*m*/*z*: [M + H]^+^ 358.1388}.

#### (8-((2-Aminothiazol-5-yl)methyl)naphthalen-2-yl)boronic acid (44)

To a stirred solution of *N*-Boc-derivative 43 (407 mg, 1.06 mmol) in chloroform (10 mL) was added TFA (3.98 mL, 53.0 mmol) at room temperature. The reaction mixture was stirred at room temperature for 17 h. Subsequently brine (15 mL) was added, and the solution was basified with NaOH (2.33 g, 58.3 mmol). After collecting the organic phase, the aqueous phase was extracted with DCM/2-propanol (3 × 30 mL, 4 : 1 v/v) and then EtOAc/2-propanol (3 × 30 mL, 4 : 1). The combined organic layers were dried over sodium sulfate and the solvent was evaporated *in vacuo* to give 44 (302 mg, 1.06 mmol, quant.) as a light greyish-white solid; m.p. 281 °C (decomposition); IR (ATR) *ν̃*/cm^−1^ 2929, 1599, 1554, 1515, 1456, 1379, 1315, 1256, 1157, 1051, 834, 754, 699; *δ*_H_ (500 MHz; (CD_3_)_2_SO) 8.69 (d, *J* = 1.1 Hz, 1H, 8-H), 8.21 (s, 2H, B(OH)_2_), 7.90–7.82 (m, 2H, 5-H, 6-H), 7.77 (d, *J* = 8.1 Hz, 1H, 4-H), 7.46 (dd, *J* = 8.2, 7.0 Hz, 1H, 3-H), 7.37 (dd, *J* = 7.0, 1.2 Hz, 1H, 2-H), 6.74 (s, 1H, 4′-H), 6.64 (s, 2H, NH_2_), 4.40 (s, 2H, CH_2_). *δ*_C_ (126 MHz; (CD_3_)_2_SO) 167.70 (C-2′), 137.26 (C-1), 135.59 (C-4′), 134.46 (C-4a), 131.55 (C-7) 130.78 (C-8), 130.49 (C-8a), 130.44 (C-6), 127.16 (C-5), 126.74 (C-4), 126.30 (C-3), 125.94 (C-2), 124.49 (C-5′), 29.58 (CH_2_); HRMS (ESI): calcd.: 285.0864; found: {*m*/*z*: [M + H]^+^ 285.0863}.

#### *N*-(5-((7-Bromonaphthalen-1-yl)methyl)thiazol-2-yl)-2-((4,6-dimethylpyrimidin-2-yl)thio)acetamide (45)

To a stirred solution of aminothiazole 38 (627 mg, 1.96 mmol) in dry DMF (5 mL) were added DMAP (120 mg, 0.982 mmol), EDC⋯HCl (461 mg, 2.36 mmol) and a solution of 2-((4,6-dimethylpyrimidin-2-yl)thio)acetic acid (389 mg, 1.96 mmol) in dry DMF (1 mL) at room temperature. The reaction mixture was stirred at room temperature for 16 h. Water (50 mL) and brine (50 mL) were added, and the mixture was extracted with DCM (3 × 100 mL). The combined organic layers were dried over sodium sulfate, the solvent was evaporated *in vacuo* and the crude product was purified by FCC (DCM/EtOAc 90 : 10) to give 45 (509 mg, 1.02 mmol, 52%) as a white solid. Analytical data are in alignment with literature.^20^

#### *N*-(5-((7-Cyanonaphthalen-1-yl)methyl)thiazol-2-yl)-2-((4,6-dimethylpyrimidin-2-yl)thio)acetamide (46)

To a stirred solution of 2-((4,6-dimethylpyrimidin-2-yl)thio)acetic acid (194 mg, 0.976 mmol) in dry DMF (3 mL) were added DIPEA (429 μL, 2.44 mmol) and HATU (371 mg, 0.976 mmol) and the reaction mixture was stirred at room temperature for 1 h. Aminothiazole 39 (216 mg, 0.814 mmol) was added and the reaction mixture was stirred at room temperature for another 18 h. Then the mixture was diluted with water (150 mL) and extracted with EtOAc (4 × 100 mL). The combined organic layers were dried over sodium sulfate. After evaporating the solvent *in vacuo*, the crude product was purified by FCC (hexanes/EtOAc 40 : 60) to give amide 46 (105 mg, 0.235 mmol, 29%) as a white solid; m.p. 218 °C; IR (ATR) *ν̃*/cm^−1^ 2897, 2221, 1687, 1581, 1530, 1429, 1374, 1322, 1258, 1243, 1161, 966, 876, 841, 819, 755, 715; *δ*_H_ (500 MHz; (CD_3_)_2_SO) 12.20 (s, 1H, CONH), 8.75 (d, *J* = 1.6 Hz, 1H, 8-H), 8.13 (d, *J* = 8.4 Hz, 1H, 5-H), 7.97 (d, *J* = 8.2 Hz, 1H, 4-H), 7.80 (dd, *J* = 8.5, 1.5 Hz, 1H, 6-H), 7.68 (dd, *J* = 8.2, 7.0 Hz, 1H, 3-H), 7.61 (dd, *J* = 7.1, 1.3 Hz, 1H, 2-H), 7.33 (s, 1H, 4′-H), 6.92 (s, 1H, CH_2_), 4.64 (s, 2H, CH_2_S), 4.05 (s, 2H, CH_2_S), 2.25 (s, 6H, 4-CH_3_, 6-CH_3_). *δ*_C_ (126 MHz; (CD_3_)_2_SO) 168.86 (C-2′′), 166.96 (C-4′′, C-6′′), 166.85 (NHCO), 156.77 (C-2′), 137.45 (C-1), 135.07 (C-4′), 134.99 (C-4a), 130.74 (C-5′), 130.58 (C-8), 130.19 (C-5), 130.09 (C-8a), 129.12 (C-3), 128.31 (C-2), 127.49 (C-4), 126.32 (C-6), 119.32 (CN), 116.08 (C-5′′), 108.65 (C-7), 34.01 (CH_2_S), 28.95 (CH_2_), 23.19 (4-CH_3_, 6-CH_3_); HRMS (ESI): calcd.: 444.0958; found: {*m*/*z*: [M–H]^−^ 444.0956}; purity (HPLC): 210 nm: >95%; 254 nm: >95% (method 3c).

#### *N*-(5-((7-(Cyanomethyl)naphthalen-1-yl)methyl)thiazol-2-yl)-2-((4,6-dimethylpyrimidin-2-yl)thio)acetamide (47)

To a stirred solution of 2-((4,6-dimethylpyrimidin-2-yl)thio)acetic acid (73.4 mg, 0.370 mmol) in dry DMF (0.5 mL) were added DIPEA (130 μL, 0.741 mmol) and HATU (141 mg, 0.370 mmol) and the reaction mixture was stirred at room temperature for 1 h. Aminothiazole 40 (69.0 mg, 0.247 mmol) was added and the reaction mixture was stirred at room temperature for another 18 h. Then the mixture was diluted with water (50 mL) and extracted with EtOAc (4 × 50 mL). The combined organic layers were dried over sodium sulfate. After evaporating the solvent *in vacuo*, the crude product was purified by FCC (hexanes/EtOAc 50 : 50) to give amide 47 (45.5 mg, 0.0990 mmol, 40%) as a white solid; m.p. 179 °C; IR (ATR) *ν̃*/cm^−1^ 2925, 1687, 1580, 1549, 1524, 1505, 1434, 1403, 1340, 1280, 1260, 1229, 1177, 1155, 1121, 1035, 951, 932, 891, 845, 823, 792, 757, 717; *δ*_H_ (500 MHz; (CD_3_)_2_SO) 12.18 (s, 1H, NHCO), 8.13 (s, 1H, 8-H), 7.98 (d, *J* = 8.4 Hz, 1H, 5-H), 7.85 (dd, *J* = 6.5, 3.0 Hz, 1H, 4-H), 7.51–7.45 (m, 3H, 2-H, 3-H, 6-H), 7.31 (s, 1H, 4′-H), 6.92 (s, 1H, 5′′-H), 4.54 (s, 2H, CH_2_), 4.21 (s, 2H, CH_2_CN), 4.05 (s, 2H, CH_2_S), 2.26 (s, 6H, 4-CH_3_, 6-CH_3_). *δ*_C_ (126 MHz; (CD_3_)_2_SO) 168.85 (C-2′′), 166.98 (C-4′′, C-6′′), 166.77 (NHCO), 156.62 (C-2′), 135.98 (C-1), 134.87 (C-4′), 132.64 (C-4a), 130.97 (C-8a), 130.91 (C-5′), 129.52 (C-5), 129.15 (C-7), 127.20 (C-2, C-4), 126.03 (C-3), 125.95 (C-6), 122.86 (C-8), 119.18 (CN), 116.09 (C-5′′), 34.00 (CH_2_S), 29.28 (CH_2_), 23.21 (4-CH_3_, 6-CH_3_), 22.89 (CH_2_CN); HRMS (ESI): calcd.: 458.1115; found: {*m*/*z*: [M–H]^−^ 458.1113}; purity (HPLC): 210 nm: >95%; 254 nm: >95% (method 3c).

#### (8-((2-(2-((4,6-Dimethylpyrimidin-2-yl)thio)acetamido)thiazol-5-yl)methyl)naphthalen-2-yl)boronic acid (48)

To a stirred solution of amine 44 (304 mg, 1,07 mmol) in dry DMF (5 mL) were added DMAP (65.4 mg, 0.535 mmol), EDC⋯HCl (251 mg, 1.28 mmol) and a solution of 2-((4,6-dimethylpyrimidin-2-yl)thio)acetic acid (212 mg, 1.07 mmol) in dry DMF (1 mL) at room temperature. The reaction mixture was stirred at room temperature for 16 h. Water (100 mL) and brine (50 mL) were added, and the mixture was extracted with EtOAc (3 × 100 mL). The combined organic layers were dried over sodium sulfate, the solvent was evaporated *in vacuo*, and the crude product was purified by FCC (DCM/MeOH/NH_3_ (aq., 25%) 100 : 5 : 0.05) to give amide 48 (82.5 mg, 0.178 mmol, 17%) as a white solid; m.p. 153 °C (decomposition); IR (ATR) *ν̃*/cm^−1^ 2928, 1691, 1623, 1583, 1533, 1438, 1385, 1313, 1265, 1163, 1030, 972, 893, 837, 758, 668; *δ*_H_ (500 MHz; (CD_3_)_2_SO) 12.17 (s, 1H, NHCO), 8.68 (s, 1H, 8-H), 8.20 (s, 2H, B(OH)_2_), 7.88–7.84 (m, 2H, 5-H, 6-H), 7.79 (d, *J* = 8.1 Hz, 1H, 4-H), 7.47 (dd, *J* = 8.2, 7.0 Hz, 1H, 3-H), 7.41 (dd, *J* = 7.0, 1.3 Hz, 1H, 2-H), 7.27 (s, 1H, 4′-H), 6.92 (s, 1H, 5′′-H), 4.57 (s, 2H, CH_2_), 4.05 (s, 2H, CH_2_S), 2.26 (s, 6H, 4-CH_3_, 6-CH_3_). *δ*_C_ (126 MHz; (CD_3_)_2_SO) 168.87 (C-2′′), 166.99 (C-4′′, C-6′′), 166.76 (NHCO), 156.58 (C-2′), 136.80 (C-1), 134.80 (C-4′), 134.51 (C-4a), 131.73 (C-7), 131.21 (C-5′), 130.70 (C-8), 130.51 (C-6), 130.43 (C-8a), 127.23 (C-5), 127.00 (C-4), 126.33 (C-3), 126.27 (C-2), 116.11 (C-5′′), 34.01 (CH_2_S), 29.10 (CH_2_), 23.23 (4-CH_3_, 6-CH_3_); HRMS (ESI): calcd.: 465.1221; found: {*m*/*z*: [M + H]^+^ 465.1220}; purity (HPLC): 210 nm: >95%; 254 nm: >95% (method 3a).

#### 2-((4,6-Dimethylpyrimidin-2-yl)thio)-*N*-(5-((7-formylnaphthalen-1-yl)methyl)thiazol-2-yl)acetamide (49)

Bromoarene 45 (200 mg, 0.400 mmol), Pd(OAc)_2_ (2.70 mg, 0.0120 mmol, 3 mol%), dppf (9.99 mg, 0.0180 mmol, 4.5 mol%), *N*-formylsaccharin (134 mg, 0.601 mmol) and Na_2_CO_3_ (63.7 mg, 0.601 mmol) were added to a 30 mL glass tube, which was then evacuated and backfilled three times with N_2_. A degassed solution of Et_3_SiH (84.1 μL, 0.521 mmol) in DMF (2 mL) was added to the glass tube under N_2_ atmosphere. The mixture was stirred for 10 min at room temperature and subsequently warmed to 80 °C and stirred for another 16 h. After cooling to room temperature, the mixture was diluted with water (100 mL) and extracted with DCM (3 × 50 mL). After the combined organic layers were dried over sodium sulfate, the solvent was evaporated *in vacuo* and the crude product was purified using two consecutive PTLCs (hexanes/EtOAc/NEt_3_ 50 : 50 : 1 and DCM/EtOAc 70 : 30) to give 49 (18.8 mg, 0.0419 mmol, 11%) as a white solid; m.p. 219 °C; IR (ATR) *ν̃*/cm^−1^ 2961, 1921, 2852, 1691, 1580, 1533, 1434, 1368, 1341, 1297, 1259, 1231, 1168, 1187, 1168, 1134, 1014, 972, 886, 860, 794, 762, 751, 711; *δ*_H_ (500 MHz; CDCl_3_) 11.43 (bs, 1H, NHCO), 10.02 (s, 1H, CHO), 8.45 (s, 1H, 8-H), 7.90 (d, *J* = 8.5 Hz, 1H, 5-H), 7.84 (dd, *J* = 8.5, 1.5 Hz, 1H, 6-H), 7.78 (d, *J* = 8.2 Hz, 1H, 4-H), 7.52 (dd, *J* = 8.3, 7.0 Hz, 1H, 3-H), 7.44 (d, *J* = 7.1 Hz, 1H, 2-H), 7.01 (s, 1H, 4′-H), 6.76 (s, 1H, 5′′-H), 4.53 (s, 2H, CH_2_), 3.76 (s, 2H, CH_2_S), 2.40 (s, 6H, 4-CH_3_, 6-CH_3_). *δ*_C_ (126 MHz; CDCl_3_) 192.53 (CHO), 170.05 (C-2′′), 168.49 (C-4′′, C-6′′), 167.65 (NHCO), 157.19 (C-2′), 137.92 (C-1), 137.47 (C-4a), 135.61 (C-4′), 134.64 (C-7), 131.92 (C-5′), 131.41 (C-8a), 130.54 (C-8), 130.35 (C-5), 129.29 (C-3), 128.39 (C-4), 128.17 (C-2), 123.02 (C-6), 117.24 (C-5′′), 34.71 (CH_2_S), 30.74 (CH_2_), 23.94 (4-CH_3_, 6-CH_3_); HRMS (ESI): calcd.: 417.0920; found: {*m*/*z*: [M + Na]^+^ 417.0919}; purity (HPLC): 210 nm: >95%; 254 nm: >95% (method 3b).

### Biology

#### Sirtuin assays

The determination of inhibitory activity on the corresponding sirtuin enzymes was carried out based on a fluorescence-based assay commissioned by Reaction Biology Corporation (Malvern, USA) following an internal experimental protocol. Test substances were prepared in DMSO, delivered into the respective enzyme mixture (prepared with reaction buffer: Tris-HCl, pH = 8) and incubated for 10 minutes at 30 °C. Subsequently the substrate mixture (NAD^+^ and a 7-amino-4-methylcoumarin-based fluorogenic peptide substrate) was added to initiate the deacetylation reaction. After 2 hours incubation at 30 °C, 2 mM nicotinamide (universal sirtuin inhibitor, to stop the reaction) and protease-based developer (to generate fluorescence by the cleavage of 7-amino-4-methylcoumarin) was added. At 30 °C 1 hour later, the respective fluorescence was measured (extinction/emission = 360 nm/460 nm). The inhibitory effect of the test compounds is indirectly proportional to the amount of converted fluorescent substrate standardized as 100% activity of the control without inhibitor. The test substances were tested in 10-dose IC_50_ triplicate mode, with 3-fold serial dilution starting at 50 μM final reaction concentration and if necessary, starting at 100 μM. For each serially diluted replicate of the triplicate, an IC_50_ value was determined by sigmoidal curve fitting, resulting in three IC_50_ values, from which the mean and corresponding standard deviation were subsequently calculated. Data processing was performed based on Prism 8.0.2 software (GraphPad Software, Boston, USA). Subtype selectivity on SIRT1, SIRT3 and SIRT5 was evaluated in a single dose duplicate mode at 50 μM final test compound concentration according to previous outlined internal protocol of Reaction Biology Corporation by determining the enzyme activity of the respective sirtuins in % (no inhibitor control as 100% activity).

#### Fluorescence thermal shift assays

Fluorescence thermal shift assays were performed in white 96-well plates (Hard-Shell PCR Plates, BioRad, USA) with a total volume of 20 μL per well and a final DMSO concentration of 5% (v/v), following a published protocol.^19,21^ Briefly, 10 μL SIRT2 (final concentration of 6.0 μM, purified according to published protocol^21^) and SYPRO Orange (5× final concentration, Sigma-Aldrich, Germany) in assay buffer (25 mM Tris-HCl, 150 mM NaCl, 1 mM DTT, pH 8.0) were mixed with 10 μL of compound (final concentration of 30 μM and 10 μM) and incubated at 25 °C and 350 rpm for 5 min. The compounds were prepared as 10 mM stock solutions in DMSO and diluted in the assay such that a final DMSO concentration of 5% (v/v) was achieved. If necessary, NAD^+^ was added to a final concentration of 2.5 mM. Fluorescence intensity was recorded during a temperature gradient of 1 °C per 20 s from 25 to 95 °C using a real-time PCR machine (C1000 Touch™ Thermal Cycler, CFX96™ Real-Time System, BioRad, USA). Melting temperatures were determined using GraphPad Prism, following a published procedure.^52^

#### Cloning and expression of SIRT2 56-356

The human *SIRT2* 2-389 gene (Table S4) was synthesized and cloned in a pETDuet based vector encoding an N-terminal His_6_-SUMO tag by Eurofins Genomics. This construct served for Q5 mutagenesis with primers A-D (Table S5) to yield His_6_-SUMO-Ser-SIRT2 56-356. All plasmids were controlled by sequencing. *SIRT2* 56-356 was expressed as an N-terminal His_6_-SUMO fusion in Rosetta(DE3) cells. Overnight cultures were grown in LB with ampicillin (180 mg L^−1^) and chloramphenicol (50 mg L^−1^) at 37 °C, 130 rpm. Expression cultures were inoculated with 25 mL overnight culture per liter autoinduction medium from Studier^53^ (adapted according to Table 2) with the antibiotics ampicillin (90 mg L^−1^) and chloramphenicol (25 mg L^−1^) added. After 18 h at 37 °C, 130 rpm, the cells were cultured for 24 h at 16 °C, 130 rpm for protein expression. Cells were harvested by centrifugation for 20 min at 5000 × *g*, 20 °C, and washed with 0.9% (w/v) NaCl. The supernatant was removed by centrifugation for 20 min at 5000 × *g*, 4 °C, and the cell pellets were stored at −20 °C.

**Table 2:** Composition of autoinduction medium

Content [g L^−1^]		Compound
5.0	g L^−1^	Yeast extract
10	g L^−1^	Peptone
0.5	g L^−1^	Glucose
1.9	g L^−1^	Lactose
5.0	g L^−1^	Glycerol
0.2	g L^−1^	Magnesium sulfate
3.4	g L^−1^	Potassium dihydrogen phosphate
3.5	g L^−1^	Disodium hydrogen phosphate
2.8	g L^−1^	Ammonium chloride
pH adjusted to		pH 6.8

#### Protein purification of SIRT2 56-356

Cell pellets were resuspended on ice in 100 mM HEPES pH 7.0, 300 mM NaCl, 20 mM imidazole, 2 mM β-mercaptoethanol, 1 mg of DNAse I, and Pefabloc® SC. After lysis by sonication, the cell debris was removed by centrifugation for 20 min at 41 000 × *g*, 4 °C. The lysate was loaded onto a NiSepharose column (HisTrap™ HP 5 mL, Cytiva) previously equilibrated with buffer A (100 mM HEPES pH 7.0, 300 mM NaCl, 20 mM imidazole, 2 mM β-mercaptoethanol) by an Äkta pure™ System (GE Healthcare) at 4 °C. The column was washed with 10 CV of buffer A, and SIRT2 was eluted by a continuous gradient from 0 to 100% buffer B (100 mM HEPES pH 7.0, 300 mM NaCl, 500 mM imidazole, 2 mM β-mercaptoethanol). All purification steps were monitored by UV absorption at 280 nm and SDS-PAGE. To remove the His_6_-SUMO-tag, the fractions containing SIRT2 were dialyzed overnight at 4 °C with His_6_-SUMO-protease against 2 L of 50 mM HEPES pH 7.0, 150 mM NaCl, 2 mM β-mercaptoethanol. Precipitated protein was removed by centrifugation, and the supernatant was subjected to a second Ni^2+^ affinity chromatography. The flow-through containing cleaved SIRT2 was collected and concentrated to a volume of 2 mL using an Amicon® centrifugal filter (Merck) with 10 000 MWCO. The protein solution was loaded on a HiLoad® 16/600 Superdex® 200 pg column (Cytiva) at 4 °C using 25 mM HEPES pH 7.0, 100 mM NaCl, 2 mM DTT as running buffer. Fractions containing SIRT2 were pooled and concentrated using Amicon® 10 000 MWCO centrifugal filters (Merck). The protein was frozen in liquid nitrogen and stored at −80 °C until further use.

#### Crystallisation and X-ray structure determination of SIRT2 56-356

SIRT2 56-356 (23.7 mg mL^−1^) was crystallized without ligand by sitting drop vapor diffusion at 20 °C. A 2 : 1 ratio of protein and reservoir solution containing 0.2 M ammonium formate, 20% (w/v) PEG3350 led to crystals within two months. Crystals were cryoprotected with 35% (v/v) glycerol and flash frozen in liquid nitrogen.

Crystal structures of SIRT2 56-356 with ligands were obtained from hanging drop crystallization experiments at 20 °C. The SIRT2 56-356 protein was diluted to 7.3 mg mL^−1^ in 25 mM HEPES pH 7.0, 100 mM NaCl, 2 mM DTT, and mixed with 5 mM NAD^+^ and 500 μM of 29 (RW-78)/31 (RW-80) dissolved in DMSO, respectively. The solution was incubated for 1 h on ice, and any precipitate was removed by centrifugation for 10 min at 16 000 × *g*. Crystals grew after 5 days in 24-well, with 300 μL of 0.1 M sodium acetate, 17% (w/v) PEG3350 as reservoir, and a drop ratio of 1 μL protein to 1 μL reservoir solution. The crystals were soaked with 10 μL of 30% (v/v) ethylene glycol and vitrified in liquid nitrogen. Diffraction images of SIRT2 56-356:RW-78, and SIRT2 56-356:RW-80 were recorded using synchrotron radiation of *λ* = 1.060 Å at the P13 beamline from PETRAIII at DESY (Deutsches Elektronen-Synchrotron, EMBL, Hamburg, Germany). Recorded reflections were processed using the XDS suite, and data reduction was performed with XSCALE.^54,55^ Phasing of the SIRT2 56-356 apo structure was performed by molecular replacement with PHASER^56^ and the coordinates of SIRT2:SirReal2 (PDB ID: 4RMG^19^). The solution was refined by restrained refinement with REFMAC5 (ref. 57) and iteratively rebuilt with COOT (v. 0.9).^58^ The resulting model was used to phase the SIRT2 56-356:RW-78 data set, followed by iterating model building in COOT^58^ with restrained refinements in REFMAC5.^57^ The ligand was prepared in AceDRG.^59^ Water molecules were positioned with ARP/wARP 8.0.^60^ The apo structure was completed by TLS refinement, whereas the SIRT2 56-356:RW-78 structure was refined with anisotropic restraints to satisfactory *R*_work_ and *R*_free_ values. The SIRT2 56-356:RW-80 structure was phased with the SIRT2:RW-78 structure in REFMAC5 (ref. 57) and built as described for SIRT2 56-356:RW-78. The geometry of the final structures was analyzed by the MOLPROBITY^61^ online tool, and the structures were deposited in the RCSB Protein Data Bank (Tables S1–S3).

#### NanoBRET assay

The NanoBRET target engagement assay was performed as previously described by Vogelmann *et al.*,^21^ with the modification that stably transfected HEK293T cells expressing the NanoLuc-tagged SIRT2(50-356) fusion protein were used instead of transient transfection. HEK293T-NLuc-Sirt250-356 cells were cultured under standard conditions (37 °C, 5% CO_2_) in DMEM supplemented with 10% fetal bovine serum, 2 mM glutamine, and 300 μg mL^−1^ hygromycin as a selection marker. For the assay, cells were trypsinized, resuspended in Opti-MEM® reduced serum medium, and adjusted to a concentration of 2 × 10^5^ cells per mL. To determine the affinities of the inhibitors, a final tracer concentration of 2 μM was used. Serially diluted inhibitor and tracer were added to the cell suspension, and 100 μL were seeded into 96-well white, sterile, nonbinding surface plates. Plates were incubated at 37 °C with 5% CO_2_ for 2 hours. For BRET measurements, NanoBRET NanoGlo Substrate (Promega cat. #N1571) was added to the wells according to the manufacturer's protocol. All measurements were performed using the 2102 EnVision™ Multilabel Reader (PerkinElmer), equipped with a 460 nm filter (donor) and a 615 nm filter (acceptor). The BRET ratio was calculated as the ratio of acceptor to donor signal, and data were normalized to vehicle-treated controls. Apparent intracellular binding affinities (EC_50_ values) were determined by fitting dose–response curves using nonlinear regression analysis in GraphPad Prism. All experiments were conducted in triplicate unless otherwise noted, and results are presented as mean ± standard deviation.

### Computational methods

#### Docking simulations

Docking simulations were carried out with Schrödinger software suite (Schrödinger Inc., New York City, USA, version 2020-3).^62^ Crystal structures of SIRT2 and respective lead structures were imported from the Protein Data Bank (PDB)^63^ (SirReal2: PDB ID: 4RMG;^19^24a: PDB ID: 5YQO^23^) and prepared with the Protein Preparation Wizard (Schrödinger Inc. New York City, USA). All ligands were prepared with the Ligand Preparation Wizard using Epik for protonation and charge calculations.^64^ Docking was performed using Glide in standard precision mode SP (all docking parameters left to their default values). Results were inspected and visualized with PyMOL 2.5.8 (Schrödinger Inc., New York city, USA). The top-ranking poses were analysed, considering the favourable spatial orientation in relation to the crystal structures of the corresponding lead compounds.

## Author contributions

This study was conceptualised and supervised by F. B. Investigation, methodology, experimental execution and data curation were performed by R. W., M. F., A. H., N. P., F. F., T. W., M. J., M. G. and E. M. H. Original manuscript draft was written and edited by R. W. and M. F. with inputs from A. H., E. M. H. and F. B. All authors partook in the discussion of the findings and thoroughly reviewed the final manuscript for publication.

## Conflicts of interest

There are no conflicts to declare.

## Supplementary Material

MD-OLF-D5MD00144G-s001

## Data Availability

Supplementary information is available: 1H and ^13^C NMR spectra of compounds, HPLC chromatograms of tested compounds, Tables S1–S5: Crystallographic data, Fig. S1: Purification of human SIRT2 56-356 and Fig. S2: Close-up view of the SIRT2 ligand binding site can be found in the SI. See DOI: https://doi.org/10.1039/D5MD00144G. The data supporting this article have been included as part of the SI. X-ray coordinates have been deposited in the RCSB Protein Data Bank under the accession codes 9S44 (SIRT2 56-356), 9S46 (SIRT2 56-356:RW-78) and 9S48 (SIRT2 56-356:RW-80).
