# Supplementary material for: Tailored SirReal-type inhibitors enhance SIRT2 inhibition through ligand stabilization and disruption of NAD+ co-factor binding
Source: RSC Med Chem. 2025 Aug 19;16(11):5419–40. doi: 10.1039/d5md00144g (PMC12412615; doi:10.1039/d5md00144g)
Supplement: MD-016-D5MD00144G-s001 [file MD-016-D5MD00144G-s001.pdf]

Supplementary Information (SI) for RSC Medicinal Chemistry.
This journal is © The Royal Society of Chemistry 2025

Supplementary Information

Tailored SirReal-type inhibitors enhance SIRT2 inhibition through ligand stabilization and disruption of NAD^+ co-factor binding

Ricky Wirawan[†a] Matthias Frei[†a], Anna Heider[b], Niklas Papenkordt[c], Florian Friedrich[c], Thomas Wein[a], Manfred Jung[c], Michael Groll[b], Eva M. Huber[b] and Franz Bracher*[a]

[a]Department of Pharmacy, Ludwig-Maximilians University Munich, Butenandtstraße 5-13, 81377 Munich, Germany. *E-mail: franz.bracher@cup.uni-muenchen.de
[b]Center for Protein Assemblies, Technical University of Munich, Ernst-Otto-Fischer-Straße 8, 85748 Garching, Germany.
[c]Institute of Pharmaceutical Sciences, Albert-Ludwigs-Universität Freiburg, Albertstraße 25, 79104 Freiburg im Breisgau, Germany.

[†]Equal contribution as first author to this work.

Table of Contents:

^1H and ^{13}C NMR spectra of compounds 2

HPLC chromatograms of tested compounds 37

Table S1-S5: Crystallographic data 52

Figure S1: Purification of human SIRT2 56-356 56

Figure S2: Close-up view of the SIRT2 ligand binding site 57

^{1}H and ^{13}C NMR spectra of compound **3**

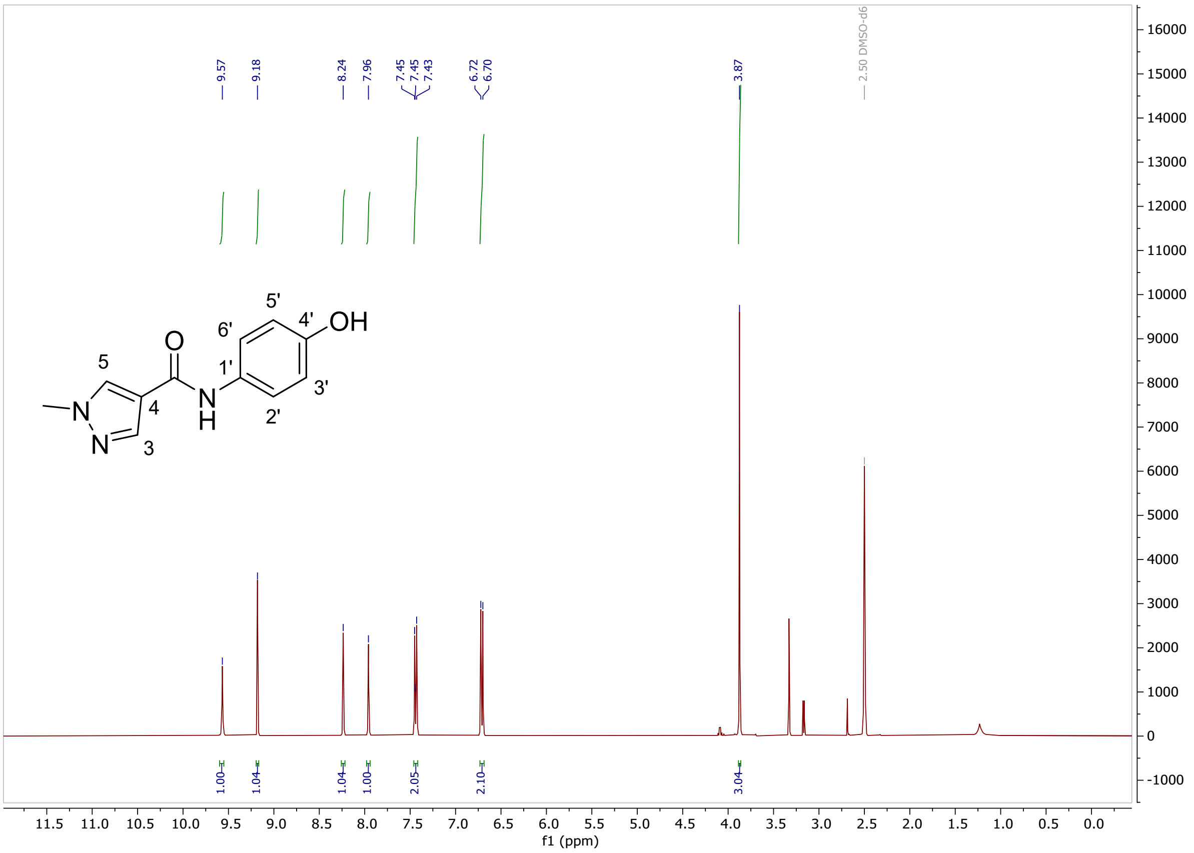

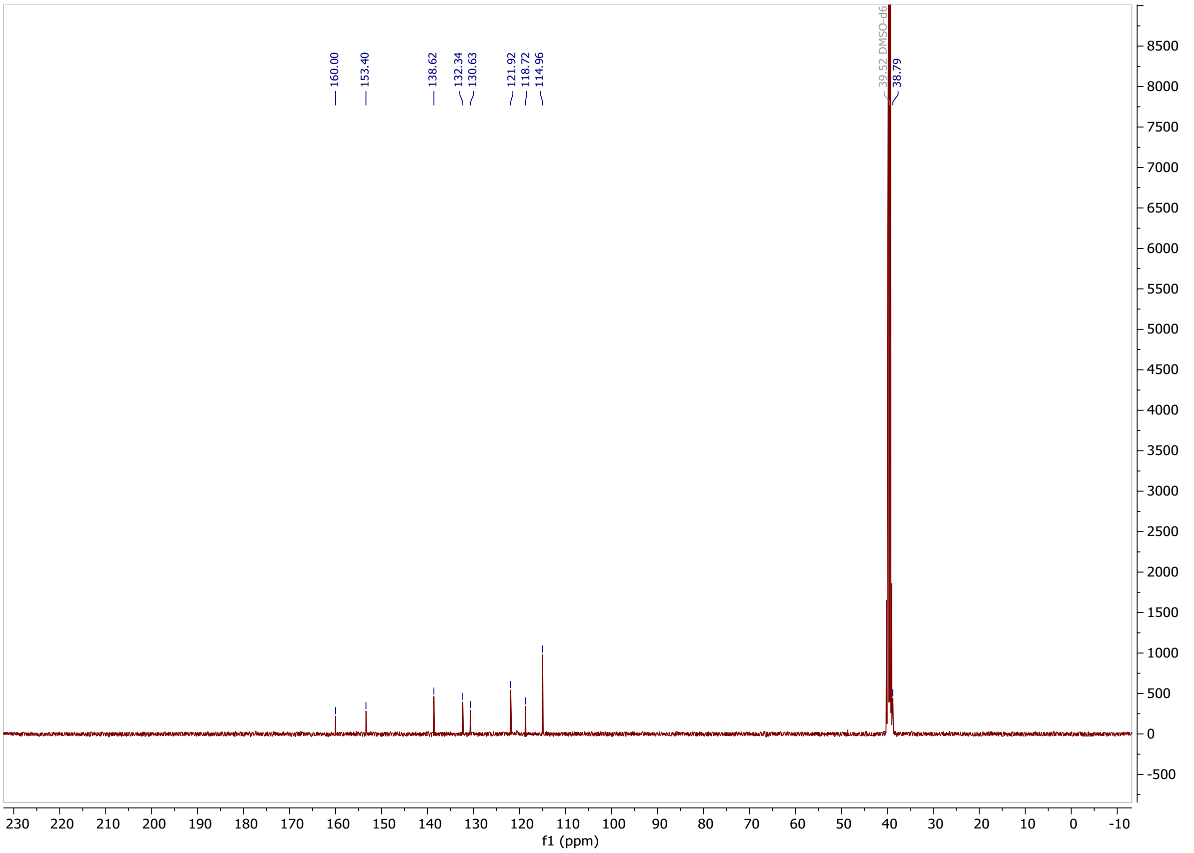

^{1}H and ^{13}C NMR spectra of compound **4**

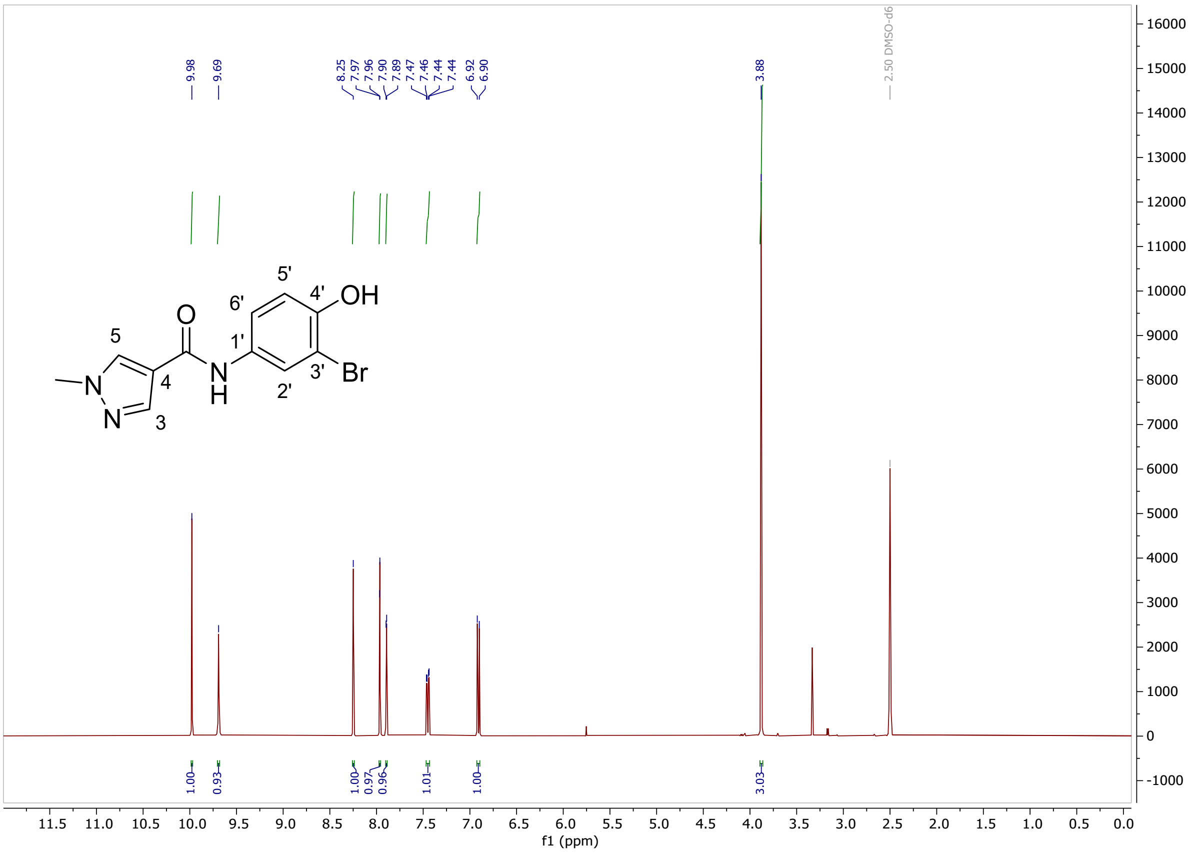

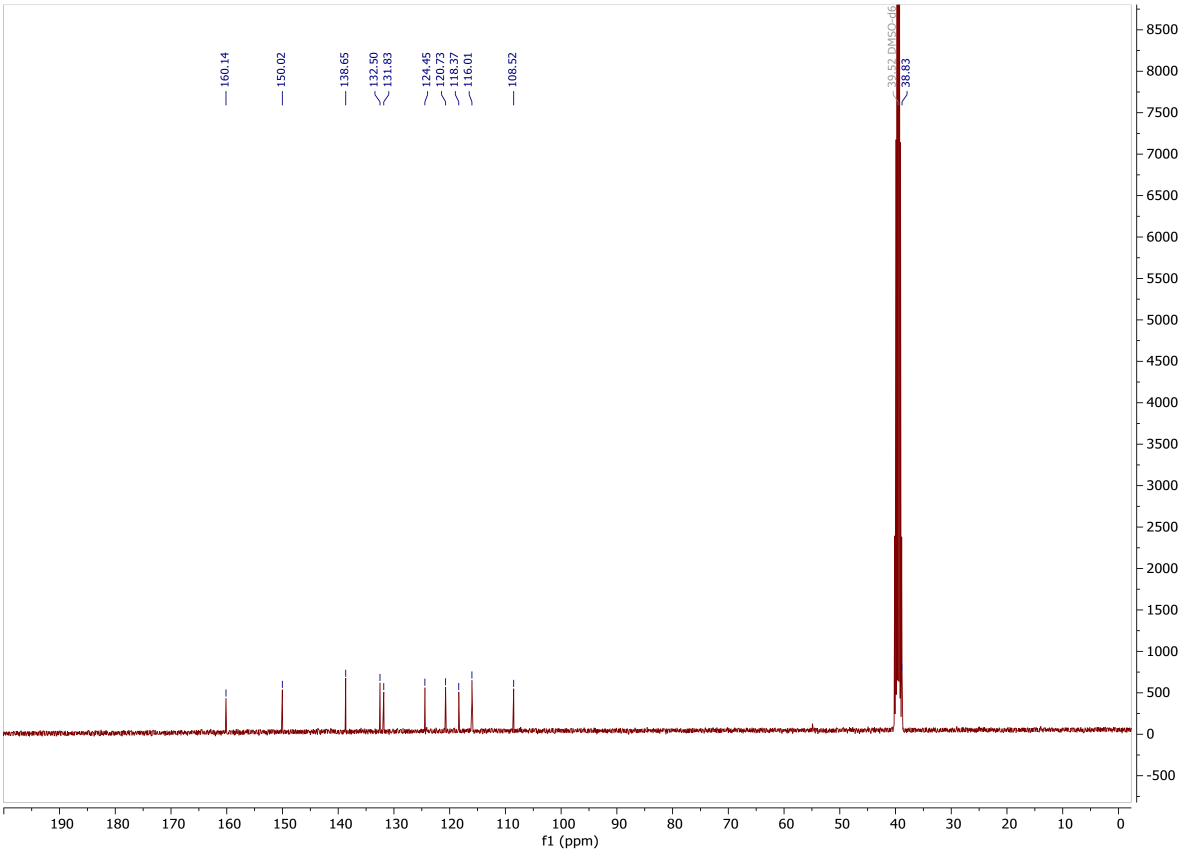

^{1}H and ^{13}C NMR spectra of compound **6**

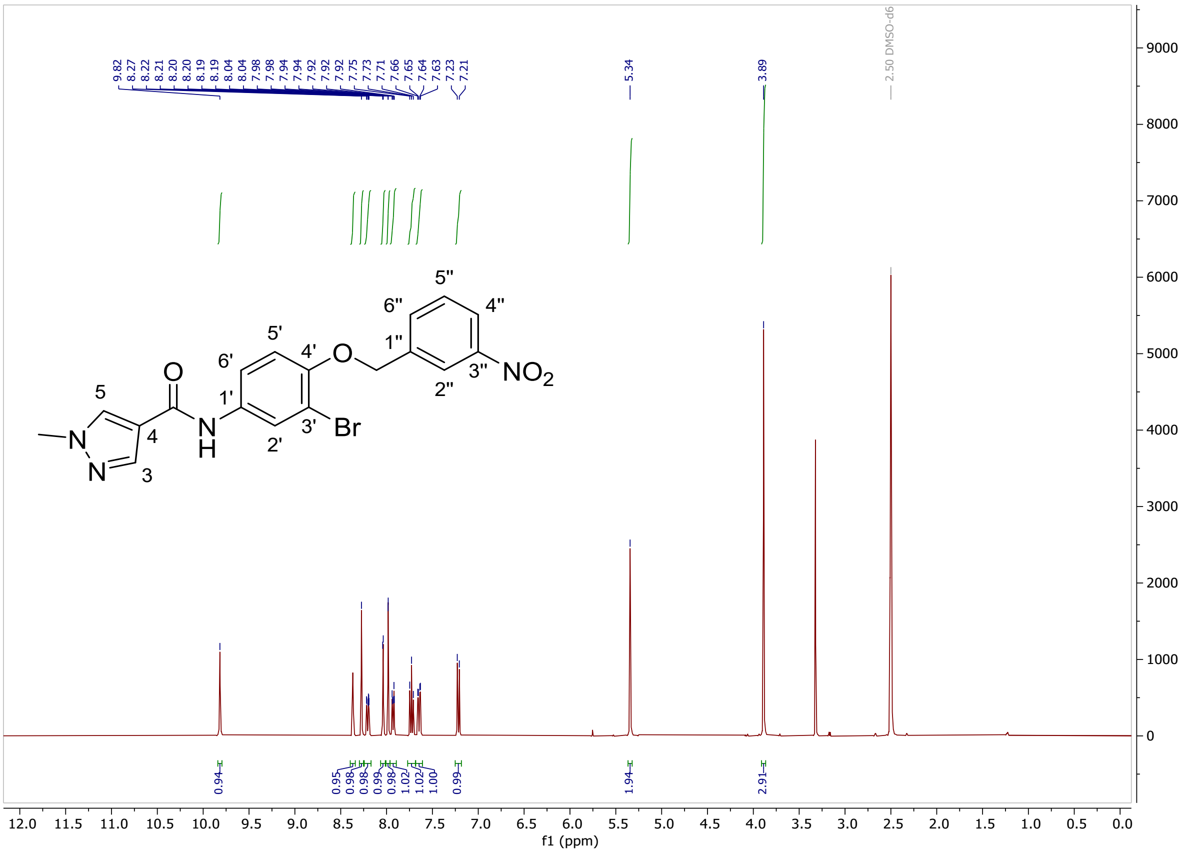

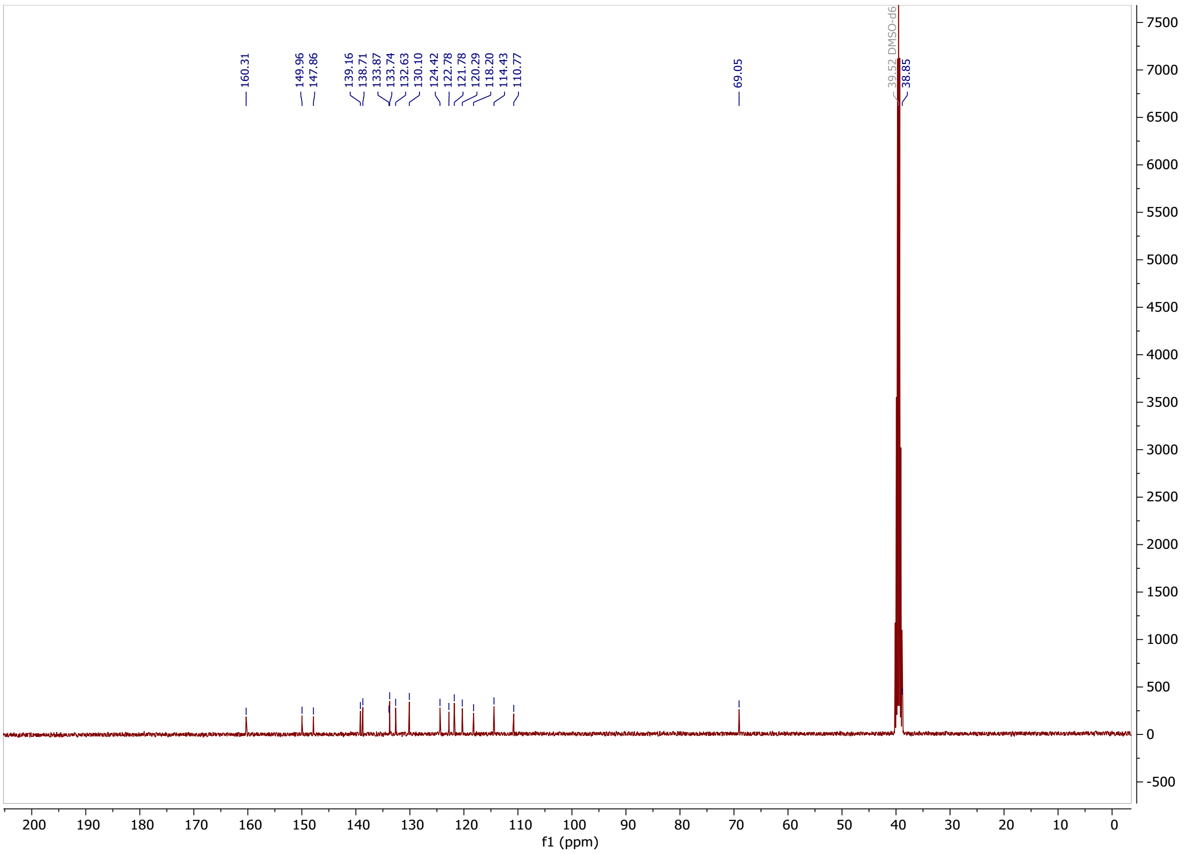

^{1}H and ^{13}C NMR spectra of compound **7**

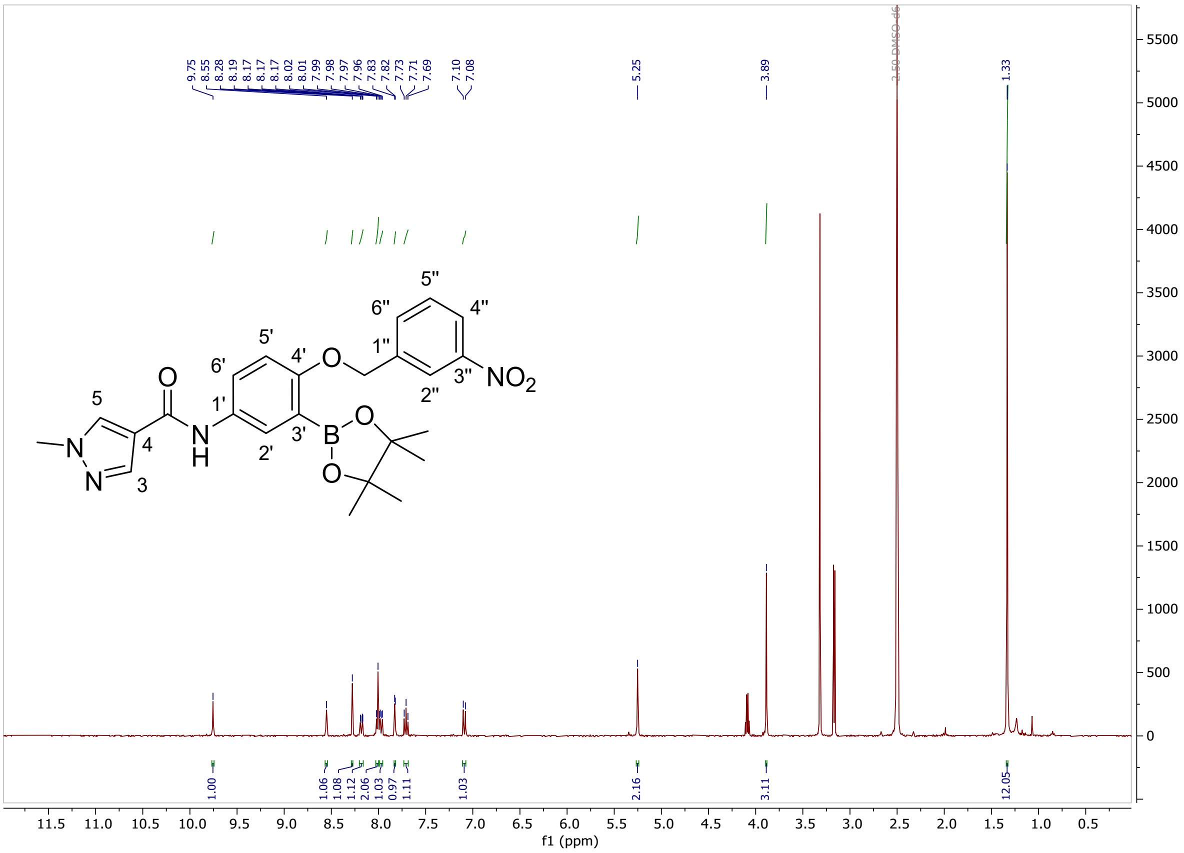

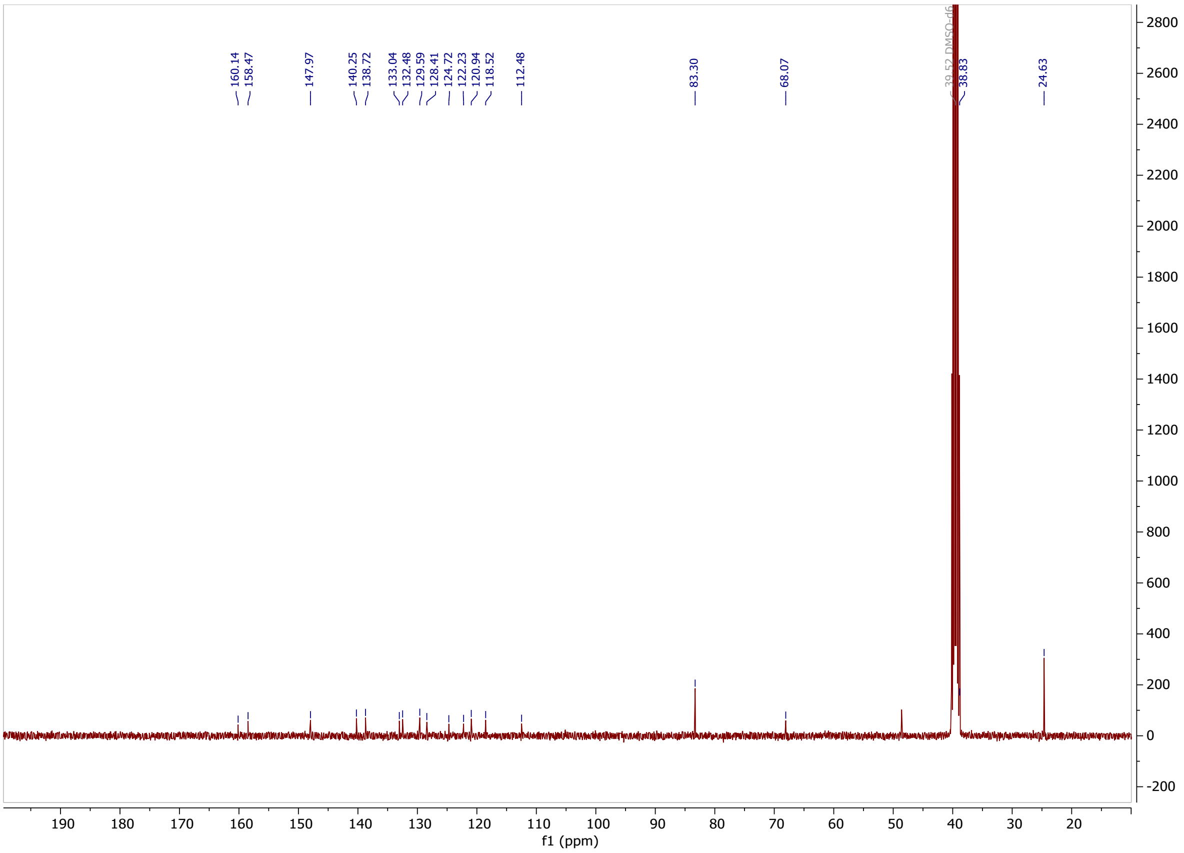

^{1}H and ^{13}C NMR spectra of compound **8**

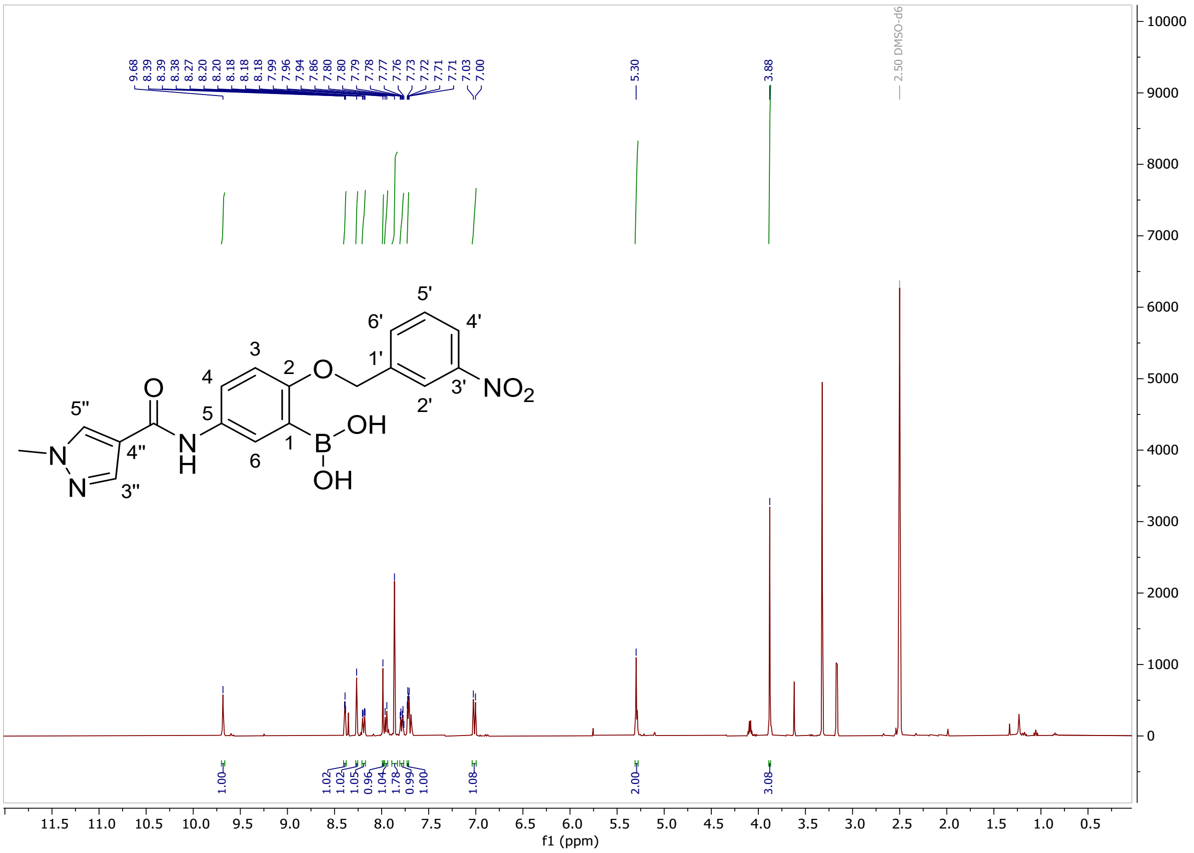

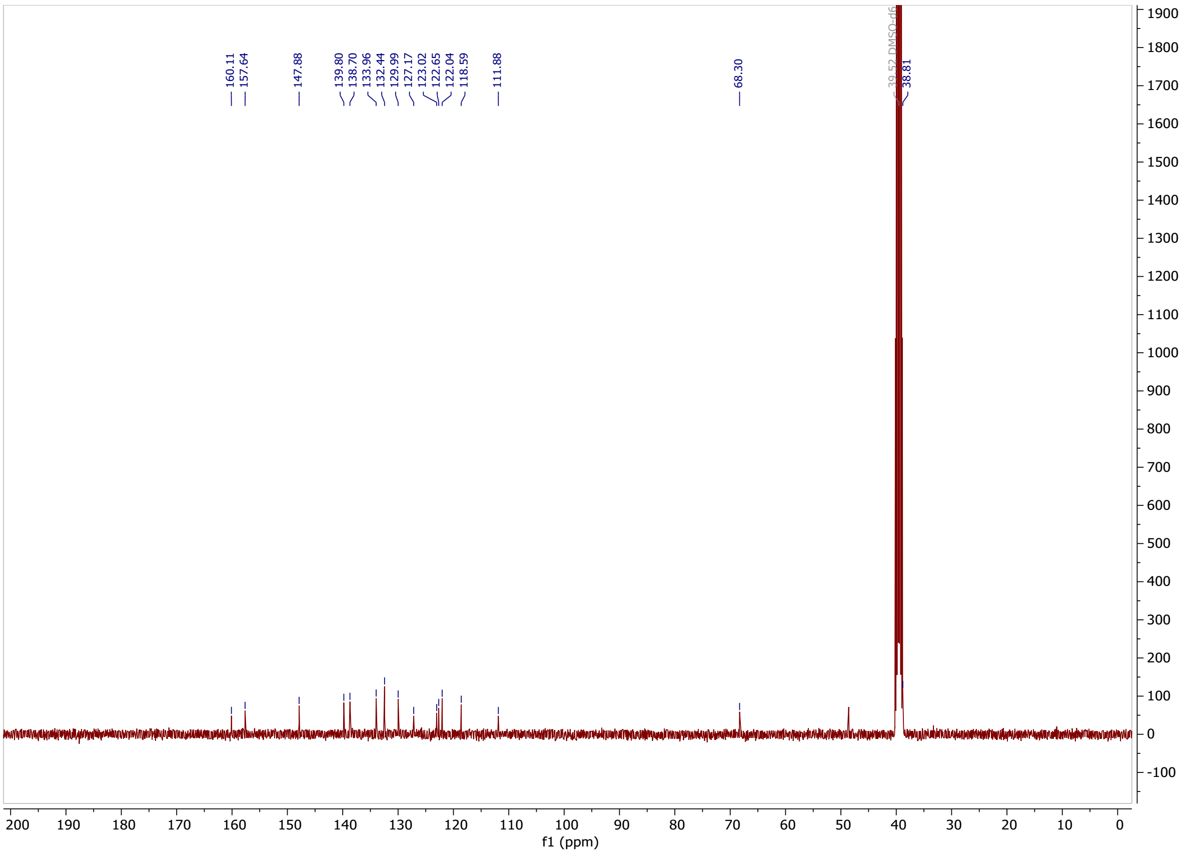

^{1}H and ^{13}C NMR spectra of compound **9**

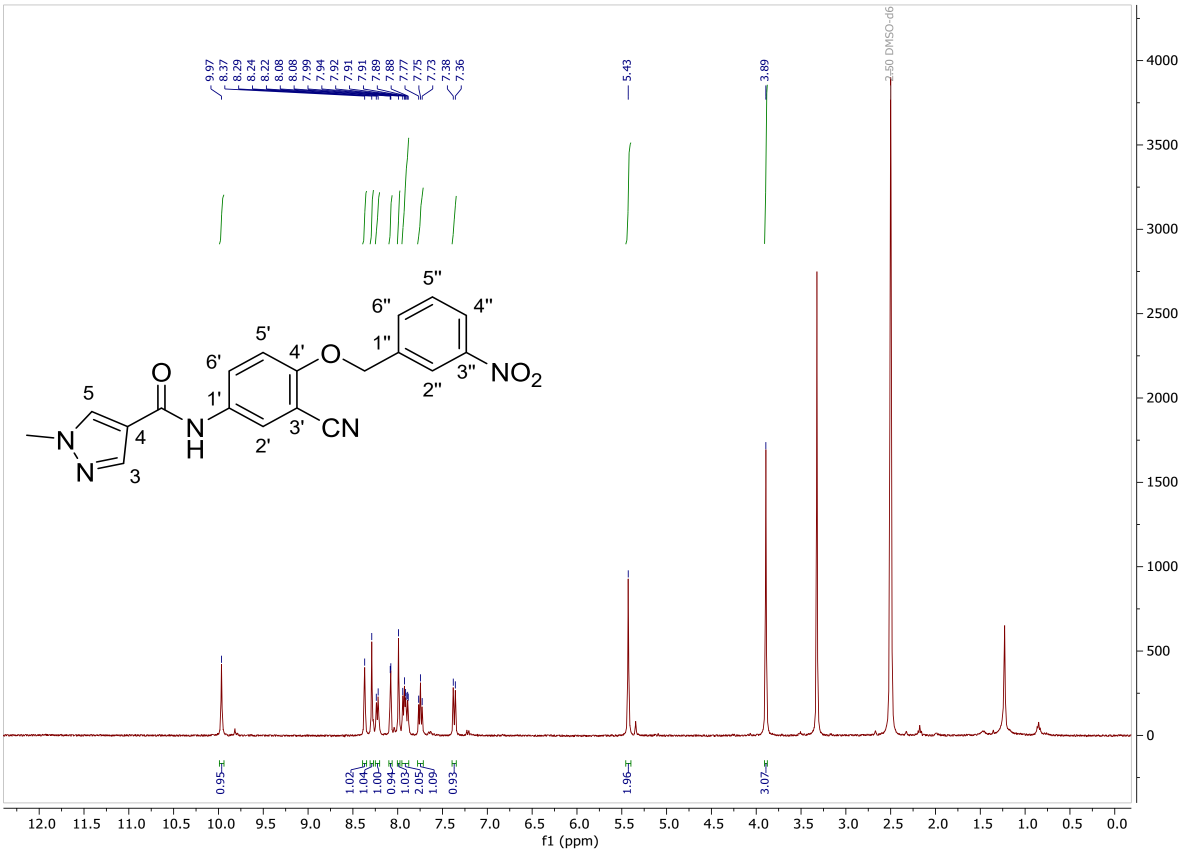

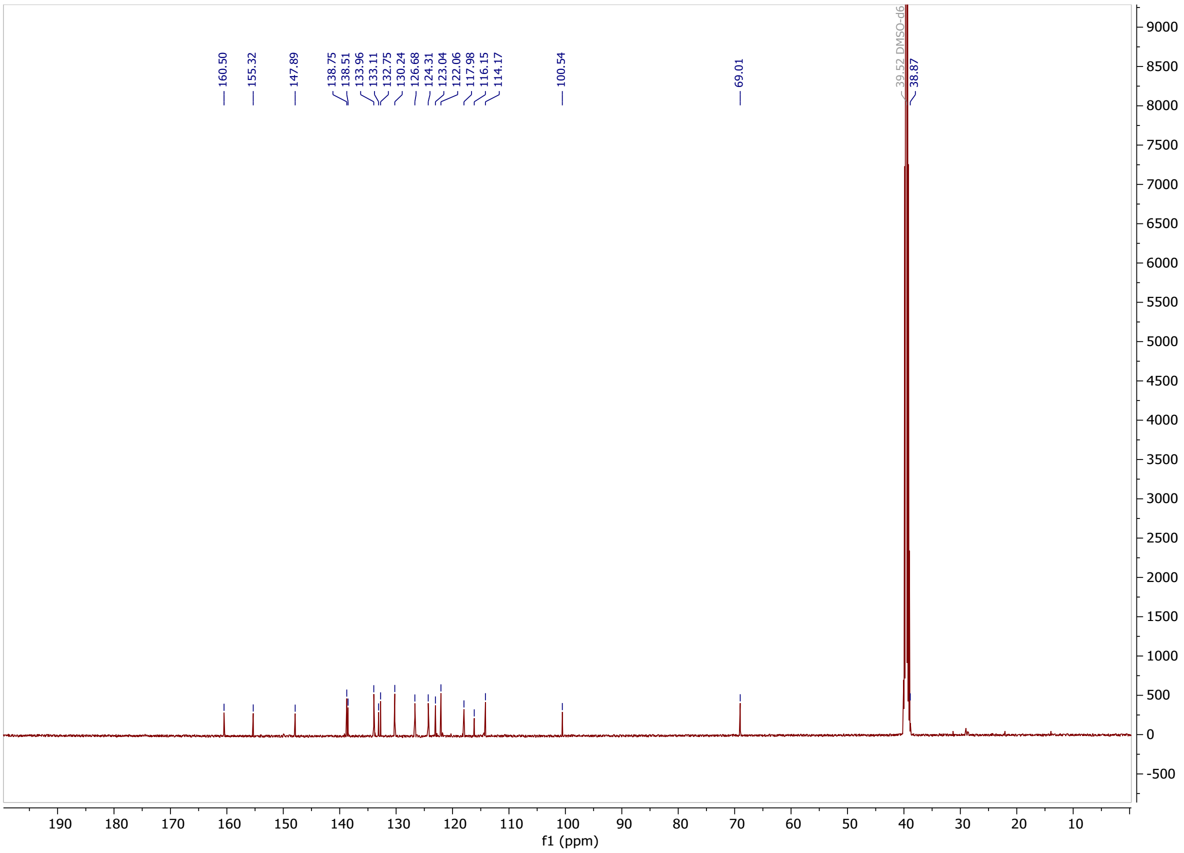

^{1}H and ^{13}C NMR spectra of compound **11**

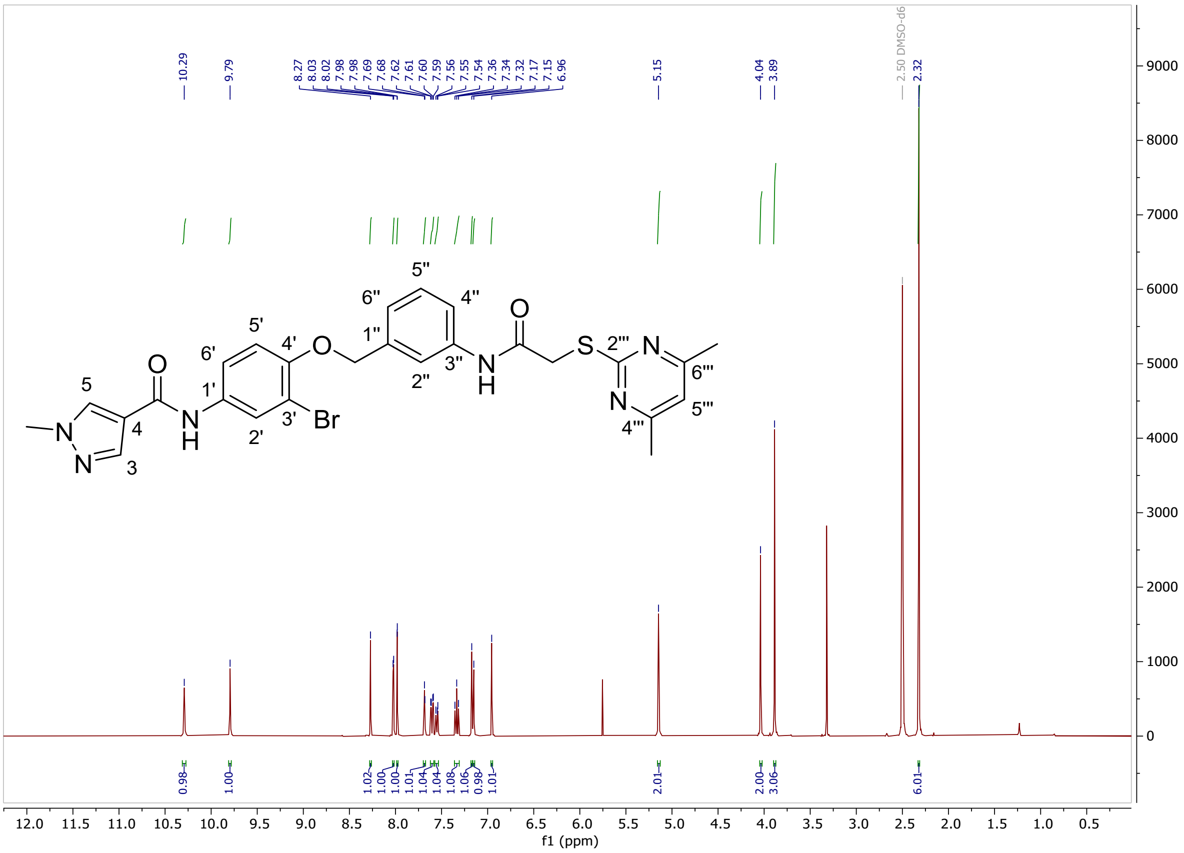

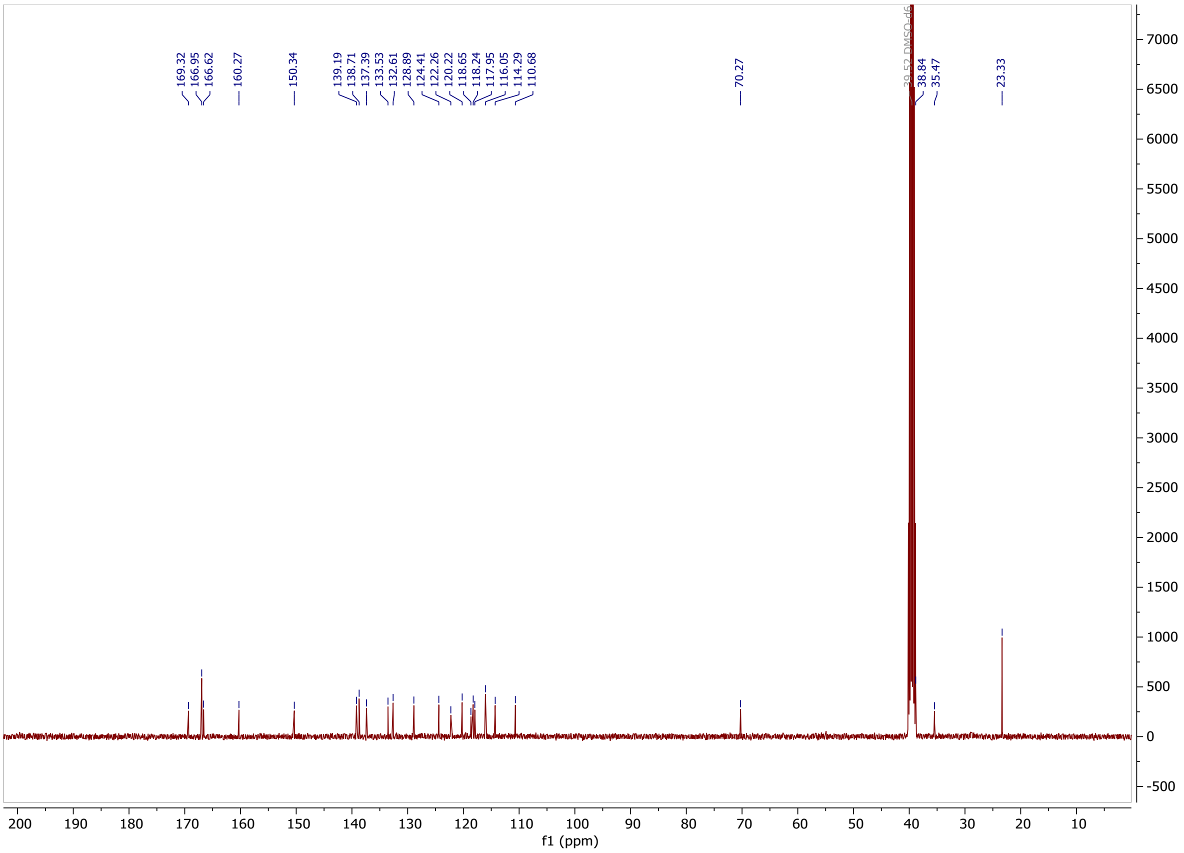

^{1}H and ^{13}C NMR spectra of compound **12**

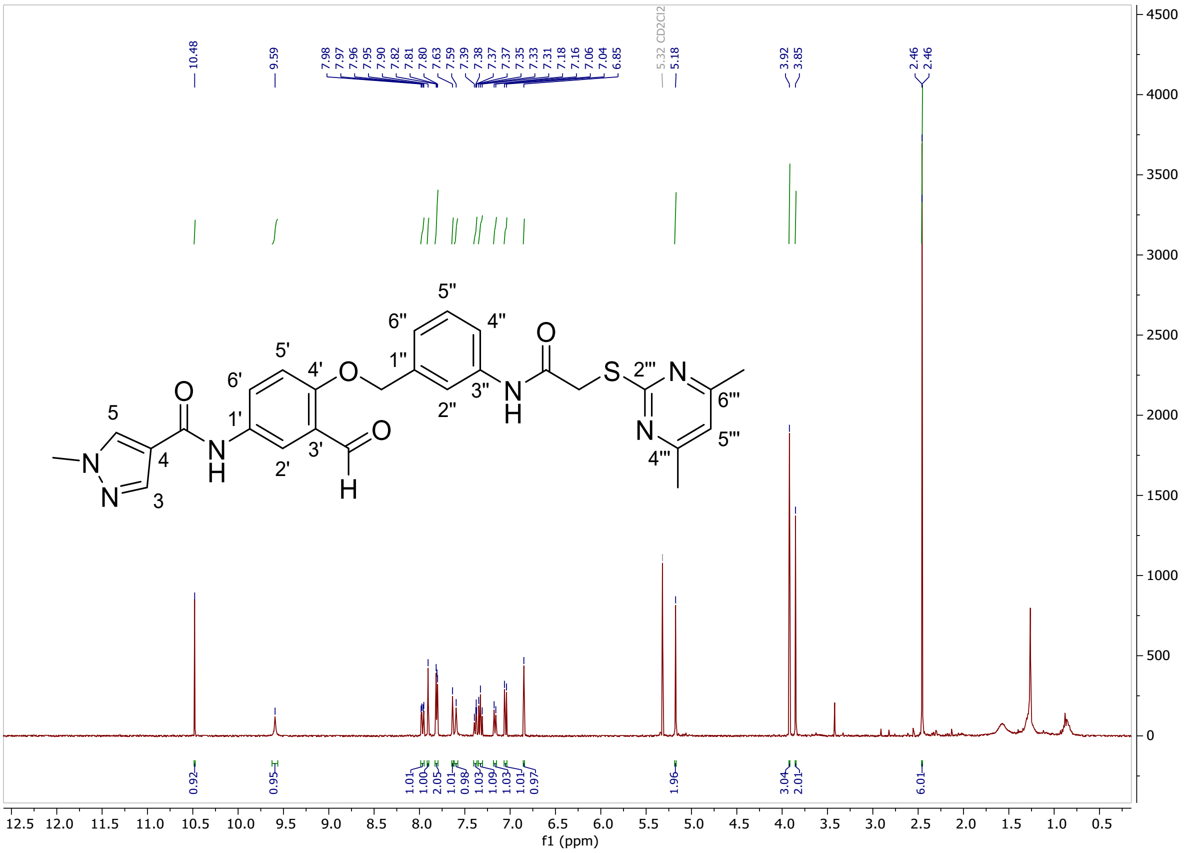

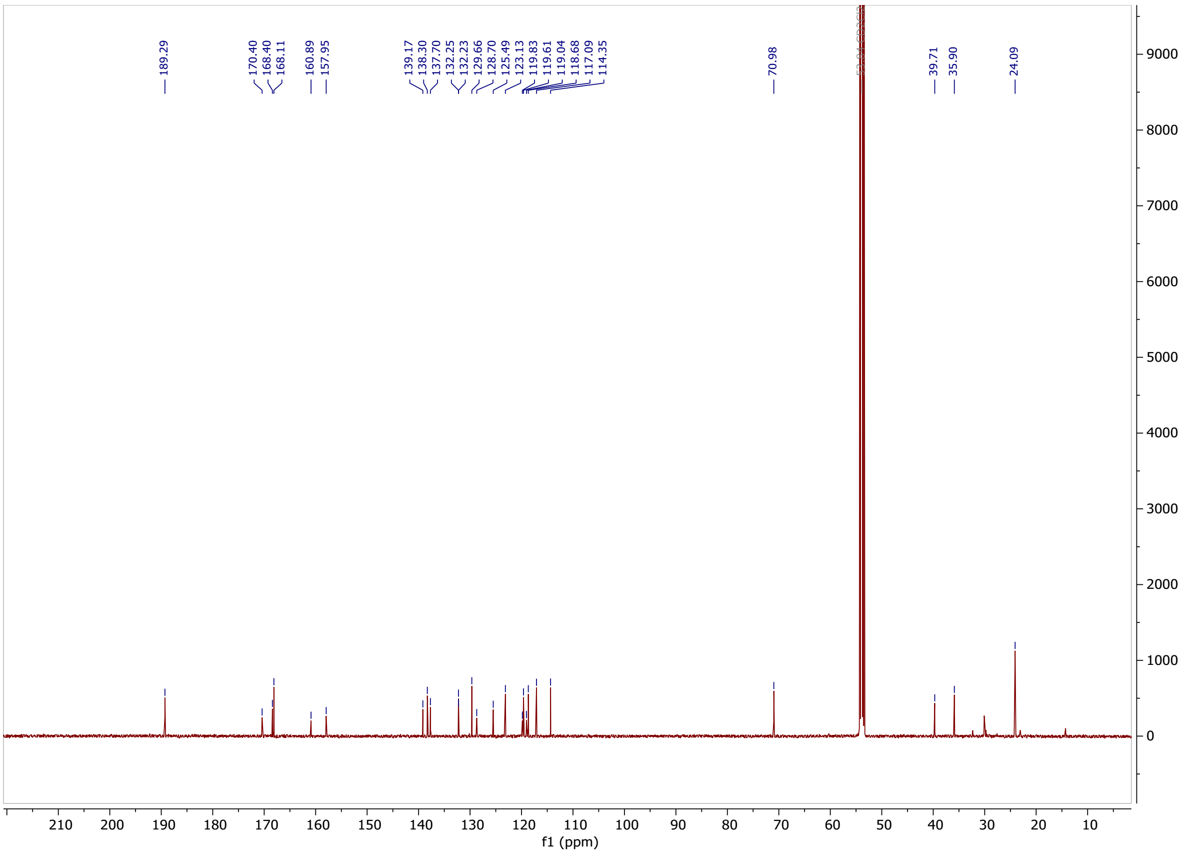

¹H and ¹³C NMR spectra of compound **13**

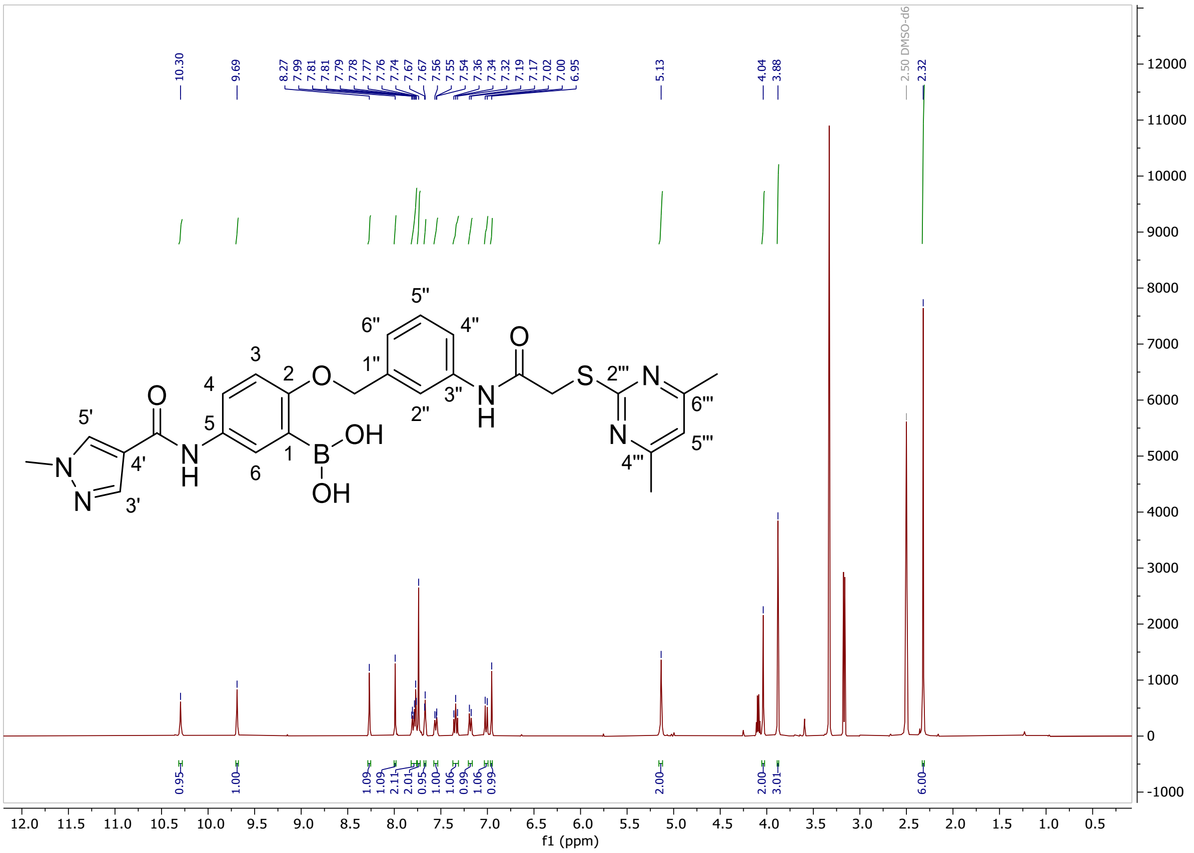

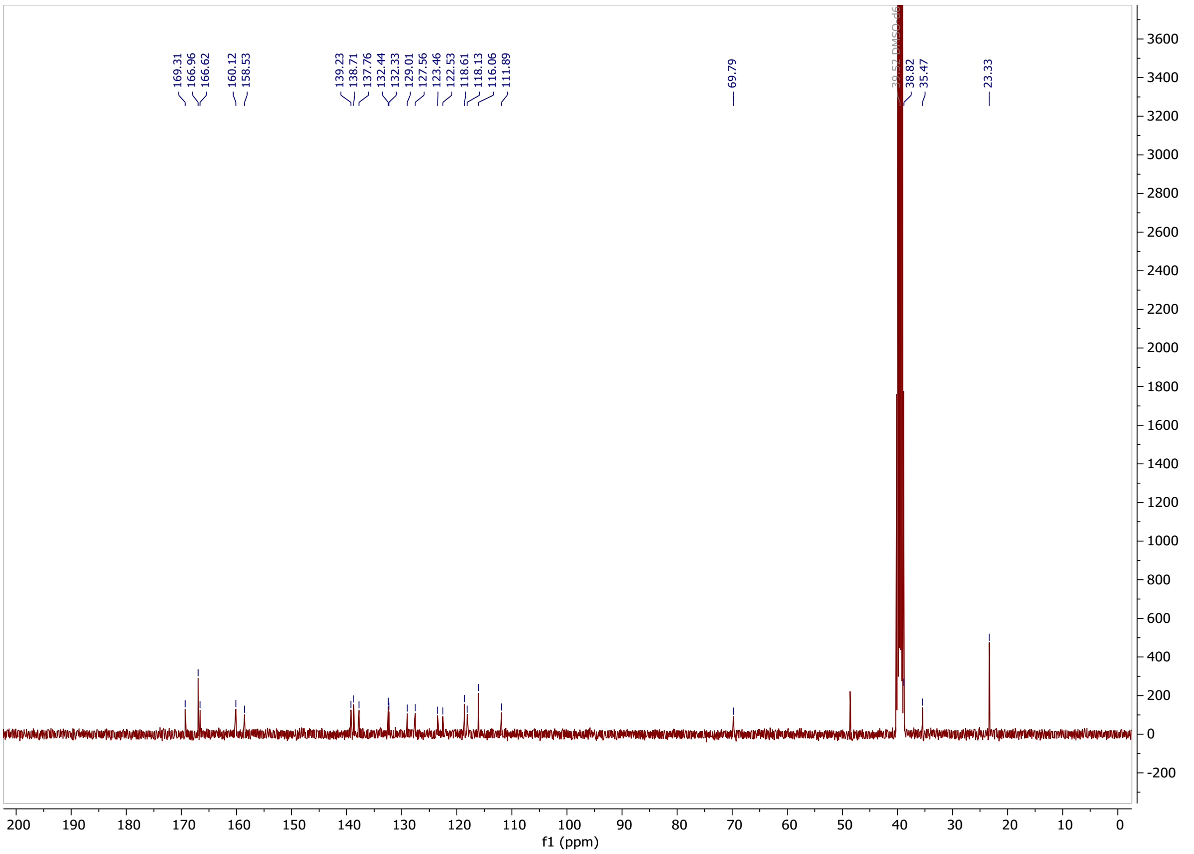

^1H and ^{13}C NMR spectra of compound **14**

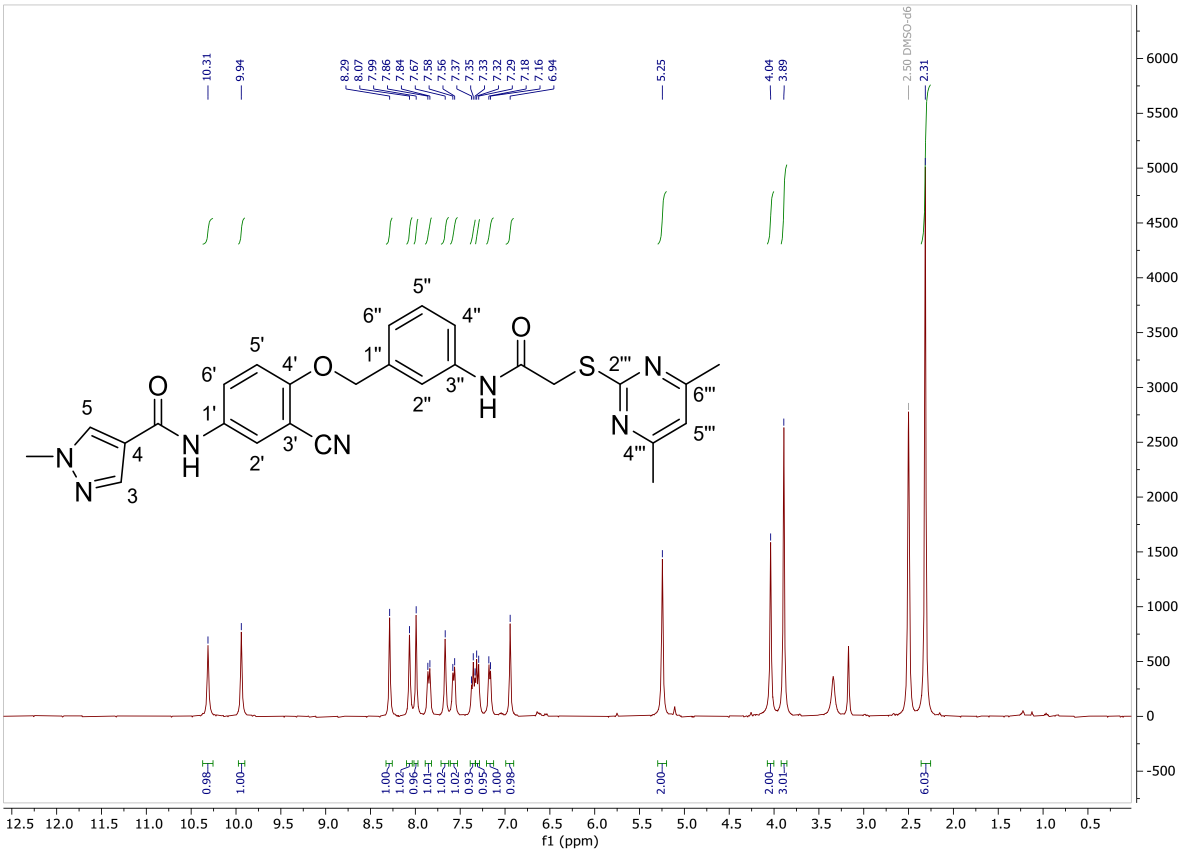

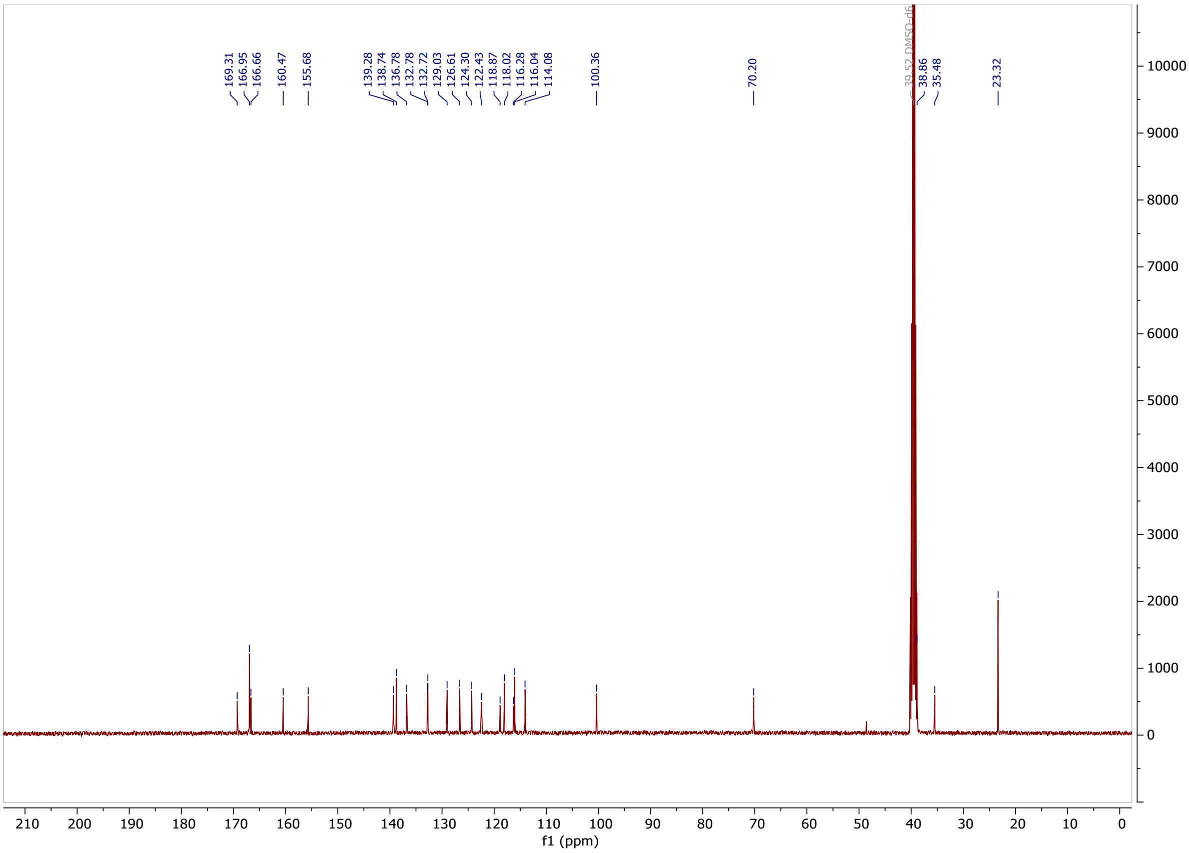

^{1}H and ^{13}C NMR spectra of compound **20**

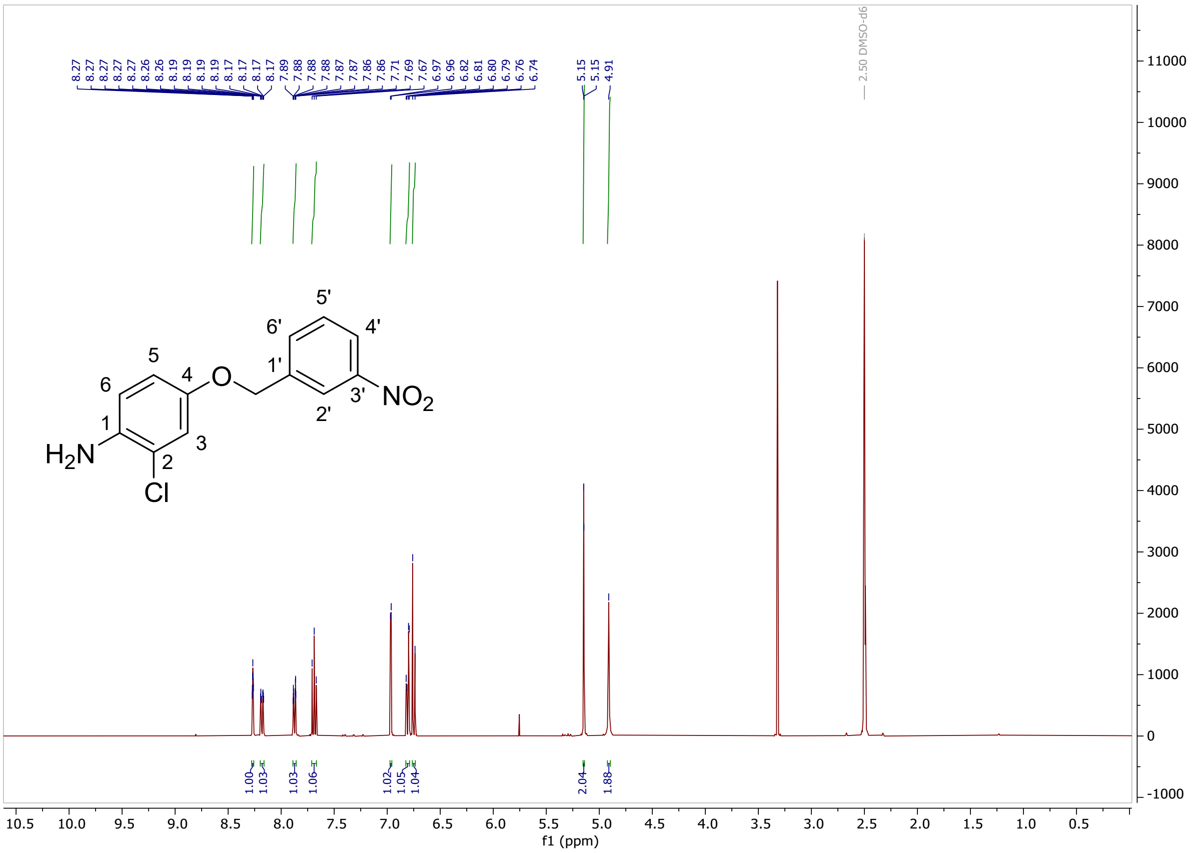

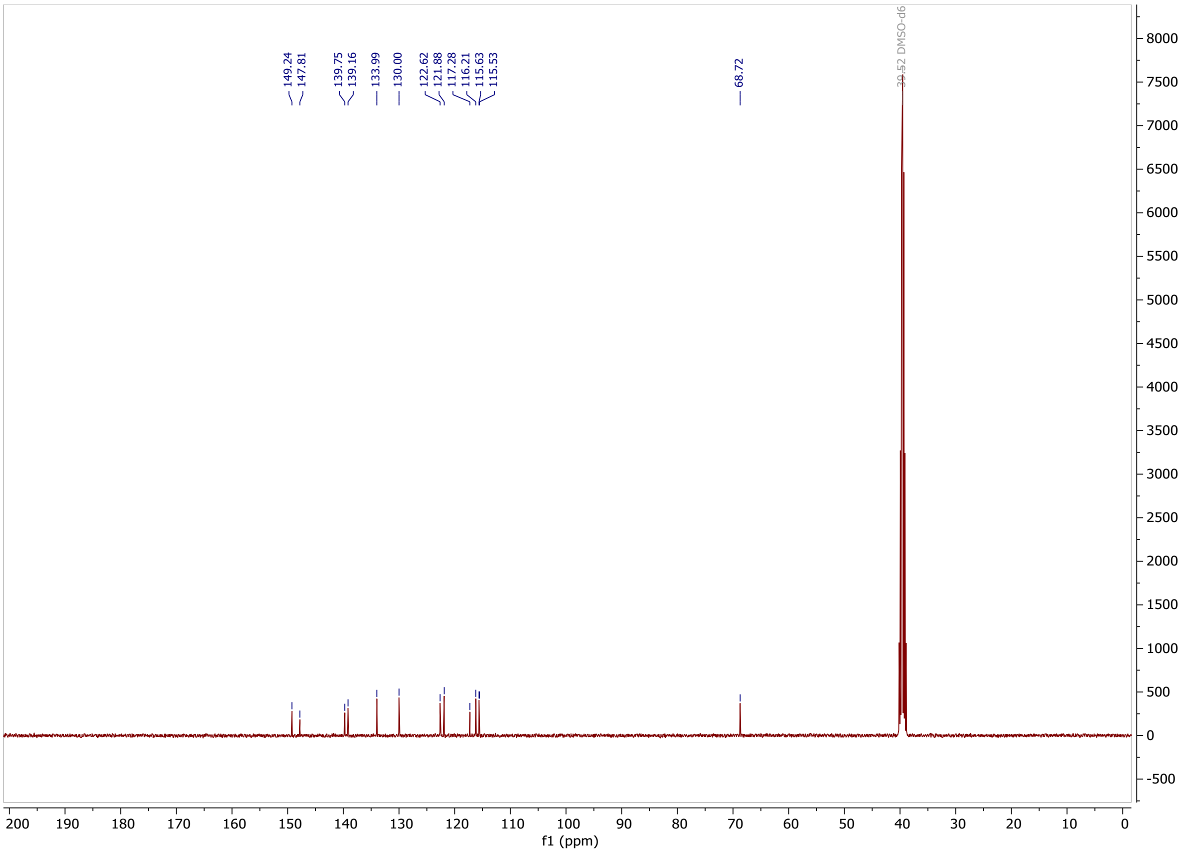

^{1}H and ^{13}C NMR spectra of compound **21**

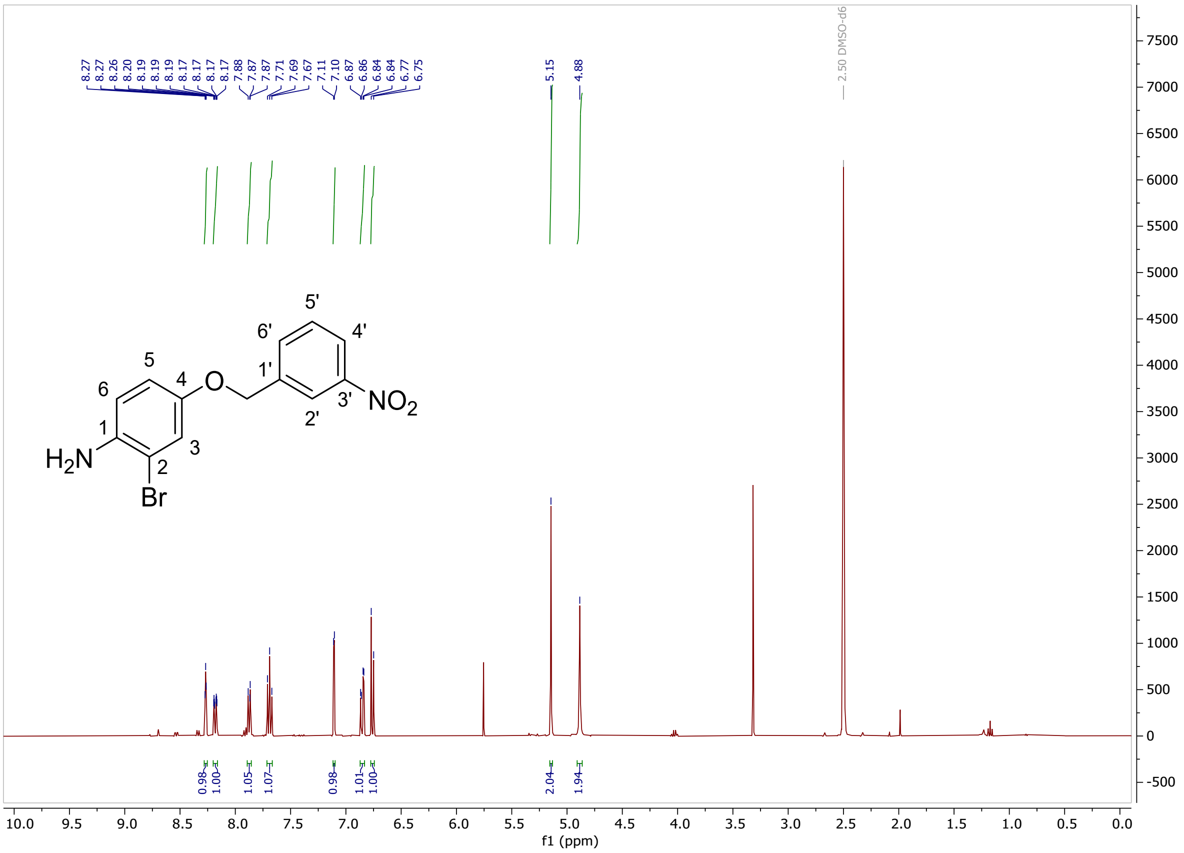

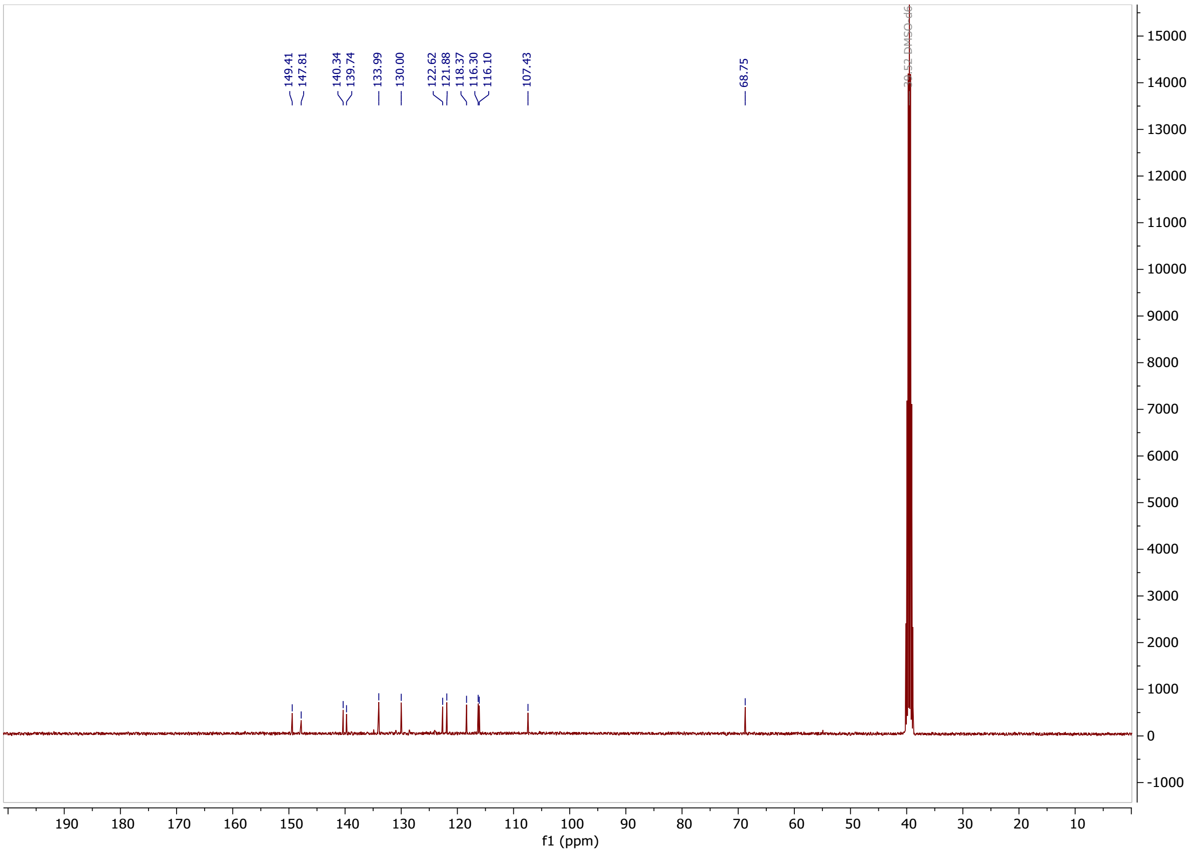

^{1}H and ^{13}C NMR spectra of compound **22**

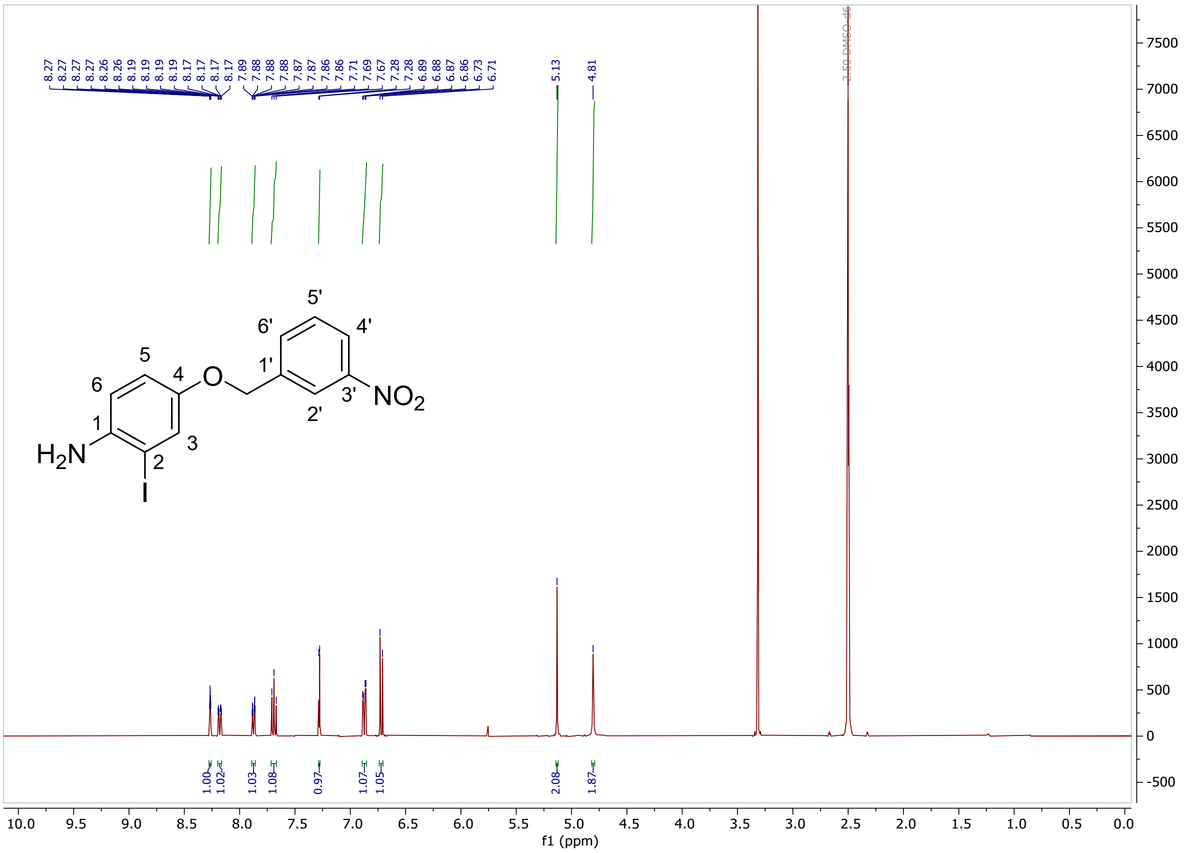

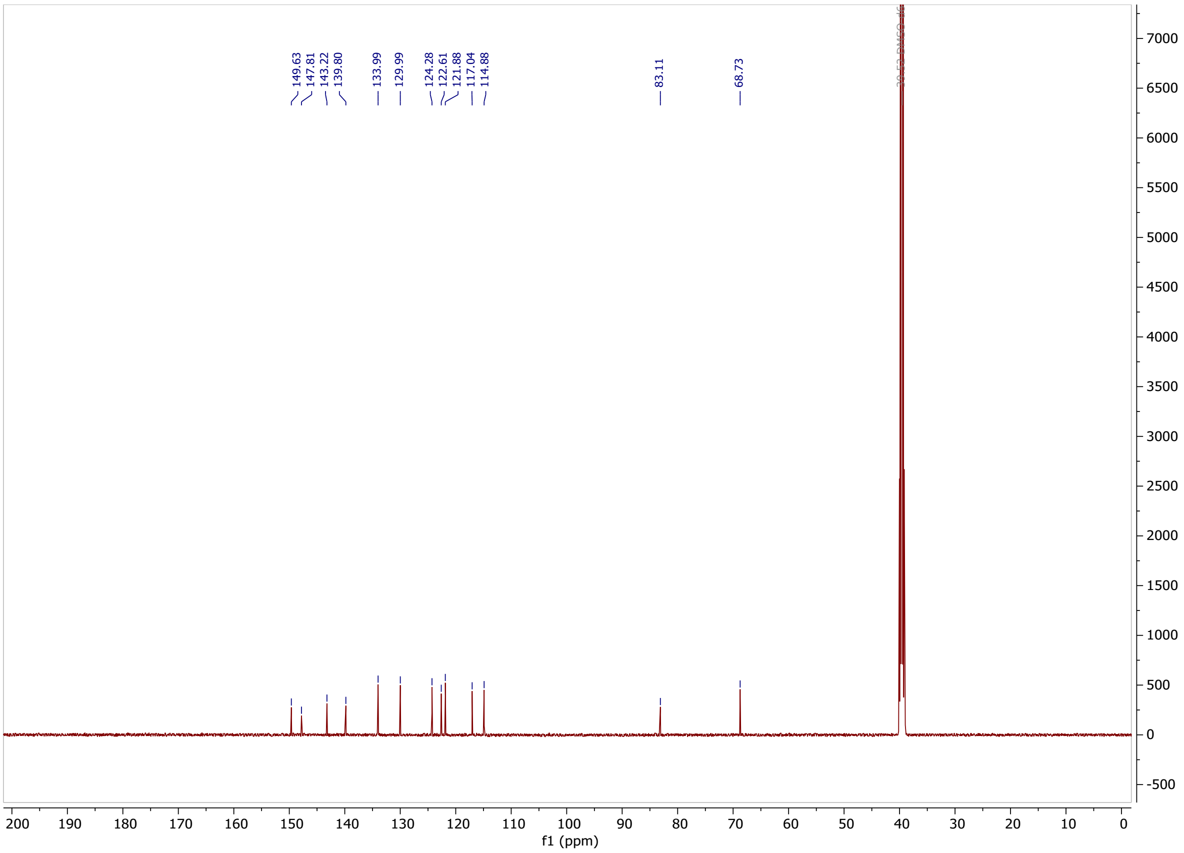

^{1}H and ^{13}C NMR spectra of compound **23**

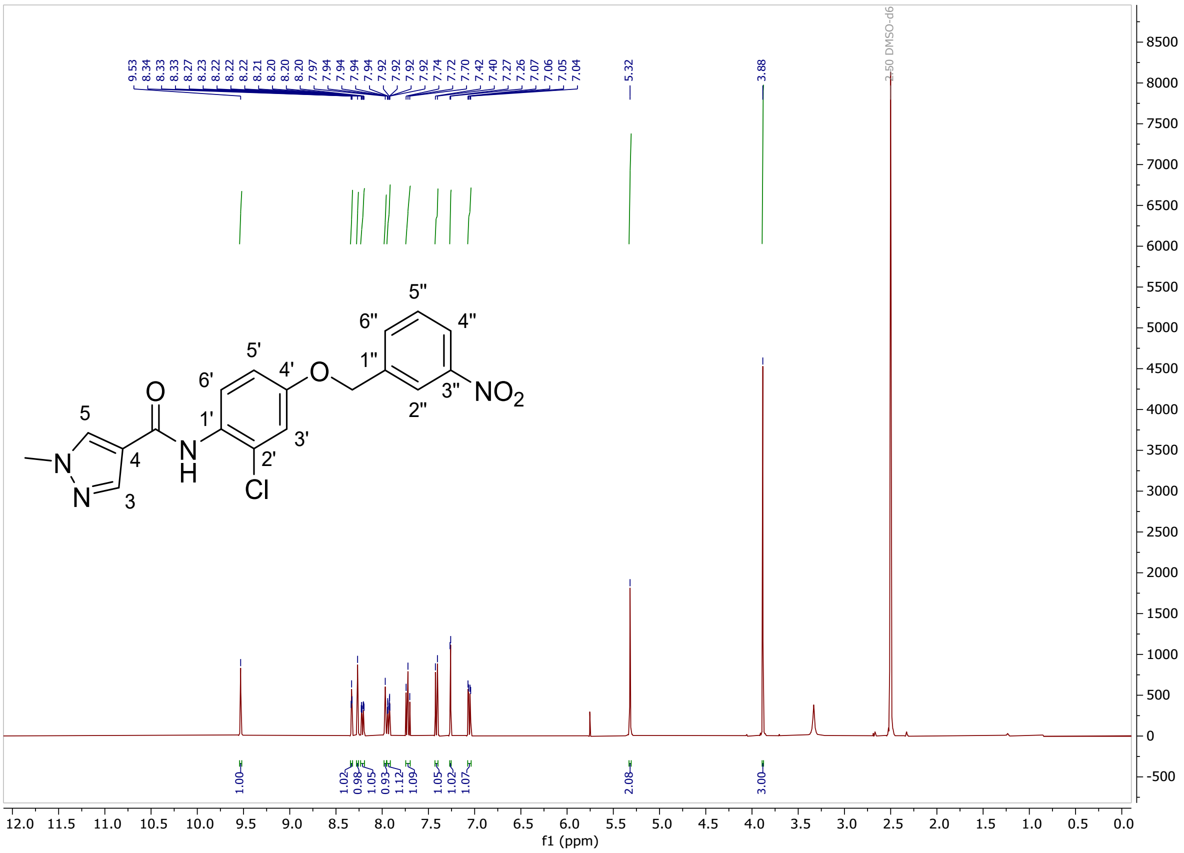

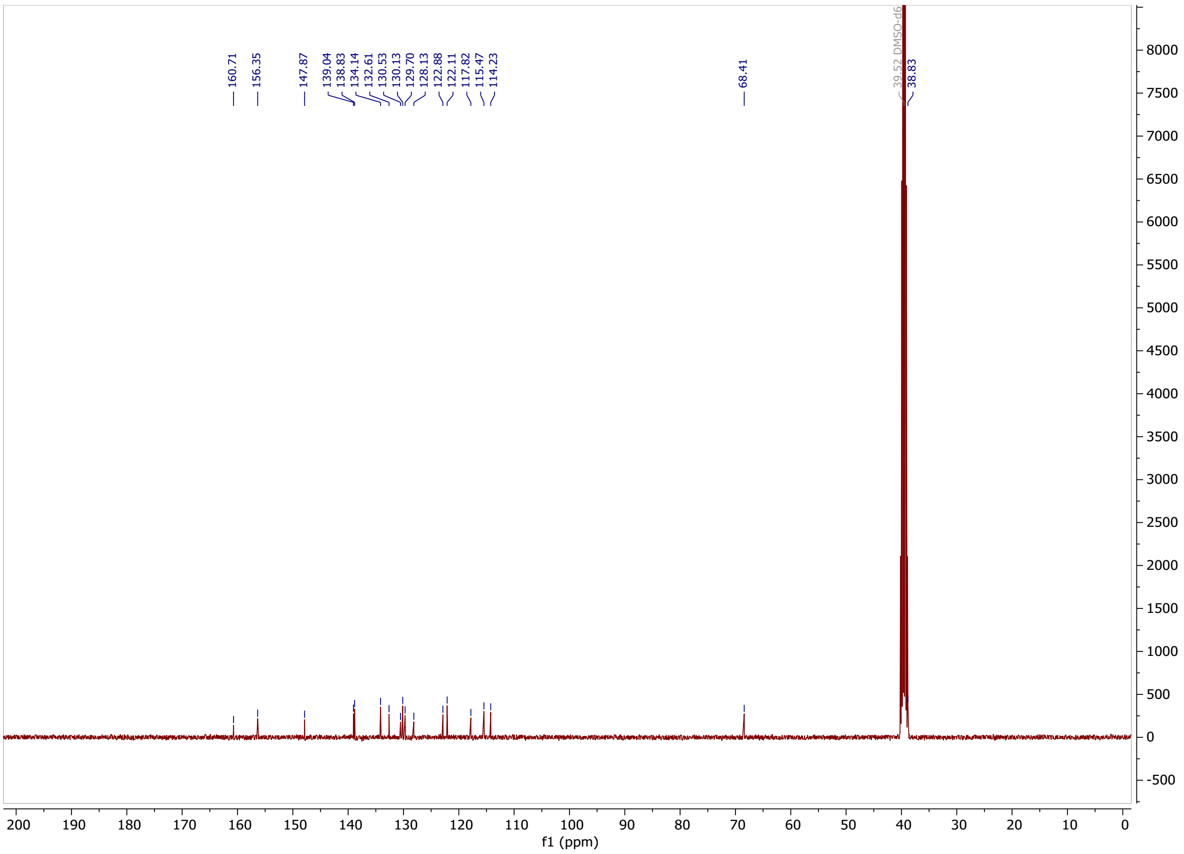

^{1}H and ^{13}C NMR spectra of compound **24**

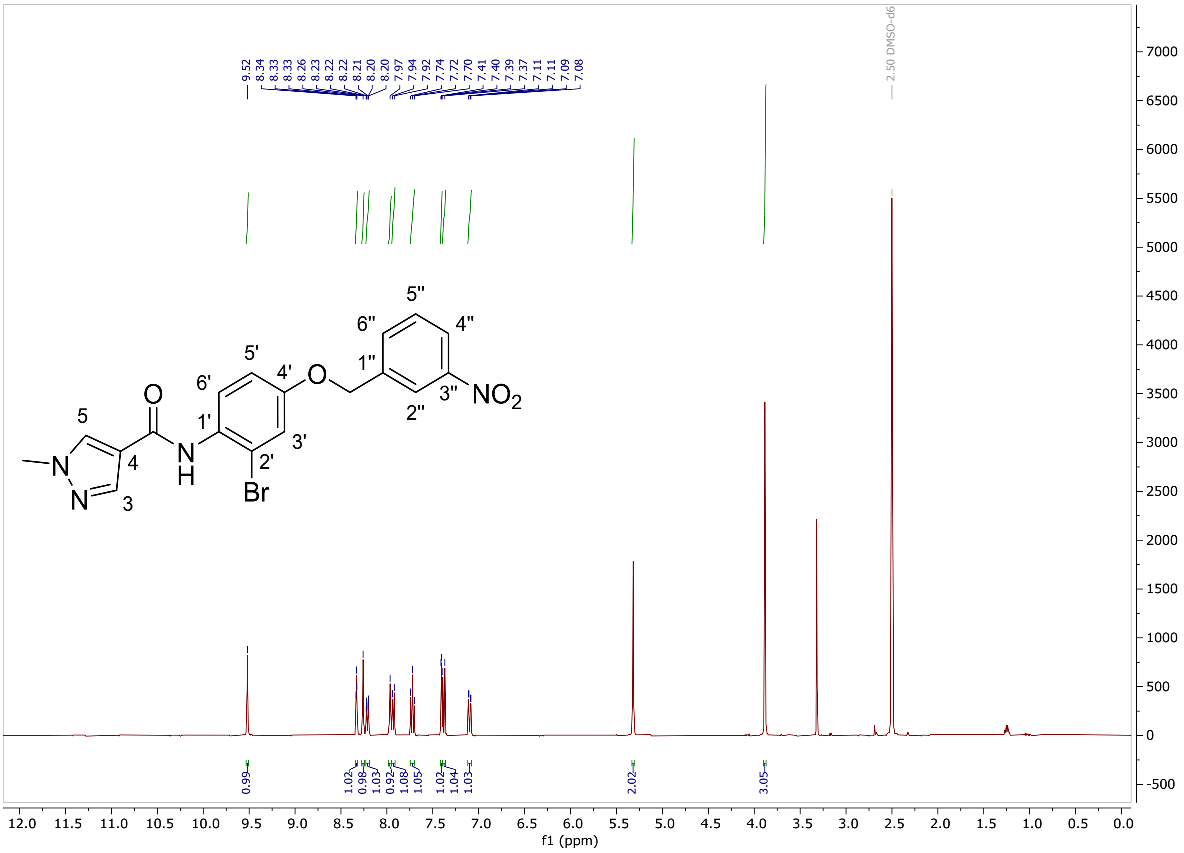

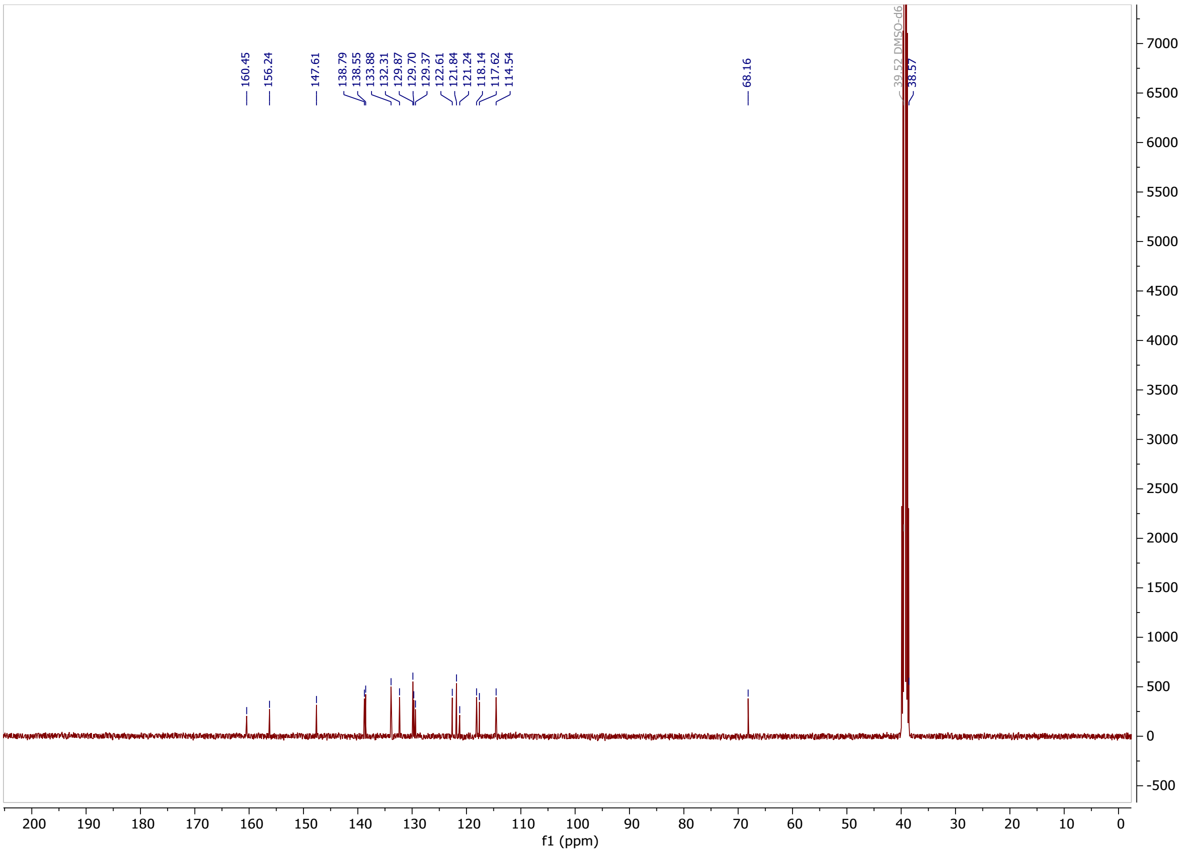

^{1}H and ^{13}C NMR spectra of compound **25**

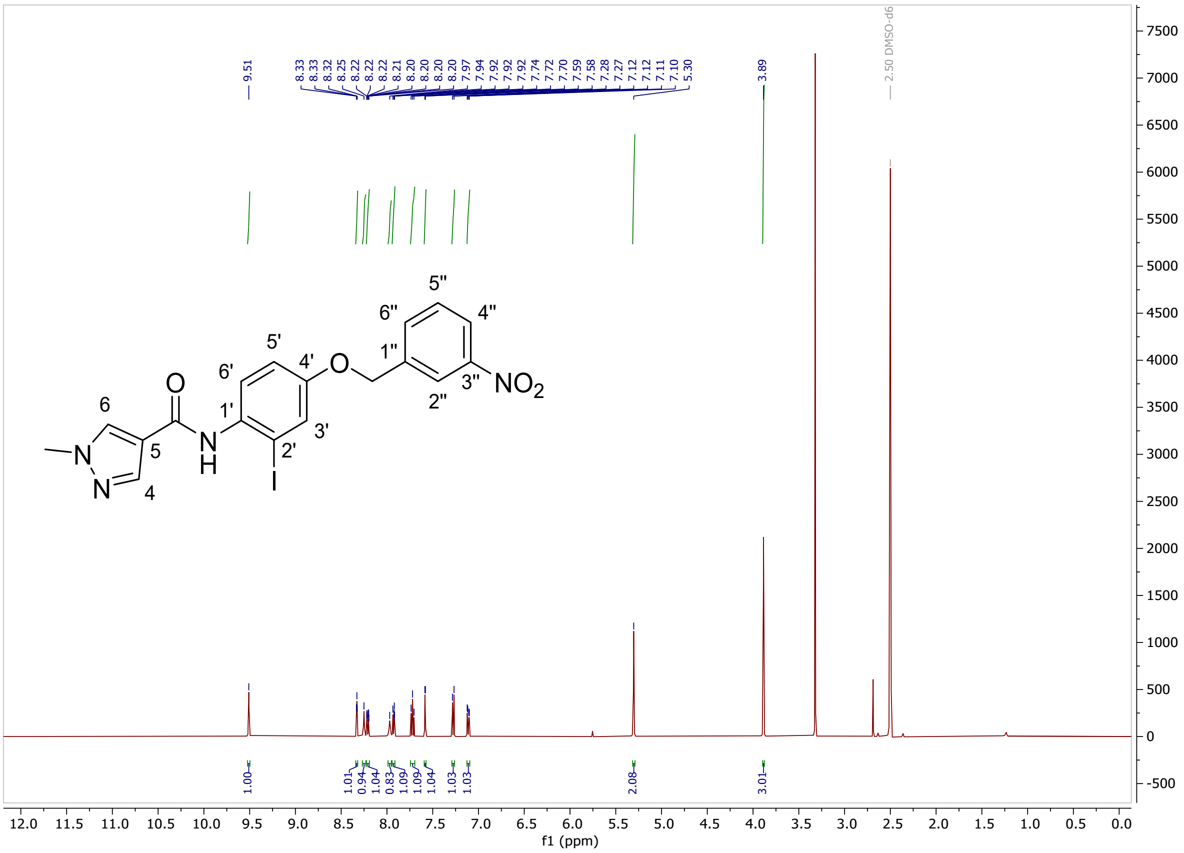

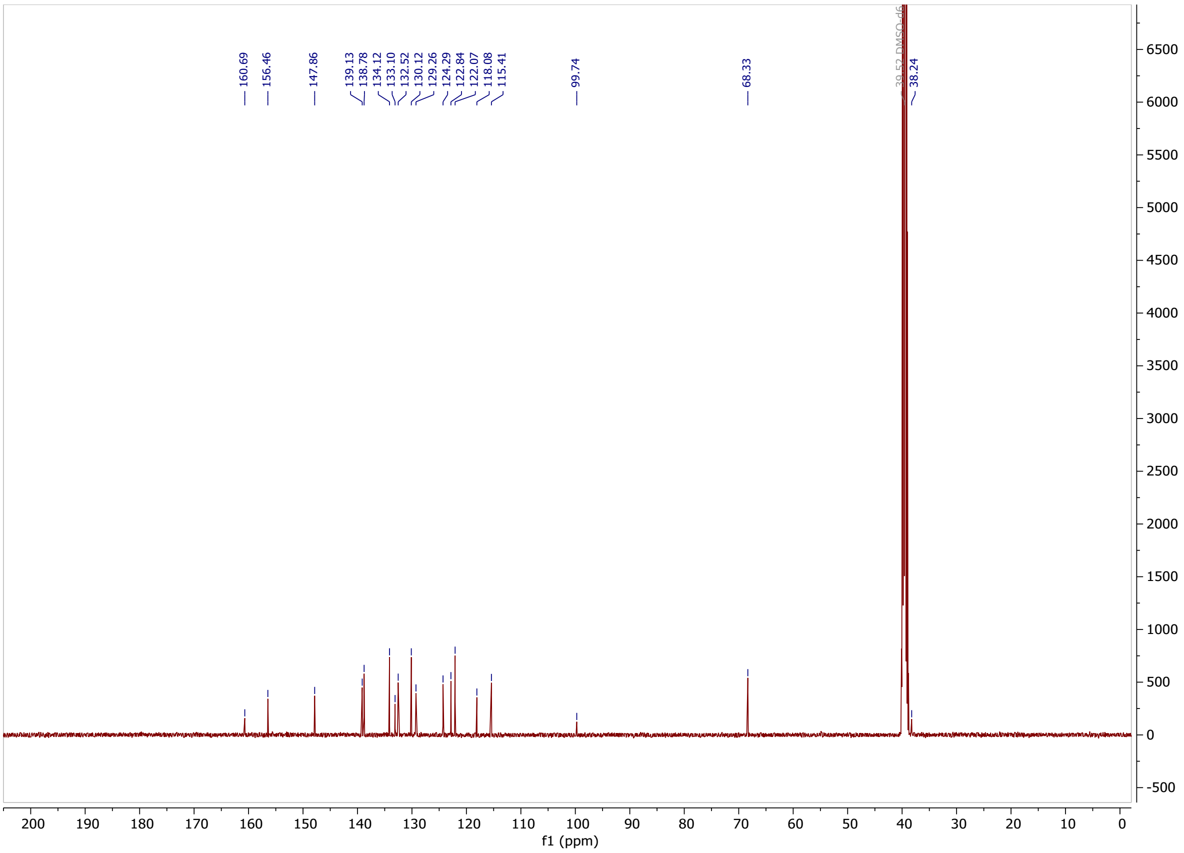

^{1}H and ^{13}C NMR spectra of compound **26**

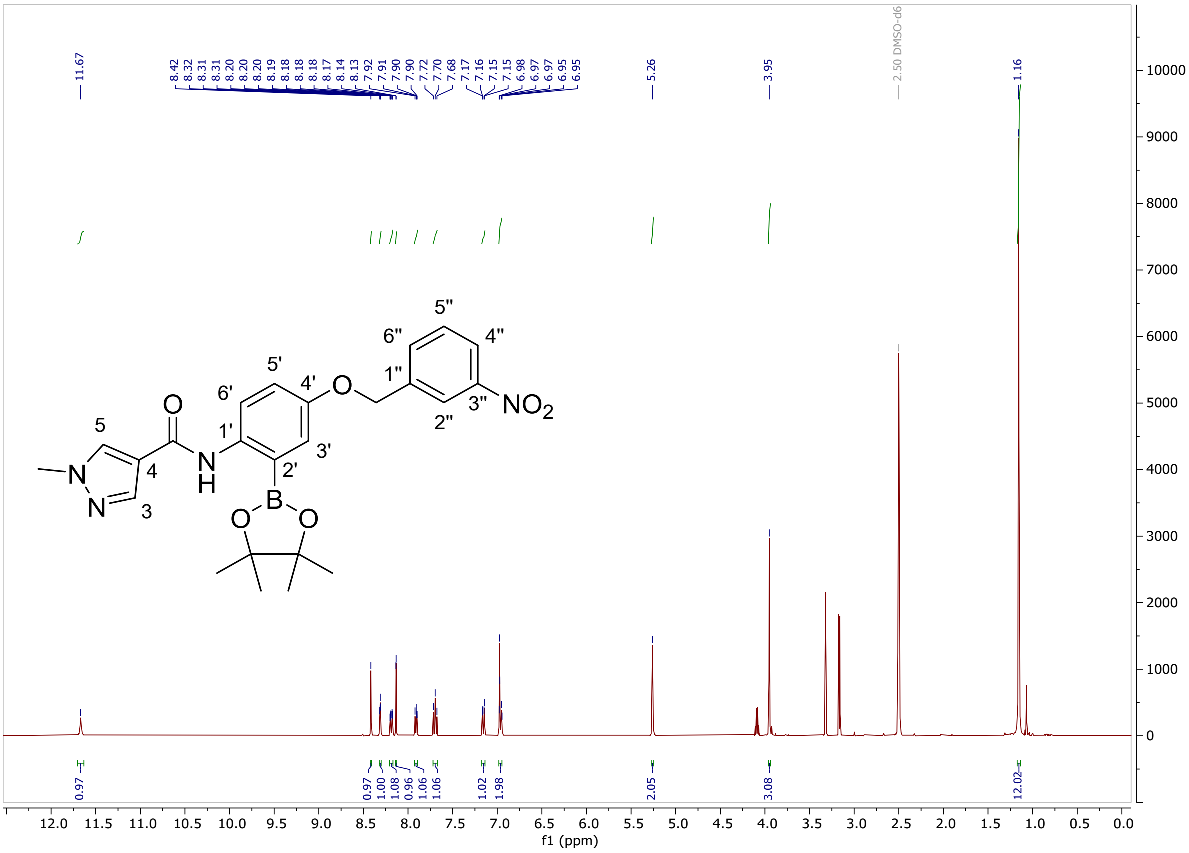

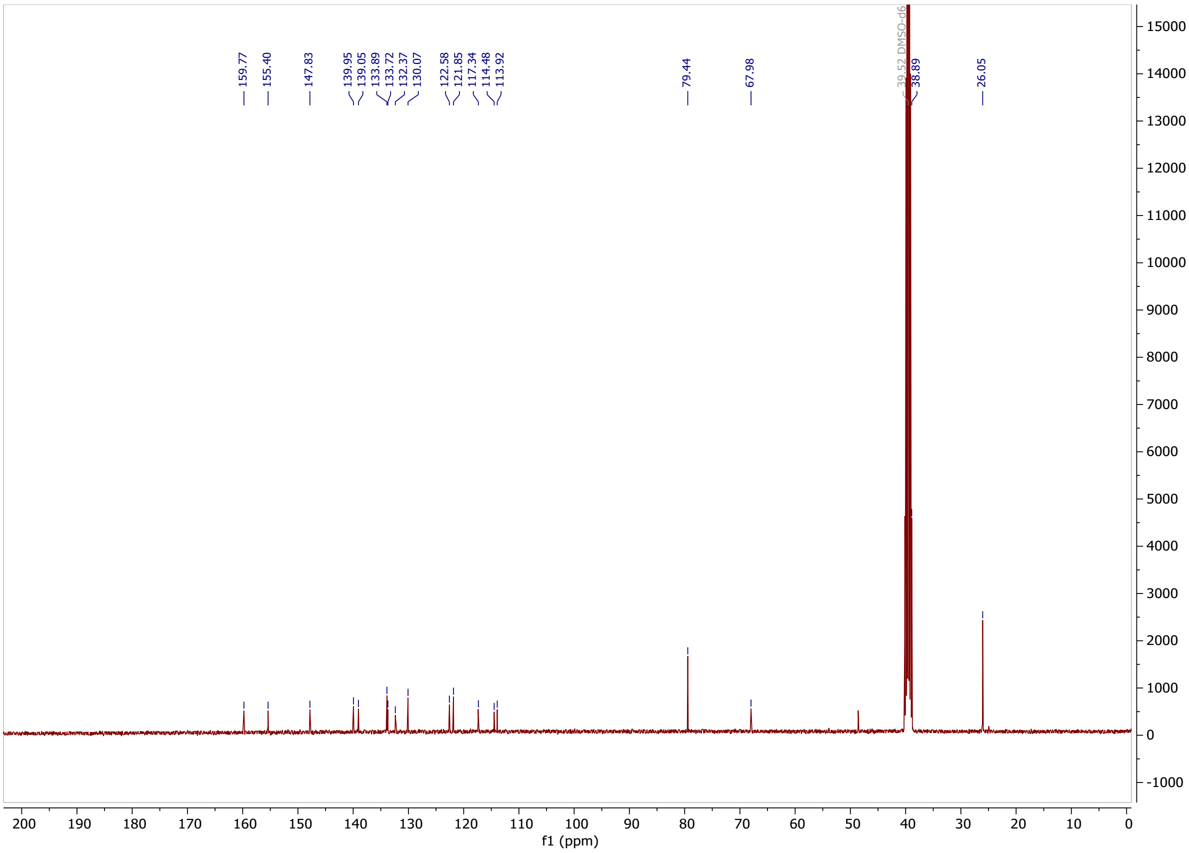

^{1}H and ^{13}C NMR spectra of compound **27**

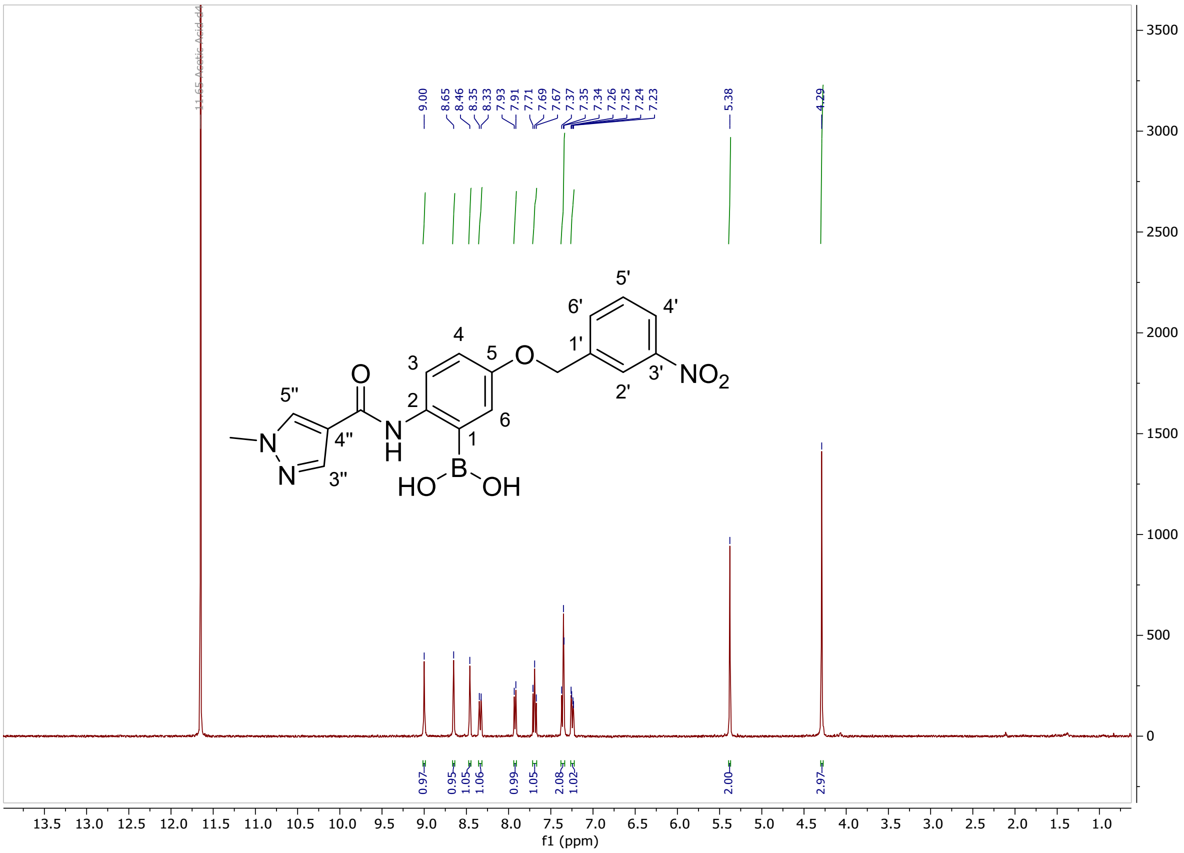

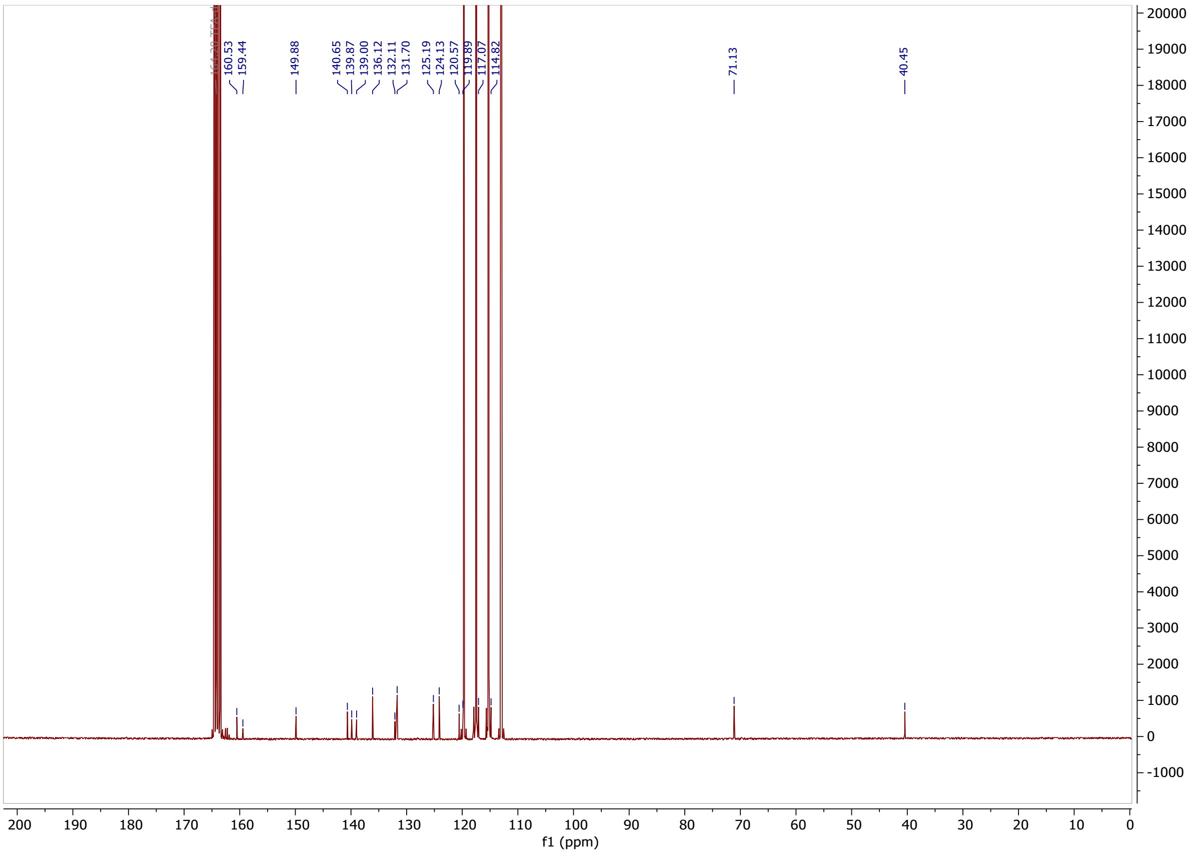

^1H and ^{13}C NMR spectra of compound **28**

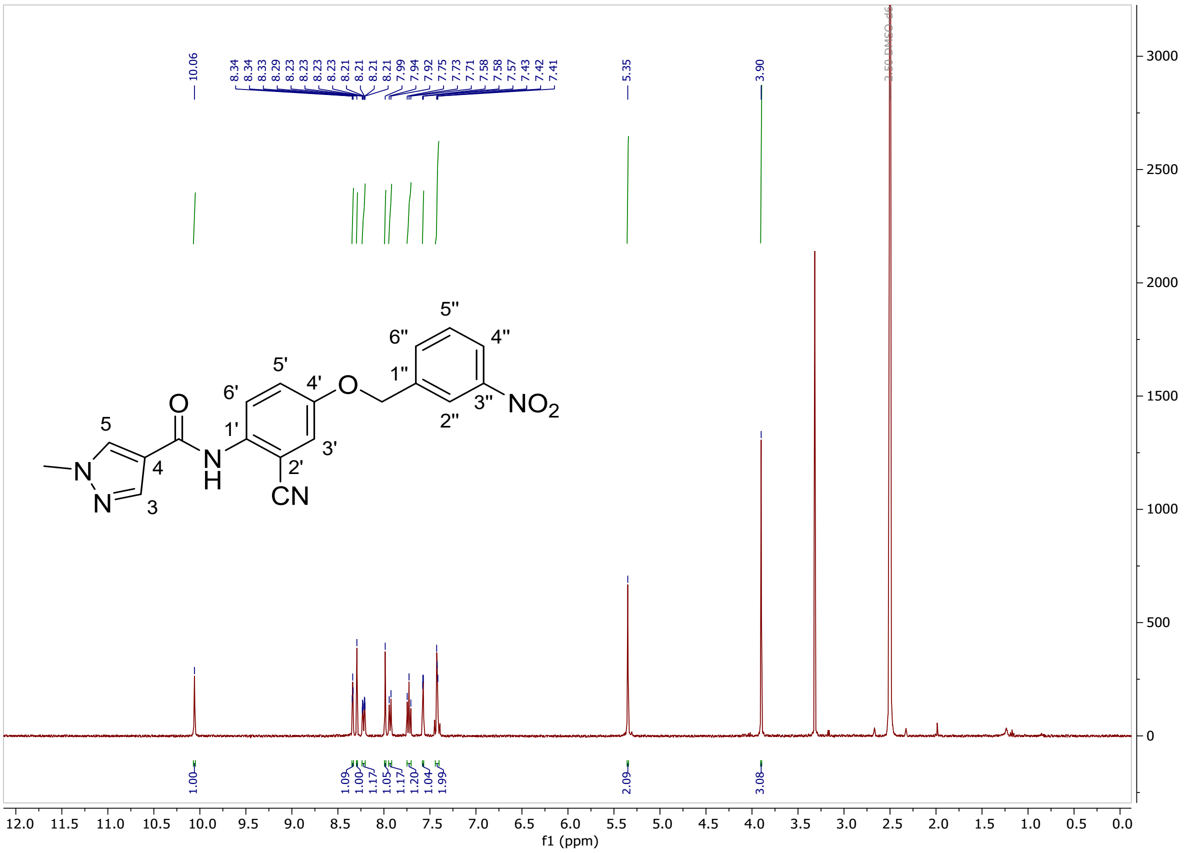

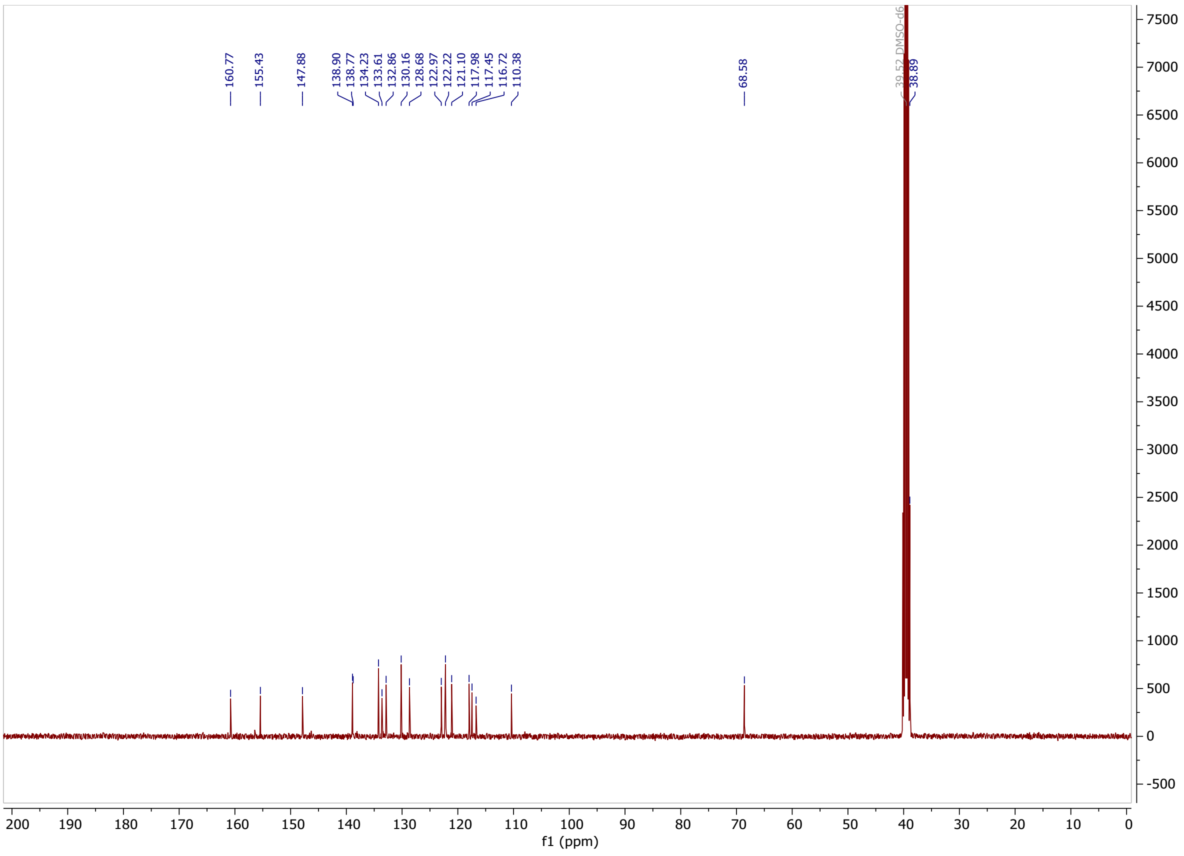

^{1}H and ^{13}C NMR spectra of compound **29**

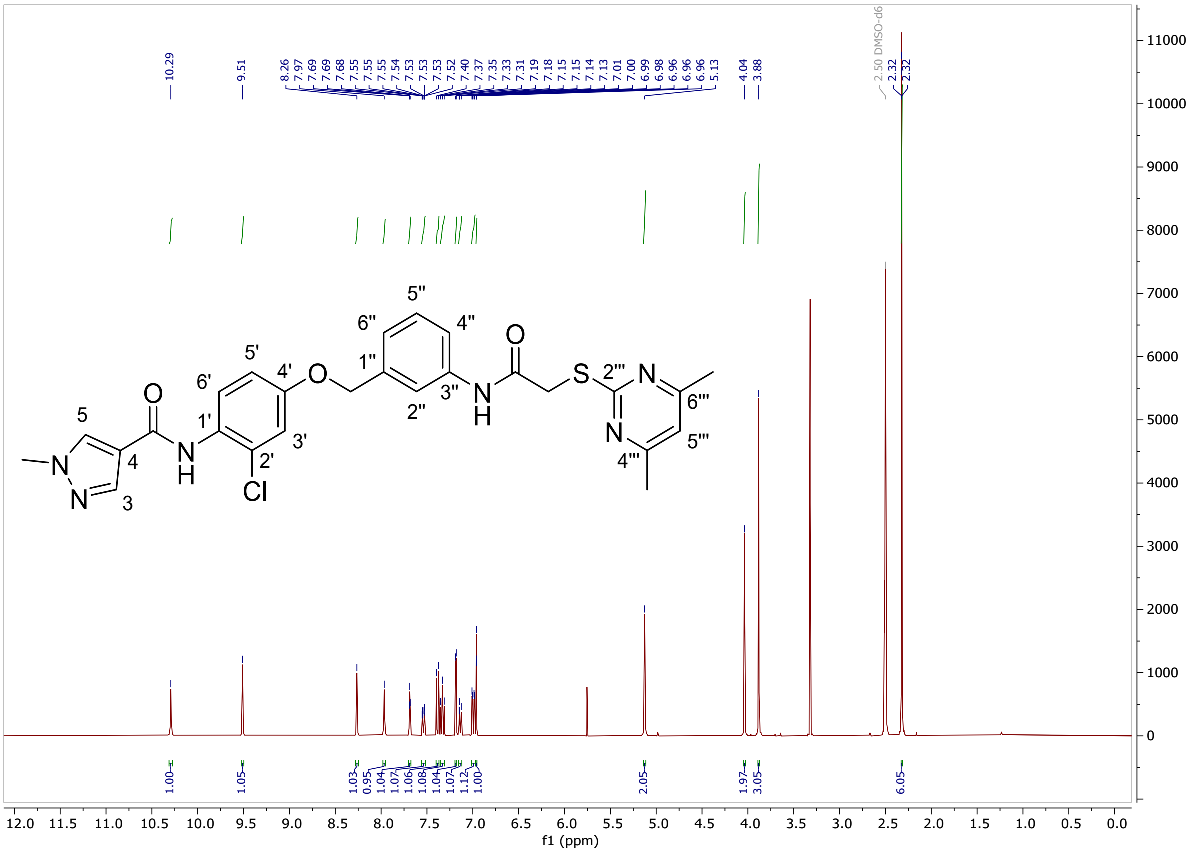

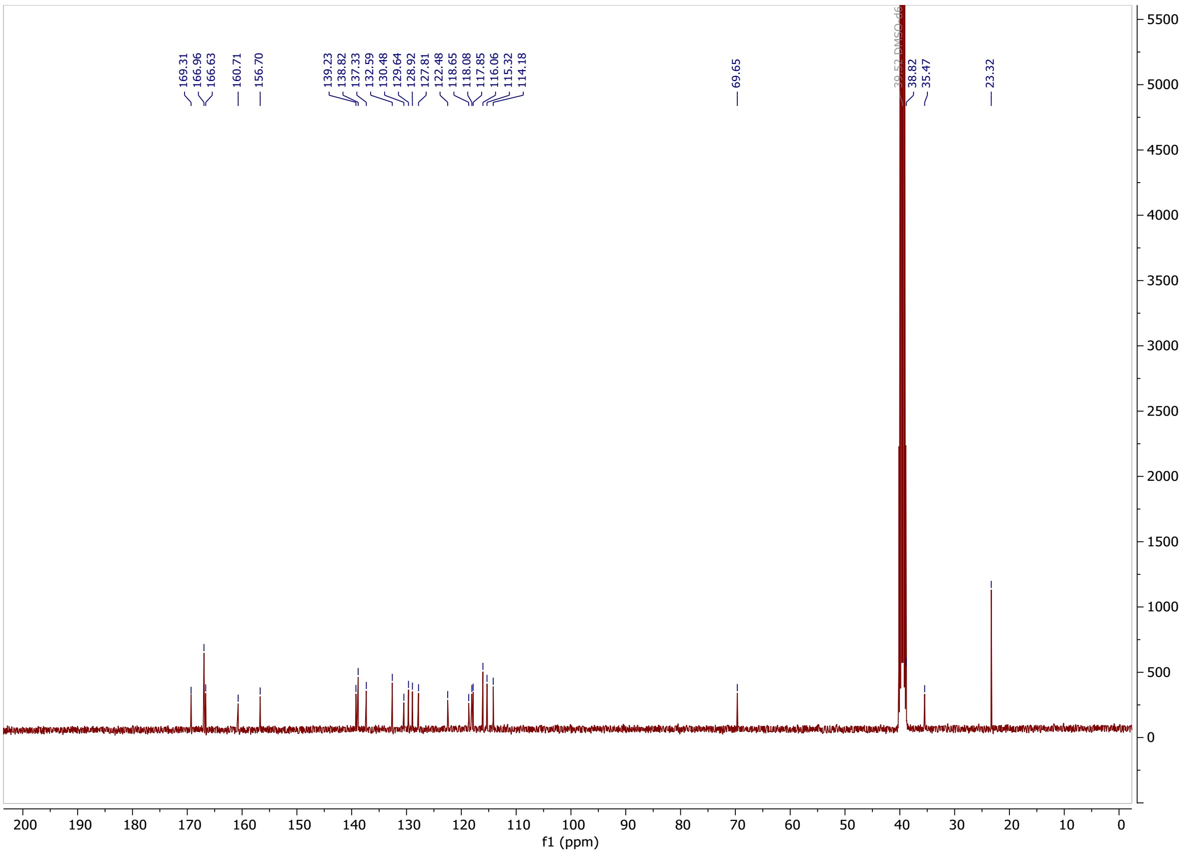

^{1}H and ^{13}C NMR spectra of compound **30**

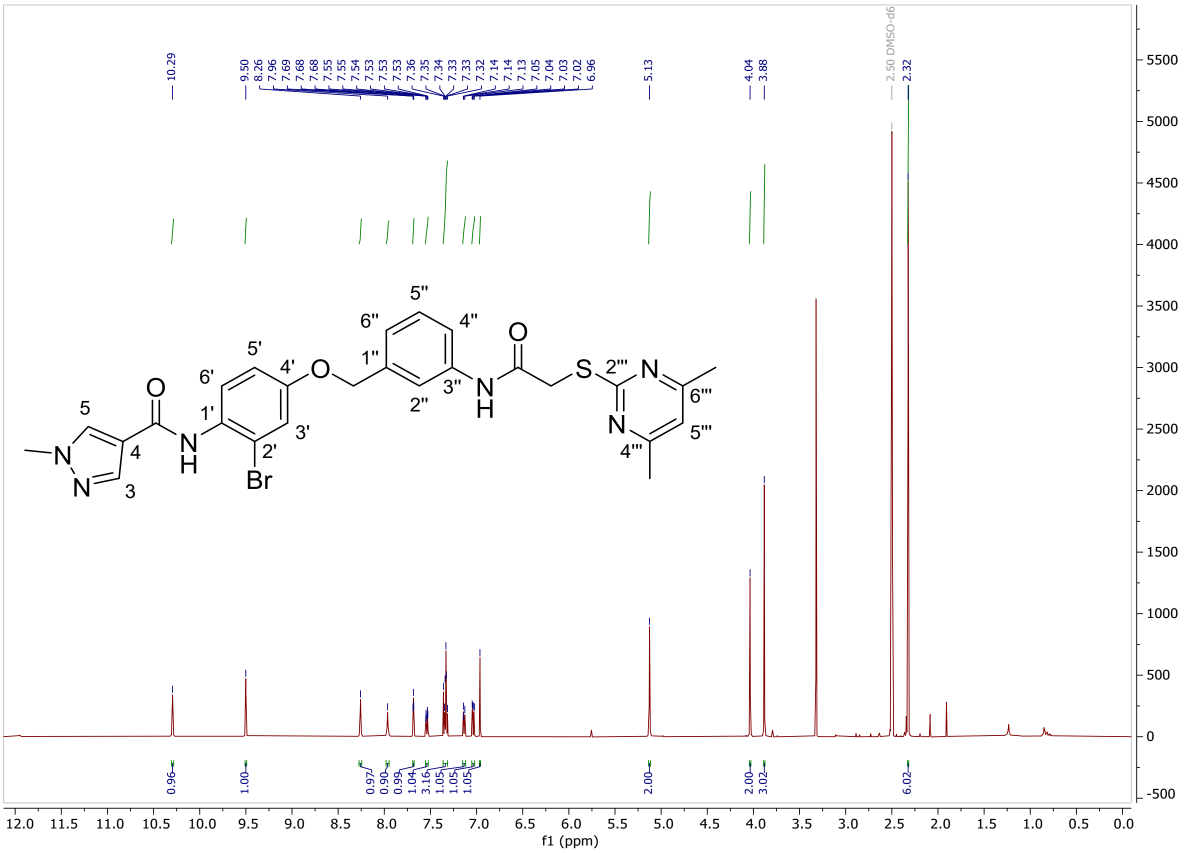

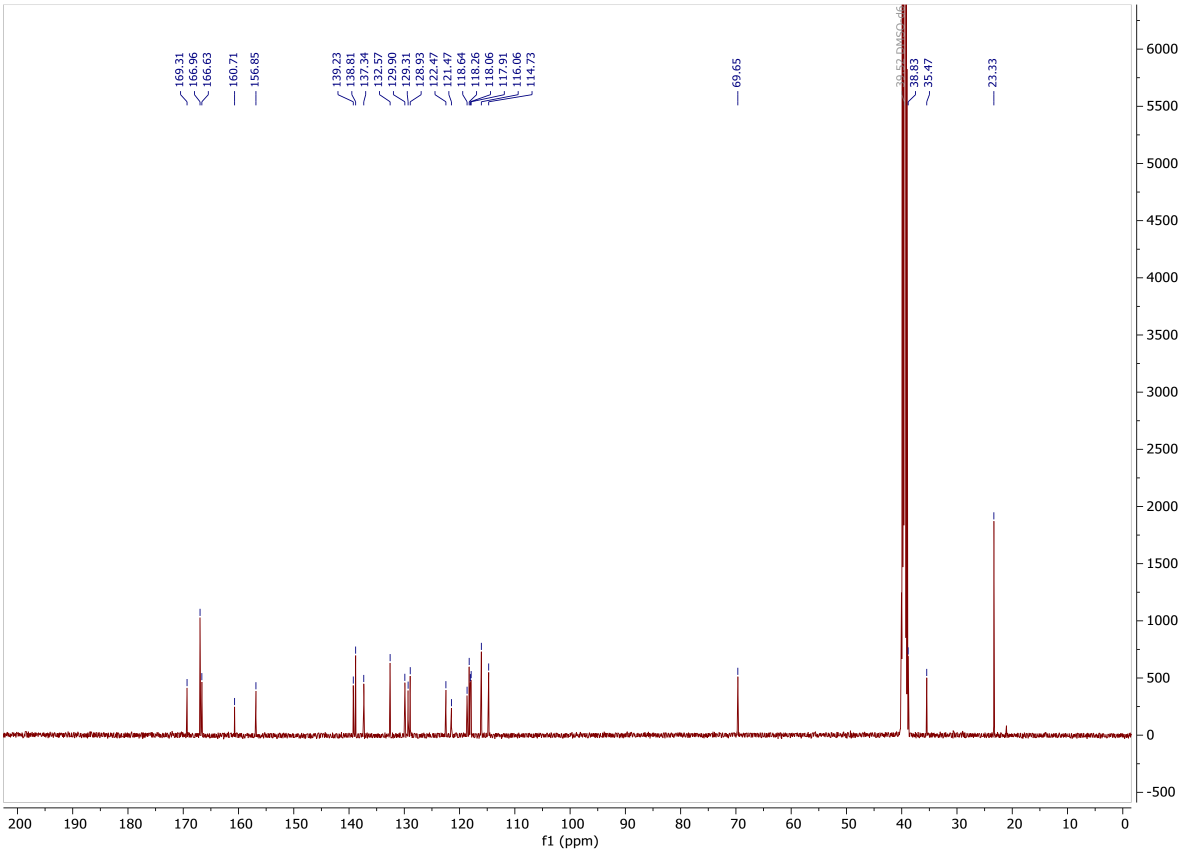

^{1}H and ^{13}C NMR spectra of compound **31**

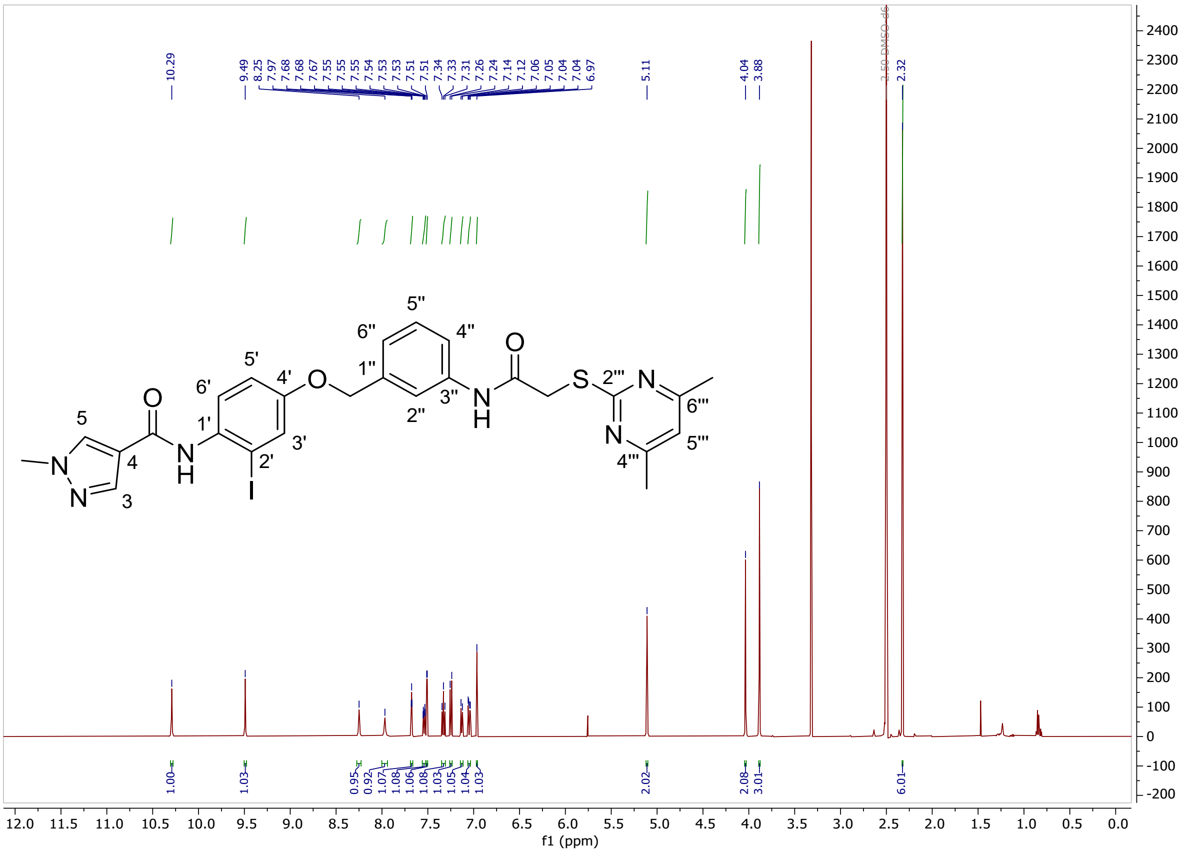

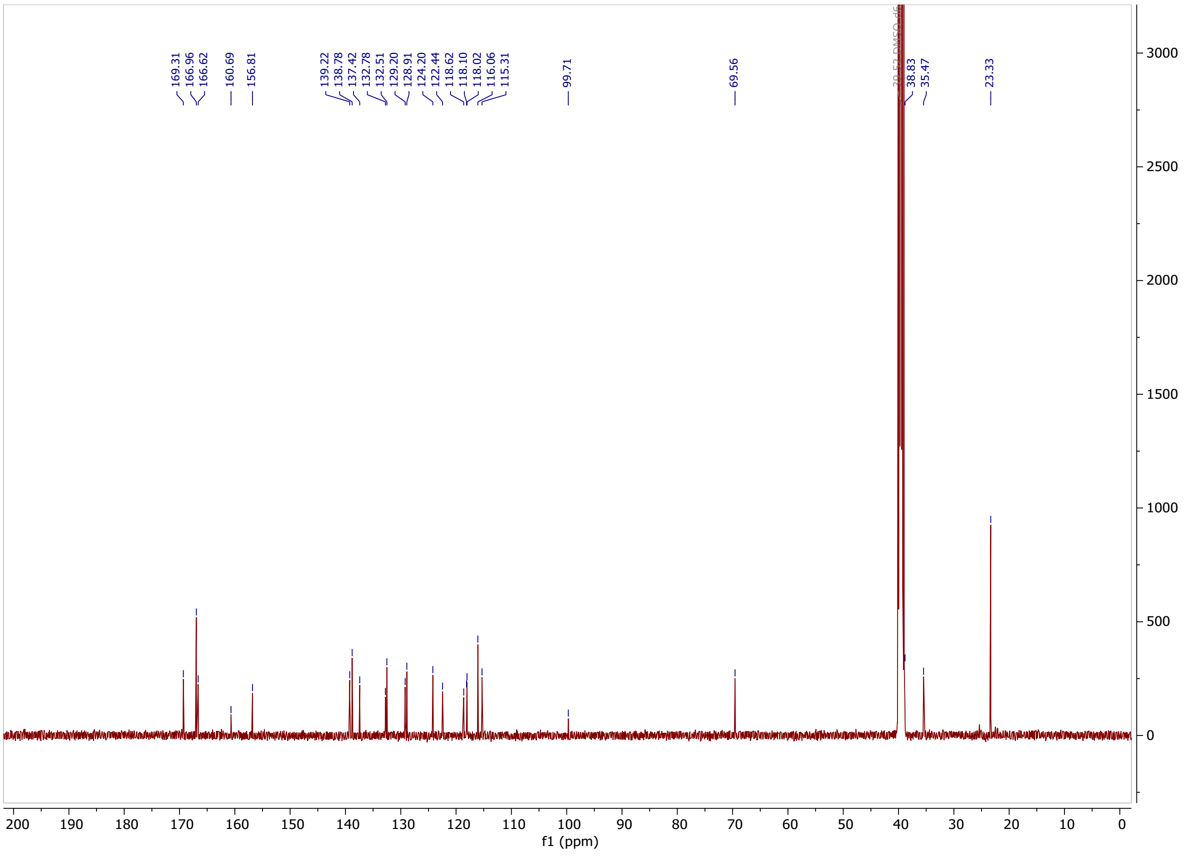

^{1}H and ^{13}C NMR spectra of compound **32**

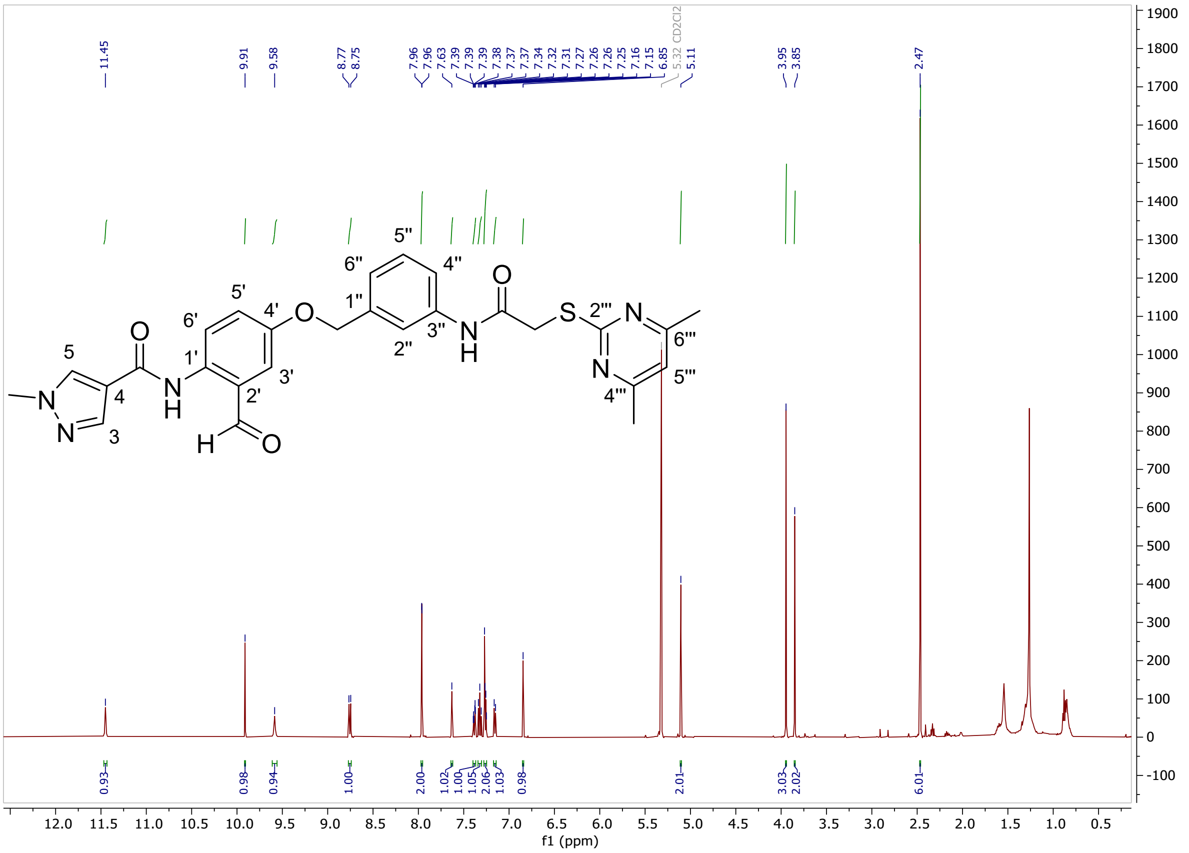

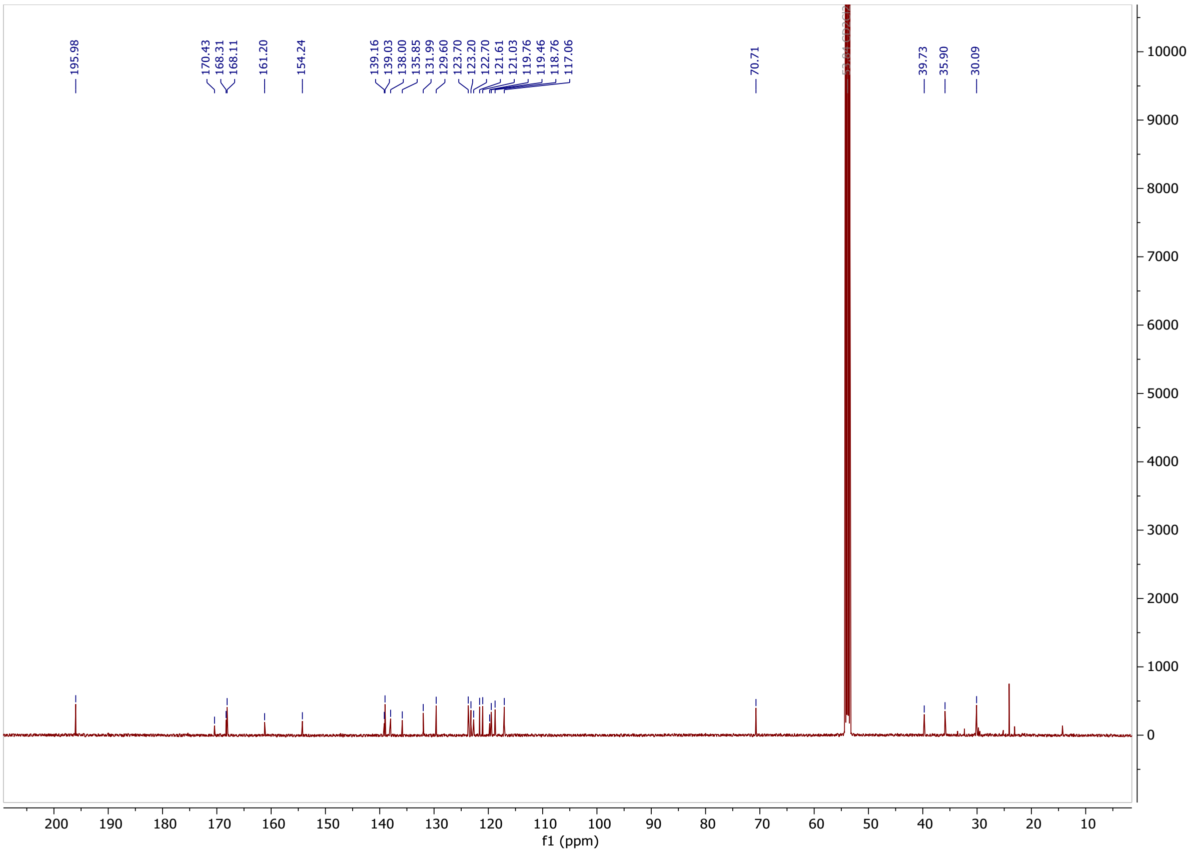

^{1}H and ^{13}C NMR spectra of compound **33**

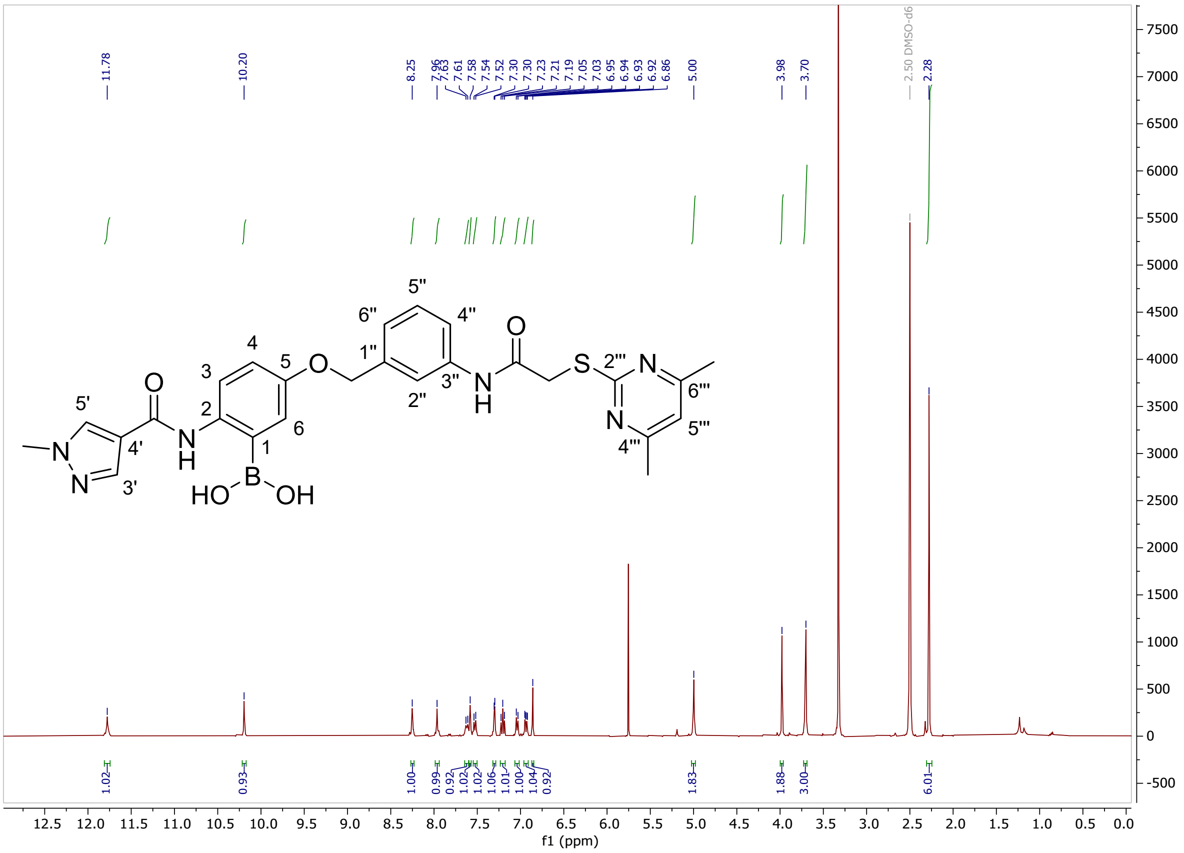

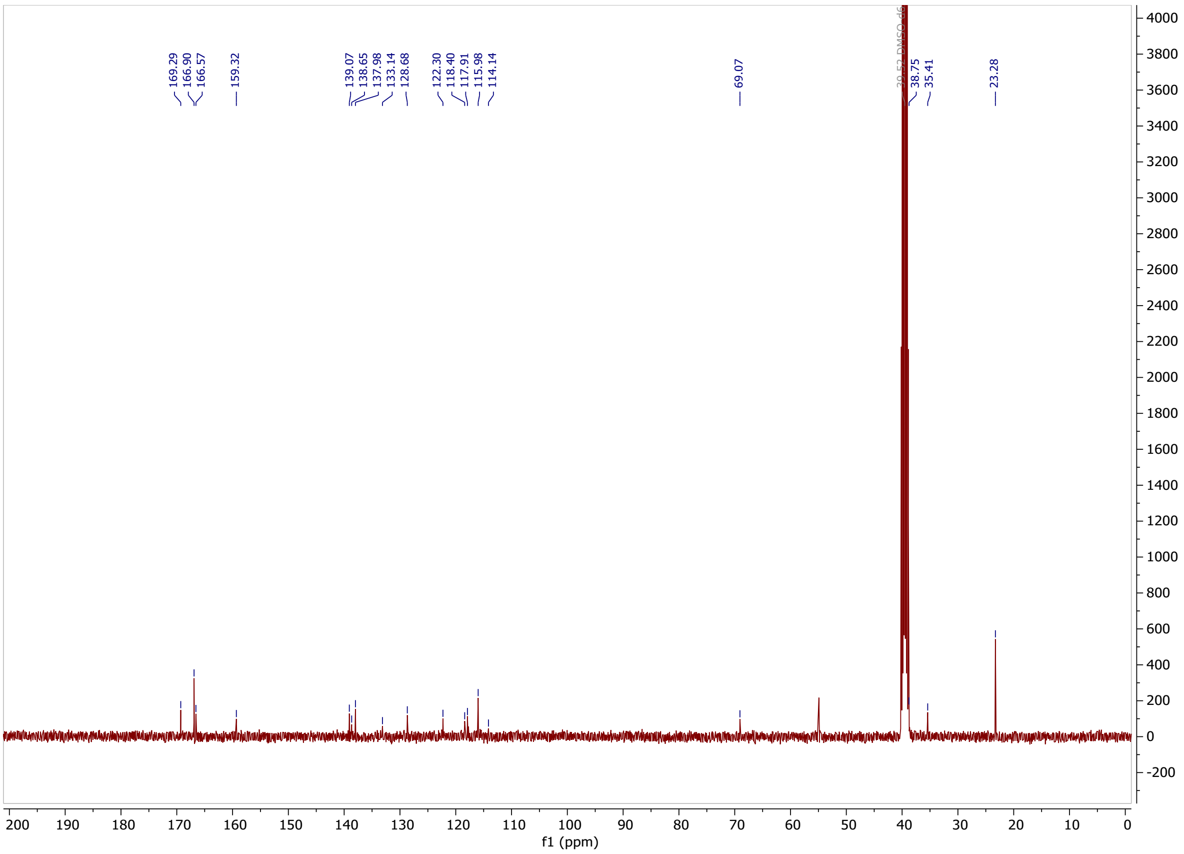

^{1}H and ^{13}C NMR spectra of compound **34**

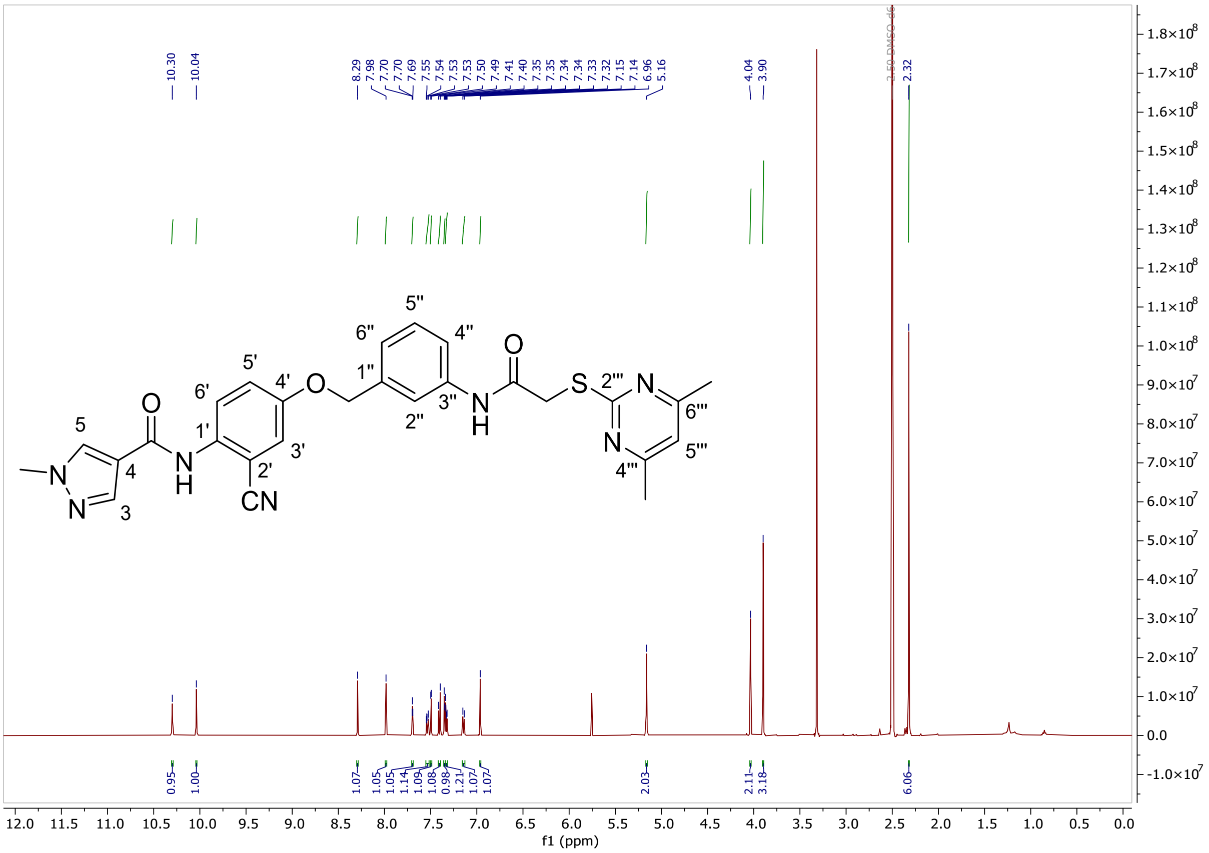

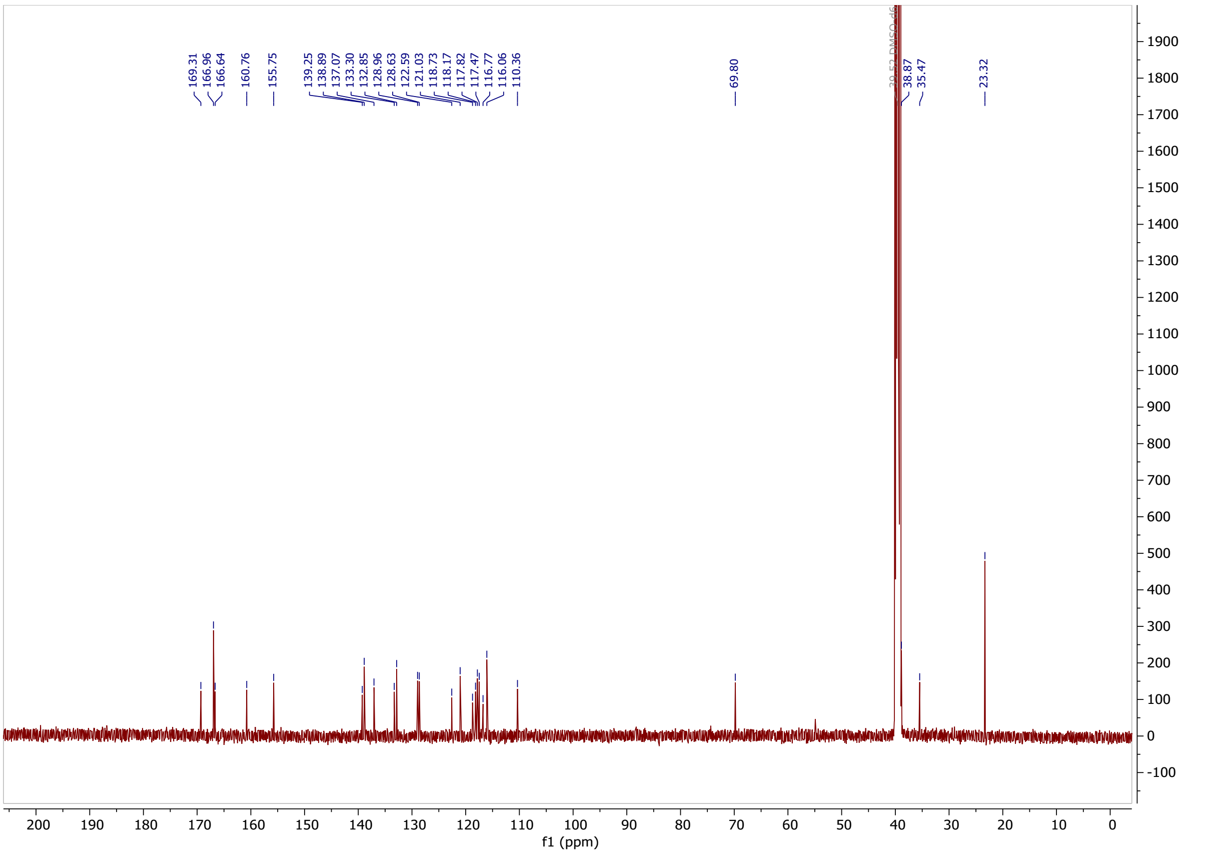

^{1}H and ^{13}C NMR spectra of compound **39**

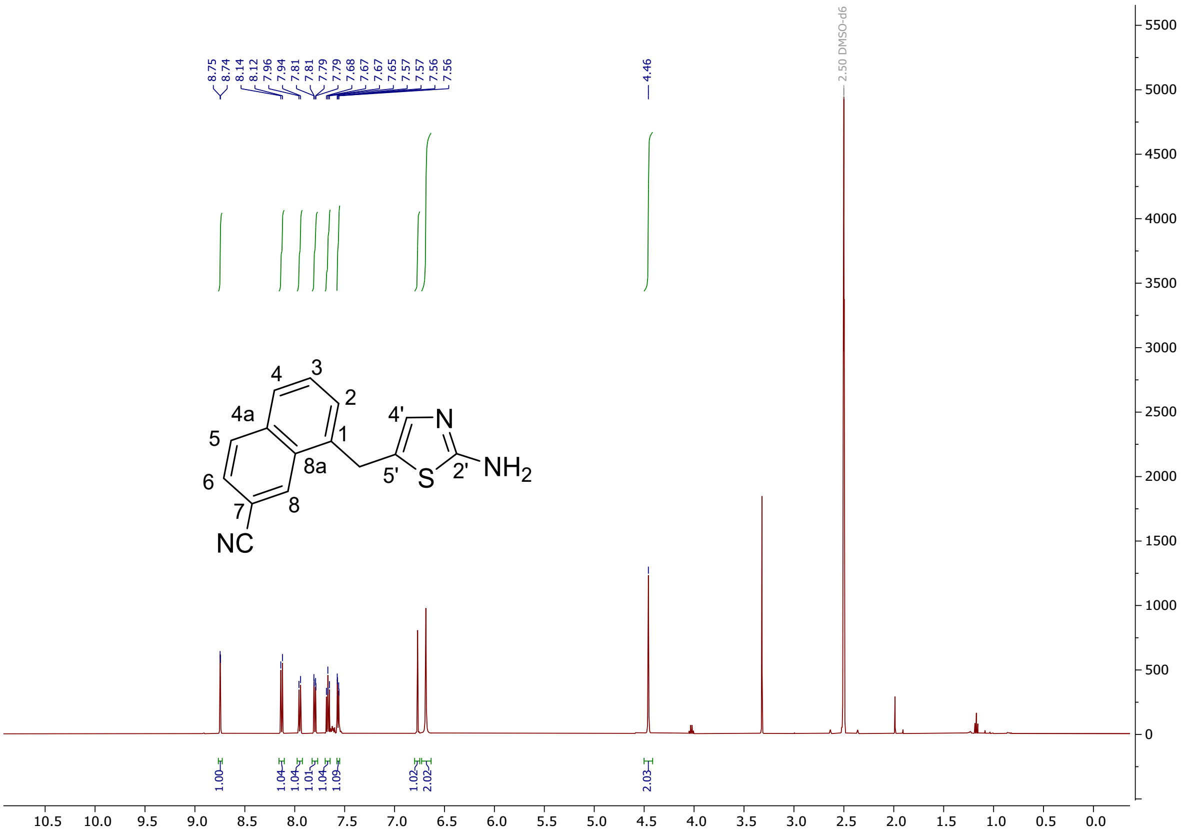

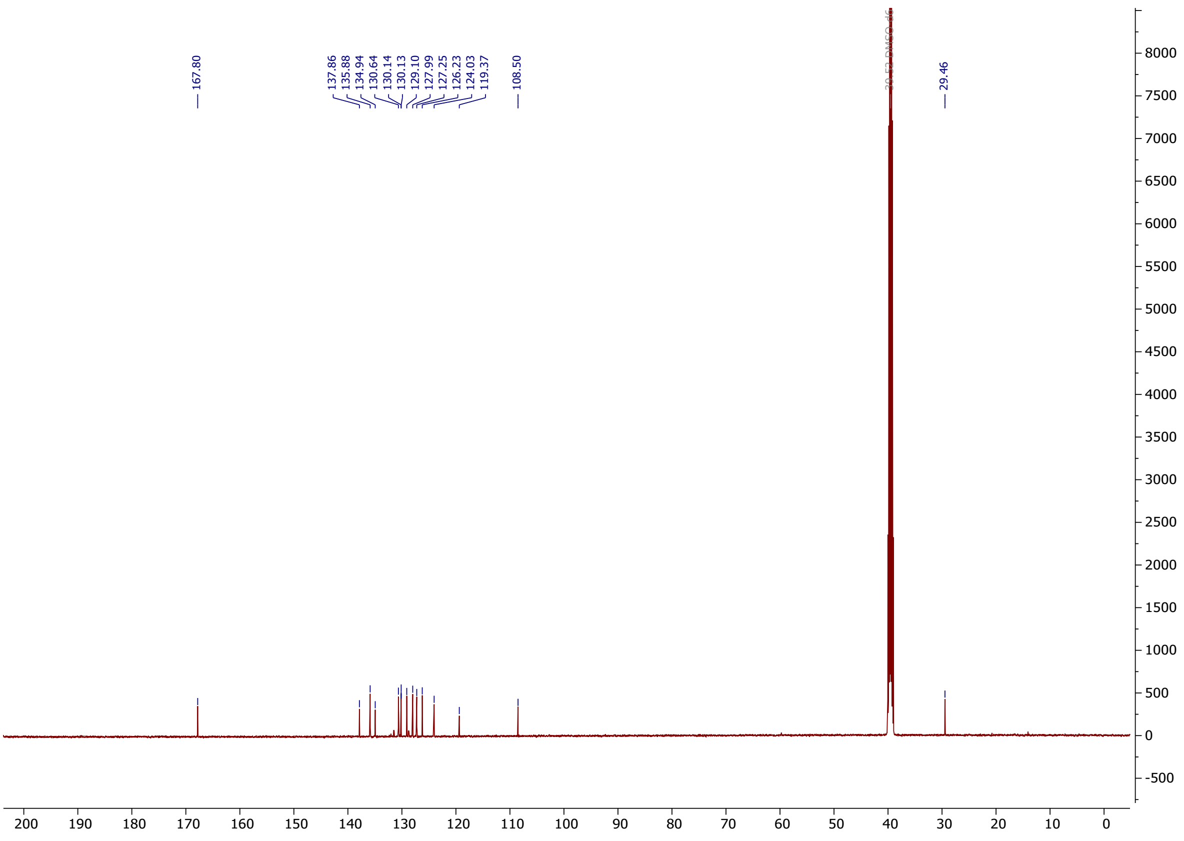

^{1}H and ^{13}C NMR spectra of compound **40**

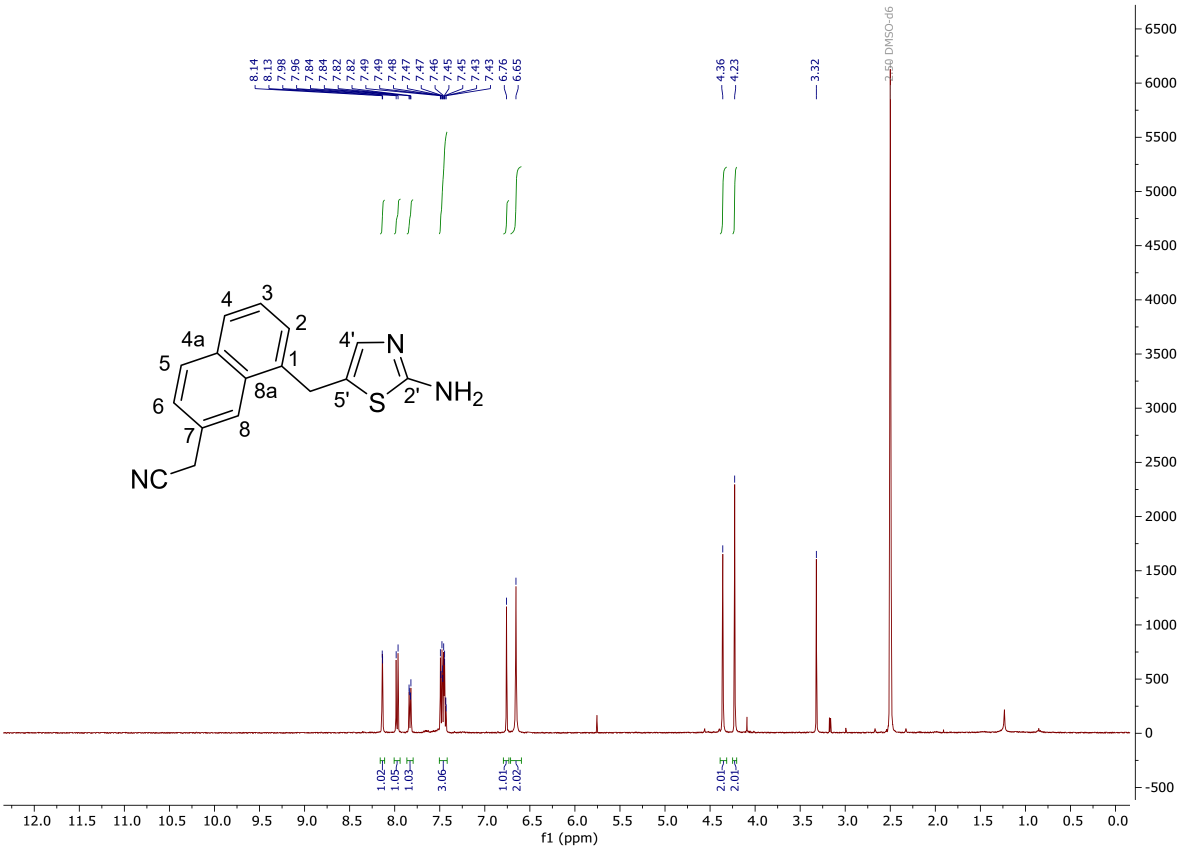

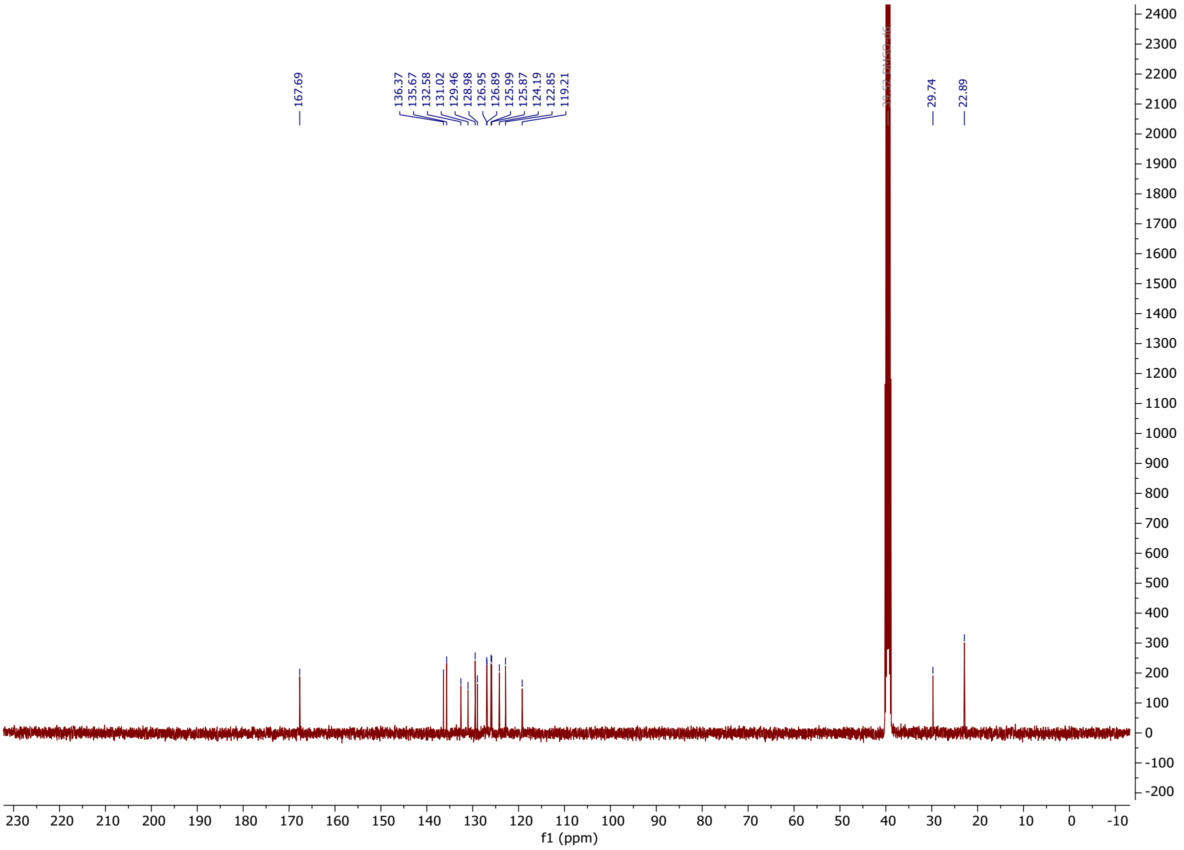

^{1}H and ^{13}C NMR spectra of compound **41**

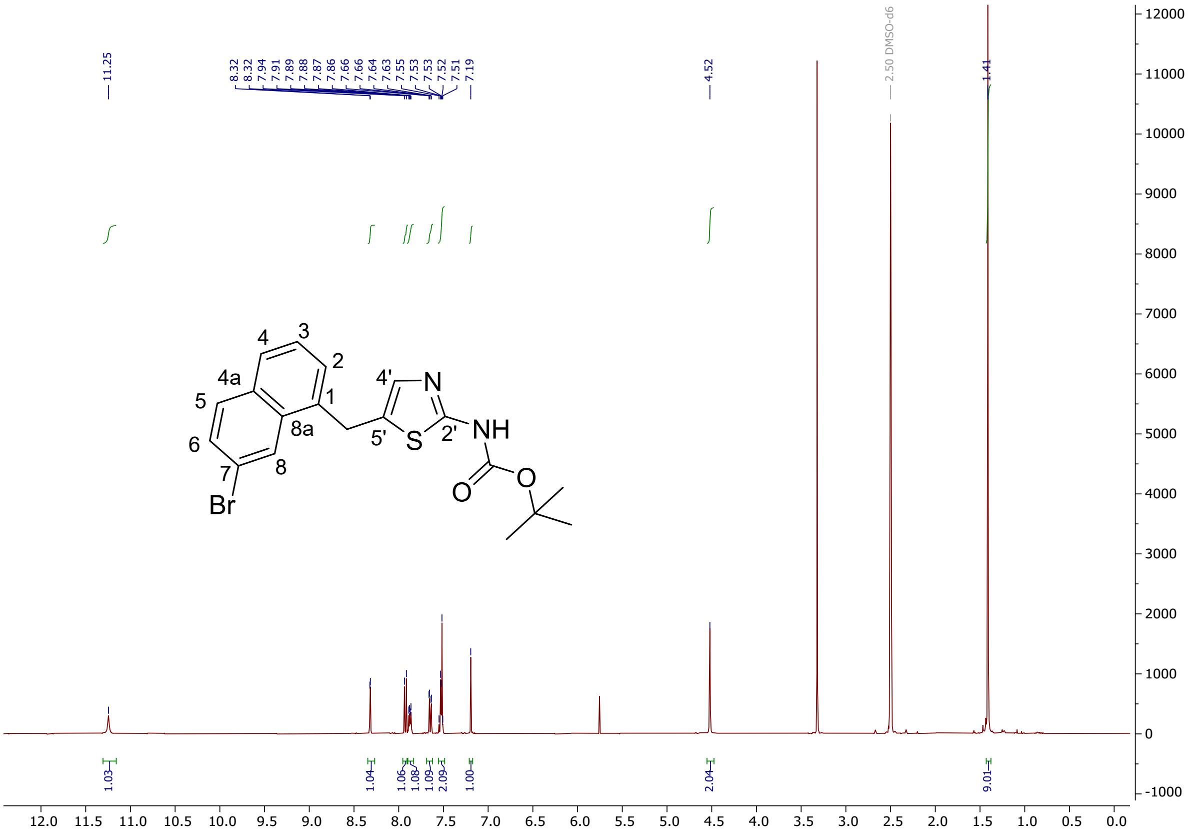

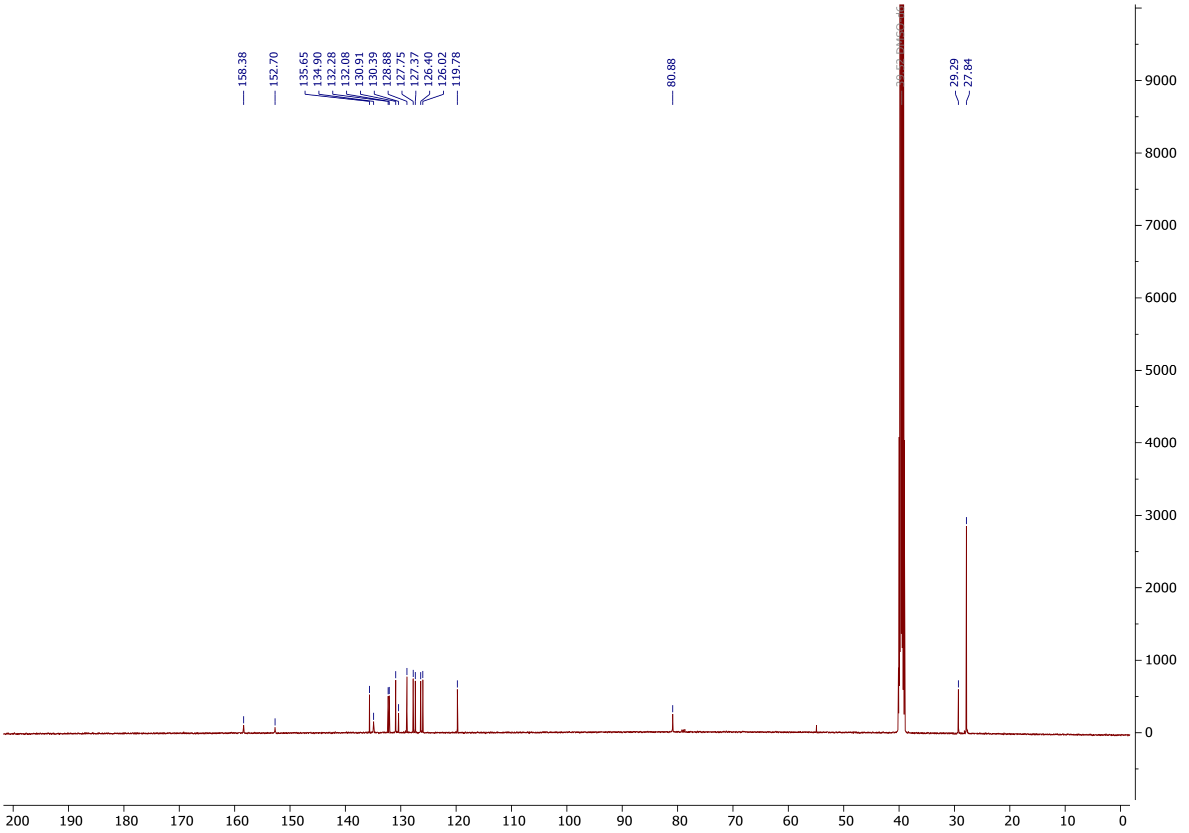

^{1}H and ^{13}C NMR spectra of compound **42**

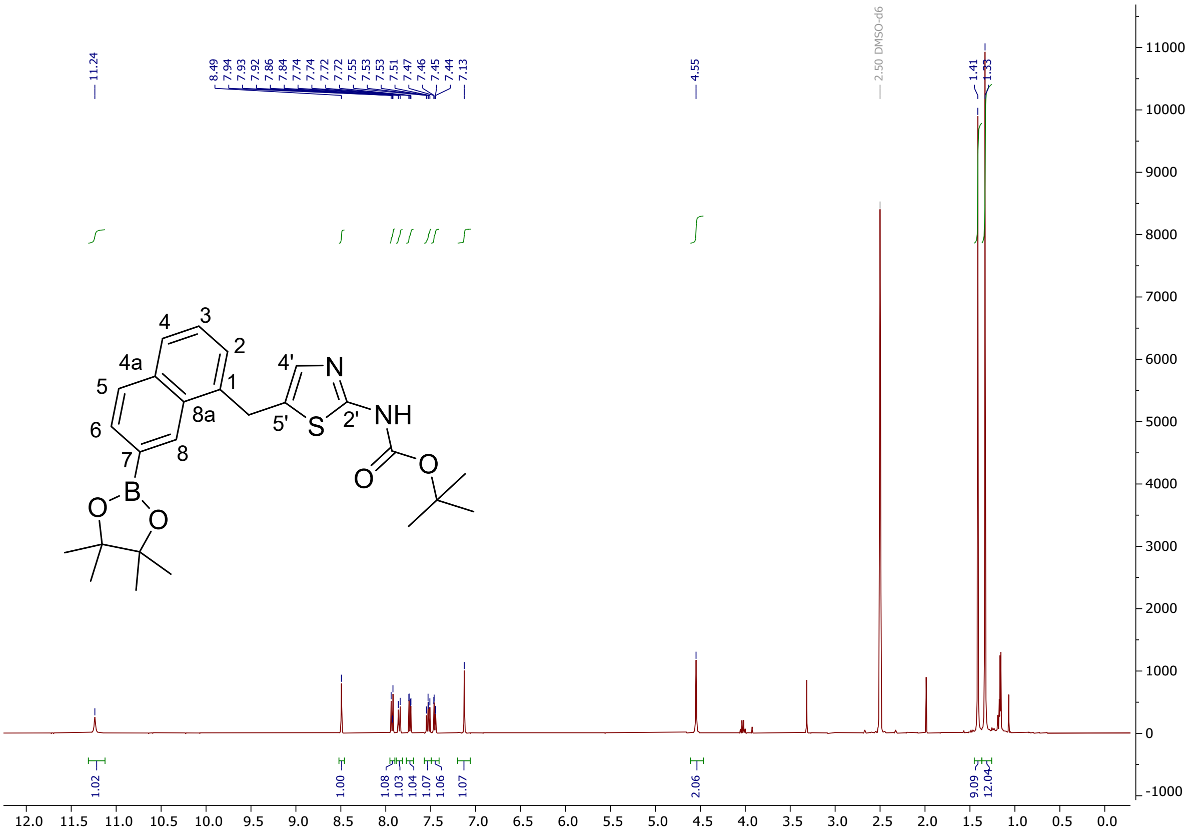

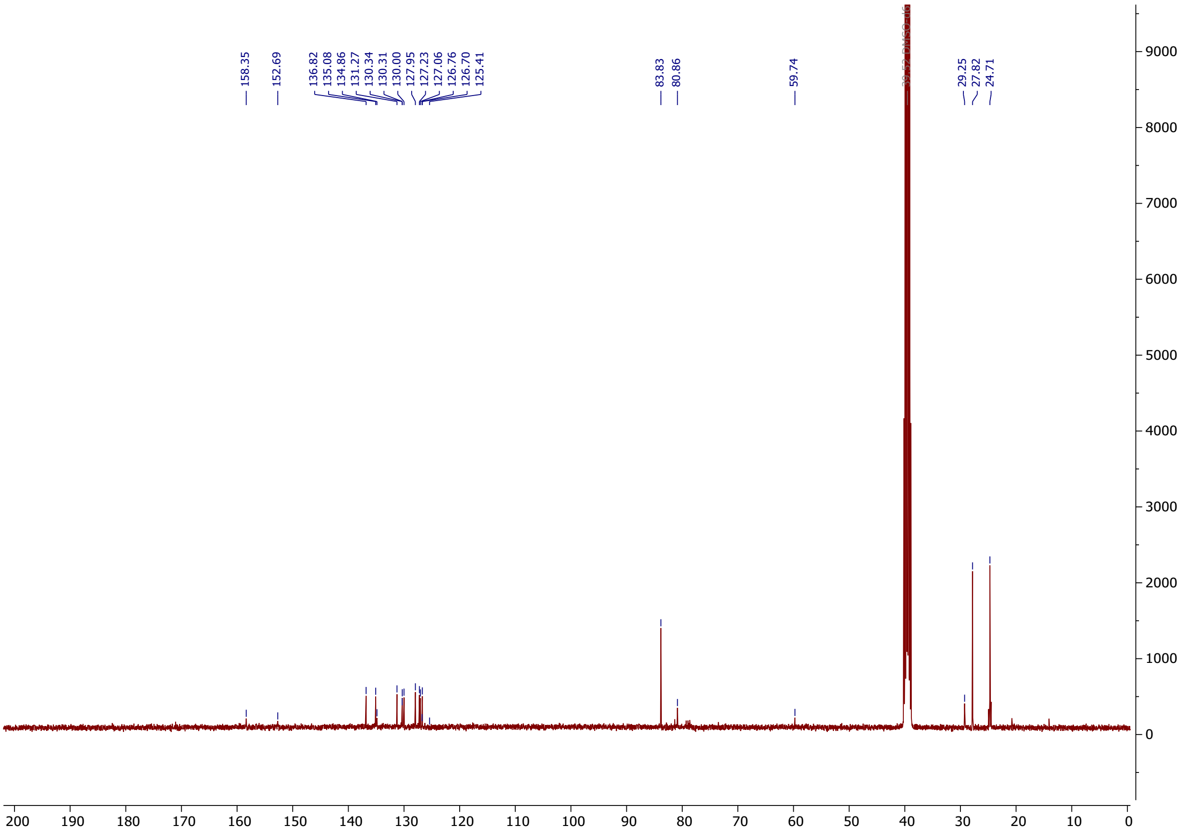

^{1}H and ^{13}C NMR spectra of compound **43**

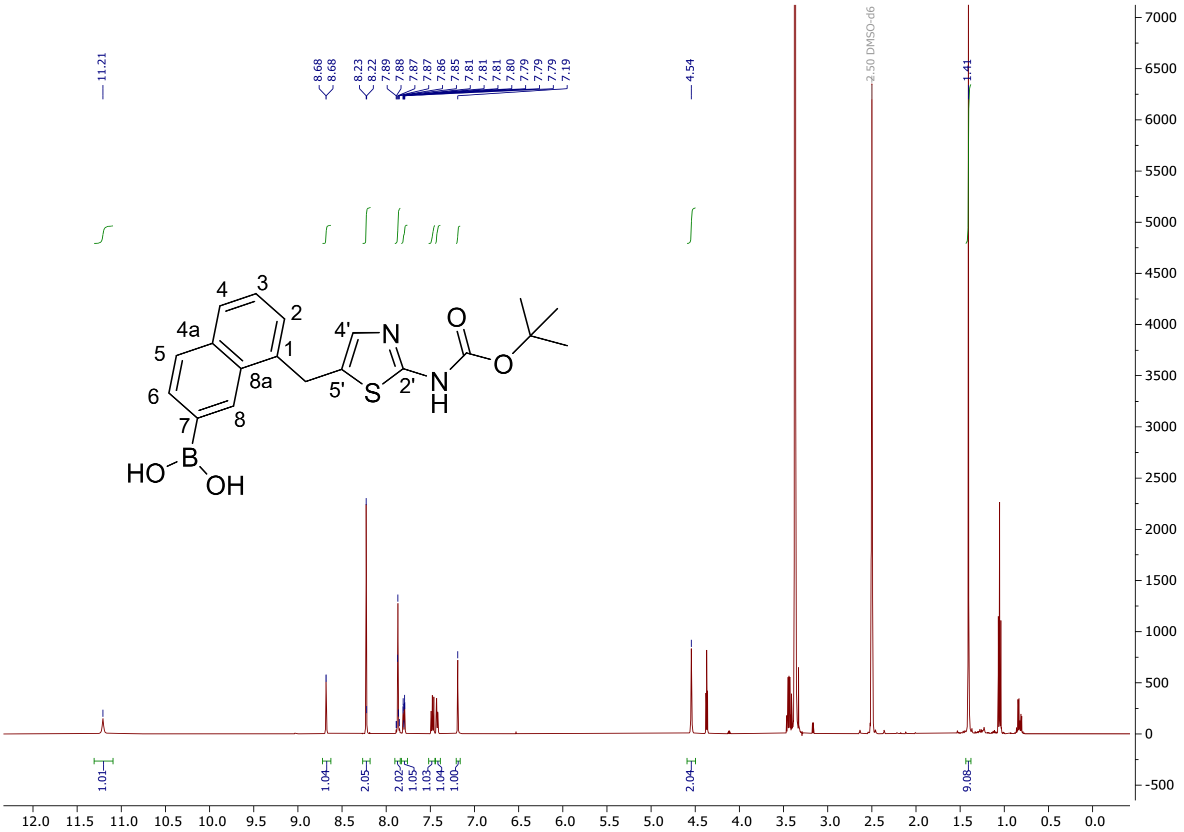

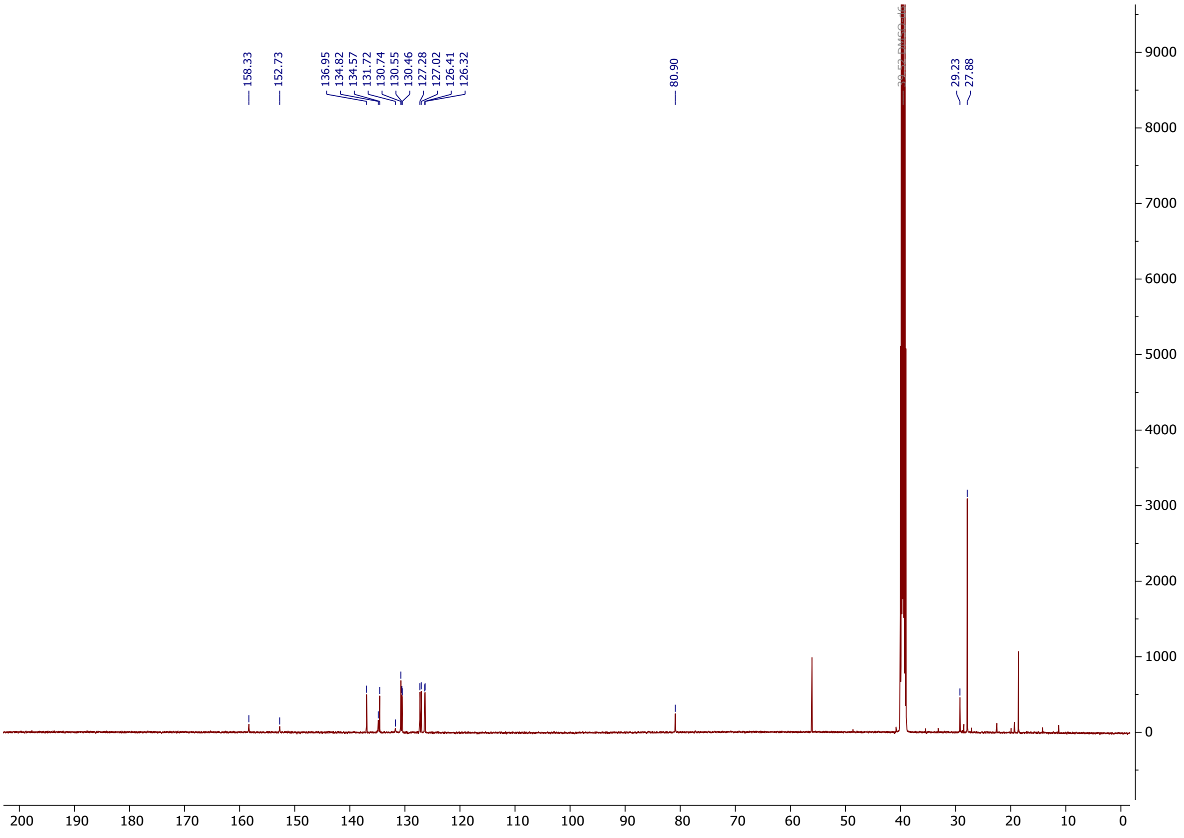

^1H and ^{13}C NMR spectra of compound **44**

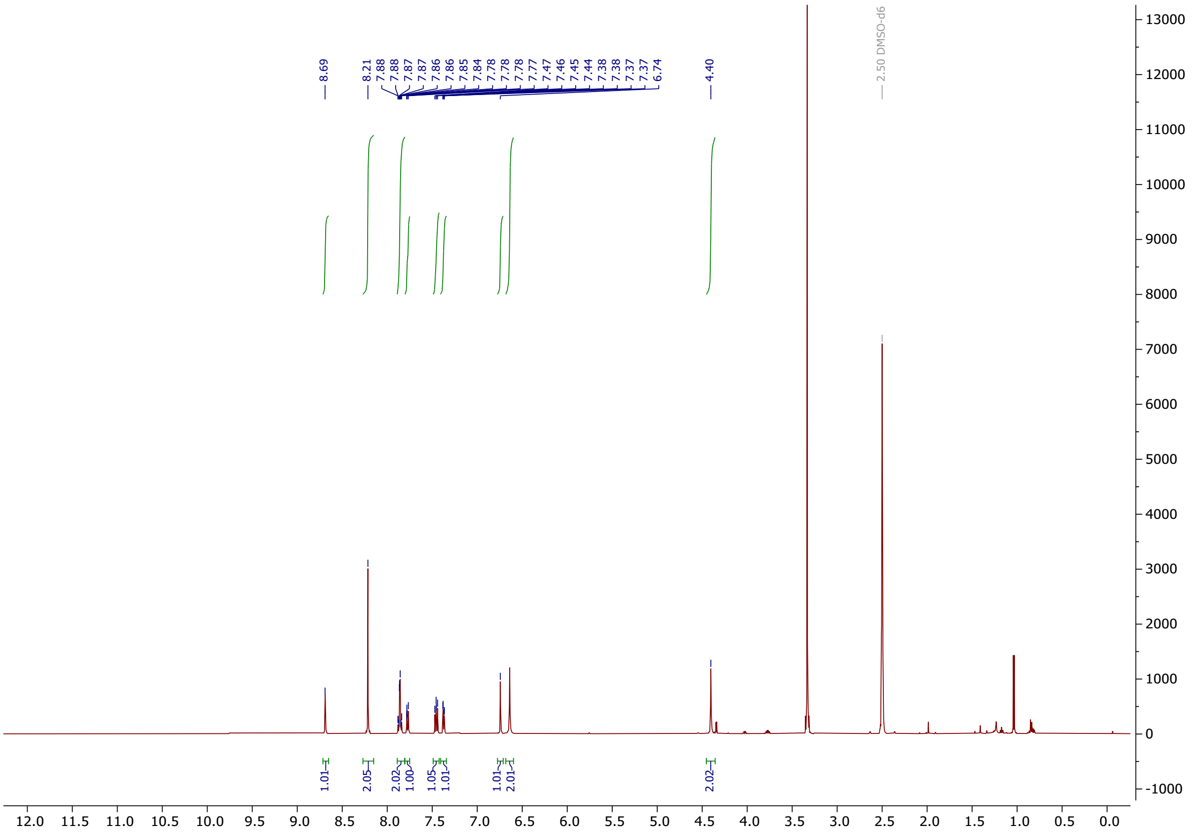

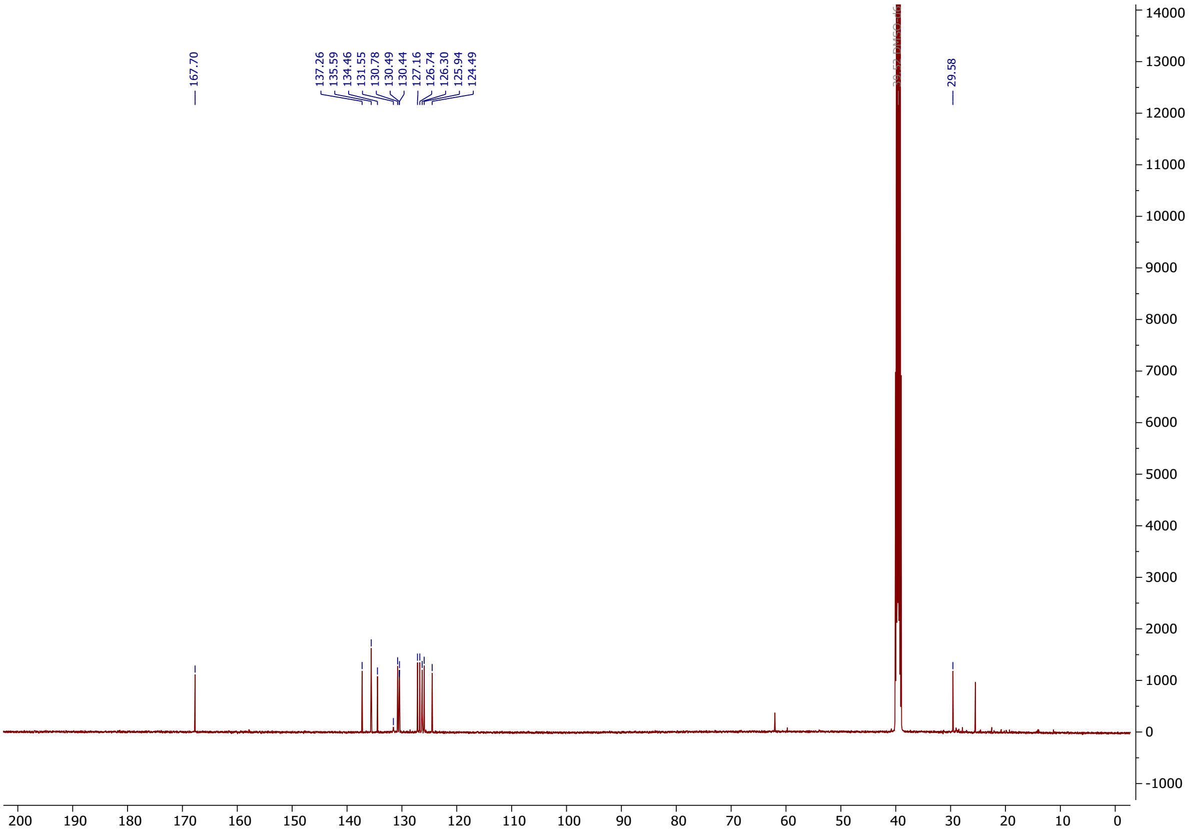

^{1}H and ^{13}C NMR spectra of compound **46**

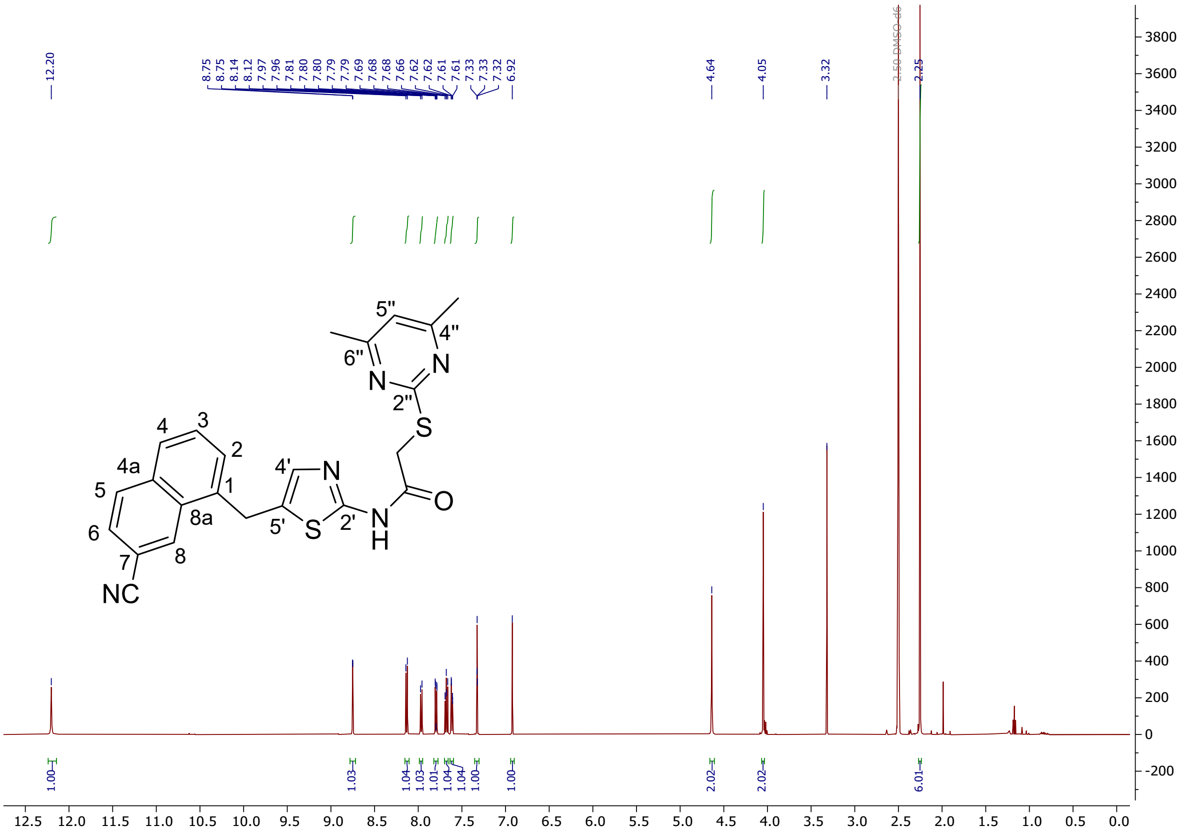

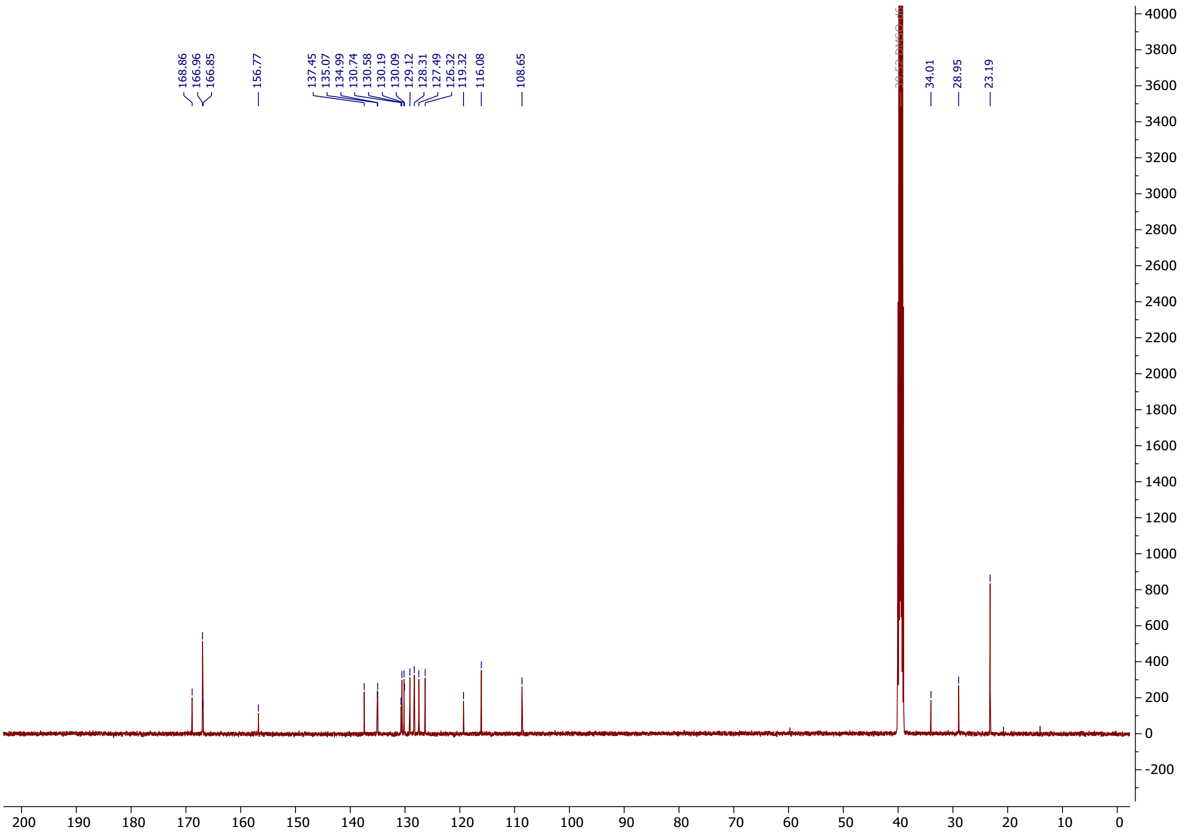

^{1}H and ^{13}C NMR spectra of compound **47**

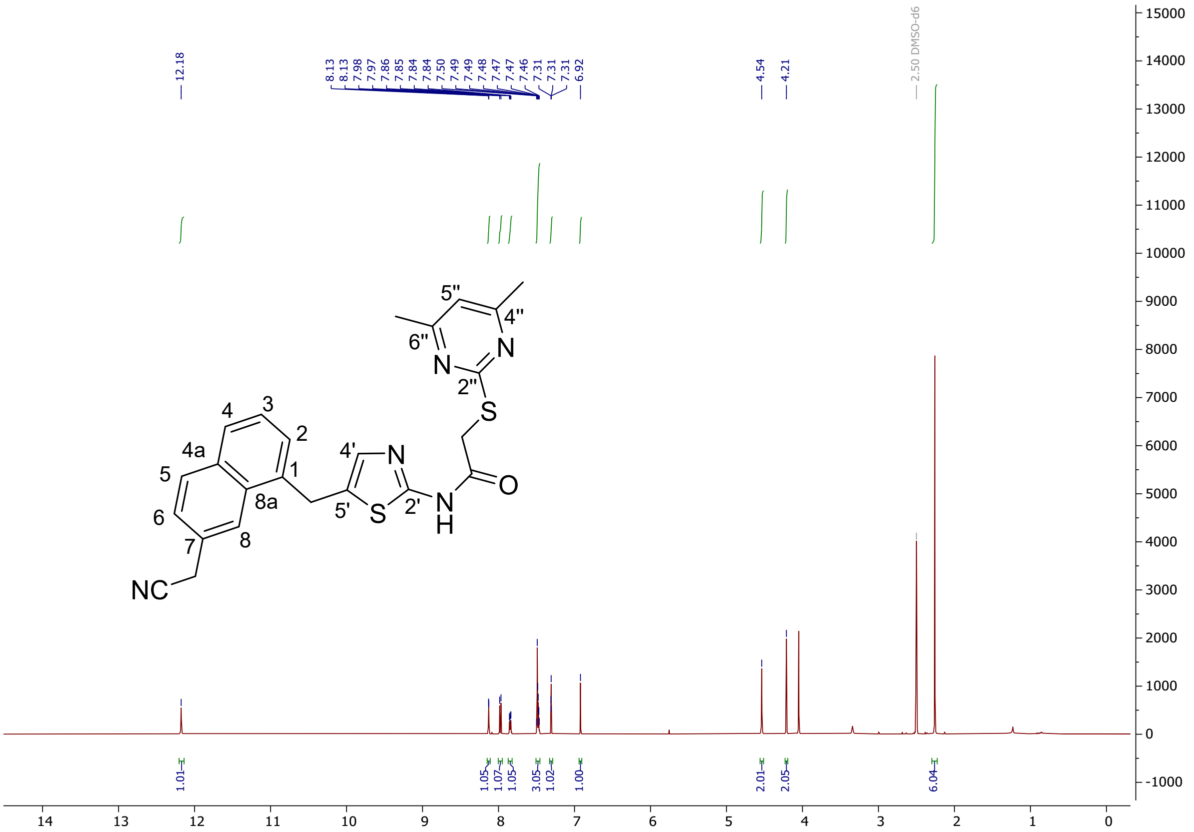

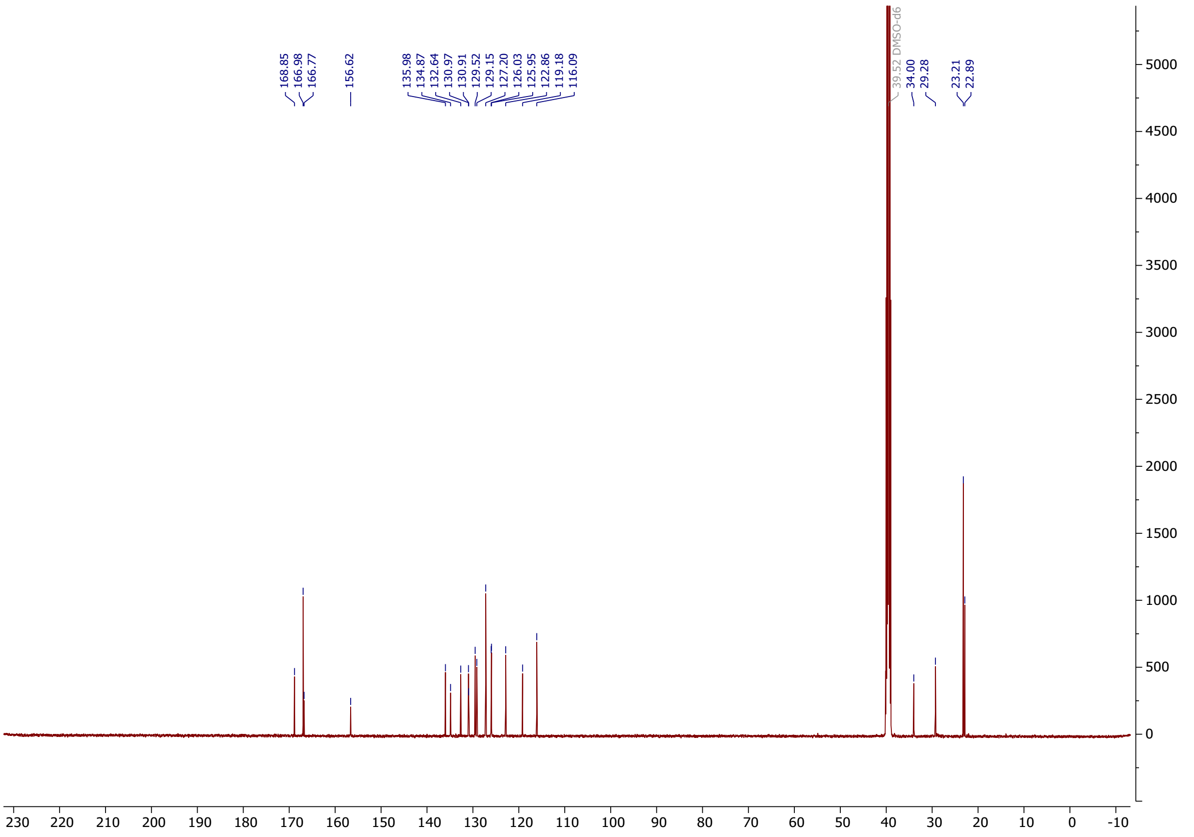

^{1}H and ^{13}C NMR spectra of compound **48**

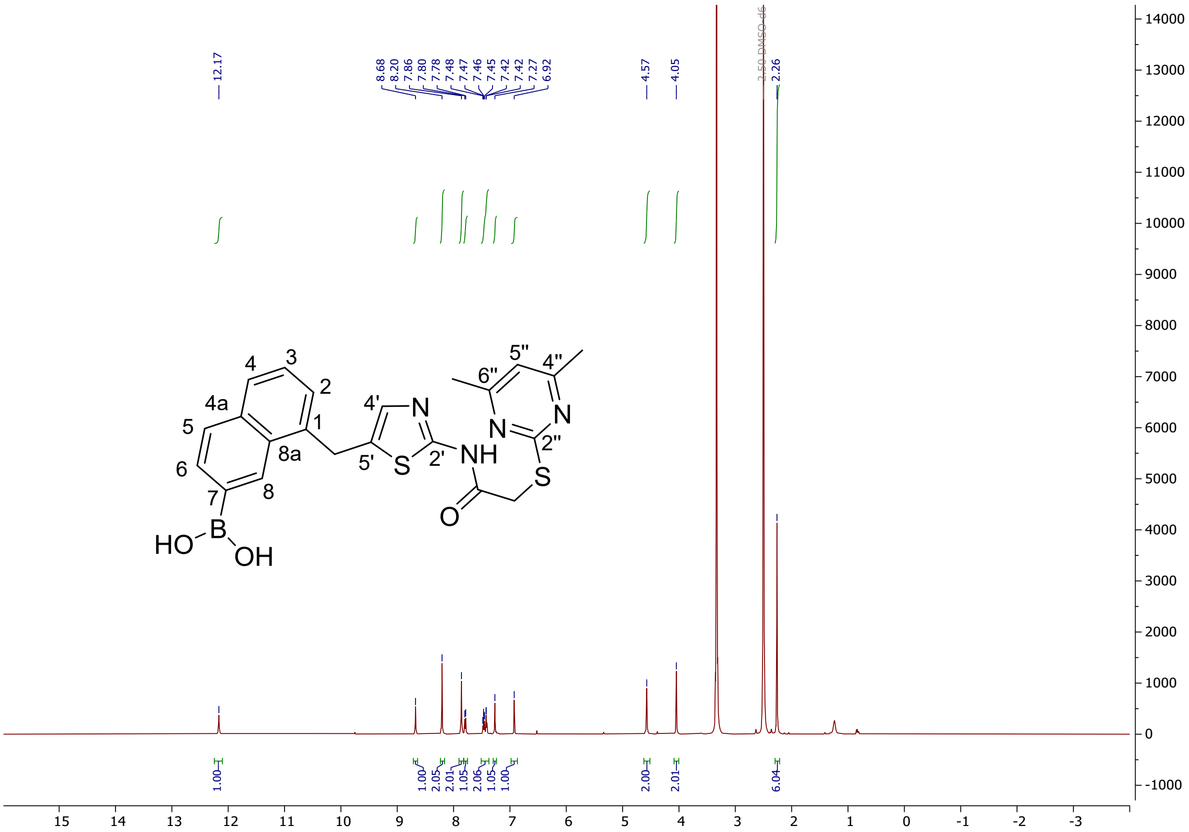

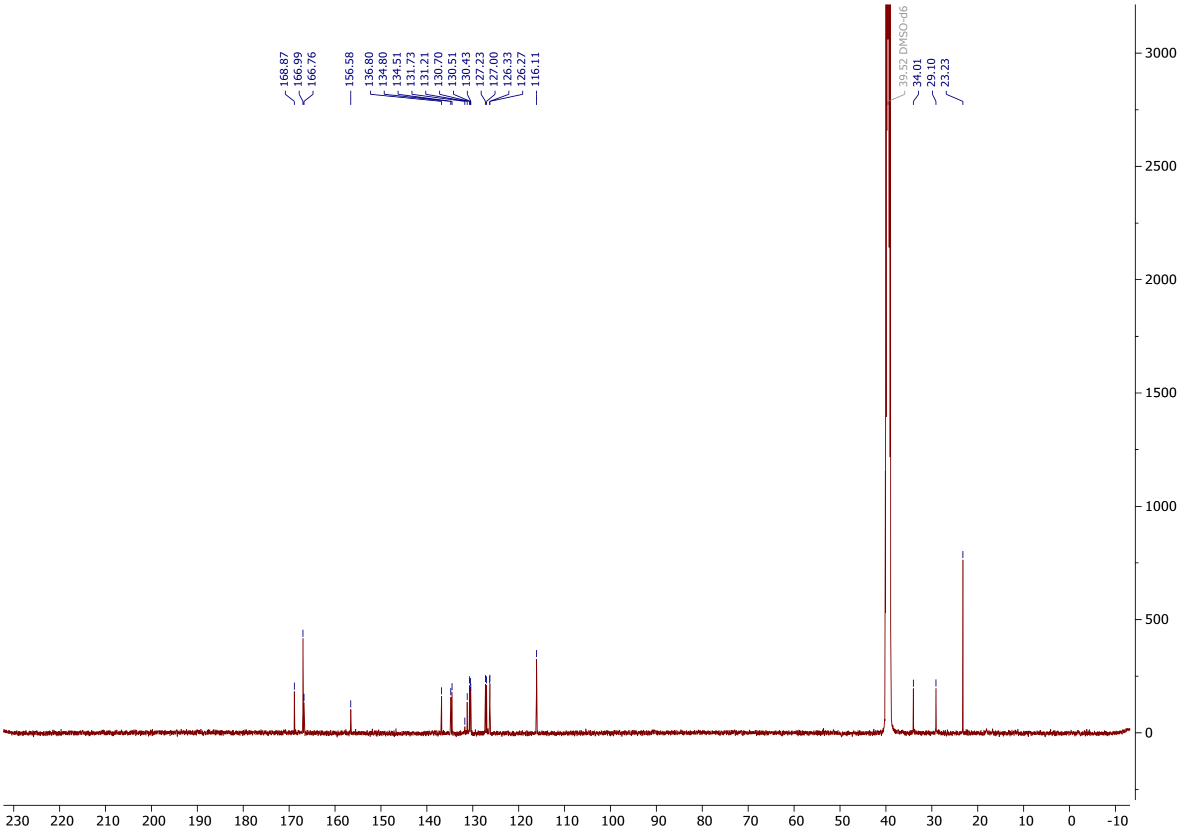

^1H and ^{13}C NMR spectra of compound **49**

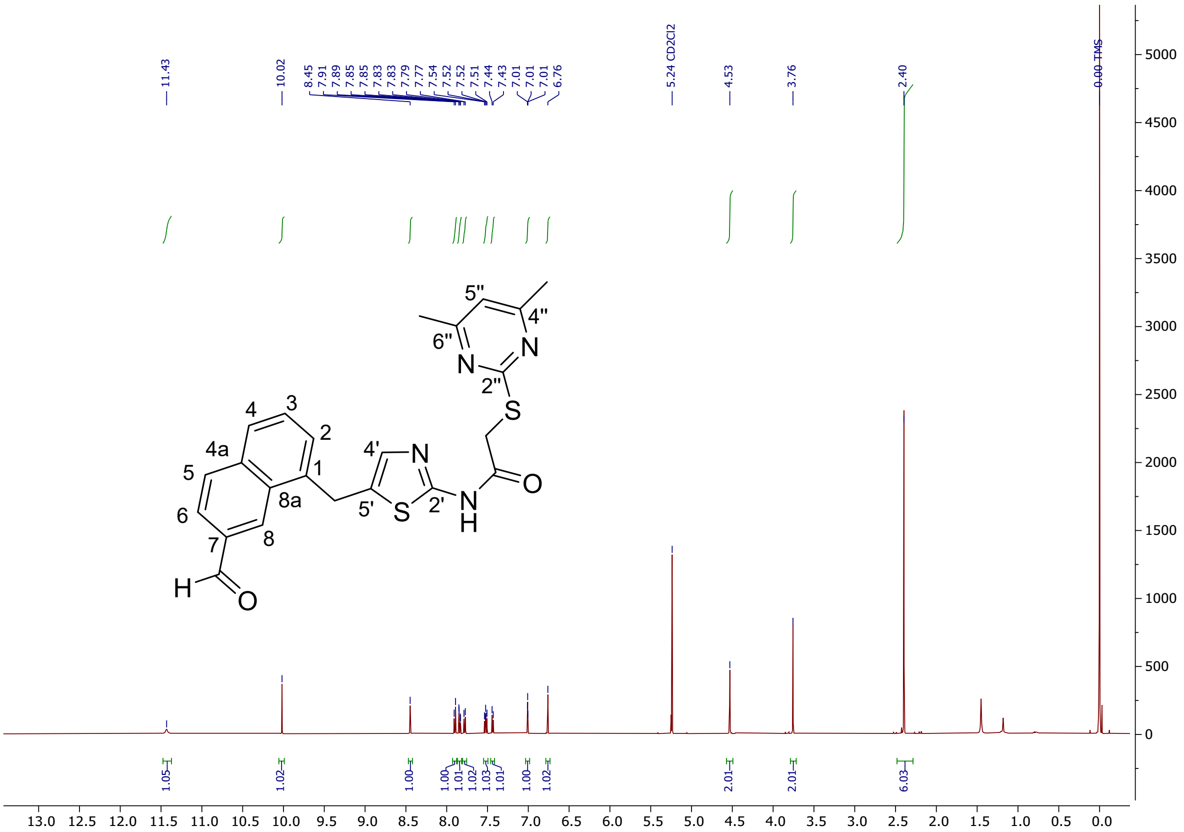

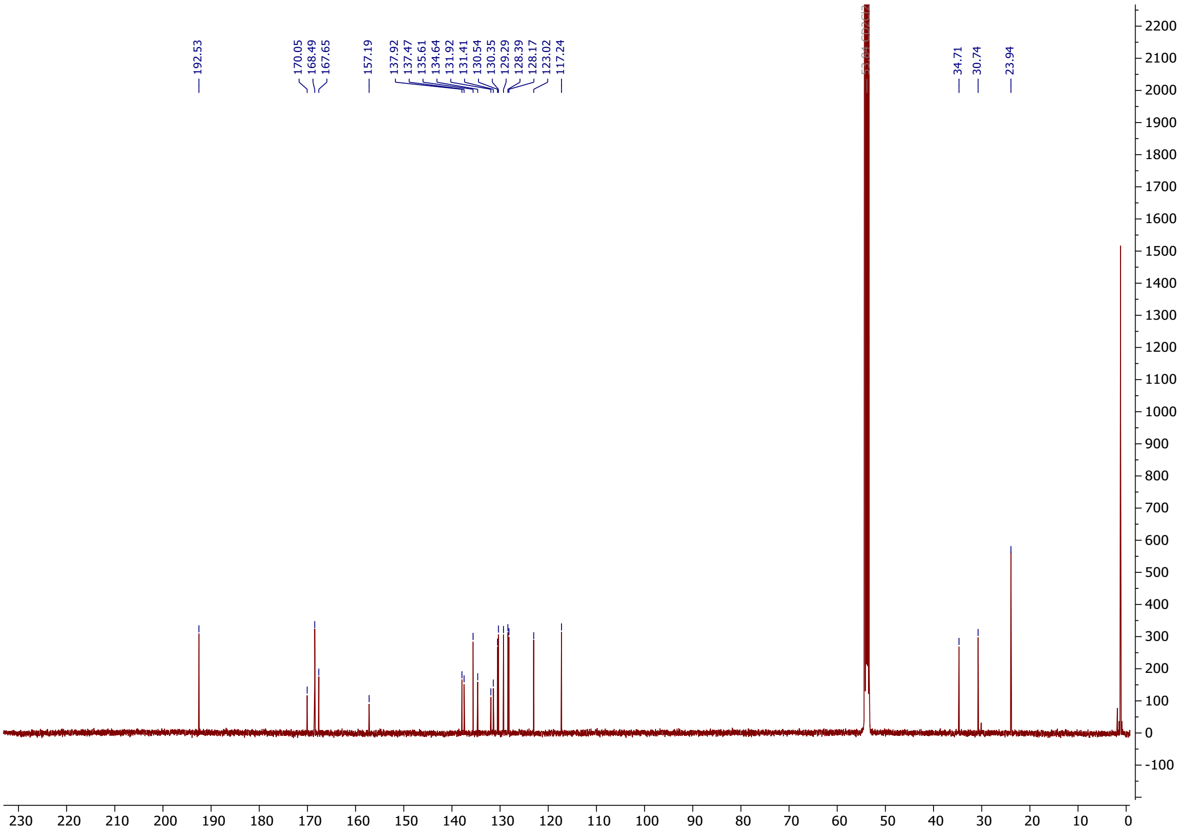

HPLC chromatogram of compound **11**

Chromatogram and Results

Injection Details

Injection Name:	**RW-50**	*Run Time (min):*	**24,99**
Vial Number:	**Vial:3**	*Injection Volume:*	**5,00**
Injection Type:	**Unknown**	*Wavelength A:*	**210**
Column:	**Zorbax SB C18 3,5µm 4,6x100mm 861953-902**	*Wavelength B:*	**254**
Instrument Method:	**35 AcN 65 Wasser**		
Processing Method:	**SG-094**	*Flow rate:*	**1,000** mL/min
Injection Date/Time:	**18.Jul.23 19:11**	*Column Temperatu*	**35,0** °C
Pump Channel A:	**35,00** AcN		
Pump Channel B:			
Pump ChannelC:			
Pump Channel D:	**65** Wasser		

Chromatogram

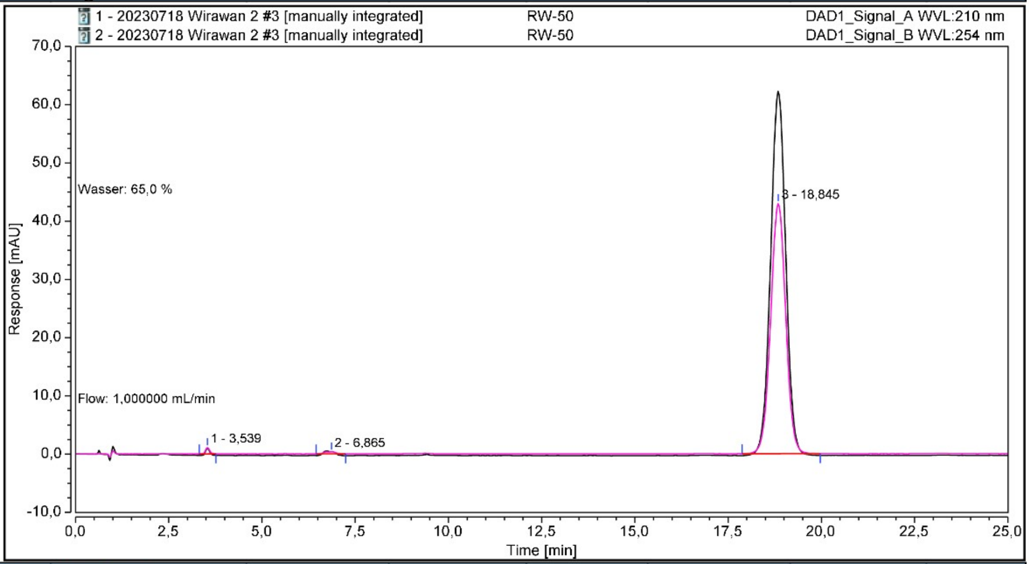

Integration Results

210nm

No.	Peak Name	Retention Time min	Area mAU*min	Relative Area %
1		3,539	0,161	0,54
2		6,725	0,247	0,82
3		18,845	29,514	98,64
**Total:**			**29,921**	**100,00**

254mn

No.	Peak Name	Retention Time min	Area mAU*min	Relative Area %
1		3,539	0,140	0,68
2		6,865	0,123	0,60
3		18,845	20,237	98,72
**Total:**			**20,499**	**100,00**

HPLC chromatogram of compound **12**

Chromatogram and Results

Injection Details

Injection Name:	**RW-57**	*Run Time (min):*	**24,99**
Vial Number:	**Vial:4**	*Injection Volume:*	**5,00**
Injection Type:	**Unknown**	*Wavelength A:*	**210**
Column:	**Zorbax SB C18 3,5µm 4,6x100mm 861953-902**	*Wavelength B:*	**254**
Instrument Method:	**35 AcN 65 Wasser**		
Processing Method:	**SG-094**	*Flow rate:*	**1,000** mL/min
Injection Date/Time:	**18.Jul.23 19:37**	*Column Temperatu*	**35,0** °C
Pump Channel A:	**35,00** AcN		
Pump Channel B:			
Pump ChannelC:			
Pump Channel D:	**65** Wasser		

Chromatogram

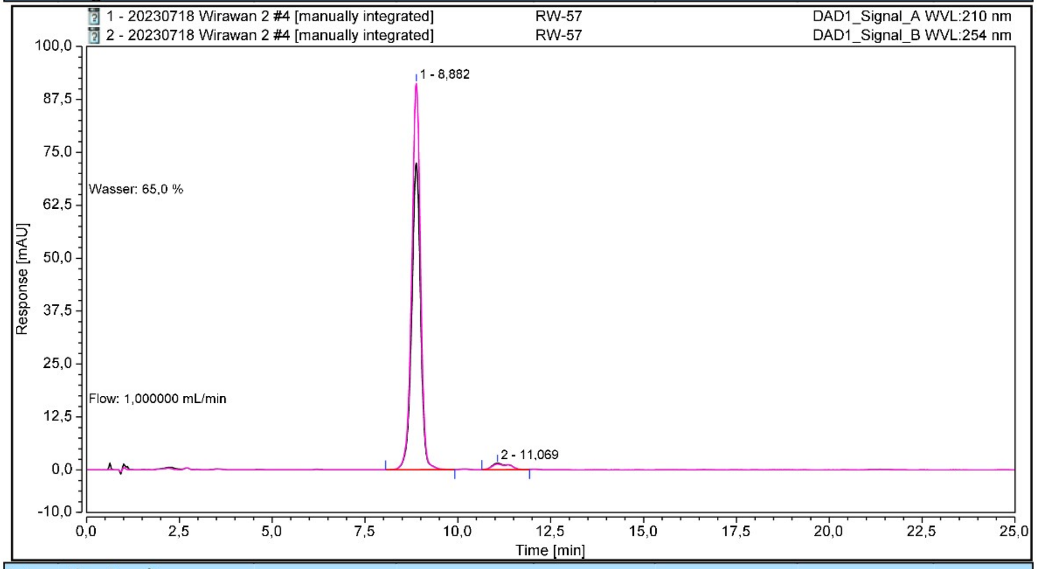

Integration Results

210nm

No.	Peak Name	Retention Time min	Area mAU*min	Relative Area %
1		8,882	20,044	96,45
2		11,069	0,738	3,55
**Total:**			**20,782**	**100,00**

254mn

No.	Peak Name	Retention Time min	Area mAU*min	Relative Area %
1		8,882	25,106	97,27
2		11,069	0,705	2,73
**Total:**			**25,811**	**100,00**

Chromeleon (c) Dionex
Version 7.2.9.11323

HPLC chromatogram of compound **13**

Chromatogram and Results

Injection Details

Injection Name:	**RW-22 in MeOH**	*Run Time (min):*	**15,31**
Vial Number:	**Vial:12**	*Injection Volume:*	**5,00**
Injection Type:	**Unknown**	*Wavelength A:*	**210**
Column:	**Zorbax SB C18 3,5µm 4,6x100mm 861953-902**	*Wavelength B:*	**254**
Instrument Method:	**35 AcN 65 Wasser**		
Processing Method:	**SG-094**	*Flow rate:*	**1,000** mL/min
Injection Date/Time:	**19.Jul.23 09:00**	*Column Temperatu*	**35,0** °C
Pump Channel A:	**35,00** AcN		
Pump Channel B:			
Pump ChannelC:			
Pump Channel D:	**65** Wasser		

Chromatogram

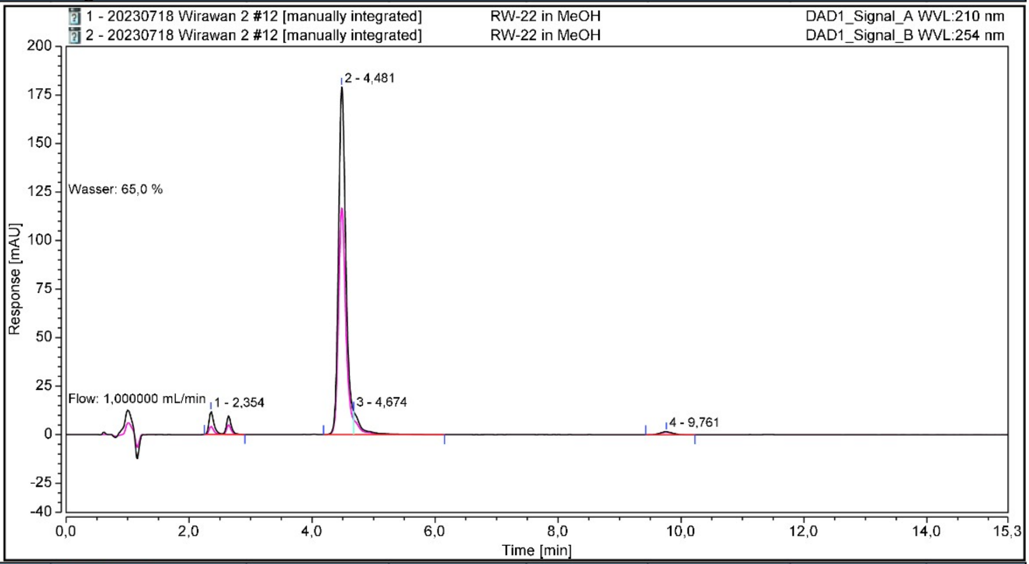

Integration Results

210nm

No.	Peak Name	Retention Time min	Area mAU*min	Relative Area %
1		2,354	1,944	6,76
2		4,481	24,817	86,26
3		4,674	1,652	5,74
4		9,761	0,356	1,24
**Total:**			**28,769**	**100,00**

254mn

No.	Peak Name	Retention Time min	Area mAU*min	Relative Area %
1		2,354	0,359	2,00
3		4,481	16,320	90,58
4		4,694	0,924	5,13
n.a.	n.a.	n.a.	n.a.	n.a.
**Total:**			**17,604**	**97,70**

HPLC chromatogram of compound **14**

Chromatogram and Results

Injection Details

Injection Name:	**RW-41**	*Run Time (min):*	**9,99**
Vial Number:	**Vial:3**	*Injection Volume:*	**5,00**
Injection Type:	**Unknown**	*Wavelength A:*	**210**
Column:	**Zorbax SB C18 3,5µm 4,6x100mm 861953-902**	*Wavelength B:*	**254**
Instrument Method:	**35 AcN 65 Wasser**		
Processing Method:	**SG-094**	*Flow rate:*	**1,000 mL/min**
Injection Date/Time:	**23.Nov.23 14:52**	*Column Temperatu*	**35,0 °C**
Pump Channel A:	**35,00** AcN		
Pump Channel B:			
Pump ChannelC:			
Pump Channel D:	**65** Wasser		

Chromatogram

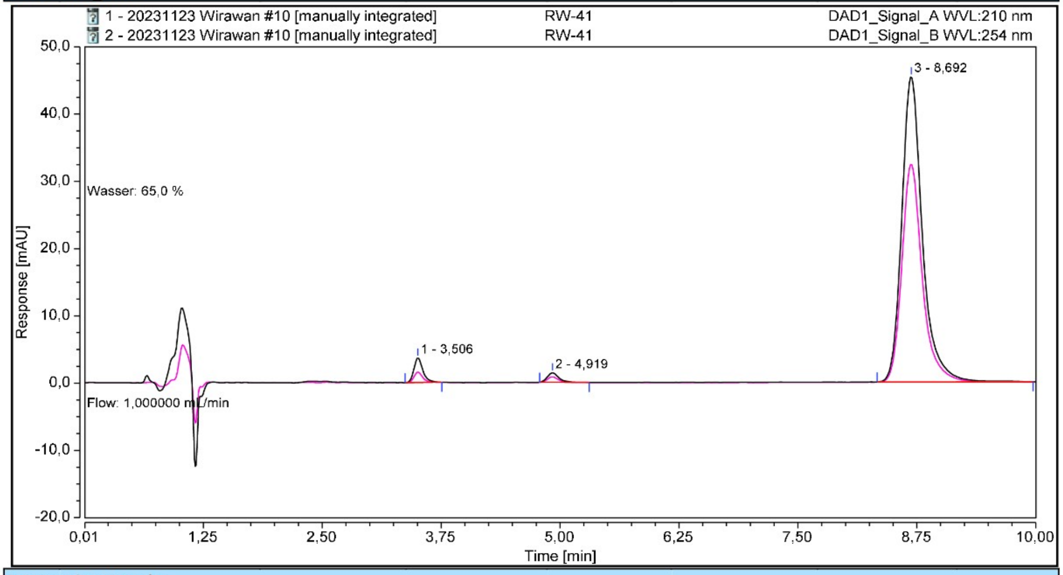

Integration Results

210nm

No.	Peak Name	Retention Time min	Area mAU*min	Relative Area %
1		3,506	0,401	3,34
2		4,919	0,198	1,65
3		8,692	11,417	95,02
**Total:**			**12,016**	**100,00**

254mn

No.	Peak Name	Retention Time min	Area mAU*min	Relative Area %
1		3,506	0,168	2,01
2		4,919	0,127	1,52
3		8,692	8,068	96,47
**Total:**			**8,363**	**100,00**

Chromeleon (c) Dionex
Version 7.2.9.11323

HPLC chromatogram of compound **29**

Chromatogram and Results

Injection Details

Injection Name:	**RW-78**	*Run Time (min):*	**9,99**
Vial Number:	**Vial:4**	*Injection Volume:*	**5,00**
Injection Type:	**Unknown**	*Wavelength A:*	**210**
Column:	**Zorbax SB C18 3,5µm 4,6x100mm 861953-902**	*Wavelength B:*	**254**
Instrument Method:	**35 AcN 65 Wasser**		
Processing Method:	**SG-094**	*Flow rate:*	**1,000** mL/min
Injection Date/Time:	**23.Nov.23 15:02**	*Column Temperatu*	**35,0** °C
Pump Channel A:	**35,00** AcN		
Pump Channel B:			
Pump ChannelC:			
Pump Channel D:	**65** Wasser		

Chromatogram

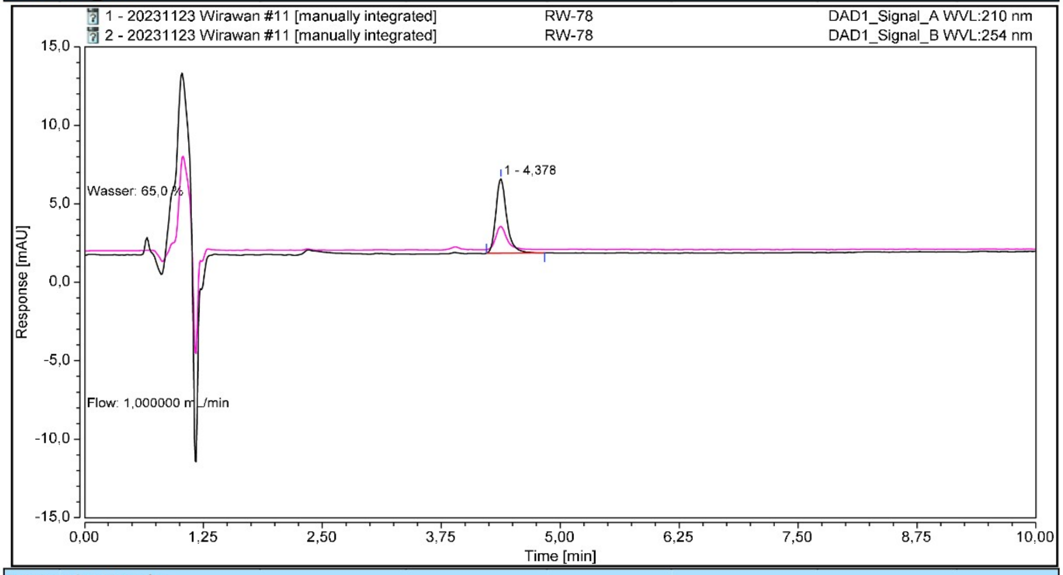

Integration Results

210nm

No.	Peak Name	Retention Time min	Area mAU*min	Relative Area %
1		4,378	0,626	100,00
**Total:**			**0,626**	**100,00**

254mn

No.	Peak Name	Retention Time min	Area mAU*min	Relative Area %
2		4,378	0,193	89,04
**Total:**			**0,193**	**89,04**

HPLC chromatogram of compound **30**

Chromatogram and Results

Injection Details

Injection Name:	**RW-63**	*Run Time (min):*	**24,99**
Vial Number:	**Vial:6**	*Injection Volume:*	**5,00**
Injection Type:	**Unknown**	*Wavelength A:*	**210**
Column:	**Zorbax SB C18 3,5µm 4,6x100mm 861953-902**	*Wavelength B:*	**254**
Instrument Method:	**35 AcN 65 Wasser**		
Processing Method:	**SG-094**	*Flow rate:*	**1,000** mL/min
Injection Date/Time:	**18.Jul.23 20:28**	*Column Temperatu*	**35,0** °C
Pump Channel A:	**35,00** AcN		
Pump Channel B:			
Pump ChannelC:			
Pump Channel D:	**65** Wasser		

Chromatogram

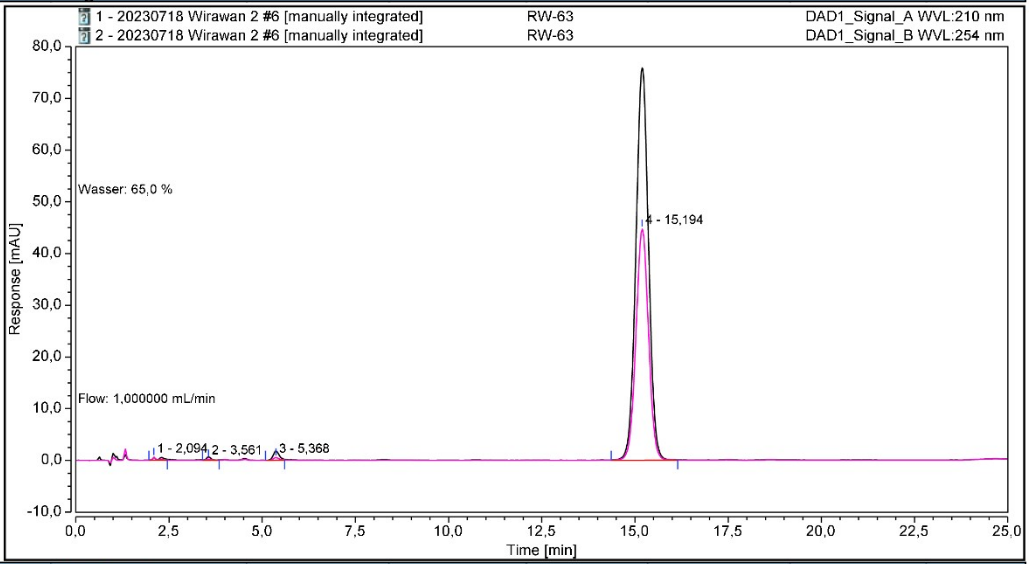

Integration Results

210nm

No.	Peak Name	Retention Time min	Area mAU*min	Relative Area %
n.a.	n.a.	n.a.	n.a.	n.a.
1		3,561	0,066	0,22
2		5,368	0,323	1,07
3		15,194	29,702	98,71
**Total:**			**30,091**	**100,00**

254mn

No.	Peak Name	Retention Time min	Area mAU*min	Relative Area %
1		2,094	0,061	0,35
2		3,561	0,025	0,14
3		5,368	0,084	0,48
4		15,194	17,534	99,04
**Total:**			**17,704**	**100,00**

HPLC chromatogram of compound **31**

Chromatogram and Results

Injection Details

Injection Name:	**RW-80**		*Run Time (min):*	**14,99**	
Vial Number:	**Vial:5**		*Injection Volume:*	**5,00**	
Injection Type:	**Unknown**		*Wavelength A:*	**210**	
Column:	**Zorbax SB C18 3,5µm 4,6x100mm 861953-902**		*Wavelength B:*	**254**	
Instrument Method:	**50 Acetonitril 50 Wasser Honey**				
Processing Method:	**SG-094**		*Flow rate:*	**1,200**	**mL/min**
Injection Date/Time:	**24.Nov.23 09:31**		*Column Temperatu*	**50,0**	**°C**
Pump Channel A:	**50,00**	Acetonitril			
Pump Channel B:		Methanol			
Pump ChannelC:					
Pump Channel D:	**50**	Wasser			

Chromatogram

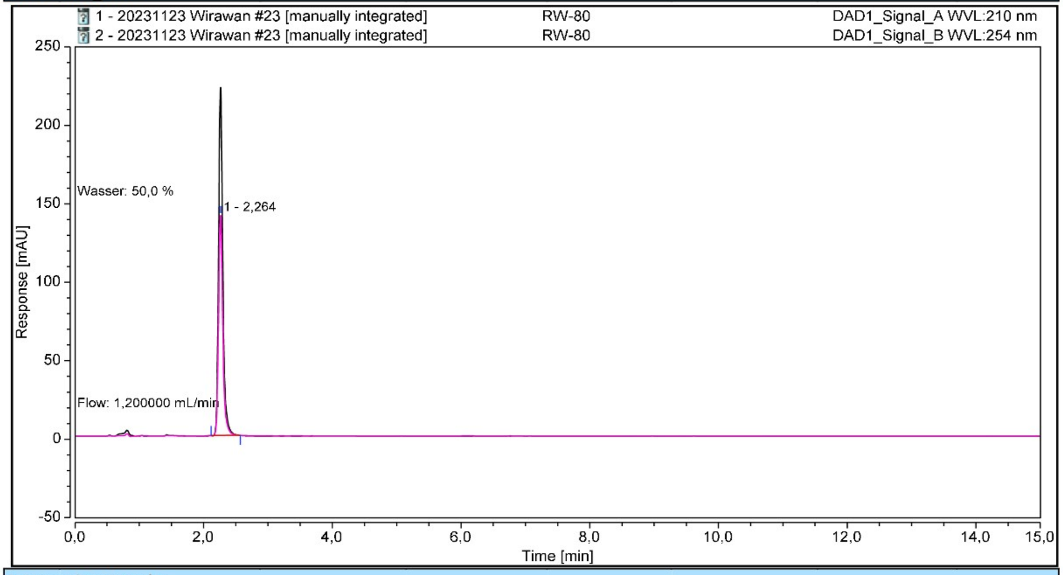

Integration Results

210nm

No.	Peak Name	Retention Time min	Area mAU*min	Relative Area %
1		2,264	17,110	100,00
**Total:**			**17,110**	**100,00**

254mn

No.	Peak Name	Retention Time min	Area mAU*min	Relative Area %
1		2,264	10,756	100,00
**Total:**			**10,756**	**100,00**

HPLC chromatogram of compound **32**

Chromatogram and Results

Injection Details

Injection Name:	**RW-64 DMSO /ACN**	*Run Time (min):*	**9,99**
Vial Number:	**Vial:23**	*Injection Volume:*	**5,00**
Injection Type:	**Unknown**	*Wavelength A:*	**210**
Column:	**Zorbax SB C18 3,5µm 4,6x100mm 861953-902**	*Wavelength B:*	**254**
Instrument Method:	**50 AcN 50 Wasser**		
Processing Method:	**SG-094**	*Flow rate:*	**1,200 mL/min**
Injection Date/Time:	**19.Jul.23 11:03**	*Column Temperatu*	**35,0 °C**
Pump Channel A:	**50,00** AcN		
Pump Channel B:			
Pump ChannelC:			
Pump Channel D:	**50** Wasser		

Chromatogram

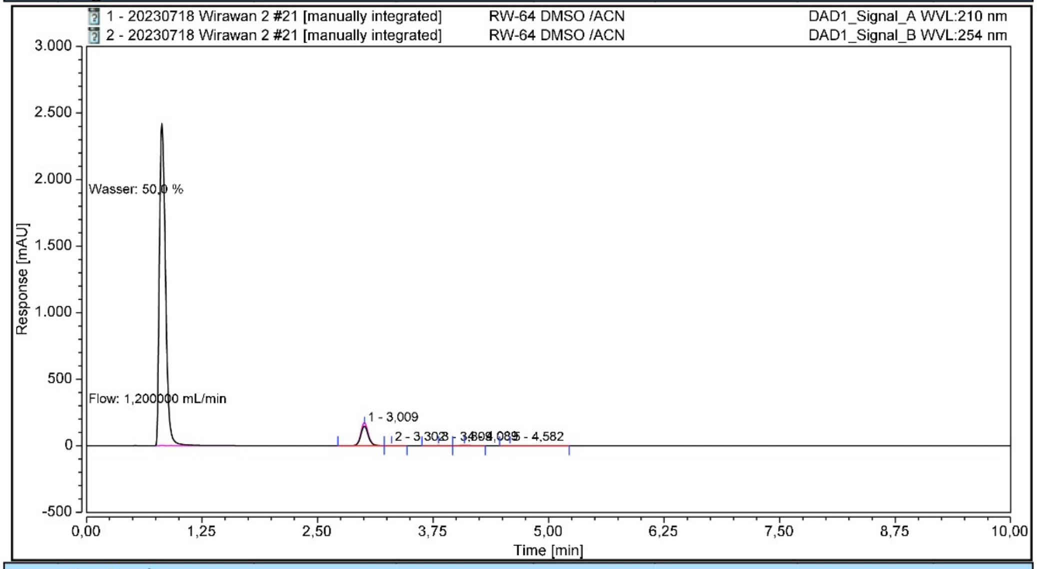

Integration Results

210nm

No.	Peak Name	Retention Time min	Area mAU*min	Relative Area %
1		3,009	13,767	96,39
2		3,302	0,071	0,50
3		3,809	0,122	0,85
4		4,089	0,233	1,63
5		4,582	0,090	0,63
**Total:**			**14,282**	**100,00**

254mn

No.	Peak Name	Retention Time min	Area mAU*min	Relative Area %
1		3,009	16,108	98,63
n.a.	n.a.	n.a.	n.a.	n.a.
2		3,816	0,072	0,44
3		4,089	0,151	0,93

HPLC chromatogram of compound **33**

Chromatogram and Results

Injection Details

Injection Name:	**RW-46 in MeOH + Wasser**	*Run Time (min):*	**9,99**	
Vial Number:	**Vial:13**	*Injection Volume:*	**5,00**	
Injection Type:	**Unknown**	*Wavelength A:*	**210**	
Column:	**Raptor C18 5µm 4,6x150mm**	*Wavelength B:*	**254**	
Instrument Method:	**35 AcN 65 Wasser**			
Processing Method:	**SG-094**	*Flow rate:*	**0,700**	**mL/min**
Injection Date/Time:	**25.Jul.23 12:13**	*Column Temperatu*	**35,0**	**°C**
Pump Channel A:	**35,00** AcN			
Pump Channel B:				
Pump ChannelC:				
Pump Channel D:	**65** Wasser			

Chromatogram

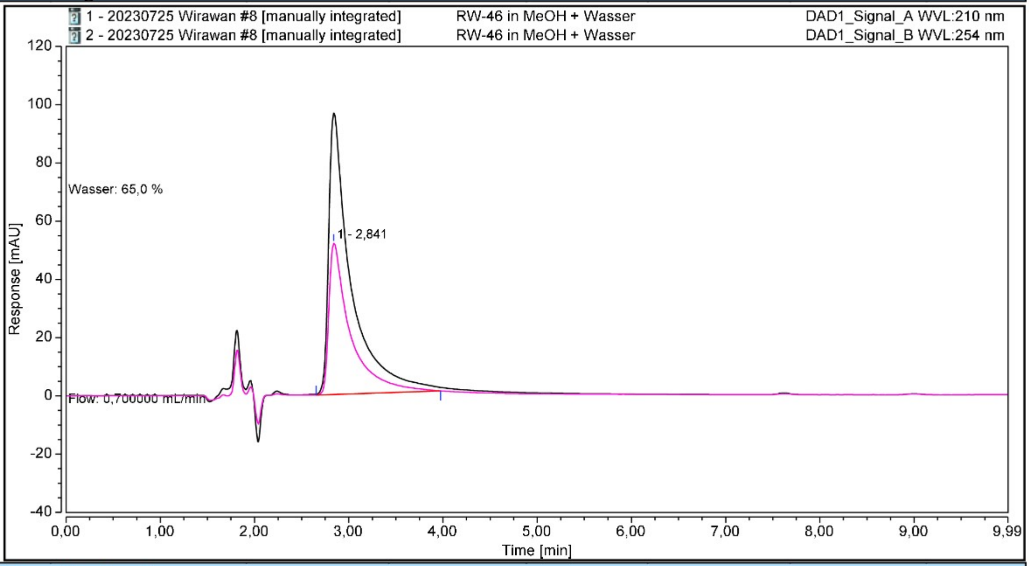

Integration Results

210nm

No.	Peak Name	Retention Time min	Area mAU*min	Relative Area %
1		2,841	25,538	100,00
**Total:**			**25,538**	**100,00**

254mn

No.	Peak Name	Retention Time min	Area mAU*min	Relative Area %
1		2,841	13,799	100,00
**Total:**			**13,799**	**100,00**

HPLC chromatogram of compound **34**

Chromatogram and Results

Injection Details

Injection Name:	**RW-61**	*Run Time (min):*	**24,99**
Vial Number:	**Vial:5**	*Injection Volume:*	**5,00**
Injection Type:	**Unknown**	*Wavelength A:*	**210**
Column:	**Zorbax SB C18 3,5µm 4,6x100mm 861953-902**	*Wavelength B:*	**254**
Instrument Method:	**35 AcN 65 Wasser**		
Processing Method:	**SG-094**	*Flow rate:*	**1,000 mL/min**
Injection Date/Time:	**18.Jul.23 20:03**	*Column Temperatu*	**35,0 °C**
Pump Channel A:	**35,00** AcN		
Pump Channel B:			
Pump ChannelC:			
Pump Channel D:	**65** Wasser		

Chromatogram

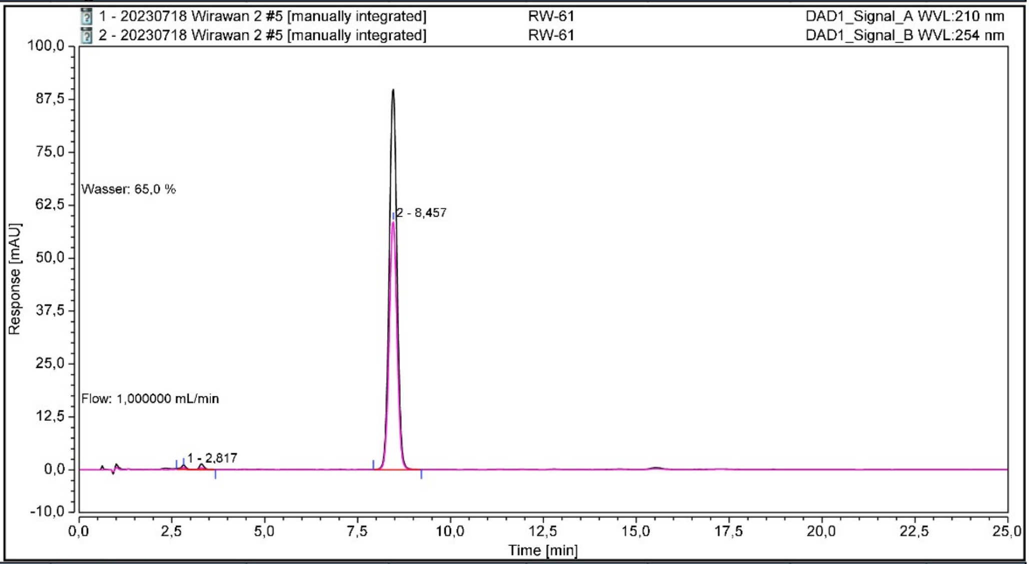

Integration Results

210nm

No.	Peak Name	Retention Time min	Area mAU*min	Relative Area %
1		2,817	0,103	0,47
3		8,457	21,893	98,73
**Total:**			**21,996**	**99,19**

254mn

No.	Peak Name	Retention Time min	Area mAU*min	Relative Area %
1		2,817	0,081	0,56
2		8,457	14,282	99,44
**Total:**			**14,363**	**100,00**

HPLC chromatogram of compound **46**

Chromatogram and Results

Injection Details

Injection Name:	**fm 295**	*Run Time (min):*	**14,99**
Vial Number:	**Vial:4**	*Injection Volume:*	**10,00**
Injection Type:	**Unknown**	*Wavelength A:*	**210**
Column:	**Eclipse Plus C18 5µm 4,6x 150mm USUXB17231**	*Wavelength B:*	**254**
Instrument Method:	**50 AcN 50 Wassser**		
Processing Method:	**SG-094**	*Flow rate:*	**1,200** mL/min
Injection Date/Time:	**24.Mrz.23 16:30**	*Column Temperatu*	**50,0** °C
Pump Channel A:	**50,00** Acetonitril		
Pump Channel B:			
Pump ChannelC:	Phosphatpuffer pH 5		
Pump Channel D:	**50** Wasser		

Chromatogram

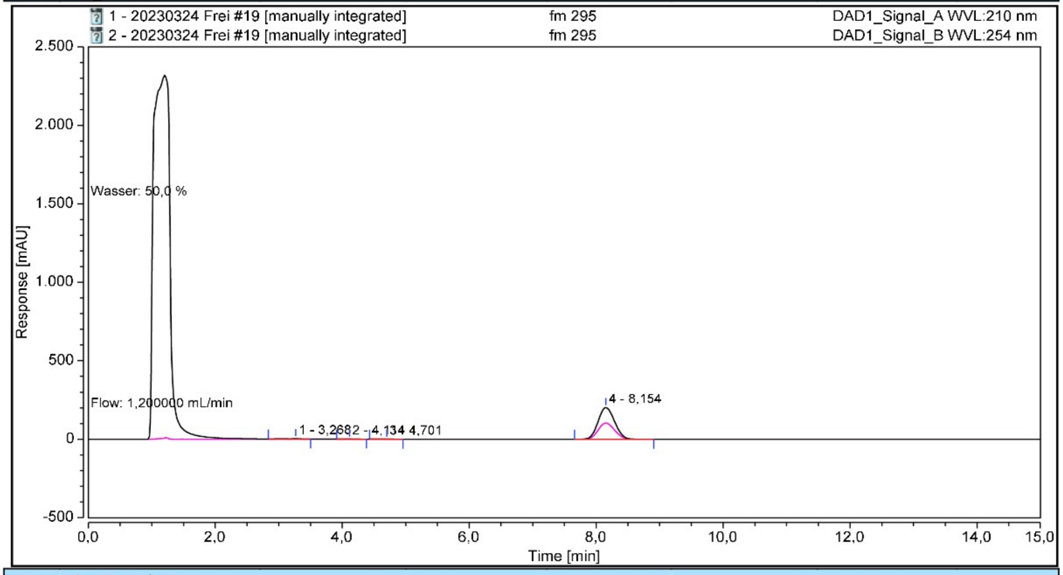

Integration Results

210nm

No.	Peak Name	Retention Time min	Area mAU*min	Relative Area %
1		3,268	0,675	1,10
2		4,114	0,229	0,37
3		4,701	0,073	0,12
4		8,154	60,603	98,41
**Total:**			**61,579**	**100,00**

254mn

No.	Peak Name	Retention Time min	Area mAU*min	Relative Area %
1		3,268	0,247	0,79
2		4,114	0,060	0,19
n.a.	n.a.	n.a.	n.a.	n.a.
4		8,154	31,011	98,91

Chromeleon (c) Dionex
Version 7.2.9.11323

HPLC chromatogram of compound **47**

Chromatogram and Results

Injection Details

Injection Name:	**fm 302**	*Run Time (min):*	**9,99**
Vial Number:	**Vial:6**	*Injection Volume:*	**10,00**
Injection Type:	**Unknown**	*Wavelength A:*	**210**
Column:	**Eclipse Plus C18 5µm 4,6x 150mm USUXB17231**	*Wavelength B:*	**254**
Instrument Method:	**50 AcN 50 Wassser**		
Processing Method:	**SG-094**	*Flow rate:*	**1,200 mL/min**
Injection Date/Time:	**24.Mrz.23 14:34**	*Column Temperatu*	**50,0 °C**
Pump Channel A:	**50,00** Acetonitril		
Pump Channel B:			
Pump ChannelC:	Phosphatpuffer pH 5		
Pump Channel D:	**50** Wasser		

Chromatogram

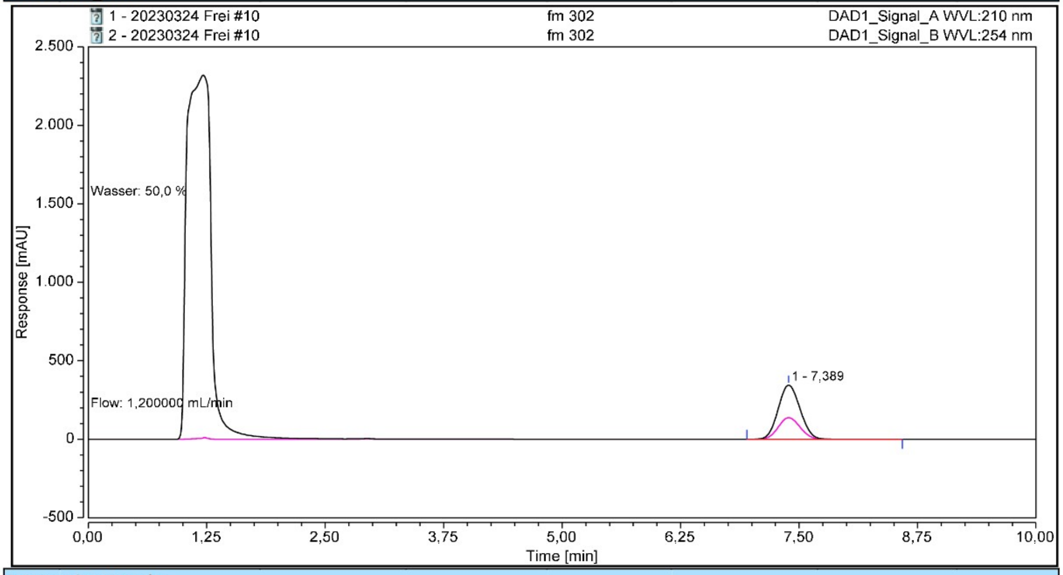

Integration Results

210nm

No.	Peak Name	Retention Time min	Area mAU*min	Relative Area %
1		7,389	91,840	100,00
**Total:**			**91,840**	**100,00**

254mn

No.	Peak Name	Retention Time min	Area mAU*min	Relative Area %
1		7,389	36,535	100,00
**Total:**			**36,535**	**100,00**

HPLC chromatogram of compound **48**

Chromatogram and Results

Injection Details

Injection Name:	**fm 320 Acn**	*Run Time (min):*	**9,99**
Vial Number:	**Vial:11**	*Injection Volume:*	**10,00**
Injection Type:	**Unknown**	*Wavelength A:*	**210**
Column:	**Eclipse Plus C18 5µm 4,6x 150mm USUXB17231**	*Wavelength B:*	**254**
Instrument Method:	**50 AcN 50 Wassser**		
Processing Method:	**SG-094**	*Flow rate:*	**1,200** **mL/min**
Injection Date/Time:	**24.Mrz.23 13:19**	*Column Temperatu*	**50,0** **°C**
Pump Channel A:	**50,00** Acetonitril		
Pump Channel B:			
Pump ChannelC:	Phosphatpuffer pH 5		
Pump Channel D:	**50** Wasser		

Chromatogram

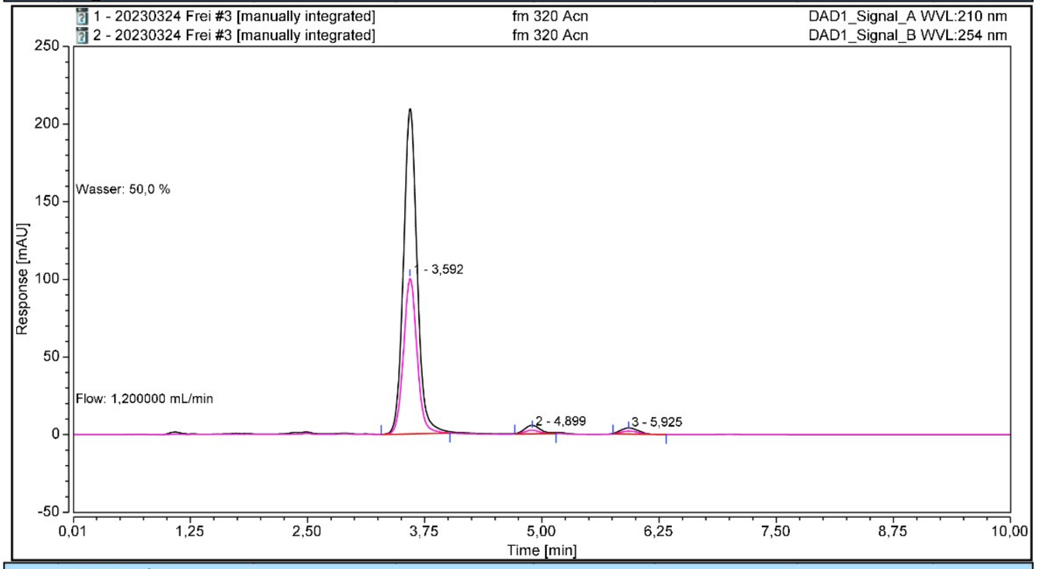

Integration Results

210nm

No.	Peak Name	Retention Time min	Area mAU*min	Relative Area %
1		3,592	34,769	94,75
2		4,899	0,988	2,69
3		5,925	0,939	2,56
**Total:**			**36,696**	**100,00**

254mn

No.	Peak Name	Retention Time min	Area mAU*min	Relative Area %
1		3,592	16,757	95,42
2		4,899	0,407	2,32
3		5,925	0,398	2,26
**Total:**			**17,562**	**100,00**

HPLC chromatogram of compound **49**

Chromatogram and Results

Injection Details

Injection Name:	**fm 316**	*Run Time (min):*	**14,99**
Vial Number:	**Vial:10**	*Injection Volume:*	**10,00**
Injection Type:	**Unknown**	*Wavelength A:*	**210**
Column:	**Eclipse Plus C18 5µm 4,6x 150mm USUXB17231**	*Wavelength B:*	**254**
Instrument Method:	**50 AcN 50 Wassser**		
Processing Method:	**SG-094**	*Flow rate:*	**1,200** **mL/min**
Injection Date/Time:	**24.Mrz.23 18:04**	*Column Temperatu*	**50,0** **°C**
Pump Channel A:	**50,00** Acetonitril		
Pump Channel B:			
Pump ChannelC:	Phosphatpuffer pH 5		
Pump Channel D:	**50** Wasser		

Chromatogram

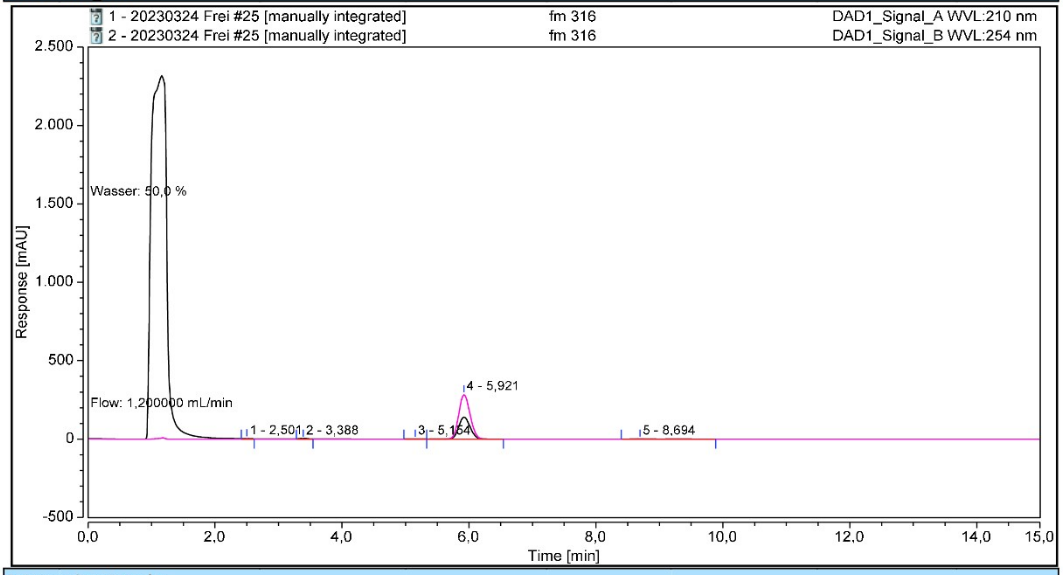

Integration Results

210nm

No.	Peak Name	Retention Time min	Area mAU*min	Relative Area %
1		2,494	0,172	0,57
2		3,388	0,512	1,69
3		5,147	0,173	0,57
4		5,921	28,811	94,83
5		8,694	0,712	2,34
**Total:**			**30,380**	**100,00**

254mn

No.	Peak Name	Retention Time min	Area mAU*min	Relative Area %
1		2,501	0,205	0,35
2		3,388	0,064	0,11
3		5,154	0,041	0,07

4		5,921	58,581	98,84
5		8,694	0,375	0,63
**Total:**			**59,267**	**100,00**

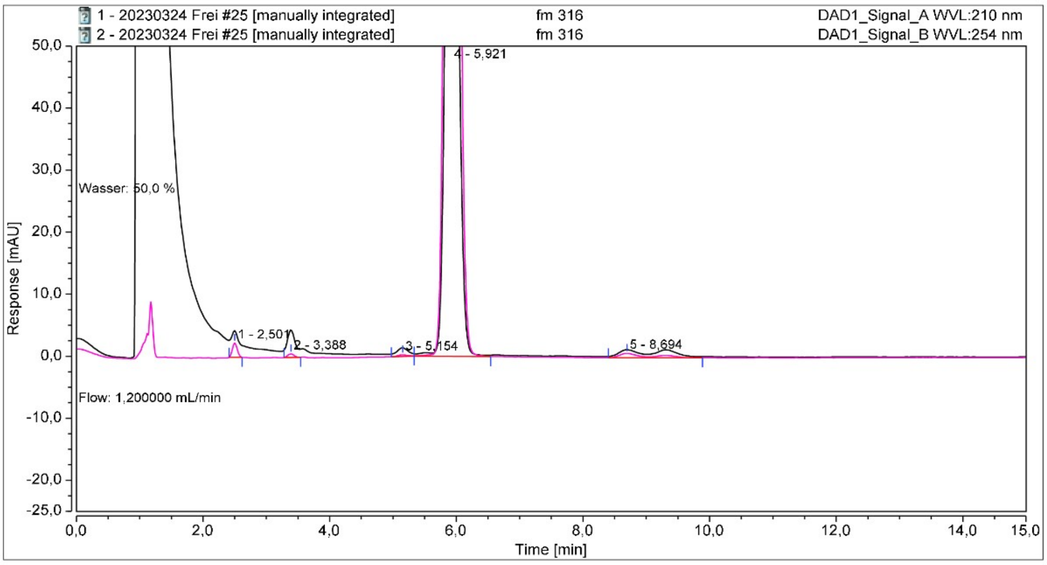

Table S1. Crystallographic data collection and refinement statistics.

	**SIRT2**
**Crystal parameters**	
Space group	$P2_1$
Cell constants	a = 35.8 Å
	b = 73.5 Å
	c = 55.0 Å
	β = 95.3°
**Data collection**	
Beam line	P13, PETRA III, DESY
Wavelength (Å)	1.060
Resolution range (Å)[b]	30.0-2.15 (2.25-2.15)
No. observations	51,769
No. unique reflections[c]	15,167
Completeness (%)[b]	97.8 (98.6)
R_{merge} (%)[b,d]	8.7 (68.8)
I/σ (I)[b]	9.2 (2.6)
**Refinement (REFMAC5)**	
Resolution range (Å)	30-1.25
No. refl. working set	14,404
No. refl. test set	758
No. non hydrogen	2251
No. of ligand atoms	1
Solvent	39
R_{work}/R_{free} (%)[e]	22.8 / 27.4
r.m.s.d. bond (Å) / angle (°)[f]	0.002 / 1.2
Average B-factor (Å^2)	47.4
Ramachandran Plot (%)[g]	97.8 / 2.2 / 0
PDB accession code	**9S44**

[a] Asymmetric unit

[b] The values in parentheses for resolution range, completeness, R_{merge} and I/σ (I) correspond to the highest resolution shell

[c] Data reduction was carried out with XDS and from a single crystal. Friedel pairs were treated as identical reflections

[d] $R_{merge}(I) = \Sigma_{hkl}\Sigma_j \mid I(hkl)_j - \langle I(hkl)\rangle \mid / \Sigma_{hkl} \Sigma_j I(hkl)_j$, where $I(hkl)_j$ is the jth measurement of the intensity of reflection hkl and <I(hkl)> is the average intensity

[e] $R = \Sigma_{hkl} \mid |F_{obs}| - |F_{calc}| \mid / \Sigma_{hkl} |Fobs|$, where R_{free} is calculated without a sigma cut off for a randomly chosen 5% of reflections, which were not used for structure refinement, and R_{work} is calculated for the remaining reflections

[f] Deviations from ideal bond lengths/angles

[g] Percentage of residues in favored region / allowed region / outlier region

Table S2. Crystallographic data collection and refinement statistics.

	**SIRT2:RW-78**
**Crystal parameters**	
Space group	$P2_1$
Cell constants	a = 35.8 Å<br>b = 73.7 Å<br>c = 55.9 Å<br>β = 94.8°
**Data collection**	
Beam line	P13, PETRA III, DESY
Wavelength (Å)	1.060
Resolution range (Å)[b]	30.0-1.45<br>(1.55-1.45)
No. observations	175,238
No. unique reflections[c]	50,844
Completeness (%)[b]	99.1 (99.0)
R_{merge} (%)[b,d]	8.8 (67.2)
I/σ (I)[b]	7.9 (1.9)
**Refinement (REFMAC5)**	
Resolution range (Å)	30-1.45
No. refl. working set	48,299
No. refl. test set	2,542
No. non hydrogen	2,653
No. of ligand atoms	62
Solvent	215
R_{work}/R_{free} (%)[e]	15.7 / 19.0
r.m.s.d. bond (Å) / angle (°)[f]	0.003 / 1.2
Average B-factor ($Å^2$)	19.3
Ramachandran Plot (%)[g]	98.6 / 1.4 / 0
PDB accession code	**9S46**

[a] Asymmetric unit

[b] The values in parentheses for resolution range, completeness, R_{merge} and I/σ (I) correspond to the highest resolution shell

[c] Data reduction was carried out with XDS and from a single crystal. Friedel pairs were treated as identical reflections

[d] $R_{merge}(I) = \Sigma_{hkl}\Sigma_j \mid I(hkl)_j - \langle I(hkl)\rangle \mid / \Sigma_{hkl} \Sigma_j I(hkl)_j$, where $I(hkl)_j$ is the jth measurement of the intensity of reflection hkl and <I(hkl)> is the average intensity

[e] $R = \Sigma_{hkl} \mid |F_{obs}| - |F_{calc}| \mid / \Sigma_{hkl} |Fobs|$, where R_{free} is calculated without a sigma cut off for a randomly chosen 5% of reflections, which were not used for structure refinement, and R_{work} is calculated for the remaining reflections

[f] Deviations from ideal bond lengths/angles

[g] Percentage of residues in favored region / allowed region / outlier region

Table S3. Crystallographic data collection and refinement statistics.

	**SIRT2:RW-80**
**Crystal parameters**	
Space group	$P2_1$
Cell constants	a = 35.9 Å
	b = 73.7 Å
	c = 55.9 Å
	β = 94.8 °
**Data collection**	
Beam line	P13, PETRA III, DESY
Wavelength (Å)	1.060
Resolution range (Å)[b]	30.0-1.45
	(1.55-1.45)
No. observations	176,079
No. unique reflections[c]	49,669
Completeness (%)[b]	96.7 (95.0)
R_{merge} (%)[b,d]	7.9 (78.8)
I/σ (I)[b]	9.5 (2.0)
**Refinement (REFMAC5)**	
Resolution range (Å)	30-1.45
No. refl. working set	47,182
No. refl. test set	2,483
No. non hydrogen	2,621
No. of ligand atoms	70
Solvent	241
R_{work}/R_{free} (%)[e]	15.6 / 18.7
r.m.s.d. bond (Å) / angle (°)[f]	0.003 / 1.2
Average B-factor ($Å^2$)	18.6
Ramachandran Plot (%)[g]	98.2 / 1.8 / 0
PDB accession code	**9S48**

[a] Asymmetric unit

[b] The values in parentheses for resolution range, completeness, R_{merge} and I/σ (I) correspond to the highest resolution shell

[c] Data reduction was carried out with XDS and from a single crystal. Friedel pairs were treated as identical reflections

[d] $R_{merge}(I) = \Sigma_{hkl}\Sigma_j \mid I(hkl)_j - \langle I(hkl)\rangle \mid / \Sigma_{hkl} \Sigma_j I(hkl)_j$, where $I(hkl)_j$ is the jth measurement of the intensity of reflection hkl and <I(hkl)> is the average intensity

[e] $R = \Sigma_{hkl} \mid |F_{obs}| - |F_{calc}| \mid / \Sigma_{hkl} |Fobs|$, where R_{free} is calculated without a sigma cut off for a randomly chosen 5% of reflections, which were not used for structure refinement, and R_{work} is calculated for the remaining reflections

[f] Deviations from ideal bond lengths/angles

[g] Percentage of residues in favored region / allowed region / outlier region

Table S4. DNA sequence of *SIRT2* 2-389 as ordered and subcloned by Eurofins Genomics.

Construct	DNA sequence
SIRT2 2-389	GCAGAGCCAGACCCCTCTCACCCTCTGGAGACCCAGGCAGGGAAG<br>GTGCAGGAGGCTCAGGACTCAGATTCAGACTCTGAGGGAGGAGCC<br>GCTGGTGGAGAAGCAGACATGGACTTCCTGCGGAACTTATTCTCCC<br>AGACGCTCAGCCTGGGCAGCCAGAAGGAGCGTCTGCTGGACGAGC<br>TGACCTTGGAAGGGGTGGCCCGGTACATGCAGAGCGAACGCTGTC<br>GCAGAGTCATCTGTTTGGTGGGAGCTGGAATCTCCACATCCGCAGG<br>CATCCCCGACTTTCGCTCTCCATCCACCGGCCTCTATGACAACCTA<br>GAGAAGTACCATCTTCCCTACCCAGAGGCCATCTTTGAGATCAGCTA<br>TTTCAAGAAACATCCGGAACCCTTCTTCGCCCTCGCCAAGGAACTCT<br>ATCCTGGGCAGTTCAAGCCAACCATCTGTCACTACTTCATGCGCCT<br>GCTGAAGGACAAGGGGCTACTCCTGCGCTGCTACACGCAGAACATA<br>GATACCCTGGAGCGAATAGCCGGGCTGGAACAGGAGGACTTGGTG<br>GAGGCGCACGGCACCTTCTACACATCACACTGCGTCAGCGCCAGCT<br>GCCGGCACGAATACCCGCTAAGCTGGATGAAAGAGAAGATCTTCTC<br>TGAGGTGACGCCCAAGTGTGAAGACTGTCAGAGCCTGGTGAAGCCT<br>GATATCGTCTTTTTTGGTGAGAGCCTCCCAGCGCGTTTCTTCTCCTG<br>TATGCAGTCAGACTTCCTGAAGGTGGACCTCCTCCTGGTCATGGGT<br>ACCTCCTTGCAGGTGCAGCCCTTTGCCTCCCTCATCAGCAAGGCAC<br>CCCTCTCCACCCCTCGCCTGCTCATCAACAAGGAGAAAGCTGGCCA<br>GTCGGACCCTTTCCTGGGGATGATTATGGGCCTCGGAGGAGGCAT<br>GGACTTTGACTCCAAGAAGGCCTACAGGGACGTGGCCTGGCTGGG<br>TGAATGCGACCAGGGCTGCCTGGCCCTTGCTGAGCTCCTTGGATG<br>GAAGAAGGAGCTGGAGGACCTTGTCCGGAGGGAGCACGCCAGCAT<br>AGATGCCCAGTCGGGGGCGGGGGTCCCCAACCCCAGCACTTCAGC<br>TTCCCCCAAGAAGTCCCCGCCACCTGCCAAGGACGAGGCCAGGAC<br>AACAGAGAGGGAGAAACCCCAGTGA

Table S5. Primers used to generate His$_6$-SUMO-Ser-*SIRT2* 56-356 by Q5-mutagenesis.

ID	Primer name	DNA-sequence
A	SIRT2_2-356_fw	TGACTGCAGGTCGAC
B	SIRT2_2-356_rv	CGACTGGGCATCTATG
C	SIRT2 _56-356_fw	GAGCGTCTGCTGGAC
D	SIRT2_56-356_rv	GGATCCACCGATCTG

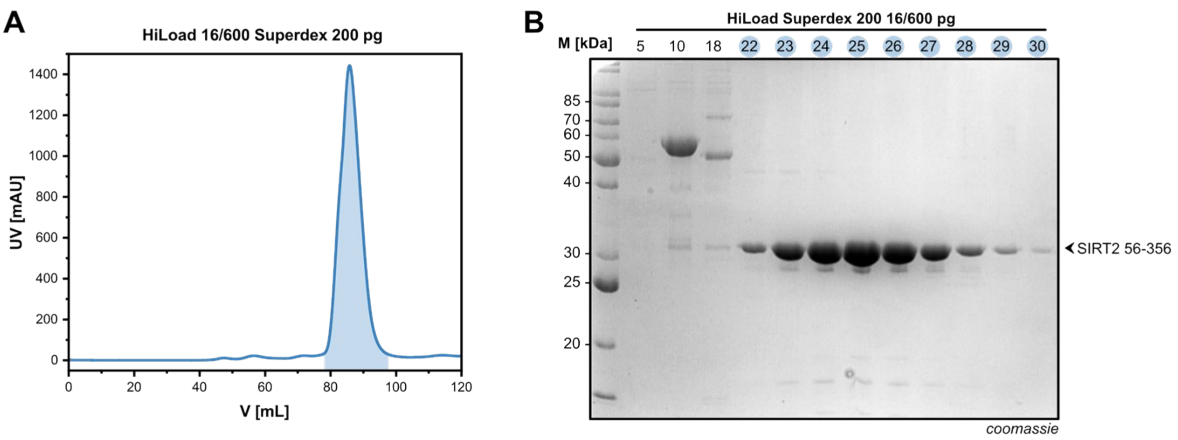

Fig. S1. Purification of human SIRT2 56-356. (**A**) Size exclusion chromatography profile of SIRT2 56-356 on a HiLoad® 16/600 Superdex® 200 pg column. (**B**) SDS-PAGE of fractions from the size exclusion chromatography. Blue fractions correspond to blue shaded peak area in panel A.

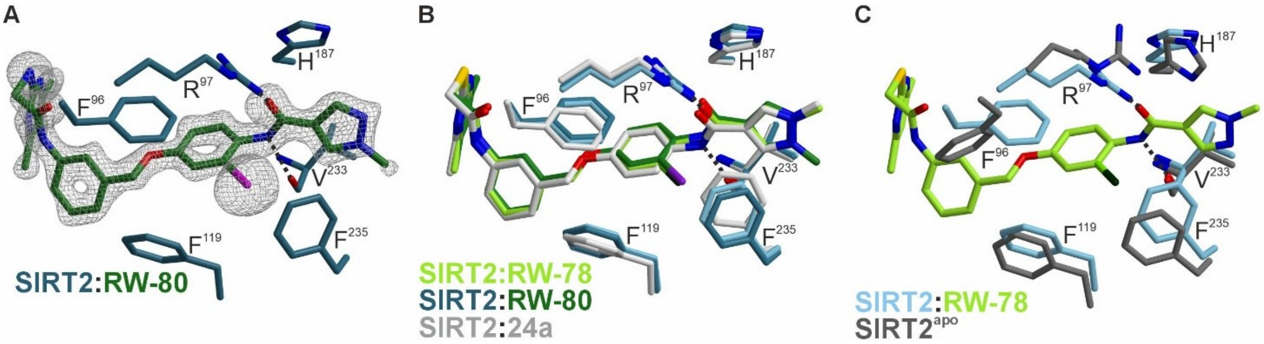

Fig. S2. Close-up view of the SIRT2 ligand binding site. Hydrogen bonds are shown as black dotted lines. Key amino acids are visualised as sticks and labelled by the one-letter code. (**A**) F_O-F_C omit electron density (grey mesh contoured to 3 σ) of **31** (**RW-80**, dark green) bound to SIRT2 (PDB ID: 9S48; blue-green). (**B**) Superposition of inhibitors **10** (**24a** (PDB ID: 5YQO)[1], white), **29** (**RW-78**, green, PDB ID: 9S46), and **31** (**RW-80**, dark green, PDB ID: 9S48) in their respective SIRT2 binding pocket. All three inhibitors are located similarly in the binding cleft. (**C**) Comparison of the inhibitor binding site in the SIRT2:**29** complex (**RW-78**, green) and the SIRT2 apo structure (grey residues, PDB ID: 9S44). Depicted residues undergo rearrangements upon ligand binding.

1. L. L. Yang, H. L. Wang, L. Zhong, C. Yuan, S. Y. Liu, Z. J. Yu, S. Liu, Y. H. Yan, C. Wu, Y. Wang, Z. Wang, Y. Yu, Q. Chen and G. B. Li, *Eur J Med Chem*, 2018, **155**, 806-823.
